# N‐Heterocyclic Carbene Organocatalysis: With or Without Carbenes?

**DOI:** 10.1002/chem.202002656

**Published:** 2020-07-23

**Authors:** Sascha Gehrke, Oldamur Hollóczki

**Affiliations:** ^1^ Mulliken Center for Theoretical Chemistry University of Bonn Beringstr. 4+6 53115 Bonn Germany

**Keywords:** density functional calculations, N-heterocyclic carbenes, organocatalysis, reaction mechanisms, umpolung

## Abstract

In this work the mechanism of the aldehyde umpolung reactions, catalyzed by azolium cations in the presence of bases, was studied through computational methods. Next to the mechanism established by Breslow in the 1950s that takes effect through the formation of a free carbene, we have suggested that these processes can follow a concerted asynchronous path, in which the azolium cation directly reacts with the substrate, avoiding the formation of the carbene intermediate. We hereby show that substituting the azolium cation, and varying the base or the substrate do not affect the preference for the concerted reaction mechanism. The concerted path was found to exhibit low barriers also for the reactions of thiamine with model substrates, showing that this path might have biological relevance. The dominance of the concerted mechanism can be explained through the specific structure of the key transition state, avoiding the liberation of the highly reactive, and thus unstable carbene lone pair, whereas activating the substrate through hydrogen‐bonding interactions. Polar and hydrogen‐bonding solvents, as well as the presence of the counterions of the azolium salts facilitate the reaction through carbenes, bringing the barriers of the two reaction mechanisms closer, in many cases making the concerted path less favorable. Thus, our data show that by choosing the exact components in a reaction, the mechanism can be switched to occur with or without carbenes.

## Graphical abstract

**With or without**: N‐Heterocyclic carbene organocatalysis can proceed either via free carbenes in the solution, or through a concerted asynchronous reaction directly from the azolium cation. The factors that control the mechanism are discussed in detail.

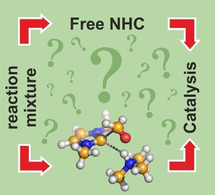

## Introduction

Since the discovery of thiamine (vitamin B1),^1^, ^2^ the mechanism of the biological processes it catalyzes have been in the focus of research. Next to the direct biochemical studies that revealed the role of this substance in life, numerous organic chemical model reactions have been designed to understand these reactions in further depth. The synthetic value of the knowledge that was gathered through these organic chemical studies was recognized by Stetter et al.,^3^ defining the field that we call today N‐heterocyclic carbene (NHC) organocatalysis.^4^, ^5^, ^6^, ^7^, ^8^ Since then, these transformations offer an ever growing portfolio of reactions, including important C−C coupling reactions, which can be often performed in a selective and asymmetric manner.

**Figure 1:**
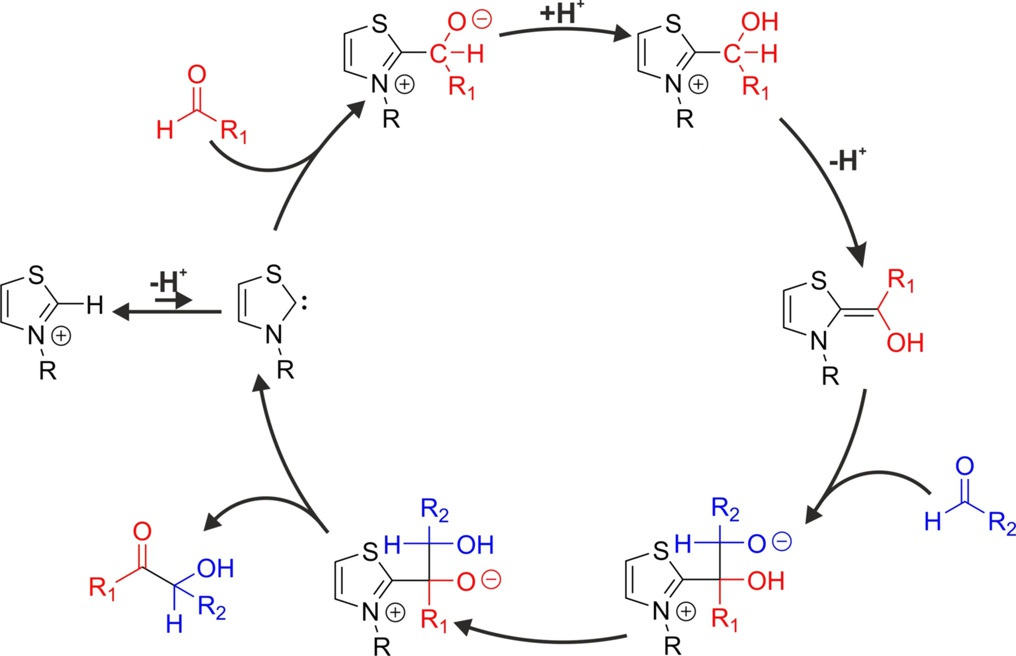
Catalytic cycle of the benzoin condensation, as suggested by Breslow in 1958.^15^

One of the most prominent examples for NHC organocatalysis is benzoin condensation, catalyzed by azolium salts in basic media. This reaction was discovered independently as an unexpected side reaction of decarboxylases in in vitro experiments,^9^ and in organic chemical research of thiazolium salts.^10^ Subsequently, the biological relevance of this process in the carbohydrate metabolism was discovered. In the underlying biochemical reactions an H_4_C_2_O_2_ unit is transferred between sugar molecules, enabling the transformation of various carbohydrates into each other.^11^, ^12^ Due to the importance and simplicity of this reaction, it became a workhorse for later studies, through which the mechanism of NHC organocatalysis was investigated. Breslow observed that in deuterated solvents the position 2 proton of thiazolium salts is spontaneously exchanged to a deuteron, inferring the mobility of this proton.^13^ Assuming that this mobility meant that the deprotonated species, an NHC, was actually present in the solution in a small quantity as an intermediate, he adjusted the mechanism of cyanide‐catalyzed benzoin condensation^14^ to interpret the catalytic effect of thiazolium salts.^15^ Through suggesting the formation of another key intermediate (called later Breslow intermediate), he defined a mechanistic picture that is today still the main school of thought in NHC organocatalysis (Figure 1).

The use of this mechanism as a template has allowed explaining and predicting selectivity in many related processes and understanding the role of structural features in the catalysts,^16^, ^17^ which strongly supports the aforementioned reaction path. Computational studies predict low barriers for the reaction of free carbenes with various substrates.^17^ Furthermore, Breslow intermediates^18^, ^19^, ^20^ and related compounds^19^, ^21^, ^22^ have been synthesized and structurally characterized. Similarly, several free NHCs have been shown to be stable,^23^, ^24^, ^25^, ^26^, ^27^ and extraordinary persistence has been evidenced for several of their derivatives.^23^, ^27^ In some specific cases, the formation of NHCs has also been observed by mass spectrometry in vaporized reaction mixtures,^28^ vaporized catalytically active^29^ solutions,^30^ or confined within the active site of a thiamine‐dependent enzyme.^31^

However, it is important to stress that the direct evidence that free carbenes can catalyze these reactions does not mean they play an actual role in catalysis, and similarly, the stability of NHCs prepared in an isolated environment does not prove their formation in the reaction mixture. Throughout the decades doubt has been raised regarding the first part of the catalytic cycle,^32^, ^33^, ^34^, ^35^, ^36^, ^37^, ^38^, ^39^, ^40^ in which the bond between the azolium ring of the catalyst and the substrate is formed. In fact, already in this early step selectivity can become an issue, if multiple substrates are available in the reaction mixture, such as in cross‐benzoin condensations, in Stetter reactions, and in the biochemical transketolase reaction. Thus, understanding this initial reaction between substrate and catalyst is of high practical relevance.^4^, ^5^, ^6^, ^7^, ^8^

Washabaugh and Jencks measured the acidity of thiamine at its active site in aqueous solution to be p*K*_a_=18.0.^33^ They argued that the catalytic activity of this compound could be explained only with a p*K*_a_<14.^33^ To resolve this contradiction, they tentatively suggested that an alternative, concerted pathway might bypass the formation of the energetically quite unstable free NHC, in which the deprotonation occurs simultaneously with the catalyst−substrate bond formation.^33^ However, speculating that the substrate would need to approach the ring in the very direction, in which the proton should leave,^33^ they dismissed this idea. Finally, they suggested that the reason for the activity of this compound within the enzyme must be due to the stabilizing effect of the protein on the carbene, which shifts the local acid‐base equilibrium within the binding site.^33^

There are indeed indications that the NHC might be more stable within the active site of the enzyme,^31^ including the enhanced activity of thiamine in the presence of these proteins.^41^, ^42^, ^43^ However, the effect of the enzyme cannot explain how NHC organocatalytic reactions can be possible in organic syntheses in the absence of any biomolecule. Imidazolium (in water: p*K*_a_=19–24,^44^, ^45^, ^46^, ^47^, ^48^ in DMSO: p*K*_a_=19–24,^45^, ^49^ in MeCN: p*K*_a_=33.6^48^), triazolium (in water: p*K*_a_=14.9–17.4^50^) and thiazolium (in water: p*K*_a_=17–19,^33^, ^50^ in DMSO: p*K*_a_=14.5,^48^ in MeCN: p*K*_a_=25.6^48^) salts have all been applied as catalysts,^4^, ^5^, ^6^, ^7^, ^8^, ^51^ and despite their high p*K*_a_ values, triethylamine (in water: p*K*_a_=10.65,^52^, ^53^ in DMSO: pKa=9.0,^53^ in THF: p*K*_a_=12.5,^53^ in MeCN: p*K*_a_=12.5^53^) has been observed to be basic enough to deprotonate them in a quantity, which is sufficient to exhibit reasonable to excellent catalytic activity.^51^

**Figure 2:**
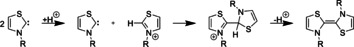
Dimerization reaction of thiazol‐2‐ylidenes in the presence of acid, reported by Arduengo and co‐workers.^26^

Although the aforementioned contradictions regarding the most fundamental acid–base theory should already be enough to raise questions, the general wisdom regarding the stability of NHCs points to further interesting issues. The hitherto synthesized stable free NHCs generally possess bulky substituents on the ring to prevent decomposition reactions, for example, dimerization, with the exception of some imidazol‐2‐ylidene derivatives.^54^ Carbene catalysts, on the other hand, showed remarkable activity with the smallest substituents without any significant decomposition, and larger functional groups are introduced merely to increase the stereoselectivity of the reaction.^4^, ^5^, ^6^, ^7^, ^8^ This is especially surprising regarding thiazolium salts. Thiazol‐2‐ylidenes showed a high propensity to dimerize in the presence of acid traces,^26^ even with the sizable 1,3‐diisopropylphenyl substituent on the nitrogen atoms. Strikingly, in the absence of acids, the same compounds did not dimerize. Arduengo and co‐workers argued that the dimerization takes place through the reaction of an NHC with a thiazolium cation (Figure 2). These findings can be rationalized as the protonation increases the electrophilicity of the C2‐carbon atom of the ring, facilitating thereby the nucleophilic attack of the NHC at the ring, which—after deprotonation—forms the NHC dimer. Should NHCs be present in the solution in the catalytic environment, the excess of the azolium cations should result in the dimers of the catalyst in an analogous process. Such decomposition reactions of thiazolium catalysts have not been reported. In fact, the role of these dimers in the catalytic mechanism has been discussed,^34^, ^35^, ^36^, ^37^, ^38^, ^39^ and it was subsequently dismissed.^55^, ^56^

For the carbene‐like reactions of ionic liquids with carbon dioxide, we discovered by static quantum chemical calculations a novel reaction mechanism, which avoids NHC formation,^57^ and the imidazolium cation reacts directly with the substrate. This mechanism was later observed also through ab initio molecular dynamics simulations.^58^ Similarly, we found an alternative pathway for NHC organocatalytic reactions,^40^ in which first a pre‐aggregation of the components occurs. The proton transfer from the azolium cation to the base and the C−C bond formation between the catalyst and the substrate take place in a single elementary step within that aggregate, avoiding thereby the formation of free NHC intermediates, analogously to the suggestion of Washabaugh and Jencks.^33^ We found that the barrier of the classical (dissociative) pathway that assumes the formation of the free NHC is by 20–30 kcal mol^−1^ higher than that of the concerted (associative) path.^40^ This mechanism can satisfyingly explain the contradictions above regarding NHC catalyst stability and acid–base equilibria, whereas suggesting that the base—being present at the catalyst−substrate bond formation step—might also be a site, through which selectivity can be enhanced.

Rico del Cerro et al. showed in a joint experimental–computational study that the H/D exchange of azolium salts follows a very similar associative mechanism, in which the formation of free carbenes is avoided.^59^ Nolan and co‐workers reported that the NHC–metal complexes from imidazolium salts and metal ions can form through an analogous path,^60^ in which the preparation or even the in situ generation of an NHC is unnecessary, allowing very simple synthetic routes to these practically highly important compounds.^60^, ^61^ Regarding the carbene‐like reactions of imidazolium acetate ionic liquids with glucose,^62^ we found indications that the concerted mechanism should be prevalent, as the aggregates that are required for the associative mechanism spontaneously occurred in ab initio molecular dynamics simulations,^63^ whereas the barriers of this mechanism were somewhat lower than that of the reaction through carbene intermediates.^64^ However, we found that in such an ionic liquid environment, and with the acetate anion as base the difference between these two mechanisms in barriers is lower,^64^ which implies that there might be experimental setups, in which the dissociative mechanism is more facile. Thus, to fully uncover the mechanistic details of NHC organocatalysis, further research is necessary.

Although considering this novel reaction mechanism has already given a deeper insight into the chemistry of azolium salts,^57^, ^59^, ^60^, ^61^, ^63^, ^64^ and resulted in actual applications,^60^, ^61^ exploiting this process in its full potential—by for example, introducing unprecedented ways to control selectivities—has not yet been achieved. To this end, hereby we aim to understand in detail the influential factors that facilitate or suppress this direct reaction mechanism, while assessing its relevance in catalytic systems. In this comprehensive work we discuss in detail the effect of possible azolium rings, substituents, solvents, bases, substrates, and counterions on this reaction.

## Computational Methods

**Static quantum chemical calculations**: All quantum chemical calculations were performed by the ORCA 4.0 program.^65^, ^66^ Geometry optimizations of the minima and transition states were undertaken by using the TPSSh functional^67^ with the D3‐BJ dispersion correction,^68^, ^69^ and the def2‐TZVPP basis set.^70^, ^71^ For the SCF cycle and the geometry optimization the tight convergence criteria were applied. The nature of the obtained stationary points was verified by making sure that the Hessian had no negative eigenvalues for minima, and a single one for transition states. Steepest descent optimizations were performed in both directions defined by the imaginary normal mode for each transition state to identify the minima the given transition state connects.

On the structures obtained by the DFT calculations, DLPNO‐CCSD(T) single‐point energies^72^, ^73^, ^74^ were calculated with the def2‐TZVPP and def2‐QZVPP basis sets^70^, ^71^ with tight settings for the localization. The obtained single point energies were extrapolated to the complete basis set limit.^75^ These electronic energies were then corrected to enthalpies and Gibbs free energies by using the thermochemical data obtained from the corresponding DFT frequency calculations. Gibbs free energies of solvation were calculated through the COSMO‐RS approach,^76^, ^77^ by using the BP‐TZVPD‐FINE method of the COSMOthrmX14 software^78^, ^79^ based on BP86/def2‐TZVPD calculations^70^, ^71^, ^80^ by the Turbomole program.^81^ Non‐covalent interaction analysis^82^, ^83^, ^84^ was performed by the Multiwfn 3.6 program package.^85^

**Classical molecular dynamics simulations**: For performing classical molecular dynamics simulations, the LAMMPS program was used.^86^ For modeling the 1,3‐dimethylimidazolium cation, the chloride and triflate anions the force field parameters by Canongia Lopes et al. were applied.^87^, ^88^ The organic solvents were described by the OPLS‐AA model,^89^ whereas for water the SPC/E parameters were chosen.^90^ In the simulations, periodic boundary conditions were applied. The starting geometry of the cubic simulation box was created by the Packmol program.^91^ The initial cell vector of the simulation box was chosen according to the density of the pure solvent in question. After a geometry optimization, 1 ns simulations in the NpT ensemble have been performed, by using Nosé‐Hoover chain thermostats and barostats at a temperature of 293 K and under 1 bar pressure. The timestep was chosen to be 1 fs. The volume of the periodic simulation box was averaged over the last 0.5 ns, and this value was used for the later steps of the simulations. An external harmonic potential was added to the system that kept the distance between centers of mass of the anion and the cation at a value of 3 Å. After 100 ps of equilibration, the force that was required to maintain the cation‐anion distance was measured, and averaged over a production run of 250 ps. The anion‐cation distance was then increased by an increment of 0.5 Å until 20 Å, repeating the equilibration and production runs at each distance. Integrating the forces versus the distance gave the free energy profile of ion pair separation.

## Results and Discussion

**Figure 3:**
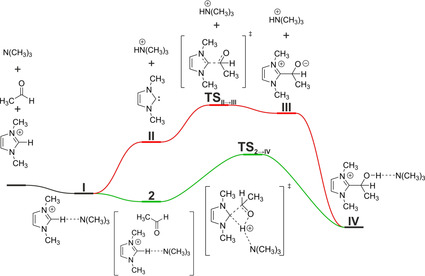
The general Gibbs free energy profile of the dissociative (red) and associative (green) reaction mechanisms of the catalyst−substrate bond formation in the benzoin condensation, and the labeling of the different species as used throughout the article.

In this paper, we compare the two reaction mechanisms for the initial steps of NHC‐catalyzed benzoin condensation, in which the catalyst−substrate bond is formed. The classical, dissociative reaction mechanism (Figure 3, red curve) follows the original proposal by Breslow.^15^ First a free NHC intermediate **II** is formed in the reaction mixture from the azolium cation by a proton transfer to a base (e.g., an amine), which reacts with the substrate, giving primary complex **III**. Concluding the formation of the catalyst−substrate bond, **III** is protonated again by the ammonium cation, yielding **IV**. This mechanism will be referred to as dissociative mechanism. The key to this path is the availability of the free NHC intermediate, which is present in the solution in a low concentration, dissociated from the rest of the components.

In contrast, in the associative path^40^ (Figure 3, green curve) the free NHC is not an intermediate of the reaction. Instead, the aggregation of the azolium catalyst, substrate, and base occurs, producing complex **2**. The bond formation between the catalyst and the substrate is undertaken through transition state **TS_2_**_→**IV**_ by a rearrangement within aggregate **2** in a single elementary step. This step comprises the C‐to‐N proton transfer from the azolium cation to the base, the C−C bond formation between the catalyst and the substrate, and the N‐to‐O proton transfer from the conjugate acid of the base (e.g., ammonium ion) to the oxygen atom of the substrate to give **IV** in a concerted asynchronous manner. An animation for the reaction mechanism is available in the Supporting Information. In this mechanism, the role of the base is to shuttle the proton from the carbon atom of the catalyst to the oxygen atom of the substrate, instead of releasing a free NHC intermediate. Formed by either of the two mechanisms, **IV** will transform to yield the Breslow intermediate, which can thereafter react with another substrate molecule in the subsequent reaction steps.

**Figure 4:**
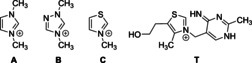
Lewis structures of the catalysts 1,3‐dimethylimidazolium (**A**) 1,4‐dimethyltriazolium (**B**), and 3‐methyltriazolium cation (**C**). The active tautomer of thiamine (**T**) is also shown, having a basic imino group in the vicinity of the mobile proton of the thiazolium ring.

The reaction mechanisms were calculated first for the three azolium catalysts that have been applied in NHC organocatalysis (imidazolium (**A**), triazolium (**B**), and thiazolium (**C**), see Figure 4) substituted with methyl groups on the relevant nitrogen atoms. The substrates were chosen to be four different aldehydes that represent a wide range of electronic effects on their reacting carbonyl group (formaldehyde, acetaldehyde, acrolein, benzaldehyde). The base was chosen to be trimethylamine.

A key question regarding the reaction is the aggregation of the reactants. The formation of a hydrogen bond between the base and the cation to give **I** was found to be thermodynamically favorable in all cases, as shown by the Δ*H* and Δ*G* values in Table 1. The association of a substrate molecule to **I** to give **2** has an enthalpy benefit of 8–11 kcal mol^−1^, which can be explained by the polarity of complex **I** and the aldehyde (Table 1). This substantial Δ*H* value is comparable in magnitude to the *T*Δ*S* entropic contribution, and therefore the relative Gibbs free energies of **I** and **2** compared to the three dissociated components are very similar. Consequently, aggregation at least to **I** should occur in all investigated cases. The low relative Gibbs free energy of **2** suggest that this aggregate is easily accessible in the solution, and in some cases, it should be even the (slightly) dominant structure in the reaction mixture.

**Table 1:** DLPNO‐CCSD(T)/CBS//TPSSh‐D3BJ/def2‐TZVPP Gibbs free relative energies and relative enthalpies for **I** and **2** with respect to the separated azolium cation, trimethylamine, and substrate in the gas phase.

Catalyst	Aldehyde	Δ*G*(**I**)	Δ*G*(**2**)	Δ*H*(**I**)	Δ*H*(**2**)
	R−CHO	[kcal mol^−1^]	[kcal mol^−1^]	[kcal mol^−1^]	[kcal mol^−1^]
**A**	H	−1.3	−1.0	−11.7	−19.3
**A**	CH_3_	−1.3	−1.4	−11.7	−21.3
**A**	vinyl	−1.3	−1.2	−11.7	−21.3
**A**	Ph	−1.3	−0.9	−11.7	−24.3
**B**	H	−2.2	−3.1	−13.2	−22.6
**B**	CH_3_	−2.2	−3.6	−13.2	−22.9
**B**	vinyl	−2.2	−4.5	−13.2	−24.4
**B**	Ph	−2.2	−2.1	−13.2	−25.6
**C**	H	−2.9	−1.7	−13.1	−20.4
**C**	CH_3_	−2.9	−0.2	−13.1	−20.4
**C**	vinyl	−2.9	−3.9	−13.1	−23.5
**C**	Ph	−2.9	−1.0	−13.1	−23.0

The enthalpy and Gibbs free energy barriers (Δ*H* ^≠^_dissoc_, Δ*G* ^≠^_dissoc_) for the dissociative mechanism were measured in all cases as the enthalpy and Gibbs free energy difference between **I** and **TS_II_**_→**III**_. For the associative mechanism, the enthalpy barriers (Δ*H* ^≠^_assoc_) were defined to be the enthalpy difference between **TS_2_**_→**IV**_ and **2**. The reference point for calculating the Gibbs free energy barrier of the associated path (Δ*G* ^≠^_assoc_) was defined separately for each cases, taking the more stable of **2** or, **I**. The relative Gibbs free energy of **TS_2_**_→**IV**_ compared to this reference point gave Δ*G* ^≠^_assoc_.

The associative mechanism has significantly lower activation enthalpies in all cases than those of the dissociative path. As discussed before, the two sets of barriers exhibit a common trend, hence a higher activation enthalpy in the dissociative mechanism usually means a higher barrier for the associative path as well.^40^ The difference between the enthalpy barriers is substantial, ranging between 19 and 29 kcal mol^−1^ (Table 2).^40^ In fact, the activation enthalpies for the dissociative mechanism are so high, that these processes seem rather unlikely to occur at all at reasonable temperatures (37–49 kcal mol^−1^), whereas the associative mechanism exhibits mild 11–25 kcal mol^−1^ barriers. In the associative mechanism, the imidazolium catalyst showed generally higher barriers (20–25 kcal mol^−1^) than those of the triazolium and thiazolium derivatives (11–17 kcal mol^−1^), which is in qualitative agreement with the lower mobility of the active proton for the imidazolium derivatives.^44^, ^45^, ^46^, ^47^, ^48^, ^50^ Catalyzed by certain imidazolium derivatives, the condensation of formaldehyde in the presence of triethylamine was too slow to observe at room temperature in earlier experiments,^51^ which is in good accordance with our data. The activation enthalpies were found to be only slightly different for the four substrates, but in most cases the highest values were found for benzaldehyde, whereas the lowest were found for acrolein. Including entropy effects by calculating Gibbs free energies increases all barriers. The increase is generally higher for the associative mechanism (2–10 kcal mol^−1^) than for the dissociative path (0–5 kcal mol^−1^, Table 2), in agreement with the higher order in **TS_2_**_→**IV**_. Nonetheless, the entropic penalty for reaching **TS_2_**_→**IV**_ is relatively low, since the association occurred at the earlier steps of the reaction, namely at the formation of **2** (see discussion above).

**Table 2:** DLPNO‐CCSD(T)/CBS//TPSSh‐D3BJ/def2‐TZVPP activation enthalpies and activation Gibbs free energies for the reaction of 1,3‐dimethylimidazolium (**A**), 1,4‐dimethyltriazolium (**B**), 3‐methylthiazolium (**C**) cations, and trimethylamine with different aldehydes in the gas phase through the associative (Δ*H* ^≠^_assoc_, Δ*G* ^≠^_assoc_) and dissociative (Δ*H* ^≠^_dissoc_, Δ*G* ^≠^_dissoc_) reaction mechanisms.

Catalyst	Aldehyde	Δ*G* ^≠^_assoc_	Δ*G* ^≠^_dissoc_	Δ*H* ^≠^_assoc_	Δ*H* ^≠^_dissoc_
	R−CHO	[kcal mol^−1^]	[kcal mol^−1^]	[kcal mol^−1^]	[kcal mol^−1^]
**A**	H	26.1	46.6	22.1	46.0
**A**	CH_3_	23.2	50.2	21.5	48.5
**A**	vinyl	23.5	48.6	20.4	45.8
**A**	Ph	26.6	52.6	24.9	47.4
**B**	H	18.3	37.2	15.2	37.3
**B**	CH_3_	19.2	40.5	16.2	39.8
**B**	vinyl	16.8	38.7	14.7	37.1
**B**	Ph	20.5	41.8	16.3	39.4
**C**	H	17.3	39.4	11.8	38.3
**C**	CH_3_	19.2	42.6	11.3	40.6
**C**	vinyl	14.1	40.8	11.5	38.1
**C**	Ph	19.3	42.7	13.0	39.2

**Figure 5:**
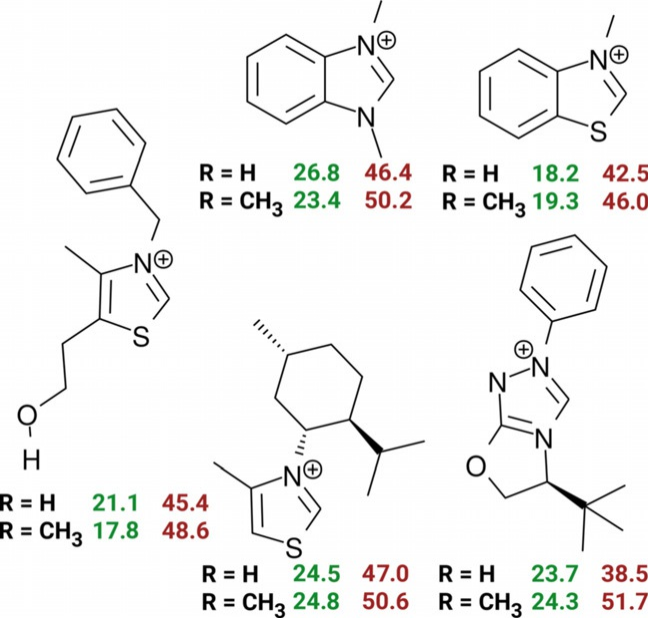
DLPNO‐CCSD(T)/CBS//TPSSh‐D3BJ/def2‐TZVPP Gibbs free energy barriers of the reaction between often applied catalysts, triethylamine and formaldehyde (above) or acetaldehyde (below) through the dissociative (red) and associative (green) reaction mechanisms.

Changing trimethylamine to other deprotonating agents, such as 1,8‐diazabicyclo[5.4.0]undec‐7‐ene (DBU) and 1,4‐diazabicyclo[2.2.2]octane (DABCO) changes both reaction barriers, but the dominance of the associative mechanism is retained (see the Supporting Information). Substituting the ring with larger groups to arrive at frequently applied catalysts does not affect the conclusions (Figure 5). Some of the compounds considered here possess bulky functional groups that control enantioselectivity in the later stages of the reaction.^6^ Nonetheless, the barriers of the reactions with formaldehyde and acetaldehyde indicate for all catalysts a distinct preference for the associative mechanism.

**Figure 6:**
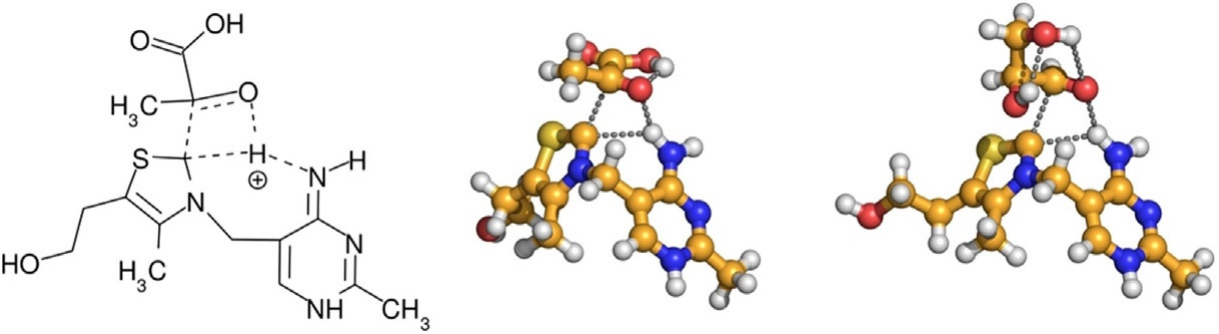
Lewis structure of the associative transition state of thiamine with pyruvic acid as a substrate (left). Ball‐and‐stick representation of the same transition state with pyruvic acid (middle) and glyceraldehyde (right) as substrate.

The most important catalyst is, of course, the biologically active thiamine (Figure 4). The calculations discussed above regarding the synthetically relevant azolium rings, bases, and aldehydes, can be and have been^15^ considered as model reactions for the biochemical reactions of thiamine. To test the feasibility of the associative mechanism on this biomolecule, we calculated the reactions of thiamine with pyruvic acid, and with glyceraldehyde, in which the bond between the catalyst and a substrate is formed. For these reactions it is not possible to define a dissociative mechanism, because the base and the azolium ring are covalently attached to each other within thiamine. We have, however, successfully located the transition state of the associative mechanism, bringing a novel insight into the corresponding biologically relevant reactions (Figure 6). The activation enthalpies were found to be reasonably low (24.4 and 15.1 kcal mol^−1^ for pyruvic acid and glyceraldehyde, respectively), which—in the presence of the enzyme—could be most likely decreased even further through stabilizing interactions within the transition state. Considering that the deprotonating agent and the catalyst are within the same molecule, entropy has a milder effect on this reaction, as shown by the activation Gibbs free energies (25.4 and 17.7 kcal mol^−1^, respectively).

Having seen the clear preference of the reaction to follow the associative path in the cases detailed above, the question is apparent: what effects make this mechanism so much faster than the dissociative path defined by NHC intermediate formation? One of the effects that make the associative mechanism favorable is the aforementioned high association enthalpy of the components to form **I** and **2**, which compensates for the entropy loss in the association of three molecules into a single cluster. Another reason must be that the associative path avoids the complete liberation of a highly reactive carbene lone pair, and instead this nucleophilic and basic center is simply flipped from the proton to the electrophilic carbonyl carbon atom of the aldehyde. Non‐covalent interaction analysis^82^, ^83^, ^84^ (see the Supporting Information) reveals that the interaction between the carbonyl carbon atom and the carbene carbon atom is in general considerable, strongly supporting this hypothesis.

Next to these clear advantages, there is another interaction in **TS_2_**_→**IV**_, which deserves special attention. It has been shown that the C‐to‐N and N‐to‐O proton transfers and the C−C bond formation occur asynchronously within the single elementary step of the reaction.^40^, ^64^ The transition state is situated late along the reaction coordinate,^64^ where the C‐to‐N proton transfer has already been completed. The mobilized proton, while it is departing the azolium ring and approaching the nitrogen atom of the amine base shuttle, will form a transient, but strong hydrogen bond donor species within this assembly, an ammonium cation. While the catalyst−substrate bond is being formed, the transient ammonium cation interacts strongly with the aldehyde oxygen atom through a hydrogen bond, which is observable in all transition states **TS_2_**_→**IV**_ through various hydrogen bonding criteria (Table 3, and the Supporting Information).

**Table 3:** Hydrogen bonding between the ammonium and aldehyde moieties in the key transition state of the associative reaction mechanism **TS_2_**_→**IV**_ (*R*: O⋅⋅⋅H distance, *α*: O⋅⋅⋅H−N angle, BI: Mayer bond index, SEN: shared electron number, BCP *ρ*(*r*) and ∇^2^ *ρ*(*r*): electron density and Laplacian, at the bond critical point of the O⋅⋅⋅H hydrogen bond respectively, ΔΔ*E* ^≠^: DLPNO‐CCSD(T)/CBS//TPSSh‐D3BJ/def2‐TZVPP activation energy change in the carbene+substrate reaction if the protonated base is present).

Catalyst	Aldehyde	*R*	*α*	BI	SEN	BCP		ΔΔ*E* ^≠^
	R−CHO	[Å]	[°]			*ρ*(*r*)	∇^2^ *ρ*(*r*)	[kcal mol^−1^]
**A**	H	1.87	140.0	0.056	0.078	0.030	0.110	−15.9
**A**	CH_3_	1.67	170.1	0.171	0.127	0.050	0.131	−17.8
**A**	Ph	1.47	174.3	0.292	0.217	0.088	0.121	−14.3
**A**	vinyl	1.72	154.8	0.095	0.108	0.042	0.130	−16.4
**B**	H	1.72	171.4	0.156	0.107	0.046	0.120	−15.5
**B**	CH_3_	1.56	173.8	0.233	0.172	0.069	0.127	−15.5
**B**	Ph	1.47	174.3	0.289	0.214	0.088	0.122	−12.0
**B**	vinyl	1.70	165.1	0.141	0.104	0.045	0.128	−15.2
**C**	H	1.71	167.8	0.154	0.105	0.046	0.120	−17.8
**C**	CH_3_	1.55	173.5	0.244	0.136	0.072	0.126	−16.5
**C**	Ph	1.46	175.5	0.296	0.216	0.090	0.118	−13.1
**C**	vinyl	1.69	169.5	0.161	0.110	0.050	0.122	−18.6
**T**	glyc^[a]^	2.03	140.5	0.058	0.021	0.021	0.083	–
**T**	pyru^[a]^	1.98	150.7	0.093	0.037	0.023	0.086	–

[a] Glyc: glyceraldehyde; pyru: pyruvic acid.

Carbonyl compounds are known to be activated in their electrophilicity by hydrogen‐bond donor molecules, which polarize the C=O bond, and thereby decrease its strength.^92^, ^93^, ^94^, ^95^ Enzymatic reactions often take effect through such hydrogen bonding, which activate molecules in a manner to enhance their desired reactivity,^94^, ^96^ whereas the active site also offers a well‐defined spatial arrangement of hydrogen‐bonding sites, inducing thereby selectivity through template effects. Mimicking these biochemical reactions, hydrogen‐bond‐supported catalysis has been subject to intensive scientific attention through the last few decades, using for instance urea, thiourea, and guanidinium derivatives as catalysts.^92^, ^93^, ^94^, ^95^ Accordingly, it is reasonable to assume that the presence of the aforementioned ammonium‐substrate interplay in **TS_2_**_→**IV**_ reaches beyond a simple hydrogen bond, and it affects the reactivity of the aldehyde directly. Considering that the transition state is situated late within the concerted asynchronous elementary step that comprises the proton transfers and the C−C bond formation,^64^ where the proton is already transferred to the amine, it can be expected that the barrier itself is largely determined by the C−C bond formation.^64^ Therefore, the activation effect of the amine on the aldehyde may have a tremendous effect on the barrier of the reaction.

To highlight the effect of this hydrogen bond on the reaction, the changes in the structure of the four selected aldehydes induced by presence of ammonium cations are shown in Table 4. The activation should manifest in weaker C=O double bonds, which is a clear sign of the polarization by moving the electron density of the π bond toward the oxygen atom, rendering the molecule more electrophilic. All data indicates that the C=O bond of the substrate becomes weaker upon interacting with an ammonium cation, implying—in agreement with literature^92^, ^93^, ^94^, ^95^—that this interaction has an effect on the electrophilicity of the substrate, and thus the reaction as well.

**Table 4:** The activation of aldehydes in electrophilicity upon hydrogen bonding to ammonium salts (*R*_O⋅⋅⋅H_: O⋅⋅⋅H distance, *R*_C=O_: C=O distance in the aldehyde, BI: Mayer bond index, SEN: shared electron number, obtained at the TPSSh‐D3BJ/def2‐TZVPP level).

Aldehyde	Donor	*R*_O⋅⋅⋅H_	*R*_C=O_	BI	SEN
R−CHO	–	[Å]	[Å]	(C=O)	(C=O)
H	–	–	1.20	2.12	2.02
CH_3_	–	–	1.21	2.13	1.97
Ph	–	–	1.21	2.08	1.92
vinyl	–	–	1.21	2.06	1.92
H	Me_3_NH^+^	1.76	1.21	1.96	1.98
CH_3_	Me_3_NH^+^	1.69	1.22	1.90	1.89
Ph	Me_3_NH^+^	1.63	1.23	1.78	1.80
vinyl	Me_3_NH^+^	1.66	1.23	1.79	1.83
H	DBUH^+^	1.87	1.21	2.00	1.98
CH_3_	DBUH^+^	1.81	1.22	1.94	1.91
Ph	DBUH^+^	1.75	1.23	1.82	1.85
vinyl	DBUH^+^	1.78	1.23	1.83	1.83

To assess how much this interaction facilitates the reaction, we compared the barriers for the reaction between a free carbene and a free aldehyde in the absence and presence of a trimethylammonium cation in hydrogen bonding with the substrate (ΔΔ*E* ^≠^ in Table 3). The obtained data shows an approximately 12–19 kcal mol^−1^ decrease in the activation energies by the presence of the ammonium ion. This significant decrease in the barriers suggests that “umpolung” catalysis by NHCs, for instance benzoin condensation, also works partly as an inherent hydrogen bond‐supported catalysis.

**Figure 7:**
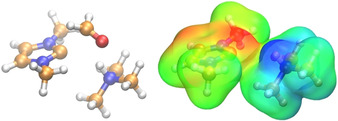
Ball‐and‐stick representation of the transition state for the associative reaction mechanism for the reaction between 1,3‐dimethylimidazolium cation, trimethylamine, and formaldehyde (left, C: orange; O: red; N: blue; H: white). Electrostatic potential map of the same structure, after the formaldehyde was removed (right). The negatively polarized (red) lone pair, and the positively polarized (blue) ammonium cation together define a binding site for the aldehyde, which can react with the catalyst, and can be activated by the protonated base to lower the barrier.

The nature of the interplay within the transition state can be perhaps best represented in the electrostatic potential maps of the carbene and the ammonium cation within the transition state **TS_2_**_→**IV**_ (Figure 7). The positively charged proton on the ammonium cation units and the negatively polarized carbene lone pair are situated in such a manner that they can together encompass the substrate in the possible most favorable arrangement, through creating a sort of template. Whereas the lone pair of the carbene interacts with the LUMO of the aldehyde at the partially positive carbonyl carbon atom, the ammonium cation can aid the nucleophilic attack through forming a hydrogen bond with the negatively polarized oxygen atom of the substrate. This cooperative effect clearly shows that through modifying the template formed by the azolium–base assembly, the feasibility of the reaction may be influenced, creating further ways to improve the selectivity of these reactions.

The effects that result in the lower barriers for the associative mechanism may also reveal the limitation of this path. Considering that hydrogen bonding with the aldehyde is of high importance, other species in the solution that can affect the solvation and hydrogen bonding situation of this moiety—most importantly the solvent and the counterion of the azolium cation—might flip the balance between the two mechanisms toward the dissociative path. Solvents with hydrogen bonding ability can take over the role of the ammonium cation in activating the substrate for the reaction. Furthermore, because intermediate **III** and transition state **TS_II_**_→**III**_ are zwitterionic structures, the mere polarity of the solvent may alter their stabilities, and thereby the corresponding barrier. Through a proton transfer from the azolium cation to the amine, solvation energies might change significantly, because such protic ammonium ions are generally stronger hydrogen bond donors than their azolium counterparts,^97^ shifting the relative free energies. Furthermore, the free NHC can be stabilized by hydrogen bonding, which also facilitates the deprotonation of the azolium cation, and hence stabilizes the dissociative path.

**Figure 8:**
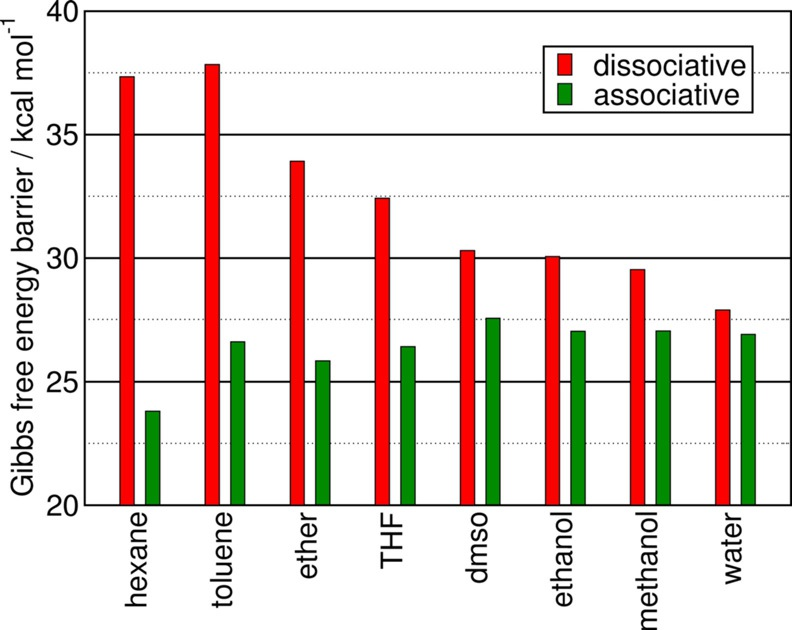
Solvent effects on the Gibbs free energy barriers of the reaction between 1,3‐dimethylimidazolium cation, trimethylamine, and acetaldehyde by using the COSMO‐RS model and the DLPNO‐CCSD(T)/CBS//TPSSh‐D3BJ/def2‐TZVPP method.^67^‐^74^, ^76^, ^77^, ^80^ For numerical values, see the Supporting Information.

Thus, we investigated solvent effects on the barriers in an array of organic solvents through the COSMO‐RS approach.^76^, ^77^, ^79^ This solvent model can accurately account for the effects of the polarity of the solvent, and also hydrogen bonding interactions, making it ideal for the present purposes. In agreement with the reasoning above, the polarity of the solvent makes a big difference (Figure 8). Moving from the less polar solvents (toluene, hexane, THF) toward the highly polar ones (DMSO), the barrier of the associative path slightly increases, whereas that of the dissociative mechanism remarkably decreases. This effect is even stronger for hydrogen bond donor solvents (EtOH, MeOH, water). In aqueous solution the advantage of the associative mechanism is reduced to approximately 1 kcal mol^−1^.

Thus, the polar environment and hydrogen bonding apparently decreases the barrier of the dissociative path through stabilizing **TS_II_**_→**III**_. However, hydrogen bonding can also have a tremendous effect on the availability of the free NHC. NHCs are highly basic compounds, and hence they can form very strong hydrogen bonds, which can result in a stabilization up to even 10–20 kcal mol^−1^.^30^, ^98^, ^99^, ^100^ Although the stabilization by hydrogen bonding activates the aldehyde substrate, it may also occupy the NHC lone pair, which is the very site, to which the electrophilic substrate should bind in the reaction. Hydrogen bonding at this position has been, therefore, repeatedly invoked as a factor that diminishes the catalytic activity of NHCs.^102^, ^103^ For this reason, at first glance the hydrogen bond between the solvent molecule and the NHC may seem rather counterproductive. Thus, next to the dissociative and associative mechanisms that were distinguished above, in which the carbene participates as a free or as a protonated species (i.e., azolium cation), hydrogen bonded carbenes should represent another degree of freedom for the carbene's lone pair, somewhere in between these two extremes.

**Figure 9:**
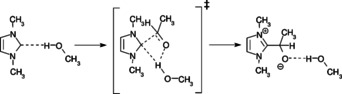
Associative reaction of an NHC directly from its hydrogen‐bonded form without the formation of a free carbene species.

Similarly to the associative mechanism detailed above, the hydrogen bond donor solvent molecule can be replaced by the substrate without forming an unstable, free NHC in the solution, through the transition state shown in Figure 9. The obtained Gibbs free energies of activation (for the reaction of 1,3‐dimethylimidazol‐2‐ylidene with formaldehyde: Δ*G* ^≠^_solv_=7.4 kcal mol^−1^; with benzaldehyde: Δ*G* ^≠^_solv_=15.7 kcal mol^−1^; both in methanol) are quite low. Comparing this mechanism to the one that proceeds through the free carbene is not possible due to technical reasons: The COSMO‐RS solvent model in all cases considers the best possible solute–solvent interactions, thus, it automatically forms a hydrogen bond between the NHC and the implicit solvent whenever the lone pair is available, hindering the calculation of a fully free carbene molecule (i.e. with an available lone pair, not blocked by protonation or hydrogen bonding). However, the values shown above for the mechanism in Figure 9 are very similar to the dissociation Gibbs free energy of a carbene‐alcohol bond, thus, that of producing a free NHC. A hypothetical free NHC, with no deactivating hydrogen bonds from the solvent, would need to react through yet another barrier to form the C−C bond with the substrate. Accordingly, it is reasonable to assume that the mechanism in Figure 9 is more feasible. Thus, in protic (hydrogen bonding) solvents the dissociative mechanism can be rationalized as a substitution of the ammonium cation by the solvent at the hydrogen bond acceptor hypovalent carbon atom, followed by the reaction depicted in Figure 9. Such exchange of hydrogen bond donors at NHCs has been discussed before, and has been found to depend on steric effects and the hydrogen bond acceptor strength of the NHC in its rate and mechanism.^99^, ^100^ The reaction of the solvent–NHC hydrogen‐bonded complex with the substrate (Figure 9) can be considered as a special case of the associative mechanism depicted in Figure 3.

The presence of the anion in the solution next to the reacting species might also have significant effects on the differences in the barriers of the associative and dissociative reactions. The first question in this regard is if the azolium cations and the anions remain associated in solution in the form of an ion pair, and can thereby affect the reaction, or if they instead dissociate into individual ions in the solution, and the reaction mechanism remains mostly unaffected by the anion. Ion pairing can be influenced by the solvent, and by the nature of the ions themselves.

To uncover in what cases ion pairing might occur in these reaction mixtures, we performed umbrella sampling calculations in a classical molecular dynamics environment on a 1,3‐dimethylimidazolium bromide ion pair, and a 1,3‐dimethylimidazolium triflate ion pair in different solvents (see Computational Methods, and the Supporting Information). Whereas the chloride anion is considered highly coordinative, the triflate anion is considered non‐coordinative in their imidazolium salts, for instance in ionic liquids.^104^ In these calculations, the free energy was obtained as a function of the distance between the anion and cation, providing an estimate for the propensity of the given anion–cation pair to stay associated. The separation of the ions is in almost all cases endothermic, only in the most dissociating aqueous solution is the energy of dissociation close to zero. Based on these findings, imidazolium salts are apparently dissolved in all the investigated solvents as ion pairs. In the lack of such well‐tested force field parameters for thiazolium and triazolium rings it is not possible to have such a quantitative insight for the corresponding salts. However, considering that those two cations are more acidic than imidazolium cations, it is reasonable to assume that they are stronger hydrogen bond donors, and therefore they would be even more strongly coordinated to the anions, and consequently the formation of ion pairs can be expected in these solutions as well.

Thus, the presence of the anions must be considered for the reactions, if the full picture is to be obtained regarding the two competing mechanisms. To this end, we chose a series of anions (halides, tetrafluoroborate, and triflate) that are often applied in synthesis as counterions for the catalysts, and examined the Gibbs free energy barriers of the associative and dissociative paths in a series of solvents. Based on the obtained data, the difference between the barriers became lower, amounting to less than 11 kcal mol^−1^ in all cases (Tables 5 and 6). In fact, the preference varies between the two mechanisms, depending on the anion and on the solvent. In general, as observed above, the increasing polarity and hydrogen bonding ability of the solvent facilitates the dissociative mechanism, and hinders the associative one. The increasing size of the halide anions shifts the preference toward the dissociative mechanism, although for the reactions with the thiazolium cation the trend between bromide and iodide is reverse. The two other anions, tetrafluoroborate and triflate, apparently make the associative mechanism more dominant than bromide or iodide, exhibiting similar differences in the two barriers to those for chloride. Imidazolium‐based salts appear to be the least favorable for the associative path to occur, whereas the very often applied triazolium catalyst shows the highest propensity to support this mechanism.

**Table 5:** DLPNO‐CCSD(T)/CBS//TPSSh‐D3BJ/def2‐TZVPP difference between the Gibbs free energy barriers of the catalyst+formaldehyde+triethylamine reaction for the associative and dissociative mechanisms (ΔΔ*G* ^≠^=Δ*G* ^≠^_assoc_−Δ*G* ^≠^_dissoc_, in kcal mol^−1^) in non‐polar media, in the presence of various anions. Negative values mean that the associative mechanism is faster in that particular case.

Catalyst	Anion	Gas phase	Hexane	Toluene	Et_2_O	THF
**A**	Cl^−^	−5.2	−1.8	−0.9	−0.7	−0.3
**A**	Br^−^	−1.2	0.9	2.0	2.0	2.4
**A**	I^−^	0.5	2.0	3.0	2.9	3.3
**A**	[BF_4_]^−^	−3.7	−1.7	−0.5	−0.5	−0.2
**A**	[CF_3_SO_3_]^−^	−4.7	−0.6	0.4	0.3	0.7
**B**	Cl^−^	−6.9	−4.7	−3.6	−3.4	−3.2
**B**	Br^−^	6.1	7.4	8.5	8.7	8.8
**B**	I^−^	8.0	8.9	9.7	9.9	10.0
**B**	[BF_4_]^−^	−6.4	−4.3	−3.1	−3.0	−2.8
**B**	[CF_3_SO_3_]^−^	−5.6	−3.8	−2.8	−2.7	−2.5
**C**	Cl^−^	−3.1	−2.7	−1.8	−1.6	−1.4
**C**	Br^−^	4.3	7.0	7.8	7.8	8.1
**C**	I^−^	2.7	5.3	6.0	5.9	6.1
**C**	[BF_4_]^−^	−4.0	−2.6	−1.6	−1.6	−1.4
**C**	[CF_3_SO_3_]^−^	−5.9	−2.0	−1.0	−1.0	−0.6

**Table 6:** DLPNO‐CCSD(T)/CBS//TPSSh‐D3BJ/def2‐TZVPP difference between the Gibbs free energy barriers of the catalyst+formaldehyde+triethylamine reaction for the associative and dissociative mechanisms (ΔΔ*G* ^≠^=Δ*G* ^≠^_assoc_−Δ*G* ^≠^_dissoc_, in kcal mol^−1^) in polar solvents, in the presence of various anions. Negative values mean that the associative mechanism is faster in that particular case.

Catalyst	Anion	DMSO	Ethanol	Methanol	Water
**A**	Cl^−^	0.5	1.7	1.8	0.3
**A**	Br^−^	3.5	4.1	4.3	4.2
**A**	I^−^	4.2	4.6	4.8	4.5
**A**	[BF_4_]^−^	0.9	2.1	2.3	1.9
**A**	[CF_3_SO_3_]^−^	1.6	2.7	2.7	1.6
**B**	Cl^−^	−2.2	−0.8	−0.5	−0.6
**B**	Br^−^	9.5	10.0	10.0	9.2
**B**	I^−^	10.5	10.8	10.7	9.7
**B**	[BF_4_]^−^	−1.8	−0.5	−0.3	−0.5
**B**	[CF_3_SO_3_]^−^	−1.6	−0.2	0.0	0.3
**C**	Cl^−^	−0.6	0.8	1.0	0.1
**C**	Br^−^	8.8	9.1	9.1	8.0
**C**	I^−^	6.7	7.2	7.1	5.8
**C**	[BF_4_]^−^	−0.6	0.8	0.9	−0.1
**C**	[CF_3_SO_3_]^−^	0.3	1.3	1.3	0.3

**Figure 10:**
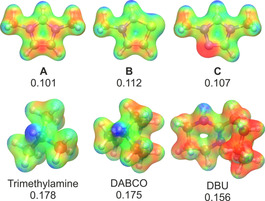
Electrostatic potential maps of catalysts **A**, **B**, and **C** (top), as well as the protonated trimethylamine, DABCO, and DBU (bottom). The more positive areas (blue) are less localized for the azolium cations (top) than for the protonated amine bases (bottom), which makes the latter group of compounds stronger hydrogen‐bond donors. The Hirshfeld charge (TPSSh‐D3BJ/def2TZVPP) at the mobile hydrogen atom is shown for each compound, supporting this hypothesis.

Under closer scrutiny, the role of the anion in decreasing the difference between the barriers can be identified. The positive charge on the azolium cations is delocalized over the whole ring, whereas on the ammonium cation it is highly localized on the N−H unit. Therefore, although azolium cations have also been shown to be hydrogen‐bond donors,^63^, ^104^, ^105^ ammonium cations offer an even stronger donor site. These differences are clearly observable in the electrostatic potential maps of the azolium and ammonium ions, as well as the charges of the hydrogen bond donor sites (Figure 10). Thus, the strong hydrogen bond, formed between the ammonium ion and the anion, can compensate for the energy demand of moving the proton from the stronger base carbene to the weaker amine. This compensation effect can be estimated through the metathesis reaction [azolium^+^X^−^]+HNMe^+^→azolium^+^+[HNMe^+^X^−^], showing the Gibbs free energy benefit of exchanging the azolium cation to an ammonium in interaction with anion X (Table 7). All anions interact apparently stronger with the ammonium cation than with any of the azolium species. Generally, the imidazolium cation **A** shows the lowest reaction Gibbs free energies, followed by the thiazolium cation **C**, whereas the least exergonic cation exchange was observed for the triazolium cation **B**. This trend is in full accordance with the discussion above, showing that the preference for the dissociative mechanism decreases in exactly this order. The Gibbs free energy is most negative in case of the halide anions, and moving toward the larger species with a delocalized charge the metathesis becomes less exergonic. Interestingly, the halides show an opposite trend, as the chloride—despite the significantly stronger interactions with the ammonium cation than with the azolium cation—decreases the least the propensity of the catalysts to undergo the associative mechanism (Tables 5 and 6). The explanation for this discrepancy might lie in the small size of the chloride anion, which allows some inter‐ action through hydrogen bonding between the anion and the ammonium moiety also in transition state **TS_2_**_→**IV**_ of the associative mechanism. Through this interplay, the system receives some stabilization also in this mechanism, albeit significantly less than in **TS_III_**_→**IV**_ of the dissociative path, where the HNMe^+^Cl^−^ ion pair is separated from the reacting catalyst–substrate pair.

**Table 7:** DLPNO‐CCSD(T)/CBS//TPSSh‐D3BJ/def2‐TZVPP Gibbs free energies (in kcal mol^−1^) of the metathesis reaction [azolium^+^X^−^]+HNMe^+^_3_→azolium^+^+[HNMe^+^_3_X^−^], showing the energy benefit of exchanging the azolium cation to an trimethylammonium cation in interaction with anion **X**.

Catalyst	Cl^−^	Br^−^	I^−^	[BF_4_]^−^	[CF_3_SO_3_]^−^
**A**	−23.7	−21.7	−18.2	−13.1	−16.5
**B**	−16.5	−14.3	−12.2	−7.2	−10.5
**C**	−19.1	−17.0	−15.2	−11.0	−14.0

The effects above result in the conclusion that controlling hydrogen bonding and polarity effects of the solvent and the anion lead to a control over the reaction mechanism of N‐heterocyclic carbene organocatalysis. The less polar solvents, and smaller halides or weakly hydrogen bonding anions lead to a preference in the associative mechanism, whereas the use of polar solvents and larger halides facilitates the dissociative mechanism. The numbers in Tables 5 and 6 show that switching between the mechanisms is possible. It is, however, very important to emphasize here the difficulties regarding the estimation of entropies in quantum chemical calculations, and it has been repeatedly discussed that entropy effects are significantly overestimated,^106^, ^107^, ^108^ also for related reactions,^64^ which results in an overestimation of the barriers for the associative reaction mechanism. Thus, although the trends for the shift in preference between the two mechanisms should be valid, it is possible that the actual numbers in Tables 5 and 6 should be somewhat more negative, and the associative mechanism slightly more preferred. The data presented here show that the associative mechanism, which avoids the formation of actual carbenes in the solution, has mild barriers, and should be considered in all cases when investigating the mechanism of NHC organocatalysis, especially in case of higher concentrations of the substrate, base and catalyst in the solution.

## Summary and Conclusion

In the present computational study, two mechanisms were compared for the formation of the catalyst−substrate bond in NHC organocatalysis. These two mechanisms fundamentally differ in terms of the occurrence of free NHCs in the reaction mixture. In the widely accepted (dissociative) mechanism of this process, as described by Breslow,^15^ the formation of free NHC intermediates via the deprotonation of the azolium salt catalyst is required, and the resulting low concentration of carbenes in the solution offers the actual catalytic activity. In an alternative, associative mechanism,^40^ the deprotonation of the very weakly acidic azolium salts is bypassed in a concerted process, in which the proton transfer and the catalyst−substrate bond formation occurs in a single elementary step. Depending on the mechanism, the selectivity of the reaction might be influenced, especially because in the transition state of the associative path the base is also present, and therefore its bulkiness and possible template effects can influence the outcome of the reaction as well. In a series of model reactions, imidazolium, triazolium, and thiazolium cations were considered as catalysts with varying substituents on the relevant nitrogen atoms, formaldehyde, acetaldehyde, benzaldehyde, and acrolein as substrates, and trimethylamine, DBU, and DABCO as bases.

In the absence of solvents and anions the reaction follows the associative mechanism, avoiding, thereby, the formation of free carbenes in the solution. The reasons for this dominance were identified to be the high enthalpic benefit of the association of the components, and the interaction of the mobile proton on one hand with the azolium ring, avoiding the complete liberation of the reactive carbene lone pair, whereas on the other hand with the oxygen atom of the substrate, which activates this molecule for the nucleophilic attack of the catalyst. This complex network of stabilizing hydrogen‐bonding interactions can be, however, disrupted and partly substituted in the presence of polar and protic solvents, which offer alternative, modes of stabilization in case of the dissociative mechanism.

Imidazolium salts in a variety of organic solvents and water were found to stay associated within the same solvent shell in the form of ion pairs. The presence of anions vanishes most of the dominance of the associative mechanism, and the barriers become more similar. The underlying reason for this effect was identified to be the stronger interaction of the anion with the protonated base than with the azolium cation, which shifts the acid–base equilibrium toward the free carbene, and partially breaks up the azolium–base‐substrate aggregates. In case of stronger hydrogen‐bond acceptor anions (e.g., halides), the dissociative process exhibits lower barriers, whereas in case of weak hydrogen‐bond acceptor anions (e.g., tetrafluoroborate and triflate), the associative path was found to be faster. An exception here was the chloride anion, which—due to its small size—can form stabilizing interactions with the hydrogen‐bond donor species also within the transition state of the associative path, retaining thereby the preference for this mechanism in many cases. Accordingly, the mechanism can be controlled through varying the anion and the solvent, and therefore introducing novel kinds of selectivities into these mechanism should also focus on carefully choosing these elements of the catalytic system.

The results above bring also new insight into the related biochemical reactions of vitamin B1, thiamine. Considering that in this reaction the thiazolium ring and the base that should deprotonate it are covalently bound, only the associative reaction mechanism is feasible. In the lack of significant entropic effects, the barriers of two model reactions of thiamine were found to be low, suggesting the viability of the associative mechanism in biological systems.

## Conflict of interest

The authors declare no conflict of interest.

## Biographical Information

*Dr. Hollóczki obtained his PhD in Chemistry at the Budapest University of Technology and Economics. In 2012, he received the Humboldt Fellowship for Postdoctoral Researchers, which financed his stay at the Leipzig University and at the University of Bonn. Currently, he is doing his habilitation as a junior group leader at the University of Bonn. In 2018, he was awarded by the ADUC Prize for his research accomplishments. The research interests of Dr. Hollóczki span from catalysis through sustainable chemistry to the environmental effects of plastic wastes*.

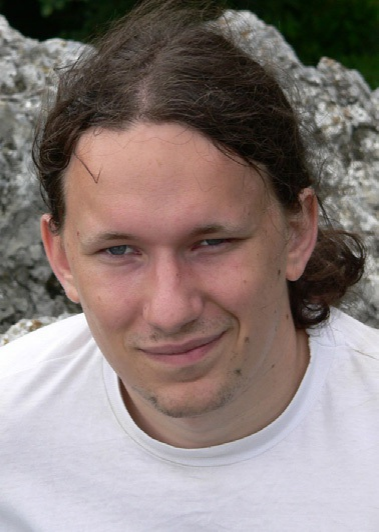

## Supporting information

As a service to our authors and readers, this journal provides supporting information supplied by the authors. Such materials are peer reviewed and may be re‐organized for online delivery, but are not copy‐edited or typeset. Technical support issues arising from supporting information (other than missing files) should be addressed to the authors.

Supplementary

## References

[1] C. Eijkman , Medicine 1897, 148, 523–532.

[2] U. Suzuki , T. Shimamura , Tokyo Kagaku Kaishi 1911, 32, 4–7.

[3] H. Stetter , R. Y. Raemsch , H. Kuhlmann , Synthesis 1976, 733–735.

[4] N. Marion , S. Díez-González , S. P. Nolan , Angew. Chem. Int. Ed. 2007, 46, 2988–3000; 10.1002/anie.200603380 17348057

[5] D. Enders , O. Niemeier , A. Henseler , Chem. Rev. 2007, 107, 5606–5655. 17956132 10.1021/cr068372z

[6] D. Enders , T. Balensiefer , Acc. Chem. Res. 2004, 37, 534–541. 15311952 10.1021/ar030050j

[7] C. S. Cazin , N-Heterocyclic carbenes in transition metal catalysis and organocatalysis, Vol. 32, Springer, Heidelberg, 2010.

[8] S. Díez-González , N-Heterocyclic carbenes: from laboratory curiosities to efficient synthetic tools, RSC, London, 2011.

[9] D. Green , W. W. Westerfeld , B. Vennesland , W. E. Knox , J. Biol. Chem. 1942, 145, 69–84.

[10] T. Ukai , R. Tanaka , T. Dokawa , J. Pharm. Soc. Jpn 1943, 63, 296–300.

[11] B. L. Horecker , P. Z. Smyrniotis , J. Am. Chem. Soc. 1953, 75, 1009–1010.

[12] E. Racker , G. de la Haba , I. G. Leder , J. Am. Chem. Soc. 1953, 75, 1010–1011.

[13] R. Breslow , J. Am. Chem. Soc. 1957, 79, 1762–1763.

[14] A. Lapworth , J. Chem. Soc. Trans. 1904, 85, 1206–1214.

[15] R. Breslow , J. Am. Chem. Soc. 1958, 80, 3719–3726.

[16] T. Dudding , K. N. Houk , Proc. Natl. Acad. Sci. USA 2004, 101, 5770–5775. 15079058 10.1073/pnas.0307256101 PMC395983

[17] O. Hollóczki , Z. Kelemen , L. Nyulaszi , J. Org. Chem. 2012, 77, 6014–6022. 22731396 10.1021/jo300745e

[18] A. Berkessel , V. R. Yatham , S. Elfert , J.-M. Neudörfl , Angew. Chem. Int. Ed. 2013, 52, 11158–11162; 10.1002/anie.201303107 24038872

[19] A. Berkessel , S. Elfert , V. R. Yatham , J.-M. Neudörfl , N. E. Schlörer , J. H. Teles , Angew. Chem. Int. Ed. 2012, 51, 12370–1237; 10.1002/anie.201205878 23081675

[20] M. Paul , P. Sudkaow , A. Wessels , N. E. Schlörer , J.-M. Neudörfl , A. Berkessel , Angew. Chem. Int. Ed. 2018, 57, 8310–8315; 10.1002/anie.201801676 29645334

[21] B. Maji , H. Mayr , Angew. Chem. Int. Ed. 2012, 51, 10408–10412; 10.1002/anie.201204524 22968992

[22] D. A. DiRocco , K. M. Oberg , T. Rovis , J. Am. Chem. Soc. 2012, 134, 6143–6145. 22455368 10.1021/ja302031v PMC3336740

[23] A. J. Arduengo III , R. L. Harlow , M. Kline , J. Am. Chem. Soc. 1991, 113, 361–363.

[24] A. J. Arduengo III , J. R. Goerlich , W. J. Marshall , J. Am. Chem. Soc. 1995, 117, 11027–11028.

[25] D. Enders , K. Breuer , G. Raabe , J. Runsink , J. H. Teles , J.-P. Melder , K. Ebel , S. Brode , Angew. Chem. Int. Ed. Engl. 1995, 34, 1021–1023;

[26] A. J. Arduengo , J. R. Goerlich , W. Marshall , J. Liebigs Annalen 1997, 1997, 365–374.

[27] A. J. Arduengo , F. Davidson , H. R. Dias , J. R. Goerlich , D. Khasnis , W. J. Marshall , T. Prakasha , J. Am. Chem. Soc. 1997, 119, 12742–12749.

[28] H. Zeng , K. Wang , Y. Tian , Y. Niu , L. Greene , Z. Hu , J. K. Lee , Int. J. Mass Spectrom. 2014, 369, 92–97.

[29] Z. Kelemen , O. Hollóczki , J. Nagy , L. Nyulászi , Org. Biomol. Chem. 2011, 9, 5362–5364. 21701727 10.1039/c1ob05639e

[30] O. Hollóczki , D. Gerhard , K. Massone , L. Szarvas , B. Németh , T. Veszprémi , L. Nyulászi , New J. Chem. 2010, 34, 3004–3009.

[31] D. Meyer , P. Neumann , R. Ficner , K. Tittmann , Nat. Chem. Biol. 2013, 9, 488. 23748673 10.1038/nchembio.1275

[32] O. Hollóczki , Chem. Eur. J. 2020, 26, 4885–4894. 31797448 10.1002/chem.201903021 PMC7187225

[33] M. W. Washabaugh , W. P. Jencks , Biochemistry 1988, 27, 5044–5053. 2844248 10.1021/bi00414a015

[34] J. Castells , F. Lopez-Calahorra , L. Domingo , J. Org. Chem. 1988, 53, 4433–4436.

[35] J. Castells , L. Domingo , F. López-Calahorra , J. Martí , Tetrahedron Lett. 1993, 34, 517–520.

[36] J. Castells , F. López-Calahorra , F. Geijo , R. Pérez-Dolz , M. Bassedas , J. Heterocycl. Chem. 1986, 23, 715–720.

[37] F. Lopez-Calahorra , J. Castells , L. Domingo , J. Marti , M. Bofill , Heterocycles 1994, 37, 1579–1597.

[38] F. López-Calahorra , R. Rubires , Tetrahedron 1995, 51, 9713–9728.

[39] J. Martí , F. López-Calahorra , J. M. Bofill , J. Mol. Struct. 1995, 339, 179–194.

[40] S. Gehrke , O. Hollóczki , Angew. Chem. Int. Ed. 2017, 56, 16395–16398; 10.1002/anie.201708305 29072807

[41] J. Crosby , G. E. Lienhard , J. Am. Chem. Soc. 1970, 92, 5707–5716. 5458742 10.1021/ja00722a027

[42] J. Crosby , R. Stone , G. E. Lienhard , J. Am. Chem. Soc. 1970, 92, 2891–2900. 5439974 10.1021/ja00712a048

[43] D. S. Kemp , J. Am. Chem. Soc. 1970, 92, 2553–2554. 10.1021/ja00711a062 5438024

[44] E. M. Higgins , J. A. Sherwood , A. G. Lindsay , J. Armstrong , R. S. Massey , R. W. Alder , A. C. O'Donoghue , Chem. Commun. 2011, 47, 1559–1561. 10.1039/c0cc03367g 21116519

[45] Y. Chu , H. Deng , J.-P. Cheng , J. Org. Chem. 2007, 72, 7790–7793. 17725367 10.1021/jo070973i

[46] Y.-J. Kim , A. Streitwieser , J. Am. Chem. Soc. 2002, 124, 5757–5761. 12010050 10.1021/ja025628j

[47] R. W. Alder , P. R. Allen , S. J. Williams , J. Chem. Soc. Chem. Commun. 1995, 1267–1268.

[48] T. L. Amyes , S. T. Diver , J. P. Richard , F. M. Rivas , K. Toth , J. Am. Chem. Soc. 2004, 126, 4366–4374. 15053626 10.1021/ja039890j

[49] N. Konstandaras , M. H. Dunn , E. T. Luis , M. L. Cole , J. B. Harper , Org. Biomol. Chem. 2020, 18, 1910–1917. 32095802 10.1039/d0ob00036a

[50] R. S. Massey , C. J. Collett , A. G. Lindsay , A. D. Smith , A. C. O‘Donoghue , J. Am. Chem. Soc. 2012, 134, 20421–20432. 23173841 10.1021/ja308420c

[51] J. H. Teles , J.-P. Melder , K. Ebel , R. Schneider , E. Gehrer , W. Harder , S. Brode , D. Enders , K. Breuer , G. Raabe , Helv. Chim. Acta 1996, 79, 61–83.

[52] H. Hall Jr , J. Am. Chem. Soc. 1957, 79, 5441–5444.

[53] S. Tshepelevitsh , A. Kütt , M. Lõkov , I. Kaljurand , J. Saame , A. Heering , P. G. Plieger , R. Vianello , I. Leito , Eur. J. Org. Chem. 2019, 6735–6748.

[54] A. J. Arduengo III , H. R. Dias , R. L. Harlow , M. Kline , J. Am. Chem. Soc. 1992, 114, 5530–5534.

[55] R. Breslow , R. Kim , Tetrahedron Lett. 1994, 35, 699–702.

[56] R. Breslow , C. Schmuck , Tetrahedron Lett. 1996, 37, 8241–8242.

[57] Z. Kelemen , B. Péter-Szabó , E. Székely , O. Hollóczki , D. S. Firaha , B. Kirchner , J. Nagy , L. Nyulászi , Chem. Eur. J. 2014, 20, 13002–13008. 25137312 10.1002/chem.201402912

[58] F. Yan , N. R. Dhumal , H. Kim , J. Phys. Chem. Chem. Phys. 2017, 19, 1361–1368. 10.1039/c6cp06556b 27976766

[59] D. Rico del Cerro , R. Mera-Adasme , A. W. King , J. E. Perea-Buceta , S. Heikkinen , T. Hase , D. Sundholm , K. Wähälä , Angew. Chem. Int. Ed. 2018, 57, 11613–11617; 10.1002/anie.201805016 29987916

[60] N. V. Tzouras , F. Nahra , L. Falivene , L. Cavallo , M. Saab , K. van Hecke , A. Collado , J. Collett , J. Christopher , A. D. Smith , C. S. J. Cazin , S. P. Nolan , Chem. Eur. J. 2020, 26, 4515–4519. 32022329 10.1002/chem.202000564

[61] N. V. Tzouras , M. Saab , W. Janssens , T. Cauwenbergh , K. van Hecke , F. Nahra , S. P. Nolan , Chem. Eur. J. 2020, 26, 5541–5551. 32077182 10.1002/chem.202000876

[62] M. T. Clough , K. Geyer , P. A. Hunt , S. Son , U. Vagt , T. Welton , Green Chem. 2015, 17, 231–243.

[63] J. Blasius , R. Elfgen , O. Hollóczki , B. Kirchner , Phys. Chem. Chem. Phys. 2020, 22, 10726–10737. 32150178 10.1039/c9cp06798a

[64] S. Gehrke , W. Reckien , I. Palazzo , T. Welton , O. Hollóczki , Eur. J. Org. Chem. 2019, 504–511.

[65] F. Neese , WIREs Comput. Mol. Sci. 2012, 2, 73–78.

[66] F. Neese , WIREs Comput. Mol. Sci. 2018, 8, e1327.

[67] J. Tao , J. P. Perdew , V. N. Staroverov , G. E. Scuseria , Phys. Rev. Lett. 2003, 91, 146401. 14611541 10.1103/PhysRevLett.91.146401

[68] S. Grimme , J. Antony , S. Ehrlich , H. Krieg , J. Chem. Phys. 2010, 132, 154104. 20423165 10.1063/1.3382344

[69] S. Grimme , S. Ehrlich , L. Goerigk , J. Comput. Chem. 2011, 32, 1456–1465. 21370243 10.1002/jcc.21759

[70] A. Schäfer , H. Horn , R. Ahlrichs , J. Chem. Phys. 1992, 97, 2571–2577.

[71] F. Weigend , R. Ahlrichs , Phys. Chem. Chem. Phys. 2005, 7, 3297–3305. 16240044 10.1039/b508541a

[72] C. Riplinger , B. Sandhoefer , A. Hansen , F. Neese , J. Chem. Phys. 2013, 139, 134101. 24116546 10.1063/1.4821834

[73] D. G. Liakos , F. Neese , J. Chem. Theory Comput. 2015, 11, 4054–4063. 26575901 10.1021/acs.jctc.5b00359

[74] D. G. Liakos , M. Sparta , M. K. Kesharwani , J. M. Martin , F. Neese , J. Chem. Theory Comput. 2015, 11, 1525–1539. 26889511 10.1021/ct501129s

[75] D. G. Liakos , F. Neese , J. Phys. Chem. A 2012, 116, 4801–4816. 22489633 10.1021/jp302096v

[76] A. Klamt , J. Phys. Chem. 1995, 99, 2224–2235.

[77] A. Klamt , V. Jonas , T. Bürger , J. C. Lohrenz , J. Phys. Chem. A 1998, 102, 5074–5085.

[78] F. Eckert , A. Klamt , AIChE J. 2002, 48, 369–385.

[79] A. Klamt , WIREs Comput. Mol. Sci. 2018, 8, e1338.

[80] O. Treutler , R. Ahlrichs , J. Chem. Phys. 1995, 102, 346–354.

[81] R. Ahlrichs , M. Bär , M. Häser , H. Horn , C. Kölmel , Chem. Phys. Lett. 1989, 162, 165–169.

[82] E. R. Johnson , S. Keinan , P. Mori-Sánchez , J. Contreras-García , A. J. Cohen , W. Yang , J. Am. Chem. Soc. 2010, 132, 6498–6506. 20394428 10.1021/ja100936w PMC2864795

[83] J. Contreras-García , W. Yang , E. R. Johnson , J. Phys. Chem. A 2011, 115, 12983–12990. 21786796 10.1021/jp204278k PMC3651877

[84] E. Pastorczak , C. Corminboeuf , J. Chem. Phys. 2017, 146, 120901. 28388098 10.1063/1.4978951

[85] T. Lu , F. Chen , J. Comput. Chem. 2012, 33, 580–592. 22162017 10.1002/jcc.22885

[86] S. Plimpton , J. Comput. Phys. 1995, 117, 1–19.

[87] J. N. Canongia Lopes , J. Deschamps , A. A. Pádua , J. Phys. Chem. B 2004, 108, 2038–2047.

[88] J. N. Canongia Lopes , A. A. Pádua , J. Phys. Chem. B 2006, 110, 19586–19592. 17004824 10.1021/jp063901o

[89] W. L. Jorgensen , D. S. Maxwell , J. Tirado-Rives , J. Am. Chem. Soc. 1996, 118, 11225–11236.

[90] H. Berendsen , J. Grigera , T. Straatsma , J. Phys. Chem. 1987, 91, 6269–6271.

[91] L. Martínez , R. Andrade , E. G. Birgin , J. M. Martinez , J. Comput. Chem. 2009, 30, 2157–2164. 19229944 10.1002/jcc.21224

[92] M. S. Taylor , E. N. Jacobsen , Angew. Chem. Int. Ed. 2006, 45, 1520–1543; 10.1002/anie.200503132 16491487

[93] P. R. Schreiner , Chem. Soc. Rev. 2003, 32, 289–296. 14518182 10.1039/b107298f

[94] R. R. Knowles , E. N. Jacobsen , Proc. Natl. Acad. Sci. USA 2010, 107, 20678–20685. 20956302 10.1073/pnas.1006402107 PMC2996434

[95] A. G. Doyle , E. N. Jacobsen , Chem. Rev. 2007, 107, 5713–5743. 18072808 10.1021/cr068373r

[96] W. Cleland , M. M. Kreevoy , Science 1994, 264, 1887–1890. 8009219 10.1126/science.8009219

[97] S. B. Lehmann , M. Roatsch , M. Schöppke , B. Kirchner , Phys. Chem. Chem. Phys. 2010, 12, 7473–7486. 20532355 10.1039/b921246a

[98] O. Hollóczki , P. Terleczky , D. Szieberth , G. Mourgas , D. Gudat , L. Nyulászi , J. Am. Chem. Soc. 2010, 132, 780–789. 10.1021/ja103578y 21174475

[99] O. Hollóczki , Phys. Chem. Chem. Phys. 2016, 18, 126–140. 26592182 10.1039/c5cp05369b

[100] S. Gehrke , O. Hollóczki , Chem. Eur. J. 2018, 24, 11594–11604. 29882612 10.1002/chem.201802286

[101] S. Gehrke , O. Hollóczki , Phys. Chem. Chem. Phys. 2016, 18, 22070–22080. 27426687 10.1039/c6cp02624a

[102] M. Feroci , I. Chiarotto , F. D'Anna , G. Forte , R. Noto , A. Inesi , Electrochim. Acta 2015, 153, 122–129.

[103] M. H. Dunn , M. L. Cole , J. B. Harper , RSC Adv. 2012, 2, 10160–10162.

[104] H. Weber , O. Hollóczki , A. S. Pensado , B. Kirchner , J. Chem. Phys. 2013, 139, 084502. 24007013 10.1063/1.4818540

[105] M. Brehm , H. Weber , A. S. Pensado , A. Stark , B. Kirchner , Phys. Chem. Chem. Phys. 2012, 14, 5030–5044. 22389030 10.1039/c2cp23983c

[106] S. Tobisch , T. Ziegler , J. Am. Chem. Soc. 2004, 126, 9059–9071. 15264839 10.1021/ja048861l

[107] D. H. Wertz , J. Am. Chem. Soc. 1980, 102, 5316–5322.

[108] Y. B. Yu , P. L. Privalov , R. S. Hodges , Biophys. J. 2001, 81, 1632–1642. 11509376 10.1016/S0006-3495(01)75817-1 PMC1301641

