# Supplementary material for: N‐Heterocyclic Carbene Organocatalysis: With or Without Carbenes?
Source: Chemistry. 2020 Jul 23;26(44):10140–51. doi: 10.1002/chem.202002656 (PMC7496998; doi:10.1002/chem.202002656)
Supplement: Supplementary file 1 — Supplementary [file CHEM-26-10140-s001.pdf]

Chemistry–A European Journal

Supporting Information

N-Heterocyclic Carbene Organocatalysis: With or Without Carbenes?

Sascha Gehrke and Oldamur Hollóczki*[a]

Contents

1 Non-Covalent Interactions **S3**

2 Bond Length and Imaginary Frequencies in $TS_{2\text{-}IV}$ **S15**

3 Reaction Barriers with Different Bases **S18**

4 Potentials of Mean Force **S19**

5 Structures **S20**

5.1 Single Molecules . . . S20
5.1.1 Precursors/Carbenes . . . S20
5.1.2 Aldehydes . . . S26
5.1.3 Bases/protonated Bases . . . S30
5.1.4 Anions . . . S36
5.1.5 Catalysts . . . S38
5.2 Reactions: Precursor + Amine + Aldehyde . . . S48
5.2.1 Imidazolium . . . S48
5.2.2 Triazolium . . . S73
5.2.3 Thiazolium . . . S98
5.3 Reactions: Imidazole + Base + Aldehyde . . . S123
5.3.1 DABCO . . . S123
5.3.2 DBU . . . S135
5.4 Reactions: Catalyst + Amine + Aldehyde . . . S144
5.4.1 Catalyst 1 . . . S144
5.4.2 Catalyst 2 . . . S157
5.4.3 Catalyst 3 . . . S168
5.4.4 Catalyst 4 . . . S180
5.4.5 Catalyst 5 . . . S192
5.5 Reactions: Precursor + Amine + Formaldehyde + Chloride . . . S205
5.5.1 Imidazolium . . . S206
5.5.2 Triazolium . . . S210
5.5.3 Thiazolium . . . S214
5.6 Reactions: Precursor + Amine + Formaldehyde + Bromide . . . S218
5.6.1 Imidazolium . . . S219
5.6.2 Triazolium . . . S223
5.6.3 Thiazolium . . . S227
5.7 Reactions: Precursor + Amine + Formaldehyde + Iodide . . . S231
5.7.1 Imidazolium . . . S232
5.7.2 Triazolium . . . S236
5.7.3 Thiazolium . . . S240
5.8 Reactions: Precursor + Amine + Formaldehyde + Tetrafluoroborate . . . S244
5.8.1 Imidazolium . . . S245

5.8.2 Triazolium S248
5.8.3 Thiazolium S252
5.9 Reactions: Precursor + Amine + Formaldehyde + Triflate S255
5.9.1 Imidazolium S256
5.9.2 Triazolium S260
5.9.3 Thiazolium S264
5.10 Reactions: Thiamine S268
5.10.1 Pyruvic Acid S268
5.10.2 Glyceraldehyde S271

1 Non-Covalent Interactions

Imidazolium + Formaldehyde

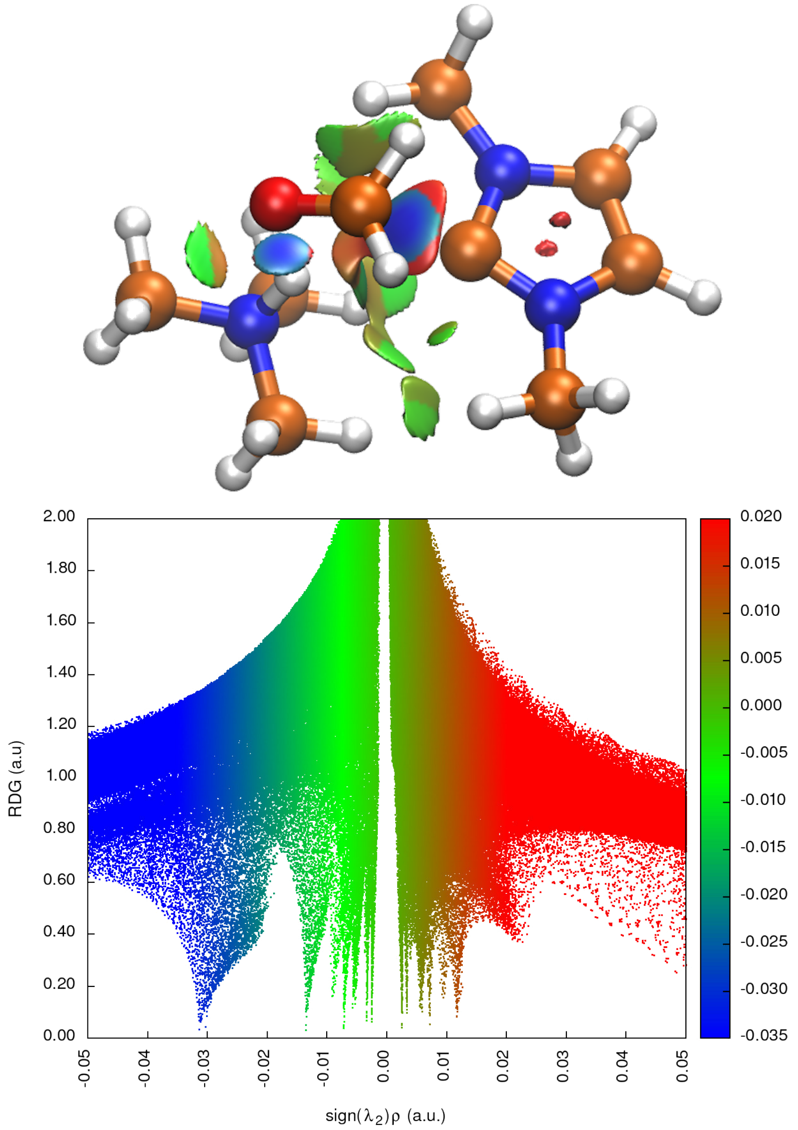

Figure S1: Non-Covalent Interaction Analysis of the associative transition state of the reaction between imidazolium and formaldehyde. At the shown isosurface the reduced density gradient becomes 0.5 a.u.. The color scheme illustrates the electron density (or the negative electron density if the second largest eigenvalue of the electron density's Hessian is negative) goes from blue (-0.035 a.u.) over green to red (0.02 a.u.).

Imidazolium + Acetaldehyde

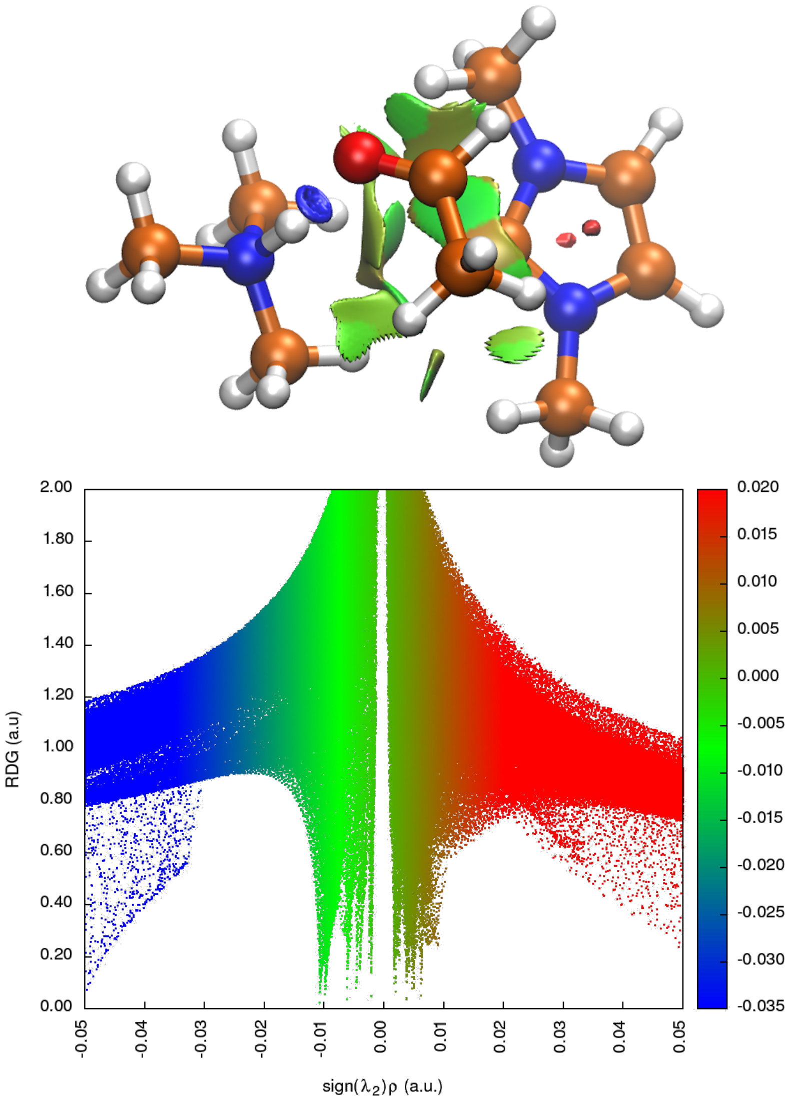

Figure S2: Non-Covalent Interaction Analysis of the associative transition state of the reaction between imidazolium and acetaldehyde. At the shown isosurface the reduced density gradient becomes 0.5 a.u.. The color scheme illustrates the electron density (or the negative electron density if the second largest eigenvalue of the electron density's Hessian is negative) goes from blue (-0.035 a.u.) over green to red (0.02 a.u.).

Imidazolium + Benzaldehyde

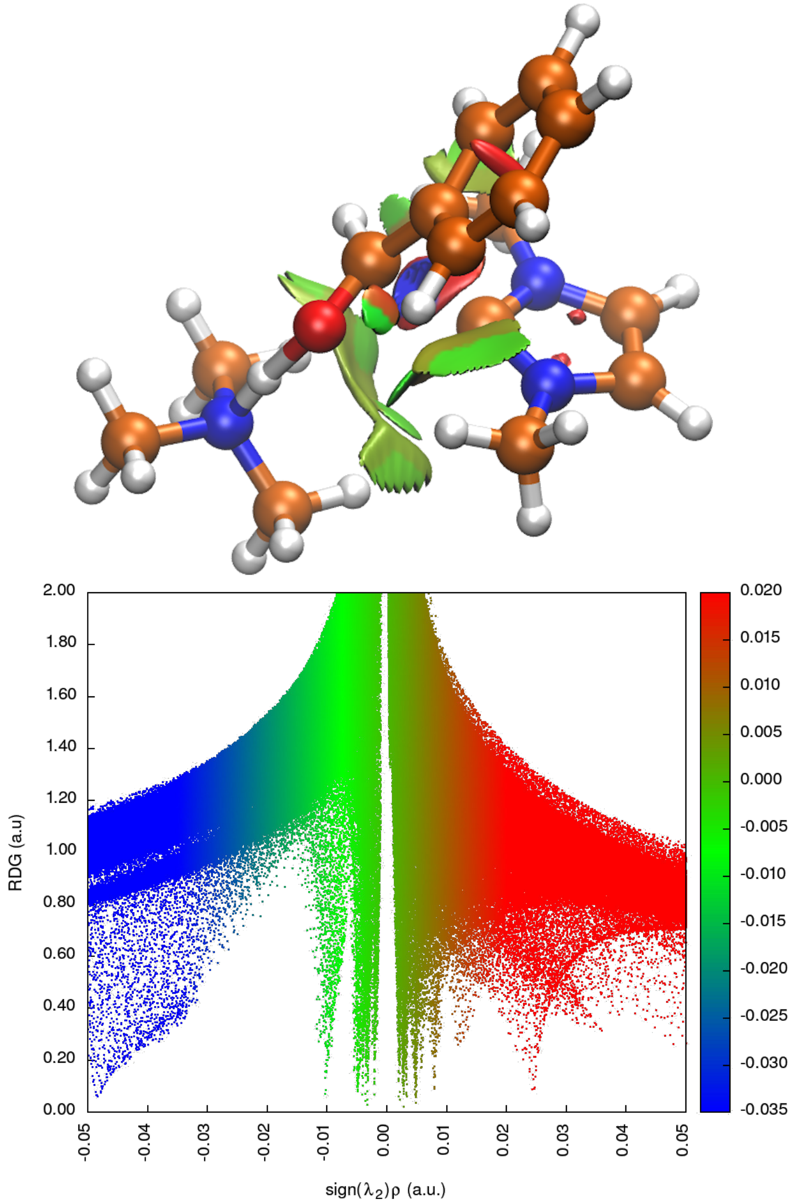

Figure S3: Non-Covalent Interaction Analysis of the associative transition state of the reaction between imidazolium and benzaldehyde. At the shown isosurface the reduced density gradient becomes 0.5 a.u.. The color scheme illustrates the electron density (or the negative electron density if the second largest eigenvalue of the electron density's Hessian is negative) goes from blue (-0.035 a.u.) over green to red (0.02 a.u.).

Imidazolium + Acrolein

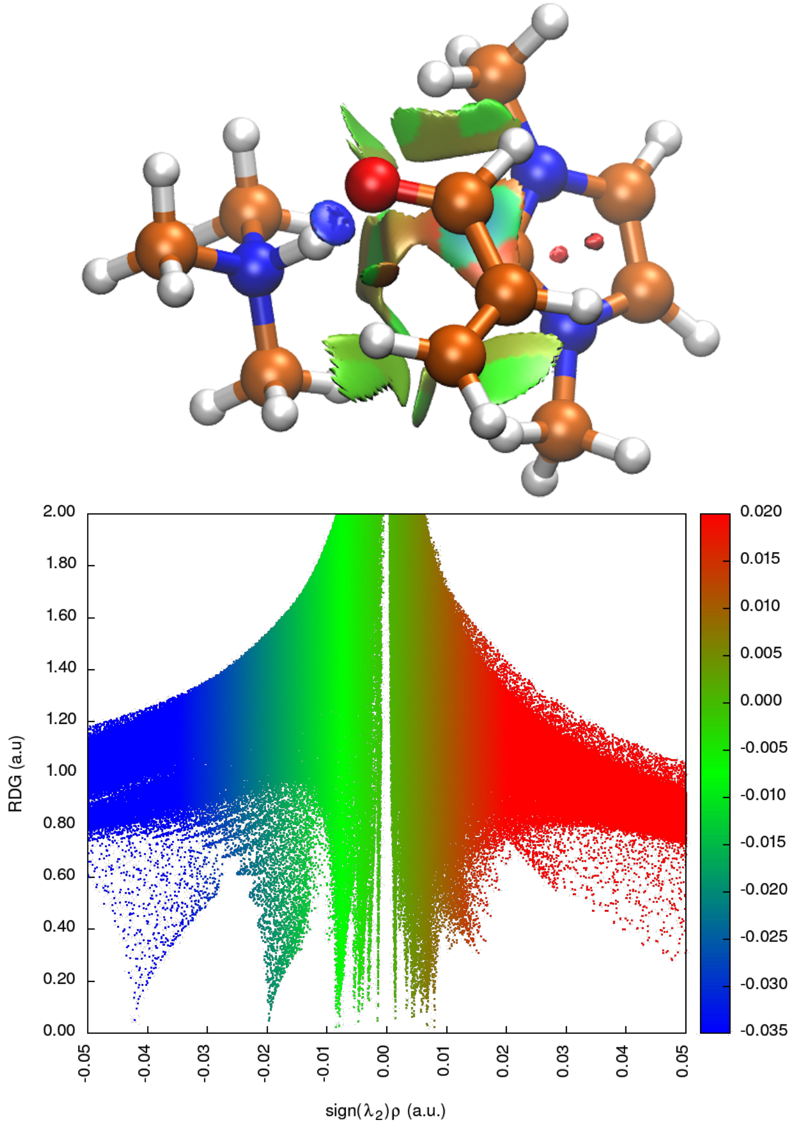

Figure S4: Non-Covalent Interaction Analysis of the associative transition state of the reaction between imidazolium and acrolein. At the shown isosurface the reduced density gradient becomes 0.5 a.u.. The color scheme illustrates the electron density (or the negative electron density if the second largest eigenvalue of the electron density's Hessian is negative) goes from blue (-0.035 a.u.) over green to red (0.02 a.u.).

Triazolium + Formaldehyde

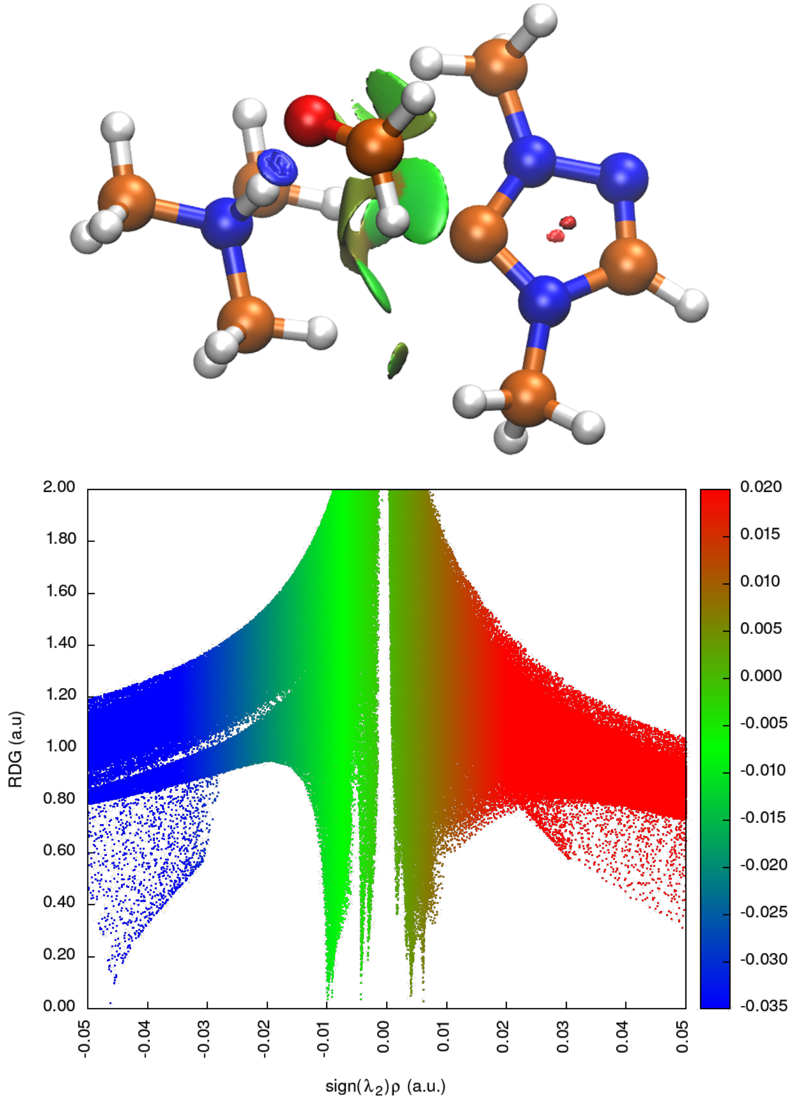

Figure S5: Non-Covalent Interaction Analysis of the associative transition state of the reaction between triazolium and formaldehyde. At the shown isosurface the reduced density gradient becomes 0.5 a.u.. The color scheme illustrates the electron density (or the negative electron density if the second largest eigenvalue of the electron density's Hessian is negative) goes from blue (-0.035 a.u.) over green to red (0.02 a.u.).

Triazolium + Acetaldehyde

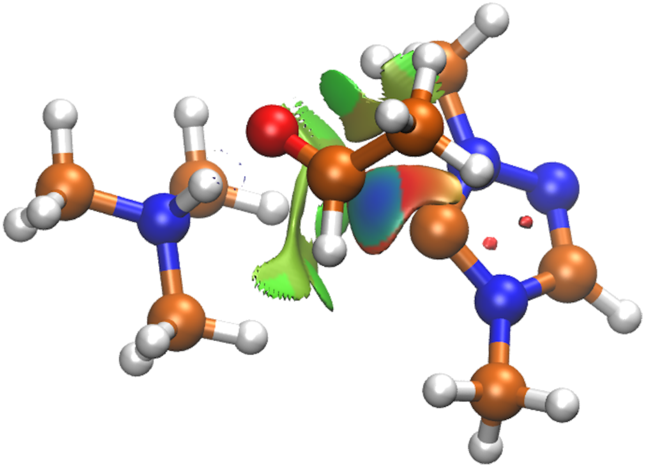

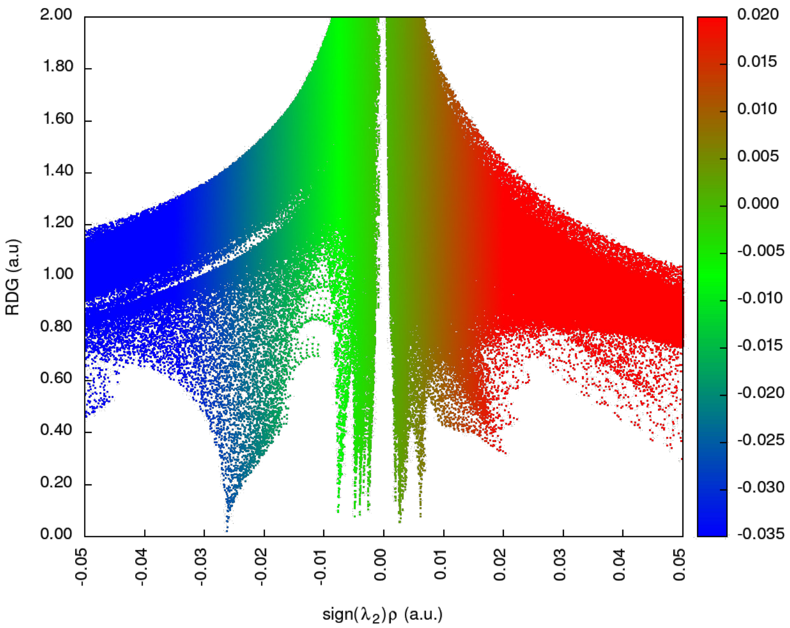

Figure S6: Non-Covalent Interaction Analysis of the associative transition state of the reaction between triazolium and acetaldehyde. At the shown isosurface the reduced density gradient becomes 0.5 a.u.. The color scheme illustrates the electron density (or the negative electron density if the second largest eigenvalue of the electron density's Hessian is negative) goes from blue (-0.035 a.u.) over green to red (0.02 a.u.).

Triazolium + Benzaldehyde

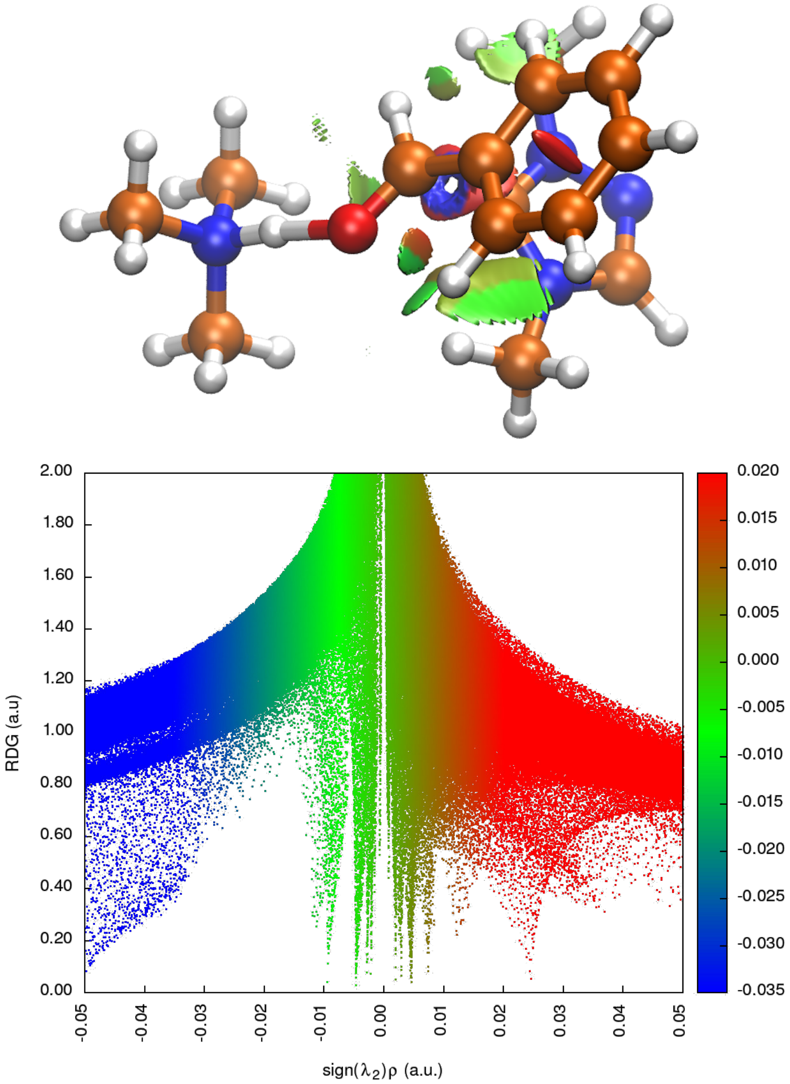

Figure S7: Non-Covalent Interaction Analysis of the associative transition state of the reaction between triazolium and benzaldehyde. At the shown isosurface the reduced density gradient becomes 0.5 a.u.. The color scheme illustrates the electron density (or the negative electron density if the second largest eigenvalue of the electron density's Hessian is negative) goes from blue (-0.035 a.u.) over green to red (0.02 a.u.).

Triazolium + Acrolein

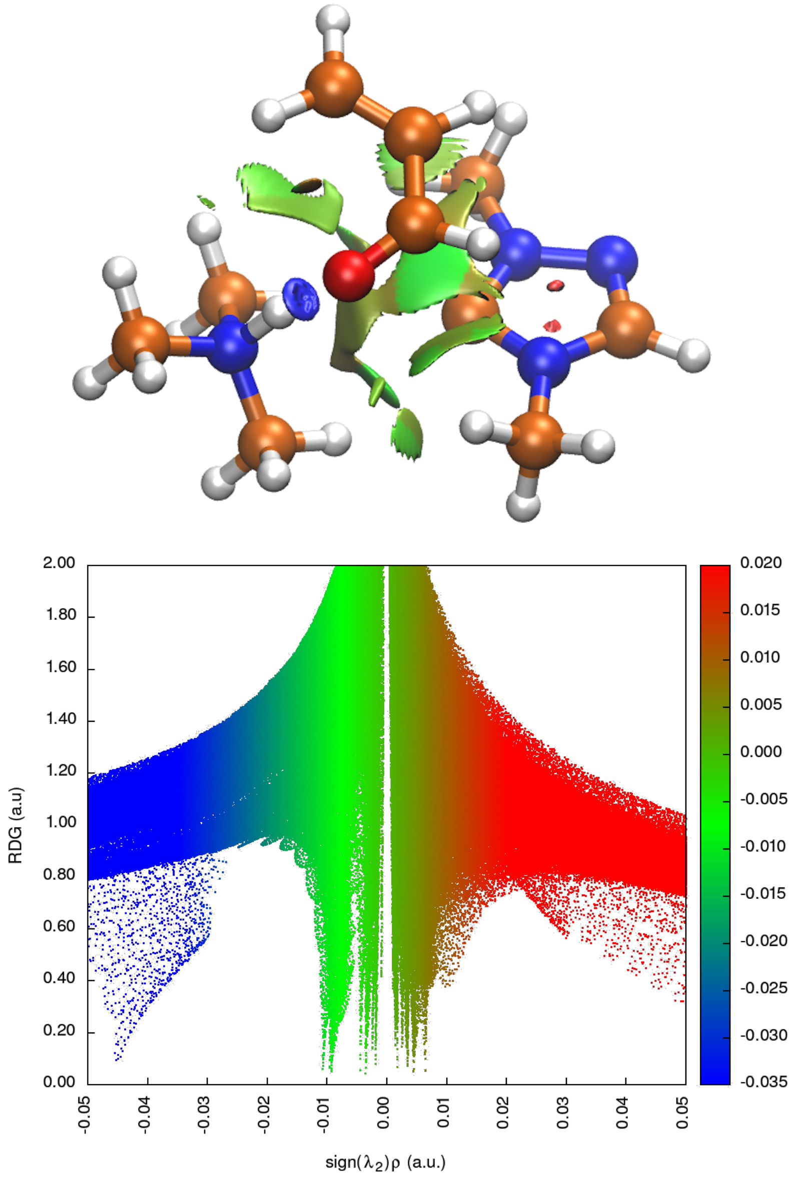

Figure S8: Non-Covalent Interaction Analysis of the associative transition state of the reaction between triazolium and acrolein. At the shown isosurface the reduced density gradient becomes 0.5 a.u.. The color scheme illustrates the electron density (or the negative electron density if the second largest eigenvalue of the electron density's Hessian is negative) goes from blue (-0.035 a.u.) over green to red (0.02 a.u.).

Thiazolium + Formaldehyde

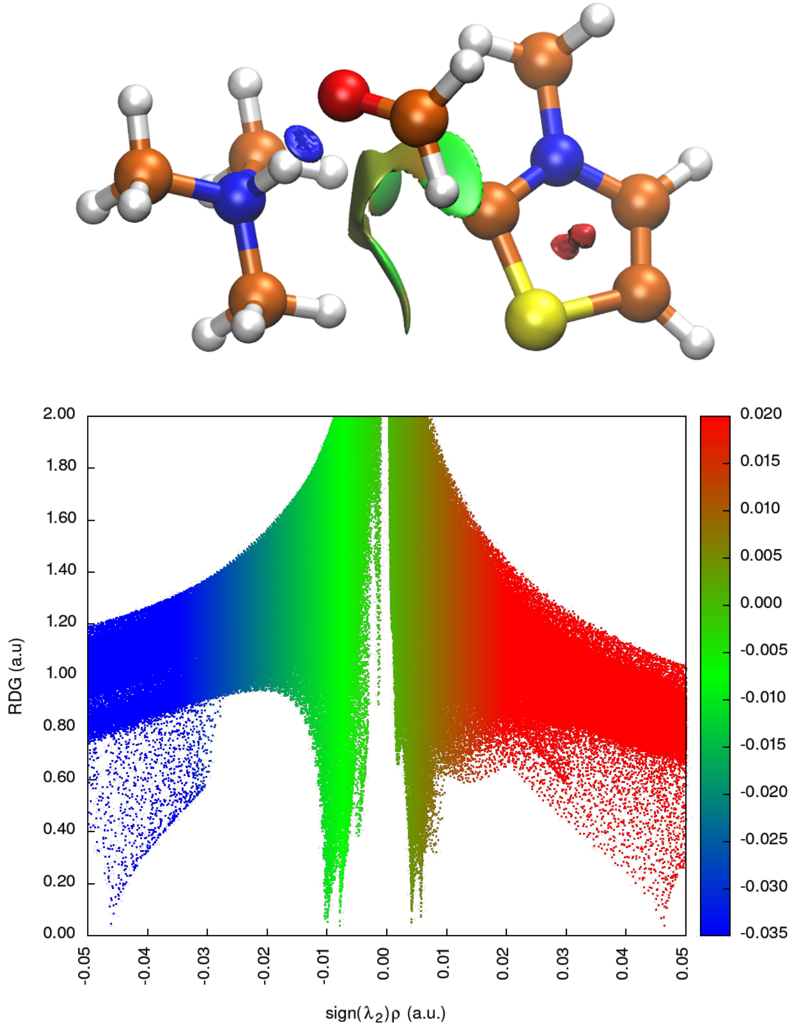

Figure S9: Non-Covalent Interaction Analysis of the associative transition state of the reaction between thiazolium and formaldehyde. At the shown isosurface the reduced density gradient becomes 0.5 a.u.. The color scheme illustrates the electron density (or the negative electron density if the second largest eigenvalue of the electron density's Hessian is negative) goes from blue (-0.035 a.u.) over green to red (0.02 a.u.).

Thiazolium + Acetaldehyde

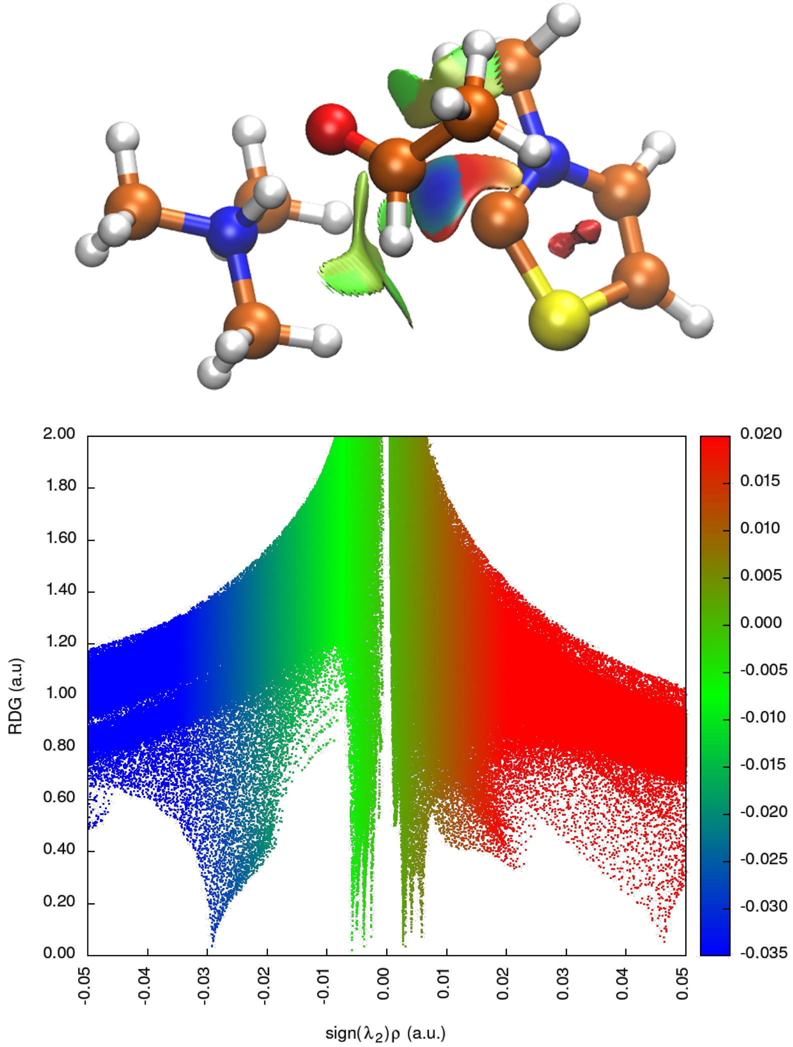

Figure S10: Non-Covalent Interaction Analysis of the associative transition state of the reaction between thiazolium and acetaldehyde. At the shown isosurface the reduced density gradient becomes 0.5 a.u.. The color scheme illustrates the electron density (or the negative electron density if the second largest eigenvalue of the electron density's Hessian is negative) goes from blue (-0.035 a.u.) over green to red (0.02 a.u.).

Thiazolium + Benzaldehyde

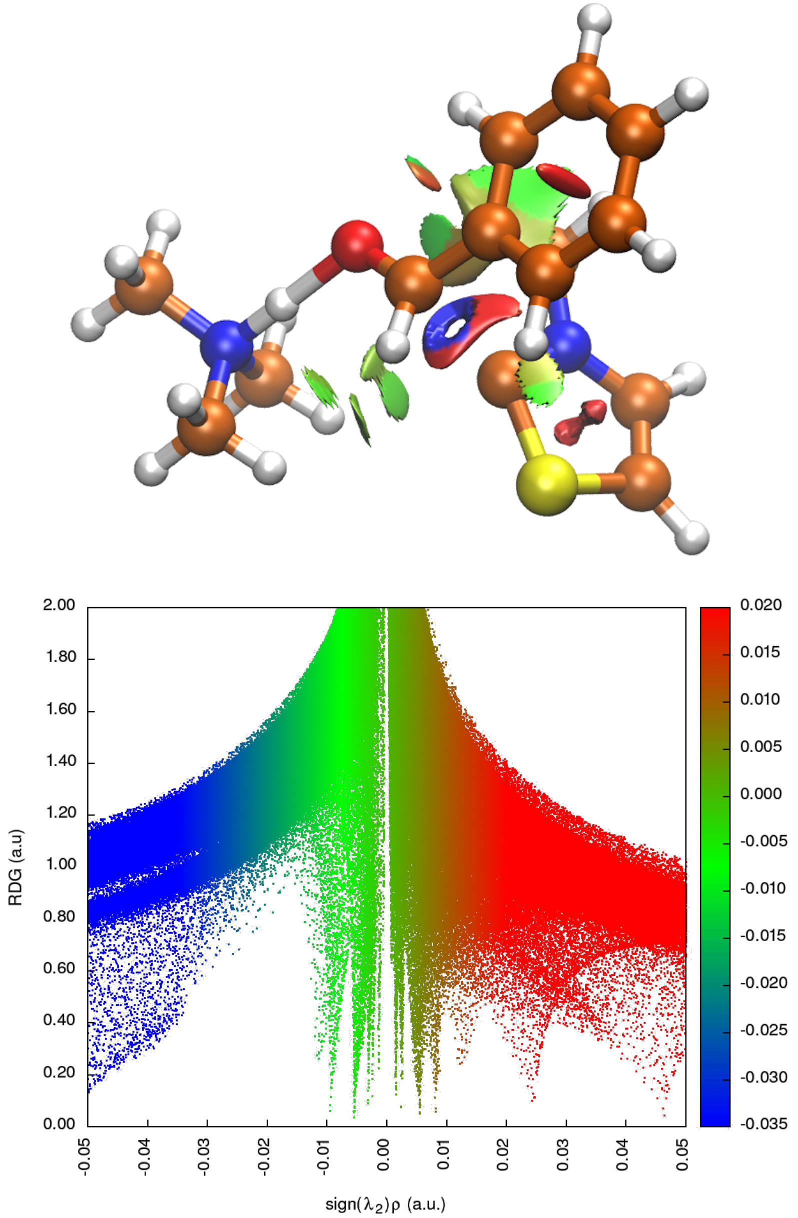

Figure S11: Non-Covalent Interaction Analysis of the associative transition state of the reaction between thiazolium and benzaldehyde. At the shown isosurface the reduced density gradient becomes 0.5 a.u.. The color scheme illustrates the electron density (or the negative electron density if the second largest eigenvalue of the electron density's Hessian is negative) goes from blue (-0.035 a.u.) over green to red (0.02 a.u.).

Thiazolium + Acrolein

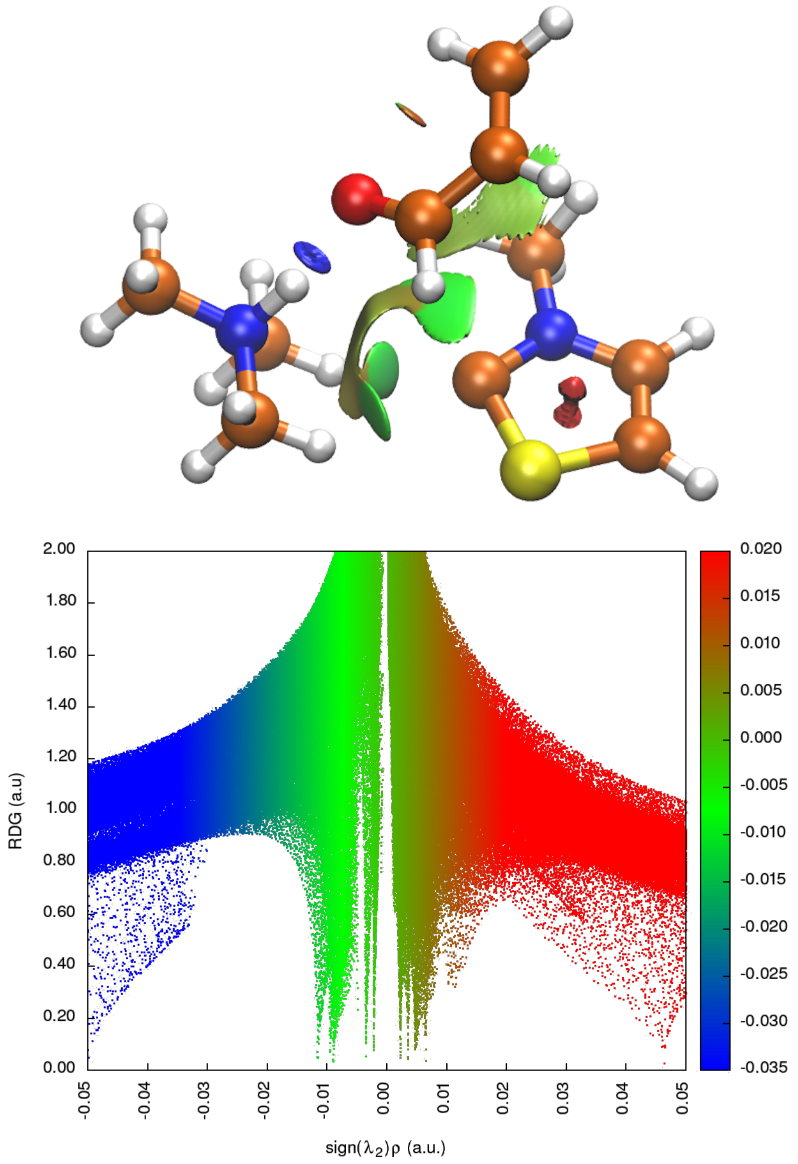

Figure S12: Non-Covalent Interaction Analysis of the associative transition state of the reaction between thiazolium and acrolein. At the shown isosurface the reduced density gradient becomes 0.5 a.u.. The color scheme illustrates the electron density (or the negative electron density if the second largest eigenvalue of the electron density's Hessian is negative) goes from blue (-0.035 a.u.) over green to red (0.02 a.u.).

2 Bond Length and Imaginary Frequencies in $TS_{2\text{-}IV}$

Table 1: Imaginary frequencies and distances between the moving hydrogen atom and interacting atoms for the associative transition states of the reactions between the model catalysts and different aldehydes activated by different bases.

catalyst	deprotonating agent	aldehyde R-CHO	frequency / cm^{-1}	C–H /Å	N–H /Å	O–H /Å
**A**	TMA	H	−117.60	2.480	1.039	1.871
**A**	TMA	CH_3	−53.57	3.344	1.050	1.668
**A**	TMA	CH_2=CH	−69.13	2.797	1.044	1.721
**A**	TMA	Ph	−125.36	3.318	1.107	1.468
**B**	TMA	H	−95.81	3.256	1.049	1.716
**B**	TMA	CH_3	−44.15	3.447	1.076	1.565
**B**	TMA	CH_2=CH	−42.13	3.176	1.046	1.702
**B**	TMA	Ph	−115.24	3.537	1.106	1.470
**C**	TMA	H	−96.65	3.227	1.049	1.715
**C**	TMA	CH_3	−55.74	3.420	1.080	1.553
**C**	TMA	CH_2=CH	−24.03	3.138	1.054	1.687
**C**	TMA	Ph	−118.84	3.406	1.110	1.464
**A**	DABCO	H	−129.06	2.482	1.036	1.851
**A**	DABCO	CH_3	−46.09	3.471	1.045	1.691
**A**	DABCO	CH_2=CH	−30.70	2.866	1.045	1.770
**A**	DABCO	Ph	−100.74	3.197	1.078	1.522
**A**	DBU	H	−85.47	2.492	1.021	2.136
**A**	DBU	CH_3	−40.10	3.021	1.021	2.002
**A**	DBU	CH_2=CH	−53.90	2.890	1.020	2.043

Table 2: Imaginary frequencies and distances between the moving hydrogen atom and interacting atoms for the associative transition states of the reactions between the model catalysts and formaldehyde activated by trimethylamine in the presence of different anions.

anion	catalyst	frequency /cm^{-1}	C–H /Å	N–H /Å	O–H /Å
BF_4^-	**A**	−43.19	2.029	1.058	3.488
BF_4^-	**B**	−53.61	2.592	1.039	3.070
BF_4^-	**C**	−48.18	2.453	1.041	3.130
Br^-	**A**	−50.20	2.579	1.046	2.848
Br^-	**B**	−82.32	2.270	1.032	2.284
Br^-	**C**	−97.18	2.251	1.032	2.341
Cl^-	**A**	−57.95	2.420	1.047	2.841
Cl^-	**B**	−48.59	2.244	1.032	2.502
Cl^-	**C**	−76.17	2.052	1.051	3.243
I^-	**A**	−78.94	2.245	1.054	3.787
I^-	**B**	−77.56	2.225	1.053	3.739
I^-	**C**	−124.86	2.246	1.034	2.238
OTf^-	**A**	−43.74	2.088	1.054	3.688
OTf^-	**B**	−41.14	2.528	1.039	2.865
OTf^-	**C**	−44.12	2.023	1.051	3.420

Table 3: Imaginary frequencies and distances between the moving hydrogen atom and interacting atoms for the associative transition states of the reactions between the more complicated catalysts and different aldehydes activated by trimethylamine and for the self-activated reactions of thiamine with pyruvate and glyceraldehyde.

catalyst	aldehyde R-CHO	frequency / cm^{-1}	C–H /Å	N–H /Å	O–H /Å
**1**	H	−51.68	2.450	1.039	1.852
**1**	CH_3	−137.97	3.384	1.051	1.666
**2**	H	−93.79	3.260	1.049	1.711
**2**	CH_3	−41.06	3.411	1.078	1.559
**3**	H	−108.42	2.580	1.036	1.890
**3**	CH_3	−72.27	2.876	1.045	1.794
**4**	H	−110.72	2.537	1.036	1.955
**4**	CH_3	−75.83	2.890	1.046	1.792
**5**	H	−100.10	2.758	1.042	1.751
**5**	CH_3	−103.86	3.613	1.119	1.441
thiamine	pyruvate	−159.65	2.435	1.022	1.985
thiamine	glyceral	−144.65	2.382	1.022	2.014

3 Reaction Barriers with Different Bases

Table 4: DLPNO-CCSD(T)/CBS//TPSSh/def2-TZVPP activation enthalpies and activation Gibbs free energies for the reaction of the 1,3-dimethylimidazolium cation and different deprotonating agents with different aldehydes in the gas phase through the associative ($\Delta H^{\ddagger}_{assoc}$, $\Delta G^{\ddagger}_{assoc}$) and dissociative ($\Delta H^{\ddagger}_{dissoc}$, $\Delta G^{\ddagger}_{dissoc}$) reaction mechanisms.

deprotonating agent	aldehyde R-CHO	$\Delta G^{\ddagger}_{assoc}$ kcal mol^{-1}	$\Delta G^{\ddagger}_{dissoc}$ kcal mol^{-1}	$\Delta H^{\ddagger}_{assoc}$ kcal mol^{-1}	$\Delta H^{\ddagger}_{dissoc}$ kcal mol^{-1}
trimethylamine	H	26.1	46.6	22.1	46.0
trimethylamine	CH_3	23.2	50.2	21.5	48.5
trimethylamine	$CH_2{=}CH$	23.5	48.6	20.4	45.8
trimethylamine	Ph	26.6	52.6	24.9	47.4
DABCO	H	23.8	43.1	20.6	50.3
DABCO	CH_3	22.3	46.5	18.7	52.6
DABCO	$CH_2{=}CH$	19.3	45.9	17.3	52.3
DABCO	Ph	24.6	50.7	21.8	56.6
DBU	H	16.8	28.6	12.4	36.1
DBU	CH_3	14.1	32.9	11.6	41.1
DBU	$CH_2{=}CH$	15.5	31.5	12.0	38.3

4 Potentials of Mean Force

Table 5: Box compositions and sizes of the potentials of mean force calculations. Each box contained a pair of 1,3-dimethylimidazolium cation and the respective anion together with the given number of solvent molecules.

Anion	Solvent	cell vector / Å	Number of Solvent Molecules
OTf^-	water	39.3054	2000
	ethanol	40.1142	650
	THF	40.3089	450
	ether	39.7777	350
	toluene	39.8968	350
	hexane	40.9030	300
Br^-	ethanol	40.0944	650
	hexane	40.9494	300

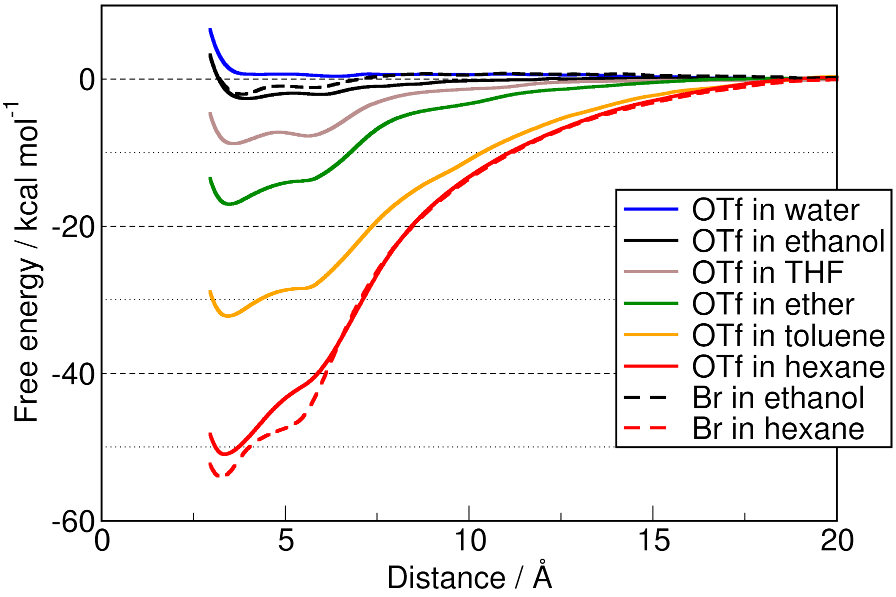

Figure S13: Potentials of mean force between the 1,3-dimethylimidazolium cation and a triflate or a bromide anion, respectively, in different solvents.

5 Structures

5.1 Single Molecules

5.1.1 Precursors/Carbenes

1,3-dimethylimidazolium

E (TPSSh/def2-TZVP) =	−305.37769950
$G - E$ (TPSSh/def2-TZVP) =	0.11014192
$H - E$ (TPSSh/def2-TZVP) =	0.14826639
E (DLPNO-CCSD(T)tight/def-TZVPP//TPSSh/def2-TZVP) =	−303.38564931
E (DLPNO-CCSD(T)tight/def-QZVPP//TPSSh/def2-TZVP) =	−303.39906355
E (DLPNO-CCSD(T)/CBS//TPSSh/def2-TZVP) =	−304.826543954

N	−0.02995012	0.00706392	−0.05985675
C	0.01966443	0.17105006	1.30959716
C	1.33137160	0.16422554	1.66536125
N	2.06453473	−0.00380677	0.50822182
C	1.21879756	−0.09626874	−0.52066195
H	1.49780875	−0.23181754	−1.55198364
C	3.52995559	−0.07027824	0.42226357
H	1.80117878	0.26302919	2.62871707
H	−0.87154861	0.27695291	1.90379300
H	3.81095466	−0.20609755	−0.61922994
H	3.95228621	0.85935812	0.80012455
H	3.88438202	−0.91336024	1.01300636
C	−1.25169183	−0.04525176	−0.87466689
H	−1.86441769	−0.88310001	−0.54609449
H	−1.79781461	0.88957598	−0.75967656
H	−0.96925847	−0.18127485	−1.91574855

1,3-dimethylimidazol-2-ylidene

E (TPSSh/def2-TZVP) =	−304.94448115
$G - E$ (TPSSh/def2-TZVP) =	0.09614253
$H - E$ (TPSSh/def2-TZVP) =	0.13400452
E (DLPNO-CCSD(T)tight/def-TZVPP//TPSSh/def2-TZVP) =	−302.95245817
E (DLPNO-CCSD(T)tight/def-QZVPP//TPSSh/def2-TZVP) =	−302.96589974
E (DLPNO-CCSD(T)/CBS//TPSSh/def2-TZVP) =	−304.400077156

C	−0.10454145	0.00392711	−0.10941805
N	0.02870582	0.16240094	1.24171888
C	1.34955330	0.16289401	1.66249452
C	2.09762278	−0.00083827	0.54622060
N	1.19989411	−0.09395001	−0.50599363
C	−1.11136430	0.31324656	2.12769141
C	1.58364611	−0.27662598	−1.89411137
H	3.16590585	−0.05718626	0.41784040
H	1.64086947	0.27663531	2.69355646
H	0.66781515	−0.31222342	−2.47907630
H	2.20444150	0.55575299	−2.23414713
H	2.13660854	−1.21050700	−2.02046818
H	−1.13594044	−0.49409637	2.86353736
H	−1.06838296	1.27214407	2.64974861
H	−2.00580848	0.27279532	1.51082141

1,4-dimethyltriazolium

E (TPSSh/def2-TZVP) =	−321.40106954
$G - E$ (TPSSh/def2-TZVP) =	0.09797508
$H - E$ (TPSSh/def2-TZVP) =	0.13608254
E (DLPNO-CCSD(T)tight/def-TZVPP//TPSSh/def2-TZVP) =	−319.36487983
E (DLPNO-CCSD(T)tight/def-QZVPP//TPSSh/def2-TZVP) =	−319.37903047
E (DLPNO-CCSD(T)/CBS//TPSSh/def2-TZVP) =	−320.842886828

C	−0.06969876	0.00313824	−0.10332960
N	−0.00513920	0.15965852	1.20909020
N	1.28234482	0.16263572	1.63999433
C	1.99112147	0.00445282	0.55554130
N	1.18963147	−0.09927741	−0.55232219
C	−1.10144772	0.31785635	2.16915477
C	1.61984797	−0.28474759	−1.94939722
H	−0.96464729	−0.03527847	−0.70255018
H	3.06790255	−0.04031554	0.52419267
H	0.73559756	−0.32666190	−2.58019739
H	2.24427312	0.55709487	−2.24288622
H	2.17552624	−1.21732542	−2.02854078
H	−1.04860542	−0.49717595	2.88819124
H	−0.98051069	1.27418396	2.67402345
H	−2.04176913	0.28805778	1.62367563

1,4-dimethyltriazol-2-ylidene

E (TPSSh/def2-TZVP) =	−320.98645240
$G - E$ (TPSSh/def2-TZVP) =	0.08443308
$H - E$ (TPSSh/def2-TZVP) =	0.12239504
E (DLPNO-CCSD(T)tight/def-TZVPP//TPSSh/def2-TZVP) =	−318.95045628
E (DLPNO-CCSD(T)tight/def-QZVPP//TPSSh/def2-TZVP) =	−318.96447494
E (DLPNO-CCSD(T)/CBS//TPSSh/def2-TZVP) =	−320.435485773

N	−0.05334910	0.00523107	−0.08323698
C	−0.06943212	0.17121263	1.28253916
N	1.25381022	0.15157342	1.53892713
N	2.08360798	−0.01238724	0.44726442
C	1.23680870	−0.09893498	−0.53486994
C	1.86258445	0.28672533	2.84750336
C	−1.25080692	−0.04886004	−0.90664331
H	1.51543923	−0.23474557	−1.56797820
H	−2.10293701	0.06545280	−0.24157788
H	−1.24805334	0.75941754	−1.64052957
H	−1.31669164	−1.00759938	−1.42471173
H	2.44804511	−0.60421463	3.07859317
H	2.51573730	1.16024162	2.86486311
H	1.05673116	0.40481843	3.56721624

3-methylthiazolium

E (TPSSh/def2-TZVP) =	−608.86809896
$G - E$ (TPSSh/def2-TZVP) =	0.06761545
$H - E$ (TPSSh/def2-TZVP) =	0.10270637
E (DLPNO-CCSD(T)tight/def-TZVPP//TPSSh/def2-TZVP) =	−606.79944670
E (DLPNO-CCSD(T)tight/def-QZVPP//TPSSh/def2-TZVP) =	−606.81973312
E (DLPNO-CCSD(T)/CBS//TPSSh/def2-TZVP) =	−608.02263549

S	1.33155456	−0.14392048	−0.88089159
C	−0.14195861	0.00835762	−0.08488458
N	0.00150457	0.16153723	1.22561608
C	1.31996725	0.16101685	1.63612906
C	2.18045802	0.00308605	0.60229423
C	−1.12878625	0.31816839	2.16413645
H	−1.10513665	−0.01390603	−0.57168031
H	3.25794924	−0.03379992	0.63662719
H	1.55294759	0.27888956	2.68331512
H	−1.09979802	−0.49731556	2.88509602
H	−1.03188361	1.27712421	2.67074015
H	−2.05639107	0.28544908	1.59931517

3-methylthiazol-2-ylidene

E (TPSSh/def2-TZVP) =	−608.45277233
$G - E$ (TPSSh/def2-TZVP) =	0.05404670
$H - E$ (TPSSh/def2-TZVP) =	0.08901391
E (DLPNO-CCSD(T)tight/def-TZVPP//TPSSh/def2-TZVP) =	−606.38550442
E (DLPNO-CCSD(T)tight/def-QZVPP//TPSSh/def2-TZVP) =	−606.40568654
E (DLPNO-CCSD(T)/CBS//TPSSh/def2-TZVP) =	−607.6139631

C	1.35001793	−0.14670387	−0.89840318
S	−0.14335439	0.01495199	−0.03306881
C	0.57430867	0.17532904	1.51672415
N	1.90404647	0.09787256	1.29497937
C	2.36140576	−0.07754784	−0.00968092
C	2.85154688	0.19682776	2.40742845
H	1.42637359	−0.27896830	−1.96605480
H	3.42257595	−0.14143467	−0.20385242
H	3.44705620	−0.71580892	2.47448880
H	3.51345790	1.05260641	2.26059016
H	2.26894504	0.32844684	3.31454019

5.1.2 Aldehydes

formaldehyde

E (TPSSh/def2-TZVP) =	−114.55390631
$G - E$ (TPSSh/def2-TZVP) =	0.00585770
$H - E$ (TPSSh/def2-TZVP) =	0.03029151
E (DLPNO-CCSD(T)tight/def-TZVPP//TPSSh/def2-TZVP) =	−113.91711611
E (DLPNO-CCSD(T)tight/def-QZVPP//TPSSh/def2-TZVP) =	−113.92255254
E (DLPNO-CCSD(T)/CBS//TPSSh/def2-TZVP) =	−114.388782982

C	−0.00000002	−0.00000053	0.02591470
O	0.00000003	0.00000017	1.22838796
H	0.93960276	0.00000018	−0.56165131
H	−0.93960277	0.00000018	−0.56165135

acetaldehyde

E (TPSSh/def2-TZVP) = −153.90198371
$G - E$ (TPSSh/def2-TZVP) = 0.03127385
$H - E$ (TPSSh/def2-TZVP) = 0.06001143
E (DLPNO-CCSD(T)tight/def-TZVPP//TPSSh/def2-TZVP) = −152.98076176
E (DLPNO-CCSD(T)tight/def-QZVPP//TPSSh/def2-TZVP) = −152.98802598
E (DLPNO-CCSD(T)/CBS//TPSSh/def2-TZVP) = −153.660034158

C	−0.01339350	0.00318079	0.05845498
O	−0.13942736	−0.49300360	1.15155848
C	1.25415322	−0.01384383	−0.74771123
H	−0.87173543	0.52049886	−0.42400690
H	1.06462061	−0.49482067	−1.71334783
H	1.55996580	1.01513435	−0.96561095
H	2.04719666	−0.53714489	−0.21559255

acrolein

E (TPSSh/def2-TZVP) =	−191.99870407
$G - E$ (TPSSh/def2-TZVP) =	0.03560563
$H - E$ (TPSSh/def2-TZVP) =	0.06638931
E (DLPNO-CCSD(T)tight/def-TZVPP//TPSSh/def2-TZVP) =	−190.83671200
E (DLPNO-CCSD(T)tight/def-QZVPP//TPSSh/def2-TZVP) =	−190.84520243
E (DLPNO-CCSD(T)/CBS//TPSSh/def2-TZVP) =	−191.68310578

C	−0.00174691	0.00001223	−0.00787069
O	−0.11849284	−0.00000210	1.19917138
C	1.29421871	0.00000454	−0.72523000
C	2.45705904	0.00000031	−0.07244640
H	1.25463961	0.00000138	−1.81051818
H	3.40697222	−0.00000529	−0.59310059
H	2.47103587	0.00000303	1.01204177
H	−0.90119468	−0.00001411	−0.65671229

benzaldehyde

E (TPSSh/def2-TZVP) =	−345.73783123
$G - E$ (TPSSh/def2-TZVP) =	0.07961015
$H - E$ (TPSSh/def2-TZVP) =	0.11645049
E (DLPNO-CCSD(T)tight/def-TZVPP//TPSSh/def2-TZVP) =	−343.55928835
E (DLPNO-CCSD(T)tight/def-QZVPP//TPSSh/def2-TZVP) =	−343.57363590
E (DLPNO-CCSD(T)/CBS//TPSSh/def2-TZVP) =	−345.118973246

C	−0.00508028	−0.00001221	0.03505769
O	−0.07357363	0.00002237	1.24551819
C	1.25135301	−0.00000692	−0.73987872
H	−0.92750510	0.00000546	−0.58431749
C	1.19533430	−0.00000620	−2.13514893
C	2.36750937	0.00000172	−2.88149495
C	3.59752854	0.00000466	−2.23017864
C	3.65860616	0.00000177	−0.83554737
C	2.49026979	−0.00000188	−0.09044995
H	0.22860047	−0.00000813	−2.62926123
H	2.32554061	0.00000561	−3.96429142
H	4.51392437	0.00001017	−2.80939548
H	4.62106826	0.00000374	−0.33737171
H	2.50792313	−0.00000616	0.99294001

5.1.3 Bases/protonated Bases

trimethylamine

E (TPSSh/def2-TZVP) =	−174.55921888
$G - E$ (TPSSh/def2-TZVP) =	0.09317127
$H - E$ (TPSSh/def2-TZVP) =	0.12603704
E (DLPNO-CCSD(T)tight/def-TZVPP//TPSSh/def2-TZVP) =	−173.33839010
E (DLPNO-CCSD(T)tight/def-QZVPP//TPSSh/def2-TZVP) =	−173.34708607
E (DLPNO-CCSD(T)/CBS//TPSSh/def2-TZVP) =	−174.246358676

N	0.01563110	−0.03893548	0.01827331
C	−0.00587251	−0.00014156	1.47164183
C	1.37766441	0.00322568	−0.48902308
C	−0.69863712	−1.19999738	−0.48768354
H	1.36057202	0.02372043	−1.58103123
H	1.87215501	0.91009455	−0.13367608
H	1.98116243	−0.86704555	−0.16935488
H	−0.70906836	−1.17551462	−1.57969447
H	−0.24321276	−2.15613202	−0.16804703
H	−1.73094839	−1.17802865	−0.13125058
H	−1.04047866	0.01792818	1.82158271
H	0.49891010	−0.87070190	1.93108926
H	0.49326873	0.90668330	1.82053378

trimethylammonium

E (TPSSh/def2-TZVP) =	−174.93727261
$G - E$ (TPSSh/def2-TZVP) =	0.10875127
$H - E$ (TPSSh/def2-TZVP) =	0.14209043
E (DLPNO-CCSD(T)tight/def-TZVPP//TPSSh/def2-TZVP) =	−173.72221821
E (DLPNO-CCSD(T)tight/def-QZVPP//TPSSh/def2-TZVP) =	−173.73077235
E (DLPNO-CCSD(T)/CBS//TPSSh/def2-TZVP) =	−174.621718538

C	−0.01040682	0.00899669	1.52449853
N	0.01430760	−0.03505236	0.02158588
C	−0.73121826	−1.22897212	−0.50759602
C	1.42055655	0.01668951	−0.50856185
H	1.38251305	0.02701806	−1.59582685
H	1.90060850	0.91846296	−0.13430243
H	1.94623516	−0.86814655	−0.15517575
H	−0.72203530	−1.19116154	−1.59488018
H	−0.22535938	−2.12534346	−0.15435762
H	−1.75199373	−1.19612567	−0.13257711
H	−1.04677701	0.01442696	1.85556874
H	0.50232941	−0.87616622	1.89562293
H	0.50109138	0.91067728	1.85471613
H	−0.46973814	0.80081046	−0.31593141

DABCO

E (TPSSh/def2-TZVP) =	−345.50280686
$G - E$ (TPSSh/def2-TZVP) =	0.15303438
$H - E$ (TPSSh/def2-TZVP) =	0.18964038
E (DLPNO-CCSD(T)tight/def-TZVPP//TPSSh/def2-TZVP) =	−343.17446604
E (DLPNO-CCSD(T)tight/def-QZVPP//TPSSh/def2-TZVP) =	−343.19007775
E (DLPNO-CCSD(T)/CBS//TPSSh/def2-TZVP) =	−344.887661301

C	0.00518432	−0.01301833	−0.05371312
C	0.00468711	−0.01388085	1.50483011
H	1.02299729	−0.00786041	−0.45346874
N	−0.68536748	1.18075844	2.01009072
H	−0.50851818	−0.89298775	1.90428947
H	1.02224501	−0.00915948	1.90523999
C	−2.06499147	1.18103873	1.50483563
C	−2.06449208	1.18190245	−0.05371979
H	−2.56969650	0.29701896	1.90428345
H	−2.56969322	2.06461754	1.90526214
N	−0.68454436	1.18218246	−0.55809067
H	−2.56893762	0.29832385	−0.45446947
H	−2.56894059	2.06592260	−0.45349081
C	0.00468552	2.37595839	1.50615557
H	1.02224290	2.37079701	1.90656132
C	0.00518519	2.37682219	−0.05238800
H	−0.50852358	3.25462073	1.90658698
H	−0.50776711	3.25592825	−0.45217407
H	1.02299914	2.37210443	−0.45214660
H	−0.50777129	−0.89167924	−0.45447210

$\mathbf{HDABCO^+}$

E (TPSSh/def2-TZVP) =	−345.88875235
$G - E$ (TPSSh/def2-TZVP) =	0.16912852
$H - E$ (TPSSh/def2-TZVP) =	0.20442991
E (DLPNO-CCSD(T)tight/def-TZVPP//TPSSh/def2-TZVP) =	−343.56723517
E (DLPNO-CCSD(T)tight/def-QZVPP//TPSSh/def2-TZVP) =	−343.58253793
E (DLPNO-CCSD(T)/CBS//TPSSh/def2-TZVP) =	−345.268968589

C	0.00755167	2.38051674	−0.05709340
N	−0.68459039	1.18215893	−0.52632502
C	0.00755489	−0.01660612	−0.05814174
C	0.03288297	−0.06044612	1.49465307
N	−0.68392854	1.18106164	1.98022088
C	0.03288767	2.42299246	1.49573784
C	−2.06845337	1.18195647	−0.05706621
C	−2.11787499	1.18126946	1.49577558
H	1.02343980	−0.01116949	−0.45231369
H	−0.49907604	−0.91492531	1.91203942
H	1.03886894	−0.02691321	1.91202033
H	−2.59154066	0.29312851	1.91312261
H	−2.59155450	2.06903218	1.91391082
H	−2.57203499	0.29965251	−0.45123780
H	−2.57202408	2.06461481	−0.45045751
H	1.03887518	2.38908178	1.91307115
H	−0.49906108	3.27710787	1.91388116
H	−0.50503739	3.25796444	−0.45049400
H	1.02343431	2.37543446	−0.45127591
H	−0.50502427	−0.89371296	−0.45231462
H	−0.68363914	1.18061994	3.00024504

DBU

E (TPSSh/def2-TZVP) =	−462.32145461
$G - E$ (TPSSh/def2-TZVP) =	0.21116982
$H - E$ (TPSSh/def2-TZVP) =	0.25560988
E (DLPNO-CCSD(T)tight/def-TZVPP//TPSSh/def2-TZVP) =	−459.17841905
E (DLPNO-CCSD(T)tight/def-QZVPP//TPSSh/def2-TZVP) =	−459.19892646
E (DLPNO-CCSD(T)/CBS//TPSSh/def2-TZVP) =	−461.479779049

C	0.14863238	0.55990958	−0.02753047
N	0.43679929	1.12906407	1.08922655
C	1.27942013	0.01341937	−0.86495373
C	−0.68044067	1.63378432	1.87458463
C	−1.92292211	0.75776584	1.73304861
H	−0.36519719	1.68794883	2.91969489
H	−0.91574874	2.66120025	1.56205362
C	−2.30152225	0.66711281	0.26270543
H	−1.69615100	−0.24157281	2.11721583
H	−2.76435492	1.15360454	2.30776888
N	−1.11787768	0.40447079	−0.55682774
H	−3.02034848	−0.14274886	0.10109912
H	−2.77938884	1.59813740	−0.06778679
C	−1.32078110	−0.35750652	−1.78165828
H	1.23342163	0.45775355	−1.86353451
H	2.19607261	0.37051404	−0.39937830
C	1.30202727	−1.53722686	−0.97134313
C	−1.18416223	−1.87603925	−1.58303276
H	−2.31311572	−0.10795498	−2.16541363
H	−0.60555105	−0.01710605	−2.53188648
H	−2.11243988	−2.28532143	−1.17098042
H	−1.04382285	−2.34186860	−2.56469583
C	−0.02537593	−2.23175169	−0.64793950
H	0.12552264	−3.31529669	−0.66096629
H	−0.30676573	−1.97408280	0.37796269
H	1.63735876	−1.82671399	−1.97254799
H	2.05097566	−1.91614788	−0.27164740

$HDBU^+$

E (TPSSh/def2-TZVP) =	−462.74011051
$G - E$ (TPSSh/def2-TZVP) =	0.22597847
$H - E$ (TPSSh/def2-TZVP) =	0.27061648
E (DLPNO-CCSD(T)tight/def-TZVPP//TPSSh/def2-TZVP) =	−459.60578452
E (DLPNO-CCSD(T)tight/def-QZVPP//TPSSh/def2-TZVP) =	−459.62612683
E (DLPNO-CCSD(T)/CBS//TPSSh/def2-TZVP) =	−461.892520487

N	−1.11702607	0.33074425	−0.52034829
C	0.11469655	0.51537164	−0.07886598
N	0.34855401	1.14305256	1.06770132
C	−0.70190888	1.65738985	1.95244090
C	−1.95260256	0.81612834	1.74868764
C	−2.29452088	0.74152725	0.26824005
C	1.27415261	0.00649583	−0.88117302
C	1.33017528	−1.54827806	−0.97947113
C	0.01594401	−2.27193960	−0.67001301
C	−1.15528588	−1.92356597	−1.59302649
C	−1.34315137	−0.41203495	−1.77563364
H	−0.33816709	1.59000940	2.97724064
H	−0.88618273	2.71000332	1.72100005
H	−1.78582798	−0.18964367	2.14152054
H	−2.78854517	1.25434998	2.29395840
H	−3.07524756	0.00227564	0.08890805
H	−2.64671382	1.70482839	−0.11132122
H	1.20372095	0.45000708	−1.87695860
H	2.19645774	0.38858809	−0.44171823
H	−2.35240247	−0.16967223	−2.10615831
H	−0.65878492	−0.02058462	−2.52808633
H	−2.06940036	−2.36749233	−1.19103444
H	−1.00435113	−2.35735931	−2.58500863
H	0.19392812	−3.34800008	−0.71725236
H	−0.26820059	−2.06416030	0.36754669
H	1.68023635	−1.81042565	−1.97990356
H	2.08687327	−1.90467208	−0.27868909
H	1.31377758	1.26519320	1.33442604

5.1.4 Anions

chloride

E (TPSSh/def2-TZVP) =	−460.28391405
$G-E$ (TPSSh/def2-TZVP) =	−0.01504214
$H-E$ (TPSSh/def2-TZVP) =	0.00236048
E (DLPNO-CCSD(T)tight/def-TZVPP//TPSSh/def2-TZVP) =	−459.55562242
E (DLPNO-CCSD(T)tight/def-QZVPP//TPSSh/def2-TZVP) =	−459.57468507
E (DLPNO-CCSD(T)/CBS//TPSSh/def2-TZVP) =	−459.844800829

bromide

E (TPSSh/def2-TZVP) =	−2574.14431341
$G-E$ (TPSSh/def2-TZVP) =	−0.01619296
$H-E$ (TPSSh/def2-TZVP) =	0.00236048
E (DLPNO-CCSD(T)tight/def-TZVPP//TPSSh/def2-TZVP) =	−2572.48103044
E (DLPNO-CCSD(T)tight/def-QZVPP//TPSSh/def2-TZVP) =	−2572.53359332
E (DLPNO-CCSD(T)/CBS//TPSSh/def2-TZVP) =	−2573.36165302

iodide

E (TPSSh/def2-TZVP) =	−297.70544935
$G-E$ (TPSSh/def2-TZVP) =	−0.01684816
$H-E$ (TPSSh/def2-TZVP) =	0.00236048
E (DLPNO-CCSD(T)tight/def-TZVPP//TPSSh/def2-TZVP) =	−254.98991620 − 296.74067393
E (DLPNO-CCSD(T)tight/def-QZVPP//TPSSh/def2-TZVP) =	−257.95530345 − 296.74299444
E (DLPNO-CCSD(T)/CBS//TPSSh/def2-TZVP) =	377.322912857

tetrafluoroborate

E (TPSSh/def2-TZVP) =	−424.70820622
$G-E$ (TPSSh/def2-TZVP) =	−0.01252368
$H-E$ (TPSSh/def2-TZVP) =	0.01945441
E (DLPNO-CCSD(T)tight/def-TZVPP//TPSSh/def2-TZVP) =	−422.92844718
E (DLPNO-CCSD(T)tight/def-QZVPP//TPSSh/def2-TZVP) =	−422.94705095
E (DLPNO-CCSD(T)/CBS//TPSSh/def2-TZVP) =	−424.277177085

F	−1.90272569	1.80492636	−0.85146723
B	−1.00786895	2.89381236	−0.93378683
F	−1.73376025	4.08417694	−1.15622342
F	−0.28594048	3.00106758	0.27480701
F	−0.10890519	2.68505289	−2.00225625

triflate

E (TPSSh/def2-TZVP) =	−961.85765140
$G - E$ (TPSSh/def2-TZVP) =	−0.00402448
$H - E$ (TPSSh/def2-TZVP) =	0.03513627
E (DLPNO-CCSD(T)tight/def-TZVPP//TPSSh/def2-TZVP) =	−958.61257669
E (DLPNO-CCSD(T)tight/def-QZVPP//TPSSh/def2-TZVP) =	−958.65249530
E (DLPNO-CCSD(T)/CBS//TPSSh/def2-TZVP) =	−960.789127651

O	−2.29210856	1.62198091	−0.36864665
S	−1.85353152	2.98045913	−0.08596083
O	−0.48834515	3.11344390	0.39986257
C	−1.73795928	3.72252492	−1.79557534
O	−2.83706496	3.83939147	0.55636054
F	−1.33691416	5.01598648	−1.76811551
F	−0.85605022	3.06279058	−2.58407851
F	−2.92592267	3.70168744	−2.44574563

5.1.5 Catalysts

Catalyst 1

E (TPSSh/def2-TZVP) =	−458.67475685
$G - E$ (TPSSh/def2-TZVP) =	0.13988252
$H - E$ (TPSSh/def2-TZVP) =	0.18342573
E (DLPNO-CCSD(T)/def-TZVPP//TPSSh/def2-TZVP) =	−455.66665476
E (DLPNO-CCSD(T)/def-QZVPP//TPSSh/def2-TZVP) =	−455.68582109
E (DLPNO-CCSD(T)tight/def-QZVPP//TPSSh/def2-TZVP) =	
E (DLPNO-CCSD(T)/CBS//TPSSh/def2-TZVP) =	−457.829457242

C	0.90992074	2.61367340	−0.90965033
C	2.01624209	2.35338091	−1.71413285
C	2.51706104	1.05678336	−1.70490996
C	1.93326861	0.05353772	−0.91908531
C	0.82886847	0.31294133	−0.11592436
C	0.32684839	1.61171446	−0.12485543
N	2.68460478	−1.09071801	−1.15886251
C	3.70654148	−0.88069883	−2.04464631
N	3.57793570	0.44452729	−2.36136827
C	4.46447023	1.12221739	−3.28656851
C	2.42763520	−2.37832406	−0.54467832
H	1.42763840	−2.73493393	−0.80496880
H	2.50804997	−2.30745859	0.54316689
H	3.17442855	−3.07172198	−0.92340193
H	3.90228196	1.51846850	−4.13619114
H	4.98287514	1.94540161	−2.78805684
H	5.18856609	0.39007214	−3.63523773
H	0.37448063	−0.45915107	0.49289398
H	−0.53331191	1.85252545	0.48843571
H	2.46413963	3.13175979	−2.31975402
H	0.49101379	3.61274114	−0.89027394

protonated Catalyst 1

E (TPSSh/def2-TZVP) =	−459.10711566
$G-E$ (TPSSh/def2-TZVP) =	0.15368064
$H-E$ (TPSSh/def2-TZVP) =	0.19761212
E (DLPNO-CCSD(T)/def-TZVPP//TPSSh/def2-TZVP) =	−456.09628852
E (DLPNO-CCSD(T)/def-QZVPP//TPSSh/def2-TZVP) =	−456.11552593
E (DLPNO-CCSD(T)tight/def-QZVPP//TPSSh/def2-TZVP) =	
E (DLPNO-CCSD(T)/CBS//TPSSh/def2-TZVP) =	−458.254496302

N	3.59060782	0.47278726	−2.37631862
C	2.51891325	1.06317013	−1.70638027
C	1.93741904	0.05747378	−0.92155952
N	2.68057745	−1.10108540	−1.14810702
C	3.65085578	−0.81070736	−2.01674983
C	0.83113220	0.30319589	−0.11443293
C	0.33672744	1.59800393	−0.13016989
C	0.91989044	2.60651687	−0.91720703
C	2.02198585	2.36260226	−1.72161461
C	2.43734980	−2.40935880	−0.53783350
C	4.49298737	1.14605573	−3.31205584
H	1.43941670	−2.75286963	−0.80836064
H	2.52189100	−2.32280148	0.54494699
H	3.17989403	−3.11212095	−0.90845703
H	3.91719969	1.53256228	−4.15237770
H	4.99947918	1.96291067	−2.79896282
H	5.22634200	0.42722288	−3.66993699
H	0.37812758	−0.46889316	0.49330565
H	−0.52316721	1.84100812	0.48070720
H	2.46663290	3.14269778	−2.32528704
H	0.49496761	3.60176203	−0.89337300
H	4.38216209	−1.51545684	−2.37802457

Catalyst 2

E (TPSSh/def2-TZVP) =	−762.18165045
$G - E$ (TPSSh/def2-TZVP) =	0.09793163
$H - E$ (TPSSh/def2-TZVP) =	0.13869148
E (DLPNO-CCSD(T)/def-TZVPP//TPSSh/def2-TZVP) =	−759.09765996
E (DLPNO-CCSD(T)/def-QZVPP//TPSSh/def2-TZVP) =	−759.12360048
E (DLPNO-CCSD(T)tight/def-QZVPP//TPSSh/def2-TZVP) =	
E (DLPNO-CCSD(T)/CBS//TPSSh/def2-TZVP) =	−761.041545115

C	0.92285604	2.45407056	−1.22794075
C	1.98992960	1.83961488	−1.86913503
C	2.21034911	0.48478323	−1.62521747
C	1.38022401	−0.23739776	−0.75888108
C	0.31128305	0.38653803	−0.11903922
C	0.09141719	1.73570380	−0.36147696
S	1.96318637	−1.88471805	−0.70775747
C	3.26831216	−1.62711680	−1.80072316
N	3.21890002	−0.33072086	−2.15918579
C	4.20158786	0.21784053	−3.09139459
H	3.69998188	0.60223947	−3.98241286
H	4.76066828	1.02618212	−2.61467337
H	4.87292059	−0.59200098	−3.36107278
H	−0.33230779	−0.16942779	0.55153867
H	−0.73464699	2.23915221	0.12645157
H	2.63058122	2.39960761	−2.53886808
H	0.73200042	3.50619182	−1.40203564

protonated Catalyst 2

E (TPSSh/def2-TZVP) =	−762.60153523
$G - E$ (TPSSh/def2-TZVP) =	0.11137101
$H - E$ (TPSSh/def2-TZVP) =	0.15235276
E (DLPNO-CCSD(T)/def-TZVPP//TPSSh/def2-TZVP) =	−759.51357395
E (DLPNO-CCSD(T)/def-QZVPP//TPSSh/def2-TZVP) =	−759.53965233
E (DLPNO-CCSD(T)tight/def-QZVPP//TPSSh/def2-TZVP) =	
E (DLPNO-CCSD(T)/CBS//TPSSh/def2-TZVP) =	−761.453915719

N	3.22574844	−0.30865837	−2.17101933
C	2.21687344	0.49361035	−1.63276931
C	1.39013129	−0.23836399	−0.76592041
S	1.92508170	−1.89077455	−0.67545226
C	3.18072912	−1.56158473	−1.75229655
C	0.31510884	0.36446265	−0.11513271
C	0.09785078	1.71004991	−0.35877114
C	0.92551237	2.44052890	−1.22597274
C	1.99462186	1.84814263	−1.87530525
C	4.23533094	0.20022837	−3.11204708
H	3.73132376	0.57505485	−4.00220151
H	4.79525964	1.00094691	−2.63020732
H	4.90469165	−0.61338334	−3.37837291
H	−0.32401240	−0.19688954	0.55362075
H	−0.72877995	2.20896040	0.13075338
H	2.62912269	2.41624692	−2.54270888
H	0.72353235	3.49100310	−1.39105656
H	3.89379948	−2.30398948	−2.07903216

Catalyst 3

E (TPSSh/def2-TZVP) =	−1032.87752904
$G - E$ (TPSSh/def2-TZVP) =	0.21119058
$H - E$ (TPSSh/def2-TZVP) =	0.26899649
E (DLPNO-CCSD(T)/def-TZVPP//TPSSh/def2-TZVP) =	−1028.04567983
E (DLPNO-CCSD(T)/def-QZVPP//TPSSh/def2-TZVP) =	−1028.08363098
E (DLPNO-CCSD(T)tight/def-QZVPP//TPSSh/def2-TZVP) =	
E (DLPNO-CCSD(T)/CBS//TPSSh/def2-TZVP) =	−1031.27962374

C	1.14428288	0.94300536	−2.04351179
C	1.93826493	0.25699670	−1.12441570
C	1.91969330	0.64614916	0.21397600
C	1.11700645	1.70227044	0.63317396
C	0.32938610	2.38454791	−0.28818466
C	0.34597059	2.00188763	−1.62666324
C	2.79236970	−0.91085228	−1.56332788
N	3.40826677	−0.69833053	−2.87827091
C	4.44768556	0.23300263	−3.05258175
C	4.80836553	0.33178831	−4.35671752
S	3.81541832	−0.76603857	−5.27898102
C	2.91263879	−1.35494940	−3.94967940
C	5.02918577	0.96037825	−1.88575066
C	5.89028705	1.16882433	−4.96095910
C	7.16927197	0.37146527	−5.19516031
O	8.12564676	1.26681825	−5.75891820
H	3.57295162	−1.10515124	−0.82394659
H	2.19052595	−1.81289539	−1.67558452
H	5.48463822	0.26721543	−1.17160336
H	5.80512577	1.64691987	−2.22314037
H	4.26768658	1.53442248	−1.35228558
H	5.56158498	1.58532372	−5.91661749
H	6.12173986	2.01473754	−4.30830325
H	7.52730521	−0.03970521	−4.24212370
H	6.96447168	−0.46708411	−5.87231183
H	8.92695713	0.76971074	−5.96221645
H	2.54243749	0.12209979	0.93242603
H	1.11334195	1.99628013	1.67651806
H	−0.29169065	3.21224153	0.03402686
H	−0.26564964	2.52978091	−2.34933091
H	1.15593942	0.64308036	−3.08521672

protonated Catalyst 3

E (TPSSh/def2-TZVP) =	−1033.30990711
$G - E$ (TPSSh/def2-TZVP) =	0.22411254
$H - E$ (TPSSh/def2-TZVP) =	0.28232716
E (DLPNO-CCSD(T)/def-TZVPP//TPSSh/def2-TZVP) =	−1028.47598842
E (DLPNO-CCSD(T)/def-QZVPP//TPSSh/def2-TZVP) =	−1028.51398187
E (DLPNO-CCSD(T)tight/def-QZVPP//TPSSh/def2-TZVP) =	
E (DLPNO-CCSD(T)/CBS//TPSSh/def2-TZVP) =	−1031.70302402

C	2.82541514	0.73025656	−3.43392365
N	3.75418843	0.86715646	−2.49983557
C	5.06038841	0.87951419	−2.98525028
C	5.09542079	0.75279508	−4.34627425
S	3.48815645	0.61126892	−4.97009702
C	3.43397237	1.00530526	−1.03892134
C	1.95986973	0.96611131	−0.77781522
C	1.21165291	2.14496973	−0.80357612
C	−0.16220429	2.10353957	−0.59588246
C	−0.79329956	0.88506526	−0.36049589
C	−0.05096566	−0.29220595	−0.32699229
C	1.32296915	−0.25257397	−0.53506900
C	6.21352717	1.02331366	−2.05226062
C	6.28089859	0.68900698	−5.25364695
C	6.71575824	−0.74958855	−5.55060210
O	7.84844432	−0.63661079	−6.38713844
H	3.87552376	1.95051051	−0.72094699
H	3.95499062	0.18980407	−0.53601201
H	6.24217117	0.20501327	−1.32839205
H	7.14569698	1.00925582	−2.61282335
H	6.16261418	1.96446020	−1.49893608
H	6.06337031	1.18930453	−6.19986007
H	7.11748173	1.22243921	−4.79860440
H	6.95145984	−1.26874365	−4.61165400
H	5.89703716	−1.29152005	−6.04353182
H	8.15872985	−1.51792005	−6.63082865
H	1.90266636	−1.16927062	−0.49964049
H	−0.53980547	−1.23832119	−0.13006174
H	−1.86295530	0.85420375	−0.19292635
H	−0.73765422	3.02091055	−0.60840293
H	1.70450991	3.09598977	−0.97765408
H	1.77206091	0.70709116	−3.20275279

Catalyst 4

E (TPSSh/def2-TZVP) =	−1000.59878784
$G - E$ (TPSSh/def2-TZVP) =	0.30238750
$H - E$ (TPSSh/def2-TZVP) =	0.36260885
E (DLPNO-CCSD(T)/def-TZVPP//TPSSh/def2-TZVP) =	−995.70023977
E (DLPNO-CCSD(T)/def-QZVPP//TPSSh/def2-TZVP) =	−995.73758222
E (DLPNO-CCSD(T)tight/def-QZVPP//TPSSh/def2-TZVP) =	
E (DLPNO-CCSD(T)/CBS//TPSSh/def2-TZVP) =	−999.013688289

C	1.94810222	2.33187715	−1.48352008
C	1.96283609	1.04336338	−0.65684170
C	0.84308765	1.08106528	0.38785913
C	0.95040572	2.34143327	1.24563107
C	0.86835112	3.62511394	0.40813434
C	2.02345712	3.58010684	−0.60289231
N	0.02546961	2.30071088	2.40667916
C	−1.37387572	2.42288620	2.33444914
C	−1.92924396	2.29618542	3.56302618
S	−0.69245505	2.03902985	4.73872791
C	0.59691331	2.08927732	3.61634089
C	−2.13748282	2.69931103	1.07740111
C	0.82907416	4.90132572	1.27475054
C	0.61384851	6.15043066	0.41687095
C	1.85398723	−0.19805786	−1.53892781
C	2.05320425	5.05874017	2.18028811
H	1.92534541	2.32049101	1.73559616
H	−2.98218497	2.35190943	3.79265169
H	−3.20180915	2.57595085	1.27778373
H	−1.97872354	3.72253550	0.72803423
H	−1.86507467	2.02299709	0.26620823
H	0.89841440	0.20309537	1.03767458
H	−0.12262345	1.04410789	−0.12810904
H	−0.05678175	3.60347474	−0.17942721
H	2.91802163	1.00274913	−0.11630835
H	2.00185952	4.47519774	−1.22916482
H	2.97935036	3.58829651	−0.06770330
H	2.77858763	2.32390546	−2.19665682
H	1.02425340	2.36166094	−2.07638739
H	2.67218076	−0.23936922	−2.26284192
H	1.88405269	−1.11258613	−0.94071438
H	0.91244425	−0.19247692	−2.09740179
H	−0.04498570	4.80451555	1.92959962
H	0.43129173	7.02207891	1.05030175
H	1.48994133	6.37069914	−0.19888236
H	−0.24554231	6.03288957	−0.25072962
H	1.95563545	5.96343646	2.78594614
H	2.15954760	4.21262608	2.86332455
H	2.97187093	5.15333264	1.59442172

protonated Catalyst 4

E (TPSSh/def2-TZVP) =	−1001.03121460
$G - E$ (TPSSh/def2-TZVP) =	0.31557673
$H - E$ (TPSSh/def2-TZVP) =	0.37617170
E (DLPNO-CCSD(T)/def-TZVPP//TPSSh/def2-TZVP) =	−996.13122012
E (DLPNO-CCSD(T)/def-QZVPP//TPSSh/def2-TZVP) =	−996.16872557
E (DLPNO-CCSD(T)tight/def-QZVPP//TPSSh/def2-TZVP) =	
E (DLPNO-CCSD(T)/CBS//TPSSh/def2-TZVP) =	−999.438429026

C	0.43626665	2.06185362	3.61594711
N	−0.01142371	2.28949788	2.38745782
C	−1.39862934	2.42411364	2.32134610
C	−1.95534762	2.28045717	3.55611657
S	−0.78235404	1.99278577	4.76710872
C	0.96749157	2.33181715	1.24281568
C	0.85263455	1.06767035	0.39203154
C	1.97721927	1.03455989	−0.65215858
C	1.96132736	2.32649127	−1.47283570
C	2.04425834	3.56947821	−0.58665632
C	0.88705550	3.62540531	0.42460117
C	1.85883945	−0.20832055	−1.53100866
C	0.85913314	4.90016161	1.29598401
C	2.12864332	5.10387777	2.12747375
C	−2.15544240	2.72064646	1.07077611
C	0.56625898	6.13846182	0.44508277
H	1.93600961	2.31393695	1.74814779
H	−3.00347335	2.33737304	3.80315483
H	−3.21842429	2.58649077	1.26563358
H	−1.99459117	3.75306663	0.75505915
H	−1.86932729	2.06050313	0.25318509
H	0.90350215	0.18307466	1.03390331
H	−0.11154725	1.04219819	−0.12424750
H	−0.03812791	3.60287585	−0.16000756
H	2.93172315	0.98916499	−0.11071032
H	2.02184552	4.46979371	−1.20274960
H	3.00024075	3.57388710	−0.05217043
H	2.79269202	2.32015077	−2.18188764
H	1.04066799	2.36134977	−2.06848947
H	2.67653568	−0.24843900	−2.25317703
H	1.89066330	−1.12302379	−0.93416805
H	0.91824151	−0.19968297	−2.08938200
H	0.01968577	4.79214319	1.99648209
H	0.41285569	7.01087667	1.08306397
H	1.39567300	6.36579218	−0.22786703
H	−0.33335120	6.00206082	−0.16160450
H	2.01746003	5.97618726	2.77410844
H	2.35407984	4.24925013	2.77297787
H	2.99774747	5.27618914	1.48940529
H	1.48435995	1.93122738	3.83421763

Catalyst 5

E (TPSSh/def2-TZVP) =	−783.52602223
$G - E$ (TPSSh/def2-TZVP) =	0.24882293
$H - E$ (TPSSh/def2-TZVP) =	0.30739862
E (DLPNO-CCSD(T)/def-TZVPP//TPSSh/def2-TZVP) =	−778.47637075
E (DLPNO-CCSD(T)/def-QZVPP//TPSSh/def2-TZVP) =	−778.50918813
E (DLPNO-CCSD(T)tight/def-QZVPP//TPSSh/def2-TZVP) =	
E (DLPNO-CCSD(T)/CBS//TPSSh/def2-TZVP) =	−782.137638418

O	−0.76215882	4.01572198	0.57502326
C	−0.11246727	3.60331537	−0.51448283
N	−0.15416099	4.48448548	−1.55234869
C	−0.98073723	5.64310654	−1.22717344
C	−1.15097843	5.40026321	0.30186099
C	0.52085864	3.98664833	−2.63644581
N	0.92465391	2.79987650	−2.11756767
N	0.54556366	2.52629185	−0.78219724
C	1.69776090	1.82415799	−2.79810790
C	2.13099737	2.08144488	−4.09860145
C	2.88356050	1.12479954	−4.76484366
C	3.20802798	−0.08132481	−4.14764126
C	2.77051816	−0.32354989	−2.85052965
C	2.01470999	0.62364927	−2.16807838
C	−2.29802210	5.71226563	−2.03561004
C	−3.09620603	4.41217190	−1.88866679
C	−3.11994537	6.90082513	−1.51881454
C	−1.95478243	5.94863326	−3.51170461
H	−0.41533433	6.56138025	−1.39972408
H	−0.49441518	6.04307690	0.88943859
H	−2.17708670	5.51051595	0.64263062
H	1.66952475	0.44689068	−1.15936750
H	3.01651169	−1.25768903	−2.35877348
H	3.79693278	−0.82326274	−4.67378141
H	3.22035835	1.32583346	−5.77535177
H	1.87056627	3.02415637	−4.56050247
H	−4.01123220	7.02886422	−2.13716101
H	−3.45521875	6.75938101	−0.48773477
H	−2.54334212	7.82949278	−1.56772170
H	−2.87257719	5.98643551	−4.10421149
H	−1.42837406	6.89922278	−3.63974340
H	−1.31320807	5.15546862	−3.90009910
H	−4.03573534	4.49161632	−2.44028692
H	−2.54206316	3.56204771	−2.29188475
H	−3.34192416	4.19796905	−0.84457165

protonated Catalyst 5

E (TPSSh/def2-TZVP) =	−783.94841438
$G - E$ (TPSSh/def2-TZVP) =	0.26232010
$H - E$ (TPSSh/def2-TZVP) =	0.32097754
E (DLPNO-CCSD(T)/def-TZVPP//TPSSh/def2-TZVP) =	−778.89430287
E (DLPNO-CCSD(T)/def-QZVPP//TPSSh/def2-TZVP) =	−778.92736056
E (DLPNO-CCSD(T)tight/def-QZVPP//TPSSh/def2-TZVP) =	
E (DLPNO-CCSD(T)/CBS//TPSSh/def2-TZVP) =	−782.551433706

C	1.67351430	0.50692948	−2.42251601
C	1.76802176	1.83022463	−2.83656595
C	2.58320073	2.22019843	−3.89441655
C	3.30381369	1.24547514	−4.57169625
C	3.21700519	−0.08764971	−4.18008022
C	2.41008173	−0.45189214	−3.10577179
N	0.99251575	2.81854843	−2.14960355
C	0.50240362	3.95177391	−2.64250362
N	−0.12855785	4.55978573	−1.62296778
C	0.00224132	3.72901091	−0.55023003
N	0.68216749	2.64658759	−0.81523729
C	−0.99885528	5.71249427	−1.30251629
C	−1.02986718	5.53911879	0.24696850
O	−0.57959813	4.16712788	0.54223997
C	−2.37176794	5.66426157	−2.01075976
C	−2.15951919	5.74705564	−3.52783291
C	−3.13566753	4.38260900	−1.66010909
C	−3.15635542	6.90205322	−1.54887182
H	−0.48031617	6.63370143	−1.57123665
H	−0.33927305	6.21307753	0.75147475
H	−2.02659683	5.63444037	0.66523305
H	1.04001678	0.24373981	−1.58652137
H	2.34899326	−1.48778053	−2.79722756
H	3.78827080	−0.84177473	−4.70666247
H	3.94809511	1.53291470	−5.39281830
H	2.68569938	3.26386256	−4.16702180
H	−4.12077563	6.93068484	−2.05813588
H	−3.35610927	6.88980406	−0.47484997
H	−2.62195715	7.82488198	−1.78993568
H	−3.12338760	5.84906777	−4.02885004
H	−1.54844759	6.61160366	−3.80266256
H	−1.69289583	4.84102879	−3.92408040
H	−4.10128484	4.38373252	−2.16806028
H	−2.59801835	3.48759208	−1.98568615
H	−3.33093125	4.29628261	−0.58867550
H	0.59634415	4.29778878	−3.65585575

5.2 Reactions: Precursor + Amine + Aldehyde

5.2.1 Imidazolium

I

E (TPSSh/def2-TZVP) =	−479.95840487
$G - E$ (TPSSh/def2-TZVP) =	0.22036480
$H - E$ (TPSSh/def2-TZVP) =	0.27553626
E (DLPNO-CCSD(T)tight/def-TZVPP//TPSSh/def2-TZVP) =	−476.73539535
E (DLPNO-CCSD(T)tight/def-QZVPP//TPSSh/def2-TZVP) =	−476.75706802
E (DLPNO-CCSD(T)/CBS//TPSSh/def2-TZVP) =	−479.092777822

C	2.15573088	1.16839903	−0.99458493
C	1.00147719	0.59015661	−0.57396650
N	0.05716972	1.59469728	−0.48631317
C	0.61632675	2.75631281	−0.84278683
N	1.89411538	2.51507766	−1.15566121
C	−1.33704655	1.41931305	−0.06682044
C	2.85871924	3.52664829	−1.59853836
N	−0.72336864	5.51697837	−0.97064346
C	−0.63941212	6.00411183	−2.35051537
C	−2.12412996	5.37340371	−0.56602317
C	−0.02041558	6.42338980	−0.05810494
H	−1.36176412	1.03142408	0.95054154
H	−2.66540221	6.33015861	−0.59464339
H	3.12597983	0.74585760	−1.19057415
H	0.11806662	3.74459296	−0.87778698
H	−1.83264903	0.72469956	−0.74348795
H	−1.83125401	2.38625447	−0.10447612
H	3.69086189	3.56426204	−0.89703505
H	3.21961613	3.27096118	−2.59370476
H	2.35583425	4.48969755	−1.62525354
H	0.77236171	−0.43334273	−0.33251904
H	−2.63094623	4.67791118	−1.23924863
H	−2.17322733	4.98673544	0.45464788
H	−0.45992458	7.43130508	−0.05395427
H	−0.06151239	6.02536109	0.95773964
H	1.02579893	6.51092752	−0.35955982
H	−1.11439168	6.98816966	−2.47302122
H	0.40834730	6.09370347	−2.64588209
H	−1.13396339	5.29711578	−3.01965170

II(formaldehyde)

E (TPSSh/def2-TZVP) =	−419.50984499
$G - E$ (TPSSh/def2-TZVP) =	0.11977969
$H - E$ (TPSSh/def2-TZVP) =	0.16601427
E (DLPNO-CCSD(T)tight/def-TZVPP//TPSSh/def2-TZVP) =	−416.86828192
E (DLPNO-CCSD(T)tight/def-QZVPP//TPSSh/def2-TZVP) =	−416.88699278
E (DLPNO-CCSD(T)/CBS//TPSSh/def2-TZVP) =	−418.796070804

C	−0.13732981	0.33910839	0.17139558
N	−0.15391881	0.30987174	1.53162917
C	1.09172249	0.03771308	2.07275574
C	1.93515999	−0.11586096	1.02502668
N	1.16849958	0.07130100	−0.11434069
C	−1.35015978	0.56725595	2.32276937
C	1.68285488	−0.01227454	−1.47002230
C	−2.44038719	−0.00992173	−1.01835096
H	−1.86573969	−0.76150496	−1.58896624
O	−3.26989754	−0.32782686	−0.19047902
H	−2.20498786	0.57768683	1.65213753
H	−1.48072765	−0.22445078	3.06243739
H	−1.26375414	1.52836300	2.83435754
H	0.85972816	0.20231090	−2.14715303
H	2.47913394	0.71952647	−1.62212780
H	2.07122980	−1.01291413	−1.67219712
H	1.26817016	−0.02280633	3.13358979
H	2.98850301	−0.33869878	0.99458966
H	−2.28620555	1.03569171	−1.33128930

TS$_{\text{II-III}}$(formaldehyde)

E (TPSSh/def2-TZVP) =	−419.50759437
$G - E$ (TPSSh/def2-TZVP) =	0.12130257
$H - E$ (TPSSh/def2-TZVP) =	0.16654989
E (DLPNO-CCSD(T)tight/def-TZVPP//TPSSh/def2-TZVP) =	−416.84898454
E (DLPNO-CCSD(T)tight/def-QZVPP//TPSSh/def2-TZVP) =	−416.86781745
E (DLPNO-CCSD(T)/CBS//TPSSh/def2-TZVP) =	−418.789406469

N	0.06550798	0.22090103	0.00572163
C	−0.01776521	0.37065772	1.37814031
C	1.22375991	0.14182328	1.86927377
N	2.02463818	−0.14263850	0.77482574
C	1.31872231	−0.10121862	−0.38270757
C	3.44033812	−0.46756239	0.84480103
C	−1.05128995	0.36840352	−0.92567586
C	1.91926223	0.34225674	−2.25226030
H	1.91163849	−0.70228305	−2.61593496
O	1.15045513	1.20000537	−2.73274242
H	−0.63414610	0.66971468	−1.88891434
H	−1.58881192	−0.57743325	−1.01349192
H	−1.72524433	1.13683627	−0.54740640
H	3.82475511	−0.51994289	−0.17078803
H	3.97543189	0.30713781	1.39611215
H	3.58603794	−1.43065708	1.33767590
H	−0.93721795	0.62596733	1.87676130
H	1.59958238	0.16050521	2.87808342
H	2.92726978	0.64242581	−1.89269242

III(formaldehyde)

E (TPSSh/def2-TZVP) =	−419.51670527
$G - E$ (TPSSh/def2-TZVP) =	0.12288015
$H - E$ (TPSSh/def2-TZVP) =	0.16698954
E (DLPNO-CCSD(T)tight/def-TZVPP//TPSSh/def2-TZVP) =	−416.86198937
E (DLPNO-CCSD(T)tight/def-QZVPP//TPSSh/def2-TZVP) =	−416.88131413
E (DLPNO-CCSD(T)/CBS//TPSSh/def2-TZVP) =	−418.803177505

C	1.25150020	−0.00709854	−0.36833663
N	−0.03009732	0.03946721	0.02720500
C	−0.09385267	0.05809797	1.41068222
C	1.17993093	0.02296642	1.87046738
N	2.00002272	−0.01950904	0.75802860
C	−1.19184652	0.06802999	−0.87610415
C	3.45562805	−0.04301435	0.75952719
C	1.78751778	−0.04673013	−1.81326382
O	0.88246407	−0.05447323	−2.76010375
H	2.48175636	−0.94306294	−1.77111630
H	−0.76080989	0.02480378	−1.88817611
H	−1.82446537	−0.79302360	−0.65641571
H	−1.74607287	0.99139143	−0.70191564
H	3.80189202	−0.81177972	0.06835804
H	3.84902520	0.92401880	0.44276629
H	3.79883584	−0.27021143	1.76677717
H	−1.03367383	0.09237476	1.93309807
H	1.57342489	0.02031025	2.87208132
H	2.51403640	0.82286336	−1.81099018

2(formaldehyde)

E (TPSSh/def2-TZVP) =	−594.52523250
$G - E$ (TPSSh/def2-TZVP) =	0.24191901
$H - E$ (TPSSh/def2-TZVP) =	0.30901083
E (DLPNO-CCSD(T)tight/def-TZVPP//TPSSh/def2-TZVP) =	−590.66329570
E (DLPNO-CCSD(T)tight/def-QZVPP//TPSSh/def2-TZVP) =	−590.69008755
E (DLPNO-CCSD(T)/CBS//TPSSh/def2-TZVP) =	−593.496841143

C	2.18286887	1.12366492	−0.86560340
N	0.98805304	0.52224269	−0.51997749
C	0.01515194	1.43857162	−0.53531884
N	0.55698546	2.60532798	−0.89190621
C	1.91093004	2.43351832	−1.09666913
C	0.81228526	−0.88922287	−0.17197696
H	1.38166767	−1.11349992	0.72915845
C	−0.15852651	3.88155274	−0.94874878
C	0.64598647	3.15654072	3.32190659
N	−2.93645914	1.03067667	0.28001251
C	−3.19307020	−0.39130535	0.51508385
H	−4.22403590	−0.57949963	0.84975318
H	2.55231889	3.24654048	−1.38880332
H	−1.04296228	1.26908327	−0.27413346
H	1.15761702	−1.51170124	−0.99616418
H	−0.24432933	−1.06995119	0.00600928
H	0.06553208	4.45416695	−0.05019899
H	0.15232556	4.42396732	−1.83955809
H	−1.22462636	3.67681683	−1.00014684
H	3.10499831	0.57235434	−0.92552724
H	−3.02757259	−0.95164188	−0.40798469
H	−2.51424819	−0.76303373	1.28638580
C	−3.10550056	1.79339083	1.51879253
C	−3.82148468	1.54231792	−0.76866779
H	−4.13000476	1.72387454	1.91350742
H	−2.41474467	1.41590476	2.27483910
H	−2.87824070	2.84550396	1.33509646
H	−4.88384891	1.44667770	−0.49962863
H	−3.61077344	2.59980769	−0.94356145
H	−3.64935758	0.99122867	−1.69528367
O	0.44600401	2.86033365	2.16813208
H	0.69260137	4.20924046	3.65267370
H	0.79075676	2.38818976	4.10224123

$\mathbf{TS_{2\text{-}IV}}$(formaldehyde)

E (TPSSh/def2-TZVP) =	−594.49378732
$G - E$ (TPSSh/def2-TZVP) =	0.24813107
$H - E$ (TPSSh/def2-TZVP) =	0.30926874
E (DLPNO-CCSD(T)tight/def-TZVPP//TPSSh/def2-TZVP) =	−590.61835372
E (DLPNO-CCSD(T)tight/def-QZVPP//TPSSh/def2-TZVP) =	−590.64506634
E (DLPNO-CCSD(T)/CBS//TPSSh/def2-TZVP) =	−593.461803832

C	1.99733743	0.99072798	−1.37106970
N	1.16428683	0.59412149	−0.34063207
C	0.31299436	1.58479850	0.04355225
N	0.65388526	2.61577844	−0.77966675
C	1.67271544	2.27580300	−1.65036978
C	1.20984483	−0.72588220	0.27544841
H	2.21151335	−0.92724496	0.65625066
C	0.03455798	3.93455223	−0.72255297
C	−0.06659396	2.30070169	2.39232395
N	−2.86200825	1.35478271	0.68696901
C	−2.75721828	−0.12753918	0.81964291
H	−3.69886675	−0.57576904	0.50642196
H	2.07544371	2.96174615	−2.37631242
H	−1.95962437	1.74461048	1.02457075
H	0.94105858	−1.49470022	−0.45081410
H	0.50316290	−0.73733337	1.10171065
H	0.80190717	4.70147049	−0.61477563
H	−0.53645200	4.12826204	−1.63238829
H	−0.62423328	3.96451828	0.14292199
H	2.73672090	0.34076055	−1.80757940
H	−1.94111396	−0.46691372	0.18642345
H	−2.55305438	−0.36524606	1.86139555
C	−3.93710307	1.90933560	1.56720736
C	−3.04692953	1.75908366	−0.73665917
H	−4.89322571	1.49795508	1.24728754
H	−3.71194601	1.63268899	2.59320892
H	−3.93227746	2.99244616	1.47461843
H	−3.97059274	1.31835169	−1.10877654
H	−3.10517539	2.84426588	−0.78440105
H	−2.19142717	1.40190436	−1.30538433
O	−1.24346048	2.61628118	2.51700267
H	0.70954510	3.04202017	2.14848606
H	0.30495634	1.30875270	2.69168408

IV(formaldehyde)

E (TPSSh/def2-TZVP) =	−594.55165560
$G - E$ (TPSSh/def2-TZVP) =	0.25300679
$H - E$ (TPSSh/def2-TZVP) =	0.30992147
E (DLPNO-CCSD(T)tight/def-TZVPP//TPSSh/def2-TZVP) =	−590.67564090
E (DLPNO-CCSD(T)tight/def-QZVPP//TPSSh/def2-TZVP) =	−590.70208933
E (DLPNO-CCSD(T)/CBS//TPSSh/def2-TZVP) =	−593.523081215

C	1.87515569	0.79505464	−1.34623552
N	1.30133531	0.68520272	−0.09435508
C	0.82450543	1.88586832	0.28424914
N	1.08309592	2.74791089	−0.71266179
C	1.73475041	2.08758175	−1.73290299
C	1.26323433	−0.54810209	0.69920579
H	1.97211576	−0.48583454	1.52393671
C	0.76990461	4.18487642	−0.70154141
C	0.09948686	2.21871019	1.56262716
N	−3.16269088	1.28188385	0.70635429
C	−2.84893687	−0.12312952	0.42945166
H	−3.74272758	−0.69678722	0.14916053
H	2.03423757	2.59072489	−2.63570263
H	−1.84290183	2.20737599	1.07849275
H	1.53550448	−1.37375619	0.04669631
H	0.25729367	−0.70959819	1.08068598
H	1.62854825	4.73806174	−0.32084963
H	0.55509672	4.49362494	−1.72222586
H	−0.10072308	4.33775118	−0.06927626
H	2.32120280	−0.04812054	−1.84393422
H	−2.13334405	−0.18120485	−0.39444335
H	−2.41520989	−0.58173270	1.32059157
C	−4.07756753	1.39225762	1.85335154
C	−3.74704902	1.92043496	−0.48173948
H	−5.03413587	0.89060674	1.65658555
H	−3.61525018	0.93798101	2.73074684
H	−4.26623187	2.44543335	2.06217356
H	−4.68844117	1.43965126	−0.77869994
H	−3.94075605	2.97187486	−0.26742500
H	−3.04262741	1.85312606	−1.31297383
O	−1.10505244	2.87857791	1.31078855
H	0.73708648	2.87860354	2.16095832
H	−0.02794958	1.29363202	2.13666773

II(acetaldehyde)

E (TPSSh/def2-TZVP) =	−458.85501909
$G - E$ (TPSSh/def2-TZVP) =	0.14548043
$H - E$ (TPSSh/def2-TZVP) =	0.19438334
E (DLPNO-CCSD(T)tight/def-TZVPP//TPSSh/def2-TZVP) =	−455.93526312
E (DLPNO-CCSD(T)tight/def-QZVPP//TPSSh/def2-TZVP) =	−455.95576231
E (DLPNO-CCSD(T)/CBS//TPSSh/def2-TZVP) =	−458.068036955

N	0.00566920	0.12057616	0.09607392
C	−0.00769439	0.07973367	1.48062613
C	1.28807086	0.04313272	1.87056025
N	2.03756422	0.06561288	0.70510335
C	1.26756003	0.11299464	−0.42320919
C	3.48827674	0.03280273	0.66913487
C	−1.19557624	0.18622659	−0.72366104
C	1.27198350	−0.32234191	−3.51720550
O	0.12006133	−0.57454634	−3.79768050
H	−0.89768800	0.13289480	−1.76722014
H	−1.85402971	−0.65318489	−0.49080281
H	−1.72849605	1.12158959	−0.53786508
H	3.79147654	0.14247059	−0.36942339
H	3.90393877	0.85377056	1.25755066
H	3.86414132	−0.91500795	1.06221097
H	−0.91649370	0.08080992	2.05906144
H	1.72732430	0.00386813	2.85342311
C	2.37260745	−1.34331670	−3.47829821
H	1.58248080	0.71329368	−3.28771213
H	3.15456717	−1.06344141	−4.19294911
H	2.81638657	−1.33229536	−2.47995397
H	1.98994631	−2.33612609	−3.71234863

TS$_{\text{II-III}}$(acetaldehyde)

E (TPSSh/def2-TZVP) =	−458.84898420
$G-E$ (TPSSh/def2-TZVP) =	0.14796761
$H-E$ (TPSSh/def2-TZVP) =	0.19578878
E (DLPNO-CCSD(T)tight/def-TZVPP//TPSSh/def2-TZVP) =	−455.90437155
E (DLPNO-CCSD(T)tight/def-QZVPP//TPSSh/def2-TZVP) =	−455.92497850
E (DLPNO-CCSD(T)/CBS//TPSSh/def2-TZVP) =	−458.05617666

C	0.06539414	0.23953994	0.00425184
C	−0.02166069	0.38942431	1.34769949
N	1.24534168	0.13357721	1.84721034
C	2.11650105	−0.17431013	0.85487941
N	1.37716746	−0.09954054	−0.27271598
C	1.60186250	0.15656396	3.25700969
C	4.07442571	0.09057095	0.78628927
O	4.34688261	0.66799897	−0.29325806
C	1.91861505	−0.35660865	−1.60566284
H	2.98103614	−0.10272549	−1.57513564
H	1.77588803	−1.40613007	−1.86899495
H	1.39584159	0.27400132	−2.32444370
H	2.68359873	0.07911876	3.33349429
H	1.27241955	1.09295010	3.70937301
H	1.14000615	−0.68278756	3.78043540
H	−0.67796349	0.34727033	−0.76705044
H	−0.85371941	0.65459438	1.97739471
H	4.05816175	0.70024907	1.71989235
C	4.58392771	−1.32305990	1.04576525
H	5.67251998	−1.27736676	1.15610588
H	4.15286477	−1.75419618	1.95444330
H	4.35704598	−1.97070501	0.19710239

III(acetaldehyde)

E (TPSSh/def2-TZVP) =	−458.85598523
$G - E$ (TPSSh/def2-TZVP) =	0.15148698
$H - E$ (TPSSh/def2-TZVP) =	0.19583973
E (DLPNO-CCSD(T)tight/def-TZVPP//TPSSh/def2-TZVP) =	−455.91282891
E (DLPNO-CCSD(T)tight/def-QZVPP//TPSSh/def2-TZVP) =	−455.93374960
E (DLPNO-CCSD(T)/CBS//TPSSh/def2-TZVP) =	−458.067157477

C	−0.01690890	−0.01138340	0.02487765
C	−0.08528123	0.11458308	1.37156356
N	1.21984475	0.13079541	1.83287753
C	2.08138344	0.01385458	0.79465338
N	1.32078626	−0.07037900	−0.31249849
C	1.60458976	0.19518630	3.23627153
C	3.63022797	0.13220612	0.81530295
O	4.08223239	0.72288414	−0.27595589
C	1.85133525	−0.20789375	−1.67419835
H	2.88790145	0.15956335	−1.60688845
H	1.81635223	−1.25726141	−1.97078905
H	1.23309026	0.38900671	−2.34449039
H	1.71475816	−0.80884688	3.64866506
H	2.55464512	0.72127700	3.31500516
H	0.83450463	0.73528775	3.78504054
H	−0.79076103	−0.05320432	−0.72132622
H	−0.92773564	0.20736394	2.03447407
H	3.80697602	0.71467011	1.76354219
C	4.20061203	−1.28810133	1.09068243
H	5.28577692	−1.19405437	1.16062417
H	3.82177718	−1.74080218	2.01728070
H	3.96919197	−1.94819384	0.25045694

2(acetaldehyde)

E (TPSSh/def2-TZVP) =	−633.87650726
$G - E$ (TPSSh/def2-TZVP) =	0.26993403
$H - E$ (TPSSh/def2-TZVP) =	0.33867062
E (DLPNO-CCSD(T)tight/def-TZVPP//TPSSh/def2-TZVP) =	−629.72842680
E (DLPNO-CCSD(T)tight/def-QZVPP//TPSSh/def2-TZVP) =	−629.75694525
E (DLPNO-CCSD(T)/CBS//TPSSh/def2-TZVP) =	−632.771204282

C	1.89931221	2.63150758	−1.29641620
C	2.06436690	1.29452456	−1.46467131
N	0.85360151	0.70223315	−1.16031696
C	−0.02402662	1.64859264	−0.81300758
N	0.59268139	2.82960389	−0.89741957
C	0.57010452	−0.73367618	−1.19541365
C	0.00233311	4.11702941	−0.52465232
C	0.69343091	2.40013605	3.32660803
O	0.33885921	2.27052333	2.17216192
N	−2.84750253	1.08434939	0.28428021
C	−3.84408483	0.60356829	−0.67479585
C	−2.61077590	0.09188984	1.33403578
C	−3.26987976	2.35480363	0.87638187
H	1.17877447	−1.24267462	−0.44904294
H	−3.51092958	−0.09801994	1.93782626
H	2.58553593	3.44925905	−1.43100125
H	−1.06225078	1.47093544	−0.48617331
H	0.79284891	−1.12319979	−2.18766629
H	−0.48342082	−0.87944350	−0.97253400
H	0.29061941	4.35561064	0.49762842
H	0.35613756	4.87934423	−1.21579530
H	−1.07908643	4.03544337	−0.59215791
H	2.91914565	0.72175770	−1.77943578
H	−2.30281730	−0.85391472	0.88213232
H	−1.81422287	0.44618763	1.99057305
H	−4.20452165	2.25632733	1.44869565
H	−2.48966167	2.71942239	1.54765739
H	−3.43486982	3.08993029	0.08517846
H	−4.82044240	0.41750620	−0.20287836
H	−3.97859227	1.34463930	−1.46494721
H	−3.49880647	−0.32939312	−1.12536954
H	1.09851334	3.37296441	3.67044803
C	0.62726359	1.32803309	4.36425415
H	1.62558328	1.17063805	4.78609200
H	−0.00315941	1.66949119	5.19256478
H	0.23887223	0.39709080	3.95435602

$\mathbf{TS_{2\text{-}IV}}$(acetaldehyde)

E (TPSSh/def2-TZVP) =	−633.84269750
$G - E$ (TPSSh/def2-TZVP) =	0.27249594
$H - E$ (TPSSh/def2-TZVP) =	0.33857613
E (DLPNO-CCSD(T)tight/def-TZVPP//TPSSh/def2-TZVP) =	−629.68979433
E (DLPNO-CCSD(T)tight/def-QZVPP//TPSSh/def2-TZVP) =	−629.71825856
E (DLPNO-CCSD(T)/CBS//TPSSh/def2-TZVP) =	−632.736886112

C	2.01062260	2.32389201	−1.70053438
C	2.30861275	1.01832729	−1.50748590
N	1.34039564	0.52436460	−0.65035788
C	0.42319767	1.46889424	−0.28792927
N	0.87234789	2.57453434	−0.95370417
C	1.29793277	−0.85891485	−0.20074174
C	0.21967912	3.87526686	−0.89970975
C	−0.02826105	2.61596727	2.48289905
O	−1.19639576	2.90666046	2.27624905
N	−3.06359016	1.39009261	1.03136753
C	−3.11969932	2.03379049	−0.31603069
C	−2.64492057	−0.03749009	0.91105209
C	−4.34424755	1.54514076	1.78018958
H	2.26638208	−1.15241723	0.20657925
H	−3.38424011	−0.57177764	0.31568900
H	2.50401134	3.07937343	−2.28839521
H	−2.31921318	1.88998883	1.57810588
H	1.04146572	−1.52480244	−1.02678126
H	0.54288039	−0.93778663	0.57787476
H	0.95117182	4.65114570	−0.66980022
H	−0.25287166	4.10722824	−1.85632854
H	−0.53198391	3.84690543	−0.11478158
H	3.11154329	0.41449832	−1.89539287
H	−1.66860679	−0.03939512	0.42781951
H	−2.58559712	−0.47017306	1.90826044
H	−5.13820073	1.04037633	1.23174865
H	−4.22781727	1.10147009	2.76709179
H	−4.56580657	2.60635111	1.87401687
H	−3.89471957	1.54443058	−0.90474009
H	−3.35282896	3.08878367	−0.18692954
H	−2.13910273	1.90800406	−0.77297270
H	0.75389498	3.35571181	2.24215594
C	0.44548376	1.36195184	3.13120901
H	1.27568415	0.94427592	2.55852518
H	0.83934674	1.62253717	4.12141517
H	−0.35397518	0.63134940	3.24362243

IV(acetaldehyde)

E (TPSSh/def2-TZVP) =	−633.88663737
$G - E$ (TPSSh/def2-TZVP) =	0.28086806
$H - E$ (TPSSh/def2-TZVP) =	0.33944951
E (DLPNO-CCSD(T)tight/def-TZVPP//TPSSh/def2-TZVP) =	−629.72099518
E (DLPNO-CCSD(T)tight/def-QZVPP//TPSSh/def2-TZVP) =	−629.74923521
E (DLPNO-CCSD(T)/CBS//TPSSh/def2-TZVP) =	−632.78359065

C	1.27183711	2.67734681	−1.80571190
C	1.39332404	1.32884568	−1.75124748
N	1.11436242	0.94525527	−0.45255317
C	0.82535550	2.03878500	0.28357846
N	0.92179562	3.09789491	−0.54307355
C	1.14371949	−0.45857589	−0.02337729
C	0.68622469	4.49464377	−0.15324412
C	0.37022590	2.20704634	1.72229181
O	−0.94928297	2.69650777	1.70833035
N	−2.91775644	1.03659927	0.89588812
C	−2.88016701	0.87764774	−0.56108281
C	−3.02210567	−0.26677123	1.56055157
C	−4.04449032	1.89903518	1.28749656
H	1.84089852	−0.58716875	0.80013502
H	−3.95184079	−0.78821319	1.29566585
H	1.40416898	3.36679499	−2.62103662
H	−1.60617756	1.97711185	1.41385806
H	1.47548178	−1.04999216	−0.87281013
H	0.14872132	−0.78130093	0.27400634
H	1.55352899	4.87695997	0.38528278
H	0.53607518	5.07435621	−1.06048712
H	−0.20114473	4.53286763	0.47454292
H	1.65280973	0.61036983	−2.50872781
H	−2.18121263	−0.89885169	1.26607370
H	−2.99554749	−0.12660792	2.64189581
H	−5.00763751	1.46539751	0.98751361
H	−4.03688546	2.03154103	2.36963843
H	−3.93312798	2.87533211	0.81438191
H	−3.79586614	0.41094632	−0.94903651
H	−2.76376317	1.85639645	−1.02960319
H	−2.03171868	0.24858831	−0.84005108
H	0.98515155	3.02279267	2.11891960
C	0.55478517	1.00683428	2.64186889
H	1.59723159	0.68324498	2.68700672
H	0.25317974	1.31678360	3.64214721
H	−0.07393775	0.16564325	2.35030003

II(acrolein)

E (TPSSh/def2-TZVP) =	−496.95306274
$G - E$ (TPSSh/def2-TZVP) =	0.15033641
$H - E$ (TPSSh/def2-TZVP) =	0.20263561
E (DLPNO-CCSD(T)tight/def-TZVPP//TPSSh/def2-TZVP) =	−493.78583875
E (DLPNO-CCSD(T)tight/def-QZVPP//TPSSh/def2-TZVP) =	−493.80758888
E (DLPNO-CCSD(T)/CBS//TPSSh/def2-TZVP) =	−496.091819452

C	1.49569177	0.39809139	−0.20729673
N	0.17074334	0.37257454	0.10590770
C	−0.06469129	−0.07746210	1.39429629
C	1.14927771	−0.34950274	1.92855491
N	2.07652528	−0.05453228	0.94113787
C	−0.87476183	0.75897739	−0.83005579
C	3.51252146	−0.20479598	1.09398097
C	1.90534839	0.33167347	−2.98370018
O	1.10116714	1.03543236	−3.56747700
C	1.70168001	−1.11725929	−2.73322855
H	−0.39939229	1.08584285	−1.75070058
H	−1.52505412	−0.09321311	−1.03855091
H	−1.46976821	1.57324633	−0.41164954
H	3.97524672	0.12446346	0.16667069
H	3.87670354	0.41003022	1.91990532
H	3.77184797	−1.24908679	1.28336978
H	−1.05296682	−0.15967302	1.81492874
H	1.42439785	−0.71276512	2.90457900
H	2.88217049	0.73300016	−2.66583498
C	0.67269907	−1.78621429	−3.25363723
H	2.45523759	−1.61497250	−2.13166057
H	0.53841930	−2.84903615	−3.09181401
H	−0.06191507	−1.26704480	−3.85945420

$\mathbf{TS_{II\text{-}III}}$(acrolein)

E (TPSSh/def2-TZVP) =	−496.94765459
$G - E$ (TPSSh/def2-TZVP) =	0.15351449
$H - E$ (TPSSh/def2-TZVP) =	0.20174843
E (DLPNO-CCSD(T)tight/def-TZVPP//TPSSh/def2-TZVP) =	−493.76165714
E (DLPNO-CCSD(T)tight/def-QZVPP//TPSSh/def2-TZVP) =	−493.78347573
E (DLPNO-CCSD(T)/CBS//TPSSh/def2-TZVP) =	−496.083053036

C	1.35325195	−0.08347641	−0.31115881
N	0.09125517	0.19741863	0.07293089
C	0.01435264	0.38518733	1.44033081
C	1.26768603	0.22155765	1.92782267
N	2.07296283	−0.06249745	0.83561777
C	−1.03048025	0.29163241	−0.86027847
C	3.49959460	−0.33825771	0.90543581
C	1.86973333	0.21660665	−2.18698619
O	1.19617315	1.17606274	−2.63066967
C	1.65394891	−1.18828512	−2.68159545
H	−0.63771443	0.69003387	−1.79832595
H	−1.46603009	−0.69564968	−1.01836046
H	−1.77781245	0.96219703	−0.43759499
H	3.88975407	−0.36101692	−0.10922703
H	4.00250647	0.44766624	1.47021538
H	3.67774981	−1.30261413	1.38490989
H	−0.91047521	0.62161691	1.93809360
H	1.65062111	0.28673096	2.93186458
H	2.93826163	0.38801961	−1.92243210
C	0.80660657	−1.47294305	−3.66550801
H	2.25225368	−1.96442570	−2.20720972
H	0.66780515	−2.48357069	−4.03160284
H	0.23609431	−0.67570816	−4.12945470

III(acrolein)

E (TPSSh/def2-TZVP) =	−496.95509821
$G - E$ (TPSSh/def2-TZVP) =	0.15491498
$H - E$ (TPSSh/def2-TZVP) =	0.20312457
E (DLPNO-CCSD(T)tight/def-TZVPP//TPSSh/def2-TZVP) =	−493.76991037
E (DLPNO-CCSD(T)tight/def-QZVPP//TPSSh/def2-TZVP) =	−493.79202605
E (DLPNO-CCSD(T)/CBS//TPSSh/def2-TZVP) =	−496.094235174

C	1.38147104	−0.08654291	−0.37108336
N	0.09875231	0.11144996	−0.00262564
C	0.02966931	0.35406377	1.35273545
C	1.29385467	0.29611879	1.83644584
N	2.11903594	0.02015630	0.75689116
C	−1.00235406	0.22253592	−0.95847566
C	3.56936737	−0.11181908	0.82688129
C	1.87745643	−0.08963819	−1.82346002
O	1.46879561	1.06324036	−2.34234142
C	1.41924160	−1.38005530	−2.50821407
H	−0.59571652	0.76993599	−1.81477823
H	−1.33247614	−0.76845735	−1.26800530
H	−1.81720666	0.76032715	−0.47632574
H	4.02787170	0.50926795	0.05908524
H	3.89178669	0.22628791	1.81014785
H	3.86926212	−1.15014477	0.68372671
H	−0.90551163	0.55177124	1.84631784
H	1.67994858	0.42720804	2.83176625
H	2.99122758	−0.20746197	−1.73349882
C	0.71507225	−1.34556706	−3.63269409
H	1.73297086	−2.32422817	−2.06073225
H	0.41415311	−2.24488791	−4.15808971
H	0.44840183	−0.37876267	−4.04830632

2(acrolein)

E (TPSSh/def2-TZVP) =	−671.97385003
$G-E$ (TPSSh/def2-TZVP) =	0.27469681
$H-E$ (TPSSh/def2-TZVP) =	0.34512298
E (DLPNO-CCSD(T)tight/def-TZVPP//TPSSh/def2-TZVP) =	−667.58384553
E (DLPNO-CCSD(T)tight/def-QZVPP//TPSSh/def2-TZVP) =	−667.61361643
E (DLPNO-CCSD(T)/CBS//TPSSh/def2-TZVP) =	−670.794369386

N	2.53744410	3.38300145	−1.76575979
C	3.19473109	2.20685482	−1.46298397
C	2.25042390	1.23554118	−1.37785525
N	1.03311328	1.84035201	−1.62791084
C	1.22772249	3.14312859	−1.85496448
C	−0.26981579	1.17353051	−1.63508082
C	3.16434338	4.70051844	−1.88667235
C	1.16773363	5.28242838	1.78145633
C	0.18282179	5.04235010	2.84923734
O	1.19460146	4.69347474	0.71267350
N	−1.07126361	5.15893964	−2.29440414
C	−1.78859957	4.97727913	−3.55779721
C	−1.97296649	4.99009550	−1.15370271
C	−0.43514436	6.47637151	−2.24587031
H	−0.47140512	0.75964354	−0.64780457
H	−1.16907882	7.29516288	−2.28631867
H	4.26294427	2.16447976	−1.34085931
H	0.43300917	3.88045089	−2.05360696
H	−0.26798279	0.37788596	−2.37882145
H	−1.02806842	1.90996462	−1.88727914
H	4.02215514	4.62670095	−2.55297584
H	2.43480368	5.39184354	−2.29835230
H	3.47041685	5.04120000	−0.89944794
H	2.33563964	0.18279902	−1.17226430
H	0.13579627	6.56885371	−1.31971294
H	0.24115453	6.58732143	−3.09687392
H	−2.59229356	5.71681408	−3.69116869
H	−1.09072550	5.07265934	−4.39191027
H	−2.23409113	3.98040143	−3.58641667
H	−2.77745921	5.74084349	−1.14493961
H	−2.43568864	4.00101733	−1.19481448
H	−1.40355585	5.07545995	−0.22703803
H	1.92137149	6.06237358	1.99689487
C	−0.74354617	4.08552206	2.75656802
H	0.25124974	5.67687030	3.72650162
H	−1.46420862	3.90853460	3.54504737
H	−0.79163526	3.45167054	1.87802091

$\mathbf{TS_{2\text{-}IV}}$(acrolein)

E (TPSSh/def2-TZVP) =	−671.94154280
$G - E$ (TPSSh/def2-TZVP) =	0.28004481
$H - E$ (TPSSh/def2-TZVP) =	0.34582193
E (DLPNO-CCSD(T)tight/def-TZVPP//TPSSh/def2-TZVP) =	−667.54198869
E (DLPNO-CCSD(T)tight/def-QZVPP//TPSSh/def2-TZVP) =	−667.57171824
E (DLPNO-CCSD(T)/CBS//TPSSh/def2-TZVP) =	−670.762594397

N	2.40540582	3.12652334	−1.77350841
C	2.94864233	1.85859380	−1.87760659
C	2.05011140	1.01516044	−1.31640946
N	0.99443349	1.80136608	−0.89049205
C	1.18256159	3.12345435	−1.16702735
C	−0.19158576	1.27650060	−0.22845278
C	3.06835639	4.33496739	−2.24473212
C	1.20540354	5.09157237	0.71053192
C	0.55533951	4.24492496	1.72191724
O	0.66194901	6.03966312	0.14516464
N	−1.04894269	5.58087722	−1.89653161
C	−0.40550029	5.44509759	−3.23530578
C	−2.04331599	4.49384647	−1.66636876
C	−1.65037933	6.93486567	−1.70600008
H	0.09916510	0.62261210	0.59441914
H	−2.42854351	7.08298009	−2.45314187
H	3.90778957	1.66702069	−2.32827258
H	−0.30659992	5.49092909	−1.16782252
H	−0.80635973	0.71164106	−0.93189010
H	−0.75566429	2.11836591	0.16521899
H	4.07700067	4.39343779	−1.83408483
H	3.12792878	4.33957569	−3.33476133
H	2.49151651	5.19193890	−1.90388652
H	2.07431604	−0.05354123	−1.18576578
H	−2.06629030	6.98841046	−0.70289938
H	−0.86463607	7.67843199	−1.81597691
H	−1.17240216	5.53792213	−4.00329260
H	0.33428661	6.23508474	−3.34617108
H	0.07248075	4.46902791	−3.27526614
H	−2.84506061	4.59040563	−2.39741593
H	−1.52413645	3.54541539	−1.77583485
H	−2.43730598	4.59335348	−0.65721606
H	2.27846638	4.90212521	0.55528763
C	−0.58417301	4.60499110	2.31633513
H	1.09142029	3.34998207	2.01914059
H	−1.03242033	4.00980678	3.10214353
H	−1.07727837	5.53219560	2.04565466

IV(acrolein)

E (TPSSh/def2-TZVP) =	−671.98316359
$G - E$ (TPSSh/def2-TZVP) =	0.28363980
$H - E$ (TPSSh/def2-TZVP) =	0.34684302
E (DLPNO-CCSD(T)tight/def-TZVPP//TPSSh/def2-TZVP) =	−667.57497626
E (DLPNO-CCSD(T)tight/def-QZVPP//TPSSh/def2-TZVP) =	−667.60448409
E (DLPNO-CCSD(T)/CBS//TPSSh/def2-TZVP) =	−670.808951716

N	2.63469556	3.30726420	−1.47190383
C	2.60373650	2.16662986	−2.24162133
C	1.57666188	1.40960902	−1.78335387
N	0.99084108	2.10344179	−0.74142569
C	1.65032649	3.26400032	−0.55335843
C	−0.14345902	1.57002184	0.02617761
C	3.59284645	4.40980733	−1.62910061
C	1.39153837	4.42700070	0.38880081
C	0.50476954	4.10564871	1.56567282
O	1.00302259	5.53973405	−0.36660553
N	−1.17390550	5.35992703	−1.93053129
C	−0.59795717	5.23775896	−3.27550768
C	−2.18570241	4.32321856	−1.70691500
C	−1.75955994	6.69652071	−1.74230237
H	0.22446443	1.00776769	0.88397736
H	−2.56071985	6.89159372	−2.46732245
H	3.30476109	1.99794812	−3.04032370
H	0.13967487	5.36290023	−0.88200903
H	−0.70488353	0.90970538	−0.63111118
H	−0.77360286	2.38668900	0.36090282
H	4.00975331	4.35194132	−2.63156177
H	3.06231927	5.34964292	−1.49382576
H	4.39164961	4.30981859	−0.89398308
H	1.20818830	0.44977175	−2.10050412
H	−2.17276116	6.77159740	−0.73573672
H	−0.98210798	7.45181978	−1.86039302
H	−1.35759291	5.35912176	−4.05930747
H	0.16911035	6.00143151	−3.41024699
H	−0.14016707	4.25201484	−3.38603842
H	−3.04120364	4.43283816	−2.38717028
H	−1.74119283	3.34043002	−1.87559991
H	−2.54788998	4.38149618	−0.67928888
H	2.37921728	4.67429180	0.80412778
C	−0.53137353	4.85395034	1.92053513
H	0.81423277	3.26127347	2.17699220
H	−1.10565860	4.63086369	2.81078133
H	−0.81405676	5.72722029	1.34564755

II(benzaldehyde)

E (TPSSh/def2-TZVP) =	−650.69056771
$G - E$ (TPSSh/def2-TZVP) =	0.19592585
$H - E$ (TPSSh/def2-TZVP) =	0.24994480
E (DLPNO-CCSD(T)tight/def-TZVPP//TPSSh/def2-TZVP) =	−646.51429994
E (DLPNO-CCSD(T)tight/def-QZVPP//TPSSh/def2-TZVP) =	−646.54181300
E (DLPNO-CCSD(T)/CBS//TPSSh/def2-TZVP) =	−649.526862364

C	0.68699882	0.80011098	−0.80964319
C	0.43180518	0.03710755	0.33387756
C	1.37982596	−0.02295359	1.35800198
C	2.57694807	0.67370764	1.24391921
C	2.82569345	1.43171226	0.10328274
C	1.88117907	1.49479867	−0.92289348
C	−0.82853210	−0.71756285	0.49385941
O	−1.72301168	−0.73519885	−0.33482850
C	−1.76270003	−2.65685299	3.27847594
N	−1.54755122	−3.18146598	4.52139971
C	−2.60012634	−3.96486340	4.96711310
C	−3.52438297	−3.94429385	3.97822346
N	−2.99806950	−3.15013395	2.97245214
C	−0.33640476	−2.93232084	5.28138977
C	−3.68522554	−2.87446007	1.71849714
H	−3.07103165	−2.20322599	1.12303176
H	−3.84736647	−3.80268865	1.16606692
H	−4.65037932	−2.40299060	1.91590951
H	0.29730595	−2.28626190	4.67857748
H	−0.57002233	−2.43609105	6.22624727
H	0.18776357	−3.86830386	5.48823981
H	−4.48998178	−4.41686010	3.90859277
H	−2.60426222	−4.45757384	5.92512914
H	−0.92051297	−1.28471393	1.44133132
H	1.16382118	−0.62030725	2.23800486
H	3.31387961	0.62847902	2.03747097
H	3.75847500	1.97665622	0.01074326
H	2.08473165	2.08802146	−1.80701902
H	−0.06420462	0.83088970	−1.59023905

TS$_{\text{II-III}}$(benzaldehyde)

E (TPSSh/def2-TZVP) =	−650.68305137
$G - E$ (TPSSh/def2-TZVP) =	0.19957342
$H - E$ (TPSSh/def2-TZVP) =	0.24989381
E (DLPNO-CCSD(T)tight/def-TZVPP//TPSSh/def2-TZVP) =	−646.47883654
E (DLPNO-CCSD(T)tight/def-QZVPP//TPSSh/def2-TZVP) =	−646.50644558
E (DLPNO-CCSD(T)/CBS//TPSSh/def2-TZVP) =	−649.514550575

C	−0.03852755	0.18160753	0.42328203
C	0.07159156	0.92230268	1.55151843
N	1.41138745	0.88839647	1.90768530
C	2.13072752	0.14123394	1.03783557
N	1.23122008	−0.28012567	0.12634545
C	1.96841744	1.53150099	3.08789507
C	4.07249946	−0.02252776	0.67342039
O	4.25834614	0.14183813	−0.55419281
C	1.57341815	−1.12960898	−1.01368580
H	2.60316648	−0.90011199	−1.29240512
H	1.48017505	−2.18003859	−0.73447833
H	0.89217068	−0.90373739	−1.83341912
H	3.04414110	1.61836639	2.95530698
H	1.53569598	2.52578439	3.20011105
H	1.75904455	0.93959516	3.98106169
H	−0.89308272	−0.04656338	−0.19041762
H	−0.66592222	1.46797307	2.11483165
H	4.33726520	0.79433327	1.38004486
C	4.29594418	−1.38337657	1.30910648
C	4.27497532	−1.55235294	2.69319565
C	4.48770247	−2.80299830	3.25854227
C	4.72901521	−3.90431323	2.43847014
C	4.76976616	−3.73857590	1.05741526
C	4.56027691	−2.48180847	0.49654104
H	4.09117755	−0.69330238	3.33281629
H	4.47044741	−2.92311440	4.33644047
H	4.89611774	−4.88194183	2.87643426
H	4.97494062	−4.58963540	0.41688912
H	4.61397107	−2.32094986	−0.57412766

III(benzaldehyde)

E (TPSSh/def2-TZVP) =	−650.68943059
$G - E$ (TPSSh/def2-TZVP) =	0.19906080
$H - E$ (TPSSh/def2-TZVP) =	0.25172333
E (DLPNO-CCSD(T)tight/def-TZVPP//TPSSh/def2-TZVP) =	−646.48619320
E (DLPNO-CCSD(T)tight/def-QZVPP//TPSSh/def2-TZVP) =	−646.51414133
E (DLPNO-CCSD(T)/CBS//TPSSh/def2-TZVP) =	−649.525215217

C	−0.09134510	0.19417716	0.10801545
C	−0.03266030	0.38121366	1.44870347
N	1.30003204	0.29881605	1.80201520
C	2.05450564	0.05970491	0.70610230
N	1.20525881	−0.00113920	−0.33280230
C	1.82469266	0.39274895	3.15959272
C	3.60797214	−0.04047783	0.66213458
O	4.11273906	−0.04824325	−0.55318755
C	1.58740910	−0.25800436	−1.73032038
H	2.68627175	−0.23240035	−1.73379575
H	1.20280545	−1.23514591	−2.02562198
H	1.14703172	0.51908666	−2.35578607
H	2.12779687	−0.59297512	3.51161027
H	2.69317626	1.05076820	3.16313165
H	1.04542914	0.80242759	3.79947349
H	−0.92637566	0.18601133	−0.57021240
H	−0.80536825	0.56945595	2.17351687
H	3.89866400	0.84921752	1.29876892
C	3.97888231	−1.27203310	1.51427237
C	4.93978855	−1.17649579	2.51738933
C	5.32967489	−2.30165622	3.23996757
C	4.75157285	−3.53753763	2.96852447
C	3.79153249	−3.64373314	1.96310689
C	3.41418148	−2.51854150	1.24037583
H	5.39083625	−0.21033249	2.72457668
H	6.08481366	−2.21427186	4.01376666
H	5.05045610	−4.41491971	3.53082516
H	3.34146577	−4.60604998	1.74380963
H	2.68291832	−2.60625253	0.44221391

2(benzaldehyde)

E (TPSSh/def2-TZVP) =	−825.71643996
$G - E$ (TPSSh/def2-TZVP) =	0.32310530
$H - E$ (TPSSh/def2-TZVP) =	0.39154799
E (DLPNO-CCSD(T)tight/def-TZVPP//TPSSh/def2-TZVP) =	−820.30033836
E (DLPNO-CCSD(T)tight/def-QZVPP//TPSSh/def2-TZVP) =	−820.33592656
E (DLPNO-CCSD(T)/CBS//TPSSh/def2-TZVP) =	−824.231433225

C	2.85691652	4.34137167	1.74734243
C	2.53889456	3.55133013	0.63877997
C	3.09861343	3.84288422	−0.61081936
C	3.95967764	4.91984514	−0.74725373
C	4.25850486	5.71848347	0.35983241
C	3.70684485	5.43185014	1.60591675
C	1.60505340	2.42609654	0.80164239
O	1.09048120	1.81607633	−0.11730488
C	−1.60616199	4.09867847	2.01050752
N	−2.13700585	3.73150386	0.69726209
C	−2.53995520	2.32259390	0.68510851
C	−3.26673637	4.59305850	0.34273566
C	−0.20761007	4.13418629	−1.68851004
N	0.02619336	3.33060391	−2.72871257
C	0.81091877	3.99831593	−3.64847360
C	1.04937603	5.23604660	−3.14215480
N	0.40217139	5.30059333	−1.92419030
C	−0.43675376	1.94466282	−2.84615421
C	0.44565831	6.42503246	−0.98772071
H	0.42709827	1.28465598	−2.89074934
H	−4.10543928	4.49316881	1.04753276
H	1.61294677	6.06394070	−3.53515217
H	−0.81762615	3.90661044	−0.80086442
H	−1.04554726	1.84424682	−3.74361131
H	−1.01852560	1.70335864	−1.96227805
H	1.44906997	6.50499396	−0.57022121
H	0.17837161	7.34111465	−1.51162086
H	−0.27005666	6.23353920	−0.19265694
H	1.12298238	3.54114161	−4.57130945
H	−2.94668772	5.63768815	0.34138582
H	−3.62543251	4.33511313	−0.65600307
H	−3.30910462	2.10453050	1.44069408
H	−2.95069849	2.07114598	−0.29553687
H	−1.67030731	1.69153514	0.87605865
H	−2.35942625	3.99825402	2.80626557
H	−0.76166022	3.45347500	2.25627551
H	−1.26361392	5.13599163	1.99499233
H	1.36845278	2.16193569	1.85092941
H	2.43436260	4.09764830	2.71679281
H	3.95176125	6.04709272	2.46292044
H	4.93720176	6.55638685	0.25150916
H	4.41645505	5.13470668	−1.70630197
H	2.86380649	3.20161364	−1.45199342

TS$_{\mathbf{2\text{-}IV}}$(benzaldehyde)

E (TPSSh/def2-TZVP) =	−825.68482526
$G - E$ (TPSSh/def2-TZVP) =	0.32581009
$H - E$ (TPSSh/def2-TZVP) =	0.39490366
E (DLPNO-CCSD(T)tight/def-TZVPP//TPSSh/def2-TZVP) =	−820.25118918
E (DLPNO-CCSD(T)tight/def-QZVPP//TPSSh/def2-TZVP) =	−820.28660265
E (DLPNO-CCSD(T)/CBS//TPSSh/def2-TZVP) =	−824.195177681

C	0.14479185	4.81676986	2.53633680
C	1.09214142	4.70982306	1.51512713
C	2.37902371	4.25003727	1.80896128
C	2.71099015	3.87962256	3.10335943
C	1.75692225	3.97064219	4.11540025
C	0.47797890	4.44489322	3.83171030
C	0.75761698	5.11912953	0.13733945
O	−0.32355805	5.70457282	−0.12839463
C	0.60474367	2.96889037	−0.61801079
N	1.61296158	2.11206048	−0.92465612
C	1.31093478	0.81083898	−0.56049382
C	0.07473697	0.84720169	−0.00912601
N	−0.33472879	2.16747308	−0.05638464
C	2.86550593	2.51035180	−1.55083557
C	−1.60223191	2.65338570	0.47580434
N	−0.95165813	5.98665145	−2.60608314
C	−1.95669423	4.91292725	−2.83077369
C	−1.53757128	7.34074362	−2.79711574
C	0.24988698	5.78073510	−3.45786017
H	−1.67459427	2.41169279	1.53638620
H	−2.29280768	4.93692860	−3.86770517
H	1.98285819	−0.01300487	−0.73130884
H	−0.63285118	5.90354647	−1.54937154
H	−2.43384473	2.19131707	−0.05864929
H	−1.62939583	3.73231364	0.34824277
H	3.70557579	2.28339569	−0.89300453
H	2.99832392	1.98525414	−2.49762860
H	2.82910772	3.58146027	−1.73448917
H	−0.53839670	0.06158266	0.39863396
H	−1.48776605	3.95634759	−2.60596076
H	−2.79883794	5.07920338	−2.16171335
H	−1.87691204	7.45048995	−3.82746524
H	−2.37519448	7.45707253	−2.11209457
H	−0.77598991	8.08530843	−2.57270198
H	−0.03129294	5.84288613	−4.50952143
H	0.98163264	6.55243076	−3.22454560
H	0.65995482	4.79695971	−3.23574254
H	1.61956183	5.23097518	−0.53048925
H	3.11937720	4.18852121	1.01781541
H	3.71005804	3.52716389	3.32943922
H	2.01502680	3.68376419	5.12777913
H	−0.25388382	4.53385695	4.62562546
H	−0.83655217	5.21147364	2.30402806

IV(benzaldehyde)

E (TPSSh/def2-TZVP) =	−825.72617060
$G - E$ (TPSSh/def2-TZVP) =	0.32731393
$H - E$ (TPSSh/def2-TZVP) =	0.39480868
E (DLPNO-CCSD(T)tight/def-TZVPP//TPSSh/def2-TZVP) =	−820.30098252
E (DLPNO-CCSD(T)tight/def-QZVPP//TPSSh/def2-TZVP) =	−820.33633474
E (DLPNO-CCSD(T)/CBS//TPSSh/def2-TZVP) =	−824.247499644

C	−0.00504204	4.93865726	1.42500561
C	0.96137647	4.34642002	0.61688578
C	2.30938801	4.47572829	0.95175781
C	2.68791138	5.18303151	2.08626406
C	1.71828341	5.77201153	2.89382598
C	0.37375399	5.65164931	2.55923724
C	0.55992936	3.57013080	−0.63882358
O	−0.76018459	3.77050507	−1.05318688
C	0.72771073	2.09145723	−0.41214505
N	1.65079322	1.29843005	−0.98647662
C	1.50259695	0.00814158	−0.51587748
C	0.46282444	0.02370575	0.35390076
N	−0.00605361	1.31929311	0.40498170
C	2.65214490	1.70821845	−1.97597890
C	−1.12793402	1.76875424	1.23897994
N	−1.06401709	6.24804062	−1.96976182
C	−1.53474534	6.11428866	−3.35553871
C	−2.08047610	6.91206958	−1.14227597
C	0.20162073	6.99233102	−1.91965418
H	−0.74316951	2.21483961	2.15528603
H	−1.72614345	7.09349443	−3.81388653
H	2.13667627	−0.79487082	−0.84779456
H	−0.85944672	4.74164323	−1.39570799
H	−1.73827004	0.89948257	1.47180514
H	−1.70198018	2.49771855	0.67427100
H	3.10226674	0.80736467	−2.38522804
H	2.17071754	2.26397584	−2.77831324
H	3.42307133	2.31650637	−1.50529816
H	0.01172933	−0.76510294	0.92981924
H	−0.78022102	5.59194633	−3.94562046
H	−2.45678044	5.53202329	−3.36914500
H	−2.30994300	7.91856211	−1.51639273
H	−2.99555079	6.31829113	−1.14659328
H	−1.71351593	6.99531383	−0.11864223
H	0.08450640	8.01661959	−2.29766133
H	0.55663066	7.03515314	−0.88904053
H	0.94716619	6.48180577	−2.53224997
H	1.27430839	3.83931709	−1.42975300
H	3.07344978	4.03746594	0.31658456
H	3.73734446	5.28393004	2.33536697
H	2.01186485	6.32899922	3.77514045
H	−0.38343558	6.11604314	3.17992180
H	−1.04950805	4.85123880	1.15365017

5.2.2 Triazolium

I

E (TPSSh/def2-TZVP) =	−495.98445231
$G - E$ (TPSSh/def2-TZVP) =	0.21035766
$H - E$ (TPSSh/def2-TZVP) =	0.26322155
E (DLPNO-CCSD(T)tight/def-TZVPP//TPSSh/def2-TZVP) =	−492.71653445
E (DLPNO-CCSD(T)tight/def-QZVPP//TPSSh/def2-TZVP) =	−492.73893993
E (DLPNO-CCSD(T)/CBS//TPSSh/def2-TZVP) =	−495.111300296

N	1.88046000	2.72919100	−1.14975900
N	2.17226500	1.45239100	−0.77732500
C	1.01569600	0.95072400	−0.44320300
N	0.01107200	1.86796300	−0.59729300
C	0.58821400	2.99658400	−1.04180300
C	−1.41037200	1.69005700	−0.27525300
C	2.95017200	3.62200300	−1.58722900
N	−0.79865400	5.62864700	−1.56459700
C	0.00323400	6.67352100	−0.92254800
C	−0.95358600	5.89889400	−2.99694400
C	−2.10724300	5.52094500	−0.91409300
H	−1.56636200	0.64946500	−0.00156600
H	−2.67941300	6.45777200	−0.97692000
H	0.08361900	3.96435000	−1.26033400
H	−2.01152600	1.93091100	−1.14965600
H	−1.66347100	2.34110100	0.55883900
H	3.65561600	3.75078100	−0.76845300
H	3.45061700	3.17506100	−2.44401200
H	2.50464900	4.57527600	−1.85990800
H	0.85984400	−0.05713800	−0.09495200
H	−2.69159100	4.73415600	−1.39636600
H	−1.96972500	5.26797000	0.13910500
H	−0.47829000	7.66023300	−0.98130400
H	0.15366900	6.42182300	0.12893300
H	0.97707600	6.74222300	−1.41229200
H	−1.47502800	6.84823800	−3.18582500
H	0.02976600	5.94815100	−3.46912300
H	−1.52583200	5.09331800	−3.46123100

II(formaldehyde)

E (TPSSh/def2-TZVP) =	−435.55112168
$G - E$ (TPSSh/def2-TZVP) =	0.10839270
$H - E$ (TPSSh/def2-TZVP) =	0.15436508
E (DLPNO-CCSD(T)tight/def-TZVPP//TPSSh/def2-TZVP) =	−432.86644507
E (DLPNO-CCSD(T)tight/def-QZVPP//TPSSh/def2-TZVP) =	−432.88577619
E (DLPNO-CCSD(T)/CBS//TPSSh/def2-TZVP) =	−434.831392613

C	0.01524212	−0.09958459	0.09298892
N	−0.06710385	−0.11081770	1.39086470
N	1.24768395	0.05201010	1.77259606
C	2.13687119	0.16039772	0.76931365
N	1.31131945	0.05747491	−0.31994323
C	1.55240838	0.07875622	3.18989157
C	1.75979861	0.14481723	−1.70557074
C	4.54834469	−0.75240026	0.25733452
O	4.46845540	−1.41394442	−0.75681429
H	2.73990831	−0.32043396	−1.78553837
H	1.04972448	−0.38270810	−2.34225436
H	1.81849449	1.18915867	−2.01656665
H	2.62300673	0.23741418	3.29131781
H	1.00492457	0.89098430	3.66897996
H	1.26625370	−0.86835188	3.64875478
H	−0.82634476	−0.20563131	−0.57269726
H	4.44635068	−1.20495368	1.25983273
H	4.81865586	0.31621759	0.24835619

$TS_{II\text{-}III}$(formaldehyde)

E (TPSSh/def2-TZVP) =	−435.54730389
$G-E$ (TPSSh/def2-TZVP) =	0.11073570
$H-E$ (TPSSh/def2-TZVP) =	0.15489252
E (DLPNO-CCSD(T)tight/def-TZVPP//TPSSh/def2-TZVP) =	−432.84359911
E (DLPNO-CCSD(T)tight/def-QZVPP//TPSSh/def2-TZVP) =	−432.86311013
E (DLPNO-CCSD(T)/CBS//TPSSh/def2-TZVP) =	−434.822367201

C	0.10408973	−0.10212647	0.06312068
N	0.05270740	−0.15563490	1.36332477
N	1.37305770	−0.01892311	1.72416394
C	2.21776570	0.11593140	0.69325637
N	1.38554740	0.06267368	−0.38174408
C	1.72000555	−0.02746544	3.13280411
C	1.83659687	0.11342465	−1.77108814
C	4.04449022	−0.51345712	0.44942964
O	4.00514840	−1.40848837	−0.42765007
H	2.74330588	−0.49412083	−1.83422557
H	1.05055654	−0.29133107	−2.40680080
H	2.04409827	1.14528319	−2.05589540
H	2.80480153	−0.02683960	3.20836013
H	1.31090430	0.85793563	3.62028013
H	1.31020237	−0.92286011	3.59859541
H	−0.75315059	−0.18103200	−0.58543163
H	4.17575835	−0.79125910	1.51672059
H	4.50171738	0.47210156	0.23739390

III(formaldehyde)

E (TPSSh/def2-TZVP) =	−435.55543621
$G - E$ (TPSSh/def2-TZVP) =	0.11272519
$H - E$ (TPSSh/def2-TZVP) =	0.15651480
E (DLPNO-CCSD(T)tight/def-TZVPP//TPSSh/def2-TZVP) =	−432.85146532
E (DLPNO-CCSD(T)tight/def-QZVPP//TPSSh/def2-TZVP) =	−432.87135587
E (DLPNO-CCSD(T)/CBS//TPSSh/def2-TZVP) =	−434.832723192

C	0.15777406	0.01317969	0.04846156
N	0.11510072	−0.08503695	1.34505609
N	1.43945745	−0.03020031	1.71496952
C	2.26470082	0.06196571	0.66333496
N	1.43572306	0.13430413	−0.41502732
C	1.79496873	−0.19787753	3.11025183
C	1.90834494	0.04829900	−1.79327999
C	3.73558206	−0.25651102	0.55953432
O	3.65099326	−1.53319799	0.15544613
H	2.66006765	−0.74569654	−1.80801910
H	1.06382793	−0.19936843	−2.43340174
H	2.34042531	0.99877357	−2.10571166
H	2.87943317	−0.19492211	3.18365278
H	1.37382278	0.61857165	3.69660314
H	1.40227323	−1.15030613	3.46495312
H	−0.70863640	0.00900178	−0.59202051
H	4.21857612	−0.06500914	1.54544910
H	4.20877512	0.47325362	−0.14187024

2(formaldehyde)

E (TPSSh/def2-TZVP) =	−610.55341483
$G - E$ (TPSSh/def2-TZVP) =	0.23049463
$H - E$ (TPSSh/def2-TZVP) =	0.29403595
E (DLPNO-CCSD(T)tight/def-TZVPP//TPSSh/def2-TZVP) =	−606.64646261
E (DLPNO-CCSD(T)tight/def-QZVPP//TPSSh/def2-TZVP) =	−606.67391351
E (DLPNO-CCSD(T)/CBS//TPSSh/def2-TZVP) =	−609.515648199

N	1.88045954	2.72919108	−1.14975883
C	2.15995546	1.50624954	−0.79303492
N	1.03408302	0.82991994	−0.40660293
C	0.01041959	1.69161511	−0.52519139
N	0.53301908	2.82130577	−0.97656826
C	0.95601453	−0.54329351	0.10725897
C	−0.15432088	4.07577459	−1.27093214
N	−2.87014729	1.15924233	0.21195756
C	−3.71988980	0.97641516	−0.96836727
C	−2.84995193	−0.05752743	1.02818227
C	−3.32855331	2.29900577	1.01059180
O	0.28759860	1.64504466	2.50504341
C	0.44689912	1.97566166	3.65695115
H	1.94158924	−0.99343518	0.01734800
H	−3.84606822	−0.31092051	1.41871647
H	−1.05786407	1.49869694	−0.27923184
H	0.24098291	−1.11038178	−0.48529445
H	0.65137364	−0.50861208	1.15110654
H	0.23973026	4.85250963	−0.61821891
H	0.02479470	4.33704733	−2.31205360
H	−1.21637690	3.92963488	−1.09133989
H	3.14653943	1.07277137	−0.80324394
H	−2.49932024	−0.89724611	0.42401574
H	−2.17133556	0.08224581	1.87202902
H	−4.34378857	2.14732093	1.40455428
H	−2.64844513	2.45125305	1.85075216
H	−3.33575709	3.20045691	0.39393188
H	−4.76441645	0.76429515	−0.69855014
H	−3.70052765	1.88225956	−1.57781778
H	−3.34244021	0.14378307	−1.56522172
H	0.36477978	1.25037733	4.48516232
H	0.68167842	3.01846203	3.93288246

$TS_{2\text{-}IV}$(formaldehyde)

E (TPSSh/def2-TZVP) =	−610.52806783
$G-E$ (TPSSh/def2-TZVP) =	0.23686372
$H-E$ (TPSSh/def2-TZVP) =	0.29536488
E (DLPNO-CCSD(T)tight/def-TZVPP//TPSSh/def2-TZVP) =	−606.62020235
E (DLPNO-CCSD(T)tight/def-QZVPP//TPSSh/def2-TZVP) =	−606.64753756
E (DLPNO-CCSD(T)/CBS//TPSSh/def2-TZVP) =	−609.492779892

C	2.30799358	1.01805140	−1.33876402
N	1.33081557	0.46938700	−0.54935418
C	0.38294408	1.41708197	−0.25083829
N	0.88831941	2.48682972	−0.89762486
N	2.06571251	2.27267085	−1.57322629
C	1.28010992	−0.92496673	−0.12561888
H	2.29351968	−1.30479033	−0.00315074
C	0.31601479	3.82189719	−0.95951281
C	−0.18514707	2.70455632	2.54940107
N	−3.09992944	1.42445736	0.91399399
C	−2.71537000	−0.01761065	0.97028396
H	−3.46146255	−0.60115105	0.43260649
H	−2.35715740	1.96628051	1.41918577
H	0.75570517	−1.53266226	−0.86483125
H	0.76094999	−0.98075710	0.82871238
H	1.06421749	4.55031908	−0.64926362
H	0.00520822	4.04419302	−1.98064566
H	−0.53848685	3.85647675	−0.28881926
H	3.16656617	0.48109385	−1.70926505
H	−1.73446060	−0.10484779	0.50635796
H	−2.67793105	−0.32840880	2.01300014
C	−4.39019088	1.69010127	1.61712866
C	−3.12362342	1.91071429	−0.49900379
H	−5.18539253	1.14224417	1.11397498
H	−4.29963627	1.35887896	2.64976316
H	−4.58951407	2.75930705	1.58586161
H	−3.88901521	1.36030320	−1.04445903
H	−3.35559574	2.97402889	−0.49665010
H	−2.13526757	1.73259077	−0.91963040
O	−1.32176939	3.03300598	2.27559596
H	0.56738827	3.46098425	2.81933154
H	0.13494186	1.65352197	2.54526739

IV(formaldehyde)

E (TPSSh/def2-TZVP) =	−610.57848903
$G - E$ (TPSSh/def2-TZVP) =	0.24315193
$H - E$ (TPSSh/def2-TZVP) =	0.29675005
E (DLPNO-CCSD(T)tight/def-TZVPP//TPSSh/def2-TZVP) =	−606.65812742
E (DLPNO-CCSD(T)tight/def-QZVPP//TPSSh/def2-TZVP) =	−606.68531650
E (DLPNO-CCSD(T)/CBS//TPSSh/def2-TZVP) =	−609.542336625

C	1.21407287	3.23495953	−1.41708483
N	1.10583432	1.91993727	−1.04820176
C	0.73743045	1.91057692	0.25421077
N	0.63885296	3.19005051	0.60773937
N	0.93102460	4.02830706	−0.42470357
C	1.36053445	0.74960891	−1.89770846
H	1.56792560	1.10655994	−2.90352432
C	0.32950973	3.75749757	1.92462535
C	0.47288568	0.69105251	1.10705168
N	−2.96804978	0.74865474	0.75499414
C	−3.37724718	2.12600647	0.45084901
H	−4.39696946	2.16975425	0.04675818
H	−1.48818646	0.80775605	1.45613091
H	0.48069886	0.10840238	−1.91630067
H	2.22107598	0.19913353	−1.52110432
H	1.25148451	4.14181743	2.35929868
H	−0.37592735	4.57148369	1.77485853
H	−0.09835899	2.96654994	2.53397776
H	1.49206456	3.55822329	−2.40709204
H	−3.33637786	2.72535864	1.36121987
H	−2.69450208	2.55445199	−0.28698223
C	−2.99288135	−0.07691066	−0.45862475
C	−3.83651728	0.16560888	1.79145870
H	−4.00418736	−0.15533120	−0.87870462
H	−2.34434164	0.37217031	−1.21527905
H	−2.63437129	−1.08042808	−0.22440249
H	−4.88235593	0.12085255	1.46148060
H	−3.49317630	−0.84294641	2.02286205
H	−3.77602344	0.77299745	2.69491464
O	−0.59931845	0.87082166	1.96993697
H	1.37331092	0.49266209	1.70002978
H	0.34474371	−0.16181121	0.42716814

II(acetaldehyde)

E (TPSSh/def2-TZVP) =	−474.89709030
$G - E$ (TPSSh/def2-TZVP) =	0.13359778
$H - E$ (TPSSh/def2-TZVP) =	0.18274222
E (DLPNO-CCSD(T)tight/def-TZVPP//TPSSh/def2-TZVP) =	−471.93320875
E (DLPNO-CCSD(T)tight/def-QZVPP//TPSSh/def2-TZVP) =	−471.95432804
E (DLPNO-CCSD(T)/CBS//TPSSh/def2-TZVP) =	−474.103724266

N	0.16402586	−0.16882221	−0.23324875
C	0.13115537	−0.42524158	1.08867891
N	1.40190923	−0.04449273	1.44610978
C	2.08713376	0.39889506	0.34679435
N	1.34958588	0.34001449	−0.72248715
C	−1.68469580	0.58110333	3.36332913
C	−2.26288334	−0.77643170	3.62983003
C	1.93860915	−0.13798019	2.79815584
C	−0.92746023	−0.37240895	−1.16485534
O	−0.88025725	1.14011276	4.07673761
H	1.18345198	0.19806848	3.50486636
H	2.22063099	−1.16820954	3.02070067
H	2.81805436	0.50141522	2.87419639
H	−0.62100593	−1.06677739	−1.94813847
H	−1.76182135	−0.78518161	−0.60348304
H	−1.21122200	0.57797132	−1.61888276
H	3.10435467	0.75628316	0.36705631
H	−3.35482134	−0.70473576	3.68144244
H	−2.00782012	−1.42204589	2.78553415
H	−1.87086940	−1.19117691	4.55793505
H	−2.06071748	1.08189464	2.44906548

TS_{II-III}(acetaldehyde)

E (TPSSh/def2-TZVP) =	−474.88857601
$G - E$ (TPSSh/def2-TZVP) =	0.13686812
$H - E$ (TPSSh/def2-TZVP) =	0.18409625
E (DLPNO-CCSD(T)tight/def-TZVPP//TPSSh/def2-TZVP) =	−471.89937610
E (DLPNO-CCSD(T)tight/def-QZVPP//TPSSh/def2-TZVP) =	−471.92066610
E (DLPNO-CCSD(T)/CBS//TPSSh/def2-TZVP) =	−474.089082549

N	−0.00651161	−0.07562266	0.01899585
C	−0.02253464	−0.15493640	1.35587623
N	1.29040283	−0.01675561	1.68062380
C	2.01094371	0.13800519	0.52938089
N	1.24437662	0.10703650	−0.52288039
C	−1.36689203	0.39211898	2.60567194
C	−1.83652731	−0.93021528	3.20479973
C	1.80129876	0.00591014	3.05029970
C	−1.15050695	−0.17409208	−0.86778219
O	−0.83413083	1.26304467	3.34419420
H	1.05424852	0.52809537	3.65477481
H	1.94545133	−1.01371673	3.40922494
H	2.75258538	0.53605346	3.05825648
H	−1.08867928	−1.09332158	−1.45108187
H	−2.04820621	−0.18176041	−0.25400497
H	−1.16014310	0.68421326	−1.53833508
H	3.07980869	0.27389616	0.50250507
H	−2.69091303	−0.72268688	3.85657253
H	−2.14308250	−1.64214464	2.43250191
H	−1.04866359	−1.37562893	3.81541444
H	−2.06090577	0.73903650	1.80491998

III(acetaldehyde)

E (TPSSh/def2-TZVP) =	−474.89383157
$G - E$ (TPSSh/def2-TZVP) =	0.13868112
$H - E$ (TPSSh/def2-TZVP) =	0.18550066
E (DLPNO-CCSD(T)tight/def-TZVPP//TPSSh/def2-TZVP) =	−471.90453374
E (DLPNO-CCSD(T)tight/def-QZVPP//TPSSh/def2-TZVP) =	−471.92613396
E (DLPNO-CCSD(T)/CBS//TPSSh/def2-TZVP) =	−474.096811895

N	−0.07581031	−0.00260967	0.03930767
C	−0.10879393	−0.05021081	1.37813686
N	1.20166712	−0.06997846	1.74362881
C	1.94529495	−0.00912602	0.60307141
N	1.20213831	0.03102967	−0.46525808
C	−1.27529522	0.23573862	2.34376231
C	−1.58394883	−1.00110106	3.21659643
C	1.68049244	0.03205124	3.12287507
C	−1.19490726	0.01146710	−0.88547912
O	−0.90091307	1.34041378	2.98480079
H	0.95542264	0.68217306	3.62739274
H	1.71373728	−0.95564302	3.58263744
H	2.67867543	0.46686433	3.10642043
H	−1.29747302	−0.96516677	−1.35985386
H	−2.09601723	0.25007747	−0.32540967
H	−1.00509197	0.77043399	−1.64206691
H	3.02258835	0.01062839	0.59809867
H	−2.41275836	−0.73651932	3.87622192
H	−1.86976476	−1.87578006	2.61934252
H	−0.72876140	−1.26139899	3.84485874
H	−2.16298019	0.35745653	1.67143681

2(acetaldehyde)

E (TPSSh/def2-TZVP) =	−649.90497998
$G - E$ (TPSSh/def2-TZVP) =	0.25826140
$H - E$ (TPSSh/def2-TZVP) =	0.32653924
E (DLPNO-CCSD(T)tight/def-TZVPP//TPSSh/def2-TZVP) =	−645.71205420
E (DLPNO-CCSD(T)tight/def-QZVPP//TPSSh/def2-TZVP) =	−645.74127337
E (DLPNO-CCSD(T)/CBS//TPSSh/def2-TZVP) =	−648.790165824

N	0.50855967	3.36830128	−0.73342873
N	1.82777643	3.38658063	−1.07071191
C	2.15119943	2.12484867	−1.13912561
N	1.07970119	1.31936319	−0.86098129
C	0.04590805	2.13507485	−0.59755100
C	1.06947153	−0.14614643	−0.77599055
C	−0.21598996	4.62348564	−0.55737791
N	−2.74311143	1.34472917	0.26021466
C	−3.75285790	1.59351230	−0.77220135
C	−2.66530262	−0.08421957	0.57424993
C	−3.03485934	2.11536973	1.47145789
O	0.55362369	1.15596133	2.16708514
C	0.87724027	1.06982770	3.33565068
C	0.54625350	2.07712273	4.38591928
H	0.25113348	−0.53519653	−1.37815097
H	−3.99204128	1.82369308	1.92770957
H	−0.98649817	1.83902304	−0.30917273
H	0.95069880	−0.42981983	0.26780881
H	2.01535416	−0.51389365	−1.16612885
H	−1.24403473	4.38563763	−0.29643070
H	0.25832012	5.19229195	0.24015517
H	−0.17799651	5.18513037	−1.48882109
H	3.13204551	1.75396142	−1.38737214
H	−2.23670060	1.95918077	2.19885981
H	−3.08723703	3.17858306	1.22632127
H	−4.75915452	1.28825445	−0.45068077
H	−3.77797083	2.65875873	−1.01112221
H	−3.49665479	1.03723025	−1.67600143
H	−3.61039199	−0.47020861	0.98304308
H	−2.43416241	−0.64600783	−0.33359719
H	−1.87495447	−0.25163694	1.30840159
H	1.45872576	0.19183859	3.67919931
H	1.47269011	2.43186353	4.85010296
H	−0.01543845	2.91463393	3.97521442
H	−0.02588168	1.59005640	5.18309886

$TS_{2\text{-}IV}$(acetaldehyde)

E (TPSSh/def2-TZVP) =	−649.88118852
$G-E$ (TPSSh/def2-TZVP) =	0.26396313
$H-E$ (TPSSh/def2-TZVP) =	0.32745369
E (DLPNO-CCSD(T)tight/def-TZVPP//TPSSh/def2-TZVP) =	−645.67860340
E (DLPNO-CCSD(T)tight/def-QZVPP//TPSSh/def2-TZVP) =	−645.70769993
E (DLPNO-CCSD(T)/CBS//TPSSh/def2-TZVP) =	−648.765321187

N	1.78162938	3.17242996	−1.40842665
C	2.18429251	1.94600492	−1.55863458
N	1.56651502	1.11304572	−0.66040411
C	0.70857419	1.83848065	0.12284476
N	0.88169468	3.07595415	−0.37453391
C	1.79124333	−0.32393341	−0.54582406
C	0.24936432	4.29890025	0.09506401
C	−0.18543776	1.65957297	2.54598861
O	−1.25864133	2.27058721	2.51753617
N	−3.15683465	1.40022092	0.90759566
C	−2.82711133	0.01262406	0.47393520
C	−4.47332110	1.46701935	1.60411983
C	−3.08898619	2.34990305	−0.24025938
H	1.47134132	−0.83274550	−1.45592220
H	−5.26112031	1.16944351	0.91273837
H	−2.39924740	1.70486712	1.60824484
H	1.21026279	−0.68656230	0.29845502
H	2.84791501	−0.52627999	−0.37037382
H	−0.39837757	4.04411975	0.93029858
H	1.01699619	5.00242378	0.41574682
H	−0.32581582	4.74812419	−0.71486763
H	2.90775568	1.62534654	−2.29111353
H	−4.45017078	0.79421259	2.45922490
H	−4.63565218	2.48835299	1.94269332
H	−3.83619955	2.06972042	−0.98214743
H	−3.28437930	3.35497201	0.12804896
H	−2.08890268	2.29483854	−0.66601488
H	−3.56229206	−0.32012187	−0.25825639
H	−1.83080743	0.02425507	0.03524299
H	−2.84776801	−0.64038873	1.34465683
C	1.04774757	2.24050976	3.15318547
H	−0.15568460	0.58701795	2.31309150
H	1.94711238	1.80758772	2.71716497
H	1.05743412	3.32616075	3.06828448
H	1.03281456	1.98295288	4.22016425

IV(acetaldehyde)

E (TPSSh/def2-TZVP) =	−649.91958504
$G - E$ (TPSSh/def2-TZVP) =	0.26910523
$H - E$ (TPSSh/def2-TZVP) =	0.32593815
E (DLPNO-CCSD(T)tight/def-TZVPP//TPSSh/def2-TZVP) =	−645.71325028
E (DLPNO-CCSD(T)tight/def-QZVPP//TPSSh/def2-TZVP) =	−645.74222270
E (DLPNO-CCSD(T)/CBS//TPSSh/def2-TZVP) =	−648.808879433

N	0.40150646	1.94224899	3.70721271
C	0.23401633	3.20435993	3.43871617
N	0.27356199	3.43363989	2.08814161
C	0.48125745	2.23505266	1.49396250
N	0.55571241	1.35298133	2.48829706
C	0.12707935	4.73148445	1.41894993
C	0.81243978	−0.08919471	2.42611100
C	0.60031304	1.98158465	0.00362477
O	−0.01115907	0.77781911	−0.34442328
N	−2.67246224	0.92470989	−0.47696060
C	−3.17552099	2.20573693	−0.98737997
C	−2.99979914	−0.16485520	−1.41163708
C	−3.22411965	0.64363917	0.85401949
H	−0.71355875	4.69696927	0.72757805
H	−4.08475671	−0.27795627	−1.53355764
H	−1.03122303	0.89042203	−0.38726123
H	1.04273396	4.97932767	0.88477259
H	−0.06262156	5.48165987	2.18261890
H	0.67800263	−0.40579795	1.39600804
H	1.82812152	−0.27497575	2.77291479
H	0.10158867	−0.57833639	3.08822387
H	0.07813356	3.97724531	4.17365485
H	−2.55362833	0.04796165	−2.38339498
H	−2.58684995	−1.10041862	−1.03294289
H	−4.31908573	0.56561876	0.83570556
H	−2.81421863	−0.29716588	1.22447730
H	−2.94769670	1.44675686	1.54134174
H	−4.26660095	2.19686245	−1.11081090
H	−2.91757965	3.00384583	−0.28711879
H	−2.71718420	2.41685609	−1.95459257
C	2.07224296	1.94785250	−0.40999365
H	0.11353312	2.83631009	−0.48727319
H	2.59177472	2.86767234	−0.13145452
H	2.57579720	1.10074993	0.05794624
H	2.12067112	1.82635412	−1.49177886

II(acrolein)

E (TPSSh/def2-TZVP) =	−512.99459666
$G-E$ (TPSSh/def2-TZVP) =	0.13897183
$H-E$ (TPSSh/def2-TZVP) =	0.19104464
E (DLPNO-CCSD(T)tight/def-TZVPP//TPSSh/def2-TZVP) =	−509.78498170
E (DLPNO-CCSD(T)tight/def-QZVPP//TPSSh/def2-TZVP) =	−509.80736108
E (DLPNO-CCSD(T)/CBS//TPSSh/def2-TZVP) =	−512.127516935

N	1.17284529	0.02266827	−0.22977900
C	−0.11266587	−0.20686023	0.18144735
N	−0.19406075	−0.23498467	1.47916549
N	1.11151793	−0.00755532	1.86146640
C	1.99452992	0.15749695	0.86022564
C	1.41584635	0.02361877	3.27845432
C	1.61738619	0.13364108	−1.61459766
C	4.57097134	−0.63660013	0.09709891
O	4.42404133	−1.41478281	−0.82880182
H	2.53602573	−0.43585706	−1.74137915
H	0.84078018	−0.26827170	−2.26501718
H	1.79755802	1.17901540	−1.86678836
H	2.47871913	0.22812097	3.37904280
H	0.83380609	0.80762269	3.76411201
H	1.17271003	−0.93822358	3.73144798
H	−0.94750691	−0.35071955	−0.48569302
H	4.52864749	−0.99121454	1.14148960
C	4.90264652	0.79804853	−0.07065705
C	5.30470635	1.29270423	−1.24100172
H	4.83593146	1.41535589	0.81810747
H	5.57817208	2.33436671	−1.35944439
H	5.37009409	0.64935609	−2.11179663

$\mathbf{TS_{II\text{-}III}}$(acrolein)

E (TPSSh/def2-TZVP) =	−512.98710637
$G - E$ (TPSSh/def2-TZVP) =	0.14224077
$H - E$ (TPSSh/def2-TZVP) =	0.19005059
E (DLPNO-CCSD(T)tight/def-TZVPP//TPSSh/def2-TZVP) =	−509.75692324
E (DLPNO-CCSD(T)tight/def-QZVPP//TPSSh/def2-TZVP) =	−509.77942203
E (DLPNO-CCSD(T)/CBS//TPSSh/def2-TZVP) =	−512.116090226

N	1.27629515	0.03955638	−0.35668685
C	0.01516877	−0.14459737	0.13587107
N	0.01364174	−0.21612078	1.43645740
N	1.34516167	−0.07077676	1.75006931
C	2.14344277	0.08630415	0.68779080
C	1.74670241	−0.08891397	3.14431223
C	1.67484563	0.10757052	−1.76196489
C	3.96564023	−0.41338448	0.37907652
O	3.94681181	−1.32827679	−0.48416358
H	2.56771329	−0.51277911	−1.86910797
H	0.85440888	−0.26838372	−2.37125208
H	1.89939389	1.13876356	−2.03246309
H	2.83368296	−0.09178809	3.17820718
H	1.35900546	0.79443514	3.65250960
H	1.35263422	−0.98647814	3.61896927
H	−0.86503427	−0.22438726	−0.48094955
H	4.15107441	−0.68649446	1.44330144
C	4.55030782	0.94049994	0.06980983
C	5.16398446	1.19937326	−1.08011195
H	4.47224286	1.69160253	0.85396798
H	5.60590439	2.16594300	−1.29228734
H	5.24086645	0.42291146	−1.83355933

III(acrolein)

E (TPSSh/def2-TZVP) =	−512.99309155
$G - E$ (TPSSh/def2-TZVP) =	0.14352129
$H - E$ (TPSSh/def2-TZVP) =	0.19136406
E (DLPNO-CCSD(T)tight/def-TZVPP//TPSSh/def2-TZVP) =	−509.76292311
E (DLPNO-CCSD(T)tight/def-QZVPP//TPSSh/def2-TZVP) =	−509.78566770
E (DLPNO-CCSD(T)/CBS//TPSSh/def2-TZVP) =	−512.12448955

N	1.35206919	0.10410334	−0.37189917
C	0.09338033	−0.07082909	0.12002226
N	0.08843087	−0.22326907	1.41329273
N	1.41973279	−0.14574477	1.74561693
C	2.21131394	0.03540881	0.68010366
C	1.80478868	−0.28186216	3.13871536
C	1.76200769	0.13706334	−1.77530594
C	3.72517266	−0.16216238	0.54019302
O	3.82038251	−1.27645095	−0.17755644
H	2.57882240	−0.58401551	−1.86343093
H	0.90240719	−0.13520279	−2.38551687
H	2.11465601	1.13323614	−2.03695085
H	2.88019897	−0.43614223	3.18155945
H	1.53329243	0.61969104	3.68882640
H	1.28283333	−1.14036156	3.55740959
H	−0.78831814	−0.08305822	−0.49911587
H	4.10606827	−0.23167326	1.59297492
C	4.36709627	1.10804545	−0.01676470
C	5.09367394	1.07583942	−1.12691337
H	4.24697183	2.02461664	0.56137910
H	5.59748366	1.95305885	−1.51641027
H	5.20262117	0.13192296	−1.65174099

2(acrolein)

E (TPSSh/def2-TZVP) =	−688.00243027
$G - E$ (TPSSh/def2-TZVP) =	0.26358101
$H - E$ (TPSSh/def2-TZVP) =	0.33294796
E (DLPNO-CCSD(T)tight/def-TZVPP//TPSSh/def2-TZVP) =	−683.56712234
E (DLPNO-CCSD(T)tight/def-QZVPP//TPSSh/def2-TZVP) =	−683.59758049
E (DLPNO-CCSD(T)/CBS//TPSSh/def2-TZVP) =	−686.815594735

N	1.17458939	6.62422564	−1.90599775
N	2.48381196	6.89865529	−2.16110913
C	2.99931643	5.73311352	−2.43883859
N	2.05774138	4.74163559	−2.37006953
C	0.90380830	5.33294431	−2.02081564
C	2.26874241	3.30132582	−2.56083505
C	0.25992048	7.70440134	−1.54929426
C	1.61335843	3.43902193	1.62007695
C	1.25310025	4.13167628	2.86671322
O	1.38085076	3.85997640	0.49673027
N	−1.77172634	4.02689824	−1.49465167
C	−2.20427367	4.44230464	−0.15803987
C	−2.77046500	4.39211284	−2.50270368
C	−1.51492925	2.58465413	−1.52038523
H	3.29587904	3.15089394	−2.88398849
H	−2.41410874	1.99951399	−1.27887276
H	−0.07956949	4.84364339	−1.84731815
H	1.58782034	2.93516301	−3.32666590
H	2.09057432	2.80121191	−1.61120775
H	0.62626938	8.18933348	−0.64632609
H	0.22497587	8.42151171	−2.36732024
H	−0.72289140	7.27199269	−1.37930726
H	4.03220115	5.56255067	−2.69435603
H	−1.17697112	2.29150428	−2.51698110
H	−0.73693045	2.34268733	−0.79415593
H	−3.13369968	3.94199264	0.15067616
H	−1.42216290	4.20589886	0.56475789
H	−2.38169560	5.52032307	−0.14860783
H	−3.73740240	3.89881940	−2.32698826
H	−2.92973661	5.47244439	−2.48671563
H	−2.41248898	4.10488607	−3.49336388
H	2.12843172	2.46937100	1.74481655
C	0.66726018	5.33145724	2.86607396
H	1.49396218	3.62263047	3.79375151
H	0.40600798	5.84151384	3.78467101
H	0.44053270	5.83055966	1.93038616

$TS_{2\text{-}IV}$(acrolein)

E (TPSSh/def2-TZVP) =	−687.97723471
$G - E$ (TPSSh/def2-TZVP) =	0.26799229
$H - E$ (TPSSh/def2-TZVP) =	0.33404343
E (DLPNO-CCSD(T)tight/def-TZVPP//TPSSh/def2-TZVP) =	−683.53908122
E (DLPNO-CCSD(T)tight/def-QZVPP//TPSSh/def2-TZVP) =	−683.56950404
E (DLPNO-CCSD(T)/CBS//TPSSh/def2-TZVP) =	−686.793190774

N	1.83991680	6.37489445	−1.73020243
N	3.19437578	6.46667624	−1.94782862
C	3.55038032	5.22333545	−2.07346024
N	2.47108087	4.38912903	−1.93550673
C	1.33226926	5.12719316	−1.71516666
C	2.51095299	2.93558414	−2.04798269
C	1.10178489	7.61505207	−1.56247854
C	0.96978148	4.12238899	1.15958020
C	0.79952179	5.52132376	1.56699512
O	0.05813708	3.35698343	0.86793350
N	−1.90752710	3.70152674	−0.99044648
C	−3.08149422	3.07051692	−0.31924558
C	−2.19379986	5.09688807	−1.43784654
C	−1.40885100	2.87642181	−2.13225401
H	3.50942333	2.58196273	−1.79442694
H	−2.19792657	2.80269093	−2.87948921
H	−1.13795683	3.73685356	−0.28219958
H	2.26974997	2.62331866	−3.06515145
H	1.79120598	2.50810304	−1.35260823
H	1.61380192	8.24141352	−0.83335774
H	1.04376903	8.14646549	−2.51275460
H	0.10402278	7.36854956	−1.20894804
H	4.56012122	4.89101493	−2.25448508
H	−0.53194039	3.38170333	−2.53251582
H	−1.14862286	1.88853213	−1.75764327
H	−3.89654464	2.98920086	−1.03667579
H	−2.78957666	2.08483884	0.03696346
H	−3.38143279	3.69497026	0.51997309
H	−2.98316251	5.06806068	−2.18787205
H	−2.51524241	5.68057323	−0.57738660
H	−1.26693119	5.49260610	−1.84928446
H	2.00597341	3.74647064	1.15654562
C	−0.35714826	5.98110938	2.04980329
H	1.68666992	6.14478572	1.53695672
H	−0.46458235	6.99954663	2.40164602
H	−1.21848217	5.32829251	2.14250341

IV(acrolein)

E (TPSSh/def2-TZVP) =	−688.01196608
$G - E$ (TPSSh/def2-TZVP) =	0.27183245
$H - E$ (TPSSh/def2-TZVP) =	0.33461216
E (DLPNO-CCSD(T)tight/def-TZVPP//TPSSh/def2-TZVP) =	−683.56004281
E (DLPNO-CCSD(T)tight/def-QZVPP//TPSSh/def2-TZVP) =	−683.59030485
E (DLPNO-CCSD(T)/CBS//TPSSh/def2-TZVP) =	−686.830526727

N	1.63900223	6.13287059	−1.50951644
N	2.25187864	6.05740674	−2.72550491
C	2.77894189	4.86573772	−2.74867803
N	2.52361341	4.18781236	−1.59181158
C	1.79347758	5.01115997	−0.80364721
C	2.94265532	2.81900394	−1.25601524
C	0.96941016	7.39036334	−1.16287181
C	1.24108801	4.56171741	0.53891450
C	0.71935625	5.67960494	1.40567509
O	0.35716715	3.50542774	0.30581944
N	−1.80391843	3.92776530	−1.20922188
C	−2.88958079	3.34058448	−0.40720247
C	−2.16071032	5.28540898	−1.63484064
C	−1.51727113	3.08157878	−2.37588669
H	3.78334897	2.85474198	−0.56359117
H	−2.38866248	2.98972141	−3.03720914
H	−0.45728317	3.79861734	−0.24760640
H	3.24553839	2.32555270	−2.17625064
H	2.09706041	2.30453846	−0.80484957
H	1.69448508	8.07206010	−0.71921140
H	0.58924734	7.80865417	−2.09084135
H	0.16365110	7.18460391	−0.46608935
H	3.34083125	4.45754035	−3.57296995
H	−0.69455316	3.51782508	−2.94689771
H	−1.22682617	2.08622932	−2.03758103
H	−3.81333884	3.24187431	−0.99183494
H	−2.58600479	2.35471474	−0.05398553
H	−3.08887929	3.97891826	0.45439253
H	−3.08289037	5.29699455	−2.23076409
H	−2.30567473	5.91640258	−0.75651427
H	−1.35591483	5.69750026	−2.24684525
H	2.10541482	4.13033320	1.06512576
C	−0.46830010	5.64730139	1.99504285
H	1.41482704	6.49247687	1.60023368
H	−0.78344355	6.43772946	2.66428433
H	−1.14441388	4.81433026	1.84838251

II(benzaldehyde)

E (TPSSh/def2-TZVP) =	−666.73182945
$G - E$ (TPSSh/def2-TZVP) =	0.18409126
$H - E$ (TPSSh/def2-TZVP) =	0.23829672
E (DLPNO-CCSD(T)tight/def-TZVPP//TPSSh/def2-TZVP) =	−662.51185554
E (DLPNO-CCSD(T)tight/def-QZVPP//TPSSh/def2-TZVP) =	−662.53996078
E (DLPNO-CCSD(T)/CBS//TPSSh/def2-TZVP) =	−665.56161553

N	0.56021389	−0.53770900	0.05480373
C	0.74999519	−0.21036601	1.34730637
N	2.00804529	−0.72525505	1.55345176
C	2.47285026	−1.30903367	0.40384053
N	1.59905265	−1.21263278	−0.55304433
C	−2.22961529	1.47843332	2.24109410
O	−2.94581334	1.47786488	1.25469990
C	2.72121959	−0.64885016	2.81807094
C	−0.62057311	−0.24709261	−0.74143387
C	−2.60130888	2.09029159	3.53324234
C	−3.83426151	2.72585498	3.71007360
C	−4.15001596	3.28966912	4.93629400
C	−3.23822336	3.22228979	5.99134284
C	−2.00948543	2.59086838	5.82114044
C	−1.69236025	2.02543368	4.59189409
H	−1.33842066	0.27916852	−0.11661390
H	−0.34002888	0.37245460	−1.59396165
H	−1.05203674	−1.18062582	−1.10420676
H	2.07652165	−0.12782092	3.52133301
H	2.93985786	−1.64927887	3.19630708
H	3.65437471	−0.09483839	2.69955717
H	3.43527811	−1.78558177	0.30466138
H	−1.22973715	1.00293646	2.21369659
H	−0.73987297	1.52849216	4.43754142
H	−1.30554256	2.54158976	6.64380871
H	−3.48911503	3.66460438	6.94892020
H	−5.10420111	3.78364107	5.07918938
H	−4.52253096	2.76319634	2.87380793

$\mathbf{TS_{II\text{-}III}}$(benzaldehyde)

E (TPSSh/def2-TZVP) =	−666.72232879
$G - E$ (TPSSh/def2-TZVP) =	0.18681761
$H - E$ (TPSSh/def2-TZVP) =	0.23937155
E (DLPNO-CCSD(T)tight/def-TZVPP//TPSSh/def2-TZVP) =	−662.46990811
E (DLPNO-CCSD(T)tight/def-QZVPP//TPSSh/def2-TZVP) =	−662.49810937
E (DLPNO-CCSD(T)/CBS//TPSSh/def2-TZVP) =	−665.547609264

N	0.02112776	−0.58939526	0.19604238
C	−0.14880593	0.01986931	1.37386057
N	1.09684275	0.49499070	1.65772666
C	1.93242766	0.14466146	0.62954625
N	1.30024230	−0.52503384	−0.28954284
C	−1.88293687	0.62233615	1.77552985
O	−2.25646510	1.25804769	0.74918088
C	1.49670260	1.25339401	2.84185758
C	−1.03560958	−1.18773125	−0.60822898
C	−1.66530045	1.36432885	3.08152957
C	−1.64178481	2.75567437	3.07973222
C	−1.41878494	3.45272832	4.26387811
C	−1.23144881	2.76114595	5.45841160
C	−1.28435999	1.36811403	5.46777458
C	−1.50371580	0.67577554	4.28199206
H	−1.89841354	−0.52046829	−0.54563709
H	−0.66636561	−1.25940515	−1.62779633
H	−1.27775672	−2.18133906	−0.22960228
H	1.02484938	2.23395038	2.83617670
H	1.19228115	0.72184288	3.74003381
H	2.58035361	1.35927050	2.82161890
H	2.97961362	0.39680432	0.59404900
H	−2.31093553	−0.38341524	1.98199707
H	−1.54375052	−0.41051782	4.28121896
H	−1.16092739	0.82716024	6.39976432
H	−1.06261874	3.30406032	6.38159918
H	−1.39784434	4.53718613	4.25904767
H	−1.80407215	3.26249674	2.13505861

III(benzaldehyde)

E (TPSSh/def2-TZVP) =	−666.73041632
$G - E$ (TPSSh/def2-TZVP) =	0.18794228
$H - E$ (TPSSh/def2-TZVP) =	0.24076332
E (DLPNO-CCSD(T)tight/def-TZVPP//TPSSh/def2-TZVP) =	−662.48065059
E (DLPNO-CCSD(T)tight/def-QZVPP//TPSSh/def2-TZVP) =	−662.50910467
E (DLPNO-CCSD(T)/CBS//TPSSh/def2-TZVP) =	−665.557384281

C	0.08897867	1.35031884	0.74814727
C	0.01483553	0.05984053	1.25811971
C	0.85220929	−0.93108097	0.74769657
C	1.74715535	−0.63832124	−0.27630787
C	1.81789194	0.65662535	−0.78703319
C	0.99157680	1.65022864	−0.26903687
C	−0.99883962	−0.22208344	2.37538072
O	−1.91042526	0.72593746	2.62708311
C	−1.91868202	−1.37092627	2.01846590
N	−2.39620167	−2.31036002	2.84477187
N	−3.49654312	−2.95430825	2.33254312
C	−3.65162409	−2.40555301	1.16264034
N	−2.70532880	−1.45240357	0.91249763
C	−1.96821669	−2.57073654	4.20952166
C	−2.66833790	−0.51766300	−0.20907172
H	−2.69898766	0.48913093	0.20839004
H	−1.75250259	−0.65469424	−0.78116188
H	−3.53759388	−0.70916010	−0.83598166
H	−0.97990603	−3.03016805	4.21872268
H	−1.95022777	−1.62978677	4.75957220
H	−2.69760604	−3.24900161	4.64393447
H	−4.42625267	−2.66931169	0.46194077
H	−0.39694875	−0.56292090	3.25393821
H	0.80135225	−1.94081636	1.14856344
H	2.39039116	−1.41517578	−0.67534135
H	2.51625367	0.88799746	−1.58350901
H	1.04948919	2.65957655	−0.66213868
H	−0.58456631	2.09015407	1.16727051

2(benzaldehyde)

E (TPSSh/def2-TZVP) =	−841.74353653
$G - E$ (TPSSh/def2-TZVP) =	0.31052771
$H - E$ (TPSSh/def2-TZVP) =	0.38014638
E (DLPNO-CCSD(T)tight/def-TZVPP//TPSSh/def2-TZVP) =	−836.28134437
E (DLPNO-CCSD(T)tight/def-QZVPP//TPSSh/def2-TZVP) =	−836.31764337
E (DLPNO-CCSD(T)/CBS//TPSSh/def2-TZVP) =	−840.250598319

C	0.38916054	3.54527247	2.04548087
C	−0.30024621	2.61741289	2.83252076
C	0.14880362	2.32794885	4.12784265
C	1.28296164	2.95327941	4.61931320
C	1.98510304	3.85514785	3.81654905
C	1.54050679	4.15185826	2.53112551
C	−1.47511301	1.94055093	2.27184911
O	−1.99801936	0.94711876	2.74769307
N	−2.12381772	−1.68037230	0.25988501
C	−1.76946357	−2.74704639	−0.67980467
C	−2.99649552	−2.19342607	1.32140902
C	−2.77686287	−0.56893749	−0.43754725
C	0.38537703	−0.72678157	1.60231895
N	1.40379390	−0.09596544	1.03498027
N	2.39433538	0.18737782	1.92592417
C	1.94643346	−0.28998809	3.05378493
N	0.71385255	−0.86631294	2.89778062
C	1.54574784	0.30699761	−0.36046381
C	−0.13027632	−1.46030793	3.94331093
H	2.42543404	−0.17746007	−0.77996712
H	0.39733222	−1.37287469	4.88986973
H	−3.94281194	−2.58519817	0.92188858
H	−0.55569953	−1.06888208	1.10595912
H	−0.30300549	−2.51064880	3.71646849
H	−1.06904832	−0.91076303	3.97949229
H	1.66257314	1.38853516	−0.39933466
H	0.65017260	−0.00053935	−0.89448496
H	2.47063601	−0.24174856	3.99331711
H	−2.48999240	−3.00536708	1.84875647
H	−3.21842892	−1.38919723	2.02398008
H	−3.70142790	−0.88497823	−0.94172017
H	−3.02344342	0.21057390	0.28402565
H	−2.10155175	−0.16105654	−1.19391502
H	−2.65339803	−3.17434960	−1.17458826
H	−1.10442473	−2.35205592	−1.45131949
H	−1.25302997	−3.54845634	−0.14717621
H	−1.86519740	2.37942560	1.33208557
H	0.01740754	3.78613848	1.05428549
H	2.08156262	4.86419956	1.92067752
H	2.87304395	4.34054860	4.20363422
H	1.62011227	2.75652756	5.63021617
H	−0.42338982	1.63898020	4.73837901

$\mathbf{TS_{2\text{-}IV}}$(benzaldehyde)

E (TPSSh/def2-TZVP) =	−841.71995179
$G-E$ (TPSSh/def2-TZVP) =	0.31611844
$H-E$ (TPSSh/def2-TZVP) =	0.37930368
E (DLPNO-CCSD(T)tight/def-TZVPP//TPSSh/def2-TZVP) =	−836.24629619
E (DLPNO-CCSD(T)tight/def-QZVPP//TPSSh/def2-TZVP) =	−836.28242034
E (DLPNO-CCSD(T)/CBS//TPSSh/def2-TZVP) =	−840.223760408

N	1.95119222	0.31427772	0.33850097
C	1.02867262	0.02073202	1.26570056
N	1.76539982	−0.63345664	2.20783683
C	3.07253860	−0.68599843	1.79707992
N	3.22247004	−0.11009477	0.64103685
C	1.23991277	−1.14943214	3.46931868
C	1.74827461	1.03225632	−0.90837933
H	2.08609088	0.41647493	−1.74095716
C	−0.88826304	1.05689395	1.88195406
O	−1.66696822	0.19802049	2.37110738
C	−0.18422641	2.02972062	2.74259147
C	−0.25624423	1.92482029	4.13359782
C	0.39577580	2.85438059	4.93289357
C	1.11744646	3.89316268	4.34913652
C	1.17772264	4.01151870	2.96150220
C	0.52692674	3.08388100	2.16223873
N	−2.99412948	−1.44737253	0.90348115
C	−4.43437491	−1.07694669	0.96630377
C	−2.47809143	−1.41965402	−0.49147786
C	−2.74163890	−2.76581770	1.54602664
H	1.66163926	−0.58969148	4.30402277
H	−5.02383798	−1.80535722	0.40898486
H	−2.42699608	−0.69842874	1.48796995
H	1.49256990	−2.20509772	3.57004079
H	0.16058592	−1.02350092	3.45691580
H	2.31791448	1.96117981	−0.89258333
H	0.68650088	1.24528698	−1.00684364
H	3.86853592	−1.14956545	2.35732174
H	−4.74450241	−1.06265438	2.00934384
H	−4.55957337	−0.08594748	0.53390359
H	−2.97570880	−2.18903303	−1.08220112
H	−2.67852171	−0.43897772	−0.91963416
H	−1.40456797	−1.59998571	−0.46705770
H	−3.28427239	−3.54438100	1.00942863
H	−1.67224611	−2.96710782	1.51533628
H	−3.07814808	−2.71910351	2.57989948
H	−0.99904300	1.36354731	0.83528093
H	0.56764806	3.17278633	1.08126689
H	1.72721250	4.82836823	2.50975581
H	1.62401333	4.61805721	4.97497264
H	0.33489140	2.77787045	6.01191681
H	−0.84102830	1.12529452	4.57148636

IV(benzaldehyde)

E (TPSSh/def2-TZVP) = −841.75531075
$G - E$ (TPSSh/def2-TZVP) = 0.31534442
$H - E$ (TPSSh/def2-TZVP) = 0.38257820
E (DLPNO-CCSD(T)tight/def-TZVPP//TPSSh/def2-TZVP) = −836.28648998
E (DLPNO-CCSD(T)tight/def-QZVPP//TPSSh/def2-TZVP) = −836.32254542
E (DLPNO-CCSD(T)/CBS//TPSSh/def2-TZVP) = −840.269183657

C	0.71935309	2.39004456	1.42497880
C	0.03329048	1.33819825	2.03217409
C	−0.89236590	1.60749545	3.03692454
C	−1.12792224	2.92125869	3.43252326
C	−0.43666893	3.96780962	2.83098214
C	0.48715263	3.70085194	1.82376011
C	0.29606686	−0.10163809	1.58623736
O	−0.66578765	−1.02803756	1.98628881
C	1.60712494	−0.56792557	2.16283432
N	2.77279362	−0.71539418	1.53306429
N	3.75974194	−1.13487185	2.37408095
C	3.16571735	−1.24718002	3.52746572
N	1.84262306	−0.91107536	3.44770516
C	3.11064560	−0.47552427	0.13147940
C	0.88353695	−0.91084756	4.56208336
N	−2.90611496	−0.62203226	0.64625539
C	−2.71602671	0.42833951	−0.36435640
C	−3.26698263	−1.89728528	0.00823253
C	−3.93852101	−0.21871877	1.61212264
H	3.81445258	−1.24720826	−0.16921677
H	0.71434217	0.11431127	4.88809542
H	−4.20810568	−1.81572089	−0.55060242
H	−1.54317707	−0.86332031	1.44886540
H	1.31674298	−1.49566924	5.37001159
H	−0.04435903	−1.35515427	4.21446768
H	3.57088068	0.50694679	0.03229244
H	2.20711719	−0.53399458	−0.46938074
H	3.64396005	−1.57296639	4.43660436
H	−2.47396080	−2.19563512	−0.67882422
H	−3.37667242	−2.66556410	0.77434263
H	−4.91265069	−0.08330342	1.12477229
H	−4.03364748	−0.98587884	2.38165754
H	−3.64666938	0.72251768	2.07922460
H	−3.64018181	0.62216514	−0.92405959
H	−2.40178996	1.35092004	0.12603864
H	−1.94426707	0.11675757	−1.07068602
H	0.42334179	−0.09231661	0.49488211
H	1.42460389	2.19159910	0.62336452
H	1.01769032	4.51383652	1.34317990
H	−0.62278506	4.98953037	3.13845171
H	−1.85557647	3.12703965	4.20861215
H	−1.43863921	0.78889568	3.48839834

5.2.3 Thiazolium

I

E (TPSSh/def2-TZVP) =	−783.45152760
$G - E$ (TPSSh/def2-TZVP) =	0.17797947
$H - E$ (TPSSh/def2-TZVP) =	0.22980375
E (DLPNO-CCSD(T)tight/def-TZVPP//TPSSh/def2-TZVP) =	−780.15089099
E (DLPNO-CCSD(T)tight/def-QZVPP//TPSSh/def2-TZVP) =	−780.17939880
E (DLPNO-CCSD(T)/CBS//TPSSh/def2-TZVP) =	−782.290951792

C	1.62257727	2.38603454	−1.49273175
N	2.05306486	1.29813715	−0.86366878
C	1.41579067	1.04536451	0.33734262
C	0.47429295	1.97398920	0.62205826
S	0.40108347	3.15460468	−0.62415837
C	3.12001042	0.43153233	−1.39816047
N	2.74577173	3.34955561	−4.08911619
C	2.55473239	2.38151991	−5.17422773
C	1.93844102	4.55159582	−4.32713413
C	4.16206336	3.70362519	−3.94973707
H	2.85877953	2.78874560	−6.14859100
H	1.69755221	0.18263810	0.92138051
H	2.03919296	2.73041344	−2.47551319
H	2.72175464	−0.57142378	−1.54443264
H	3.45044116	0.84552434	−2.34623089
H	3.94647321	0.40912273	−0.68930672
H	−0.16820999	2.02129883	1.48681826
H	3.15022221	1.48716005	−4.97836349
H	1.50166320	2.09933220	−5.23167807
H	2.22600044	5.06457560	−5.25523914
H	0.88424039	4.27568092	−4.40113371
H	2.06739935	5.24946582	−3.49675973
H	4.55824917	4.18452663	−4.85492385
H	4.28651558	4.39160208	−3.11112314
H	4.74943382	2.80300250	−3.75796360

II(formaldehyde)

E (TPSSh/def2-TZVP) =	−723.01765029
$G - E$ (TPSSh/def2-TZVP) =	0.07775560
$H - E$ (TPSSh/def2-TZVP) =	0.12096462
E (DLPNO-CCSD(T)tight/def-TZVPP//TPSSh/def2-TZVP) =	−720.30238850
E (DLPNO-CCSD(T)tight/def-QZVPP//TPSSh/def2-TZVP) =	−720.32788540
E (DLPNO-CCSD(T)/CBS//TPSSh/def2-TZVP) =	−722.010195682

C	0.15014709	−0.00714951	0.02065404
N	0.18757553	0.00234503	1.36573743
C	1.39435921	−0.29400419	1.99306915
C	2.38057571	−0.55386717	1.11097646
S	1.73285209	−0.41178278	−0.48835888
C	−1.01332313	0.29625576	2.15887861
C	−1.87325288	1.22512228	−1.17106803
O	−2.51321556	1.82616609	−0.33357185
H	−1.05125798	1.70136234	−1.73374308
H	1.45015302	−0.29315338	3.07197194
H	3.40558635	−0.80692647	1.32980818
H	−1.33036144	−0.60419583	2.68790750
H	−0.78837343	1.08333197	2.87985241
H	−1.79340829	0.63102443	1.48174354
H	−2.12746528	0.19904644	−1.48543941

TS$_{\text{II-III}}$(formaldehyde)

E (TPSSh/def2-TZVP) =	−723.01363823
$G - E$ (TPSSh/def2-TZVP) =	0.08026040
$H - E$ (TPSSh/def2-TZVP) =	0.12144041
E (DLPNO-CCSD(T)tight/def-TZVPP//TPSSh/def2-TZVP) =	−720.27808309
E (DLPNO-CCSD(T)tight/def-QZVPP//TPSSh/def2-TZVP) =	−720.30370951
E (DLPNO-CCSD(T)/CBS//TPSSh/def2-TZVP) =	−722.000458302

C	0.00430212	0.00000447	−0.01178741
N	0.05638128	0.02649716	1.32603024
C	1.29628290	−0.19162781	1.90819767
C	2.26940681	−0.39764027	0.99586678
S	1.58100426	−0.30735729	−0.58625364
C	−1.15148904	0.27675094	2.12972282
C	−1.43923186	0.95505373	−0.91650437
O	−1.77016623	1.92934955	−0.19855832
H	−0.84286870	1.12280791	−1.83495341
H	1.38603516	−0.17806449	2.98409295
H	3.31568369	−0.58025562	1.18000100
H	−1.69008435	−0.66066186	2.27135745
H	−0.84801016	0.67882511	3.09492591
H	−1.75951945	0.99842789	1.57509536
H	−2.10790043	0.07910558	−1.02781903

III(formaldehyde)

E (TPSSh/def2-TZVP) = −723.03183106
$G - E$ (TPSSh/def2-TZVP) = 0.08332424
$H - E$ (TPSSh/def2-TZVP) = 0.12340558
E (DLPNO-CCSD(T)tight/def-TZVPP//TPSSh/def2-TZVP) = −720.30520043
E (DLPNO-CCSD(T)tight/def-QZVPP//TPSSh/def2-TZVP) = −720.33084459
E (DLPNO-CCSD(T)/CBS//TPSSh/def2-TZVP) = −722.024604545

C	−0.07173698	0.35566479	−0.04970715
N	−0.08936200	−0.08158990	1.30765299
C	1.19601871	−0.15257224	1.84305768
C	2.19531961	−0.11734567	0.95517675
S	1.58379254	0.03809219	−0.68535629
C	−1.21202522	0.29880042	2.15226228
C	−1.23531143	0.41843880	−0.92089827
O	−0.69045191	1.63010491	−0.35020722
H	−1.10372189	0.31377264	−1.99537706
H	1.29563246	−0.25992605	2.91353860
H	3.25089456	−0.20067158	1.14985855
H	−2.14648242	0.00381026	1.67494905
H	−1.12881310	−0.24244974	3.09489270
H	−1.23941390	1.37502350	2.34886466
H	−2.21527604	0.12970467	−0.54829528

2(formaldehyde)

E (TPSSh/def2-TZVP) =	−898.01994185
$G - E$ (TPSSh/def2-TZVP) =	0.20045162
$H - E$ (TPSSh/def2-TZVP) =	0.26337977
E (DLPNO-CCSD(T)tight/def-TZVPP//TPSSh/def2-TZVP) =	−894.08017730
E (DLPNO-CCSD(T)tight/def-QZVPP//TPSSh/def2-TZVP) =	−894.11369411
E (DLPNO-CCSD(T)/CBS//TPSSh/def2-TZVP) =	−896.694575727

N	1.48332918	2.10964420	−1.67727318
C	2.15563748	1.33751395	−0.74903477
C	1.40312041	1.07741779	0.34390447
S	−0.14315983	1.81208862	0.19178081
C	0.25210043	2.44496618	−1.31829885
C	2.10672142	2.56269350	−2.93223017
N	−1.54648764	4.32150642	−2.82081357
C	−1.95296551	5.19838929	−1.71945190
C	−0.80994527	5.08196420	−3.83280673
C	−2.70990169	3.65516742	−3.41330189
O	1.86235164	5.09207514	−0.99940396
C	2.22995476	6.12783675	−0.49680183
H	−3.42499827	4.37306664	−3.84013828
H	3.16580418	1.01762664	−0.95271378
H	−0.41673430	3.09279520	−1.93467469
H	2.51408976	1.69872599	−3.45463458
H	1.34435206	3.04345438	−3.53697847
H	2.89159055	3.27633628	−2.68944154
H	1.67312142	0.49780981	1.21185796
H	−2.38034022	2.98244397	−4.20722763
H	−3.22472152	3.07050989	−2.64836518
H	−2.61029356	6.01188649	−2.05847500
H	−2.49120085	4.61646645	−0.96783655
H	−1.06421017	5.63445448	−1.25927644
H	−1.41607979	5.89115902	−4.26545111
H	0.08128150	5.52059577	−3.37934201
H	−0.50902309	4.41544778	−4.64441096
H	3.27424383	6.27657263	−0.17104159
H	1.53806008	6.97417012	−0.34023160

$TS_{2\text{-}IV}$(formaldehyde)

E (TPSSh/def2-TZVP) =	−897.99762355
$G - E$ (TPSSh/def2-TZVP) =	0.20615426
$H - E$ (TPSSh/def2-TZVP) =	0.26202271
E (DLPNO-CCSD(T)tight/def-TZVPP//TPSSh/def2-TZVP) =	−894.05924330
E (DLPNO-CCSD(T)tight/def-QZVPP//TPSSh/def2-TZVP) =	−894.09273816
E (DLPNO-CCSD(T)/CBS//TPSSh/def2-TZVP) =	−896.674507998

C	2.24399192	0.68992356	−1.25409738
C	1.30235128	0.23080841	−0.40503313
S	0.04632355	1.41110986	−0.29678832
C	0.76846660	2.51729482	−1.38590733
N	1.92537671	1.94170898	−1.77193957
C	2.83346631	2.60223323	−2.71765109
C	1.22491802	5.41296271	−0.27025957
O	0.72380970	5.98752109	−1.21518989
N	−1.48235059	5.12182333	−2.60825787
C	−0.94168585	4.44016437	−3.82328364
C	−2.39769178	4.21687030	−1.85102219
C	−2.13738722	6.42174796	−2.94191439
H	−3.26457388	3.99654612	−2.47246389
H	3.16350613	0.20530615	−1.54805834
H	−0.66380758	5.34516162	−1.99165569
H	3.82244241	2.70560734	−2.26985510
H	2.91005441	2.01379533	−3.63288326
H	2.42422372	3.58377953	−2.93936209
H	1.29838998	−0.70329713	0.13340563
H	−1.84809279	3.30791531	−1.61867528
H	−2.70996406	4.71973383	−0.93758080
H	−2.98191244	6.23180134	−3.60247363
H	−2.47818908	6.88750781	−2.01931321
H	−1.40802554	7.06186524	−3.43378114
H	−1.77298499	4.17629631	−4.47564441
H	−0.26593850	5.12520556	−4.33178540
H	−0.40597348	3.55486947	−3.48371763
H	2.26931374	5.61208456	0.01609902
H	0.66588530	4.68906199	0.33815162

IV(formaldehyde)

E (TPSSh/def2-TZVP) =	−898.04722724
$G - E$ (TPSSh/def2-TZVP) =	0.21079771
$H - E$ (TPSSh/def2-TZVP) =	0.26514641
E (DLPNO-CCSD(T)tight/def-TZVPP//TPSSh/def2-TZVP) =	−894.09447845
E (DLPNO-CCSD(T)tight/def-QZVPP//TPSSh/def2-TZVP) =	−894.12780470
E (DLPNO-CCSD(T)/CBS//TPSSh/def2-TZVP) =	−896.723512227

C	2.30037799	0.91295701	−1.53462264
C	1.28191852	0.36134607	−0.83632815
S	0.32018912	1.58983462	−0.12177929
C	1.30014056	2.81233898	−0.76545614
N	2.29645155	2.29291004	−1.48504546
C	3.32734470	3.11713599	−2.15182549
C	1.05258889	4.29674688	−0.60632418
O	0.90354576	4.89676619	−1.85535557
N	−1.60401725	4.98281886	−2.69060562
C	−1.69860130	4.11734180	−3.87443596
C	−2.59835519	4.58898327	−1.68384539
C	−1.78409641	6.39507959	−3.06684274
H	−3.62327199	4.68995400	−2.06413525
H	3.07030169	0.40537567	−2.09491901
H	−0.09099032	4.88555631	−2.14502780
H	4.11381792	3.34911702	−1.43357780
H	3.73638293	2.54074838	−2.97765400
H	2.85013524	4.02481894	−2.51324004
H	1.05297574	−0.68474934	−0.71151452
H	−2.43853240	3.54611136	−1.40130210
H	−2.49302857	5.22100238	−0.80061270
H	−2.77224194	6.56869361	−3.51150827
H	−1.67953450	7.02072157	−2.17994469
H	−1.01609565	6.67609776	−3.78788564
H	−2.68382312	4.19097626	−4.35268589
H	−0.93464133	4.40793636	−4.59642563
H	−1.53154729	3.07948585	−3.57912176
H	1.91649353	4.73334389	−0.09163476
H	0.18413612	4.42929468	0.04848848

II(acetaldehyde)

E (TPSSh/def2-TZVP) =	−762.36382223
$G - E$ (TPSSh/def2-TZVP) =	0.10373389
$H - E$ (TPSSh/def2-TZVP) =	0.14941548
E (DLPNO-CCSD(T)tight/def-TZVPP//TPSSh/def2-TZVP) =	−759.36865446
E (DLPNO-CCSD(T)tight/def-QZVPP//TPSSh/def2-TZVP) =	−759.39592206
E (DLPNO-CCSD(T)/CBS//TPSSh/def2-TZVP) =	−761.282407404

N	0.03855653	−0.03032982	0.26003309
C	−0.06722835	−0.06839281	1.64793975
C	1.04447506	−0.55601414	2.23474888
S	2.18325905	−0.95009420	0.99066852
C	1.17998884	−0.46391451	−0.31144077
C	1.53812076	0.38126027	−3.21013421
C	2.97748016	0.77224321	−3.04227869
C	−1.08043349	0.45925408	−0.55458401
O	0.62810057	1.17044973	−3.35044557
H	−0.97379012	0.27524496	2.12490121
H	1.22375068	−0.68776935	3.28986881
H	−1.92628579	−0.22503282	−0.46457230
H	−1.37654481	1.45098127	−0.20904880
H	−0.74765548	0.51548435	−1.58682392
H	1.34721370	−0.70769253	−3.23596032
H	3.54520339	0.42013116	−3.91135591
H	3.07853467	1.85290363	−2.94916996
H	3.38361161	0.27111850	−2.16091780

$TS_{II\text{-}III}$(acetaldehyde)

E (TPSSh/def2-TZVP) =	−762.35507320
$G - E$ (TPSSh/def2-TZVP) =	0.10666591
$H - E$ (TPSSh/def2-TZVP) =	0.15074101
E (DLPNO-CCSD(T)tight/def-TZVPP//TPSSh/def2-TZVP) =	−759.33298592
E (DLPNO-CCSD(T)tight/def-QZVPP//TPSSh/def2-TZVP) =	−759.36035802
E (DLPNO-CCSD(T)/CBS//TPSSh/def2-TZVP) =	−761.267562689

N	0.03336207	0.02990053	−0.02646430
C	0.06984213	0.05699725	1.35845287
C	1.30524585	−0.17532207	1.85078035
S	2.39499367	−0.42653246	0.53432618
C	1.19170685	−0.21120399	−0.65576684
C	1.39132532	0.43011179	−2.46063361
C	2.90652373	0.36192766	−2.62175845
C	−1.19875277	0.29527815	−0.78553462
O	0.81632458	1.55219921	−2.48765364
H	−0.83790772	0.25405989	1.90865690
H	1.60688683	−0.19974112	2.88532606
H	−1.62186447	−0.64800220	−1.13125217
H	−1.90648881	0.80074975	−0.13112061
H	−0.91492323	0.93176346	−1.63049285
H	0.89806695	−0.44374975	−2.93901855
H	3.14305884	0.60427176	−3.66172540
H	3.39150975	1.10314895	−1.98393653
H	3.29410543	−0.63747281	−2.40090177

III(acetaldehyde)

E (TPSSh/def2-TZVP) =	−762.37333001
$G - E$ (TPSSh/def2-TZVP) =	0.10886457
$H - E$ (TPSSh/def2-TZVP) =	0.15263813
E (DLPNO-CCSD(T)tight/def-TZVPP//TPSSh/def2-TZVP) =	−759.36051637
E (DLPNO-CCSD(T)tight/def-QZVPP//TPSSh/def2-TZVP) =	−759.38792566
E (DLPNO-CCSD(T)/CBS//TPSSh/def2-TZVP) =	−761.290857361

N	−0.00484246	−0.05658320	−0.13372590
C	0.05462614	0.03040574	1.25553357
C	1.27885290	0.16222842	1.77662160
S	2.50591814	0.21875154	0.52034256
C	1.22474342	0.33729257	−0.74703329
C	1.52381113	0.20936208	−2.16874133
C	2.91780141	0.03015144	−2.69881320
C	−1.25358993	0.23457716	−0.82375749
O	1.19948921	1.50649026	−1.59637848
H	−0.87045360	−0.04232940	1.80930693
H	1.54882896	0.20491942	2.81800705
H	−1.22024766	−0.17349791	−1.83371417
H	−2.06288366	−0.25965861	−0.28586212
H	−1.45513828	1.30857330	−0.88296090
H	0.73528880	−0.18160673	−2.81192589
H	3.02394141	0.54221341	−3.65844781
H	3.65559237	0.43740536	−2.00595502
H	3.12793671	−1.03175584	−2.85136211

2(acetaldehyde)

E (TPSSh/def2-TZVP) =	−937.37077378
$G-E$ (TPSSh/def2-TZVP) =	0.22876793
$H-E$ (TPSSh/def2-TZVP) =	0.29341923
E (DLPNO-CCSD(T)tight/def-TZVPP//TPSSh/def2-TZVP) =	−933.14091115
E (DLPNO-CCSD(T)tight/def-QZVPP//TPSSh/def2-TZVP) =	−933.17629509
E (DLPNO-CCSD(T)/CBS//TPSSh/def2-TZVP) =	−935.966212441

C	0.85605627	2.52133886	−1.26690850
N	1.67693008	2.32535166	−2.29410295
C	1.64659609	1.03483212	−2.79626432
C	0.82446532	0.22644167	−2.09595846
S	0.08417014	1.08986497	−0.79840707
C	2.51715938	3.39042374	−2.86492265
C	1.63675785	6.20814653	0.57936055
C	2.41211518	7.47055849	0.42300241
O	1.52184888	5.35593941	−0.28241232
N	−1.20479973	3.86548985	−2.30188161
C	−1.95439430	4.20001972	−1.09495505
C	−0.80762338	5.06821566	−3.03098961
C	−1.95577452	2.94650637	−3.15093340
H	−2.90302321	4.70922194	−1.32104318
H	2.25305858	0.78427790	−3.65275984
H	0.83209411	3.45144721	−0.71656302
H	3.55808687	3.07067597	−2.83615024
H	2.21135377	3.57121901	−3.89456139
H	2.38194226	4.28776335	−2.26707206
H	0.61907212	−0.81801189	−2.26430209
H	−2.18682195	3.28569754	−0.54271977
H	−1.35244926	4.85449090	−0.46187986
H	−1.67566982	5.66199423	−3.35485804
H	−0.17865876	5.69017209	−2.39250429
H	−0.24178354	4.78477064	−3.92054453
H	−2.90814862	3.37844979	−3.49317966
H	−1.36025109	2.68825367	−4.02865407
H	−2.17988064	2.03432109	−2.59306323
H	1.13869543	6.06371042	1.55716395
H	3.17630847	7.52115253	1.20624460
H	2.87160568	7.54086121	−0.56124875
H	1.74535533	8.32283238	0.59444545

$TS_{2\text{-}IV}$(acetaldehyde)

E (TPSSh/def2-TZVP) =	−937.35031558
$G - E$ (TPSSh/def2-TZVP) =	0.23491338
$H - E$ (TPSSh/def2-TZVP) =	0.29124265
E (DLPNO-CCSD(T)tight/def-TZVPP//TPSSh/def2-TZVP) =	−933.11496343
E (DLPNO-CCSD(T)tight/def-QZVPP//TPSSh/def2-TZVP) =	−933.15021003
E (DLPNO-CCSD(T)/CBS//TPSSh/def2-TZVP) =	−935.945981414

N	2.16914247	2.29284510	−1.81140730
C	2.51069356	0.95145098	−1.66962301
C	1.85867132	0.36339353	−0.64566456
S	0.83814575	1.53733121	0.10605182
C	1.27725577	2.80736353	−0.94613512
C	2.78205239	3.11648880	−2.86238800
C	1.14018857	5.28771958	−0.45149442
O	0.75561842	5.81894898	−1.50090963
N	−1.60597123	5.07953041	−2.38683540
C	−2.34462221	6.27238992	−2.88957185
C	−1.25934427	4.13731863	−3.48856589
C	−2.35334114	4.38275762	−1.30113008
H	−3.30067646	4.01569231	−1.69525759
H	3.22684518	0.50553456	−2.34414408
H	−0.66961284	5.42409386	−1.97358662
H	3.85699261	3.18934151	−2.69388695
H	2.59972863	2.66292348	−3.83727746
H	2.33200703	4.10431918	−2.81917660
H	1.93380959	−0.66096314	−0.31758255
H	−1.74513378	3.55312649	−0.94558740
H	−2.53604329	5.08880662	−0.49304468
H	−3.28818369	5.95221423	−3.33059012
H	−2.53027484	6.94494835	−2.05430608
H	−1.73062014	6.77262123	−3.63585560
H	−2.17555757	3.74772138	−3.93140782
H	−0.68006480	4.67551148	−4.23628361
H	−0.66575734	3.32869825	−3.06579275
C	2.55117994	5.39638736	0.02575830
H	0.41004389	4.92261485	0.28263884
H	2.82010508	4.55861979	0.66786018
H	3.24610704	5.49119887	−0.80742289
H	2.61575857	6.31315759	0.62587223

IV(acetaldehyde)

E (TPSSh/def2-TZVP) =	−937.38739163
$G - E$ (TPSSh/def2-TZVP) =	0.23592050
$H - E$ (TPSSh/def2-TZVP) =	0.29534688
E (DLPNO-CCSD(T)tight/def-TZVPP//TPSSh/def2-TZVP) =	−933.14814400
E (DLPNO-CCSD(T)tight/def-QZVPP//TPSSh/def2-TZVP) =	−933.18320411
E (DLPNO-CCSD(T)/CBS//TPSSh/def2-TZVP) =	−935.988919111

N	2.54676363	2.82669902	−2.03680070
C	2.83284028	1.54566581	−2.47363047
C	2.09683713	0.60017493	−1.84979533
S	1.03689091	1.33163960	−0.71516685
C	1.60823309	2.88475976	−1.08998089
C	3.26192848	4.00137746	−2.58313116
C	1.08135218	4.14527747	−0.41885139
O	0.76689075	5.10455224	−1.38479636
N	−1.72555257	4.89773656	−2.27189321
C	−1.64760893	5.34944501	−3.66929086
C	−2.28008878	3.54069326	−2.20168715
C	−2.53462695	5.83215307	−1.47385871
H	−3.56288967	5.89966288	−1.85197047
H	3.57917390	1.40589801	−3.24049574
H	−0.20267269	4.97632964	−1.71609298
H	4.21348198	4.10652108	−2.06275400
H	3.43531515	3.82270874	−3.64222347
H	2.63339182	4.87526002	−2.43874703
H	2.11815559	−0.46663175	−2.00379212
H	−2.56360338	5.49343379	−0.43721191
H	−2.07903452	6.82202242	−1.50842382
H	−2.64015783	5.39059334	−4.13626838
H	−1.20046746	6.34342782	−3.70266558
H	−1.02107108	4.66034108	−4.23818715
H	−3.29285163	3.49102496	−2.62292454
H	−1.63959058	2.85631840	−2.76216816
H	−2.32460007	3.21757356	−1.15986050
C	2.10245290	4.70074663	0.57401186
H	0.18931108	3.83199716	0.14011553
H	2.37349228	3.95560861	1.32459047
H	3.00440903	5.03372316	0.05916398
H	1.65606897	5.56151826	1.07194107

II(acrolein)

E (TPSSh/def2-TZVP) =	−800.46136743
$G - E$ (TPSSh/def2-TZVP) =	0.10793889
$H - E$ (TPSSh/def2-TZVP) =	0.15763962
E (DLPNO-CCSD(T)tight/def-TZVPP//TPSSh/def2-TZVP) =	−797.22024413
E (DLPNO-CCSD(T)tight/def-QZVPP//TPSSh/def2-TZVP) =	−797.24877426
E (DLPNO-CCSD(T)/CBS//TPSSh/def2-TZVP) =	−799.306142732

S	1.74440433	−0.24299453	−0.51561395
C	0.35637834	0.56223157	0.08292389
N	0.29643215	0.25783291	1.39310357
C	1.28126936	−0.56881389	1.92647189
C	2.18441279	−0.95328954	1.00197527
C	−0.78624063	0.77080319	2.24019173
C	−2.17613155	1.28478843	−0.97679684
O	−2.79970215	1.95959203	−0.17771330
H	−1.52292114	1.75822285	−1.72859506
H	1.25444692	−0.82420120	2.97597065
H	3.04468179	−1.58627104	1.15029258
H	−1.35043414	−0.06459991	2.65808019
H	−0.36467349	1.36864997	3.04995017
H	−1.43687345	1.38228699	1.62241616
C	−2.28415097	−0.19294233	−1.06663547
C	−3.18301735	−0.87688488	−0.35836277
H	−1.61385390	−0.68729754	−1.76182529
H	−3.27892521	−1.95297249	−0.43957441
H	−3.84862669	−0.35663158	0.32193301

$\mathbf{TS_{II\text{-}III}}$(acrolein)

E (TPSSh/def2-TZVP) =	−800.45362370
$G - E$ (TPSSh/def2-TZVP) =	0.11166377
$H - E$ (TPSSh/def2-TZVP) =	0.15664360
E (DLPNO-CCSD(T)tight/def-TZVPP//TPSSh/def2-TZVP) =	−797.19128661
E (DLPNO-CCSD(T)tight/def-QZVPP//TPSSh/def2-TZVP) =	−797.21988515
E (DLPNO-CCSD(T)/CBS//TPSSh/def2-TZVP) =	−799.294145672

S	1.60858476	−0.21296775	−0.60923886
C	0.05408393	0.05105371	0.03816479
N	0.15016851	0.04079823	1.37183849
C	1.41497999	−0.17396171	1.89788034
C	2.35555861	−0.33767965	0.94363091
C	−1.03009861	0.25424072	2.22596419
C	−1.45534860	0.88612870	−0.79987806
O	−1.80503954	1.86338081	−0.08641735
H	−0.90351471	1.08471924	−1.74389932
H	1.54584441	−0.18897625	2.96949996
H	3.41079723	−0.50834608	1.08202102
H	−1.56686141	−0.68719105	2.33935354
H	−0.69075451	0.61437567	3.19565033
H	−1.65787307	0.99278558	1.71860228
C	−2.33282202	−0.33181231	−0.93559845
C	−3.54235142	−0.39567368	−0.38790116
H	−1.93714321	−1.14910712	−1.53561290
H	−4.18264892	−1.26211614	−0.50565066
H	−3.91648643	0.44554809	0.18560190

III(acrolein)

E (TPSSh/def2-TZVP) =	−800.45992015
$G - E$ (TPSSh/def2-TZVP) =	0.11266780
$H - E$ (TPSSh/def2-TZVP) =	0.15794405
E (DLPNO-CCSD(T)tight/def-TZVPP//TPSSh/def2-TZVP) =	−797.19469070
E (DLPNO-CCSD(T)tight/def-QZVPP//TPSSh/def2-TZVP) =	−797.22358642
E (DLPNO-CCSD(T)/CBS//TPSSh/def2-TZVP) =	−799.301471675

S	1.60069496	0.09984973	−0.64724963
C	0.02317872	0.14901606	0.00975772
N	0.07905508	−0.01834275	1.34351142
C	1.35308619	−0.15060047	1.85455424
C	2.31738984	−0.10865869	0.91117528
C	−1.11463566	0.10945844	2.18811812
C	−1.20639650	0.61274465	−0.77011203
O	−1.48018892	1.78122547	−0.18646189
H	−0.85139659	0.68032590	−1.83059407
H	1.47733268	−0.26325517	2.92067187
H	3.38306045	−0.17202242	1.05359206
H	−1.73882393	−0.77644764	2.08421522
H	−0.78839628	0.23008509	3.21936494
H	−1.64942152	0.98602824	1.81116101
C	−2.29215051	−0.45695723	−0.78158081
C	−3.51378131	−0.20067534	−0.33004489
H	−2.03724678	−1.41853960	−1.22602749
H	−4.31338680	−0.93112319	−0.37614977
H	−3.72629811	0.78077991	0.08192871

2(acrolein)

E (TPSSh/def2-TZVP) =	−975.46858673
$G-E$ (TPSSh/def2-TZVP) =	0.23167371
$H-E$ (TPSSh/def2-TZVP) =	0.29946752
E (DLPNO-CCSD(T)tight/def-TZVPP//TPSSh/def2-TZVP) =	−971.00129002
E (DLPNO-CCSD(T)tight/def-QZVPP//TPSSh/def2-TZVP) =	−971.03790670
E (DLPNO-CCSD(T)/CBS//TPSSh/def2-TZVP) =	−973.993888136

S	0.39005872	1.13920394	−1.38851455
C	1.42545222	1.20094423	−2.71519887
N	2.43581747	0.35451593	−2.58213060
C	2.42204563	−0.36553879	−1.40318249
C	1.35887807	−0.05846260	−0.62620167
C	3.52895070	0.25171336	−3.56267653
N	1.08577057	3.19261823	−4.94980859
C	0.07975614	2.78067216	−5.93242820
C	0.61922899	4.35045651	−4.18321240
C	2.36192549	3.49397880	−5.60183825
O	3.69725398	3.05794561	−2.12705770
C	3.97847137	4.06125427	−1.48994975
C	4.95047112	5.08111483	−1.91181333
C	5.67377245	4.95335019	−3.02728274
H	0.44344702	5.22760524	−4.82265022
H	3.20833565	−1.07674511	−1.20303632
H	1.30431157	1.88434805	−3.58728871
H	3.71423849	−0.79991927	−3.77380823
H	3.22506097	0.76983359	−4.46686295
H	4.41297133	0.72889548	−3.14415868
H	1.09783247	−0.47331690	0.33388146
H	−0.31807814	4.10315517	−3.67945730
H	1.36689493	4.61301589	−3.43254434
H	2.28216798	4.34391592	−6.29540552
H	3.10840662	3.73081727	−4.84159936
H	2.69560681	2.62367290	−6.17162640
H	−0.13061583	3.57184150	−6.66663884
H	0.43267438	1.89750670	−6.46828143
H	−0.85122019	2.53143675	−5.41936981
H	3.48174790	4.24965769	−0.52009760
H	5.06313379	5.94334041	−1.26315365
H	6.39287784	5.70379322	−3.33075608
H	5.56206148	4.07991480	−3.66012638

$TS_{2\text{-}IV}$(acrolein)

E (TPSSh/def2-TZVP) =	−975.44989850
$G-E$ (TPSSh/def2-TZVP) =	0.23721265
$H-E$ (TPSSh/def2-TZVP) =	0.30075632
E (DLPNO-CCSD(T)tight/def-TZVPP//TPSSh/def2-TZVP) =	−970.98182871
E (DLPNO-CCSD(T)tight/def-QZVPP//TPSSh/def2-TZVP) =	−971.01832841
E (DLPNO-CCSD(T)/CBS//TPSSh/def2-TZVP) =	−973.97689301

C	1.96054518	1.12685398	−2.89374437
N	3.13332330	0.48930338	−2.69605670
C	3.19405767	−0.48906719	−1.70802851
C	2.01629069	−0.64680850	−1.07048780
S	0.87403095	0.45275231	−1.75681041
C	4.31578419	0.79141751	−3.51054220
C	3.03190429	3.96172226	−2.09970078
C	4.18579531	3.89938938	−1.20197944
O	3.09072809	4.31720960	−3.27463022
N	1.07134042	3.83220479	−5.04700377
C	1.00056835	5.14517332	−5.75180805
C	1.49993454	2.73061957	−5.95982232
C	−0.21818736	3.49179321	−4.37640986
H	0.25306117	5.08380272	−6.54138968
H	4.11853007	−1.01985916	−1.53381157
H	1.80802273	3.92903133	−4.29897130
H	5.17207076	0.97652590	−2.86163918
H	4.53567495	−0.04630315	−4.17373800
H	4.09864499	1.68137791	−4.09388178
H	1.78666541	−1.33182059	−0.27013486
H	0.72542923	5.91348381	−5.03190098
H	1.97858386	5.36657101	−6.17405432
H	0.74586900	2.60580588	−6.73611117
H	2.45528060	3.00233339	−6.40533585
H	1.59924848	1.83209095	−5.35209569
H	−0.99472414	3.40026831	−5.13484061
H	−0.07283185	2.55127547	−3.85026551
H	−0.46974192	4.28796516	−3.67792715
H	2.06565884	3.66483999	−1.66500118
H	3.99688171	3.55103222	−0.19249287
C	5.40864437	4.26759874	−1.59805010
H	6.25873052	4.23051010	−0.92812387
H	5.57761260	4.62490139	−2.60778788

IV(acrolein)

E (TPSSh/def2-TZVP) =	−975.48419666
$G - E$ (TPSSh/def2-TZVP) =	0.23977849
$H - E$ (TPSSh/def2-TZVP) =	0.30065825
E (DLPNO-CCSD(T)tight/def-TZVPP//TPSSh/def2-TZVP) =	−971.00331791
E (DLPNO-CCSD(T)tight/def-QZVPP//TPSSh/def2-TZVP) =	−971.03969870
E (DLPNO-CCSD(T)/CBS//TPSSh/def2-TZVP) =	−974.01478376

C	2.68624848	0.98123786	−1.93981065
N	3.91507905	0.47024745	−2.02915854
C	4.05830596	−0.75941141	−1.41530123
C	2.91606561	−1.19802631	−0.84092466
S	1.65679787	−0.05951200	−1.08785802
C	5.05011541	1.14599937	−2.69302948
C	2.23944489	2.31779530	−2.49798048
C	2.82623138	3.42905267	−1.64267246
O	2.60017929	2.35232888	−3.84097106
N	1.11945580	3.98455321	−5.25309841
C	2.05343688	4.92847376	−5.88569170
C	0.44704618	3.16219089	−6.27089099
C	0.13588394	4.70773220	−4.43366244
H	1.53222706	5.61254099	−6.56709122
H	5.01736426	−1.25293501	−1.44312423
H	1.98566909	3.01964117	−4.36803161
H	5.51156898	1.83272568	−1.98420064
H	5.75890086	0.38008646	−2.99786781
H	4.66322565	1.68737319	−3.55089071
H	2.75327390	−2.11955436	−0.30587014
H	2.55130388	5.51562310	−5.11298258
H	2.80308512	4.37189437	−6.44934345
H	−0.14183056	3.77923005	−6.96113062
H	1.19592797	2.61058925	−6.84004205
H	−0.21780014	2.45075140	−5.77937341
H	−0.45229914	5.41065701	−5.03715301
H	−0.54705900	3.99304038	−3.97135597
H	0.65527412	5.26450634	−3.65228625
H	1.14554276	2.34026293	−2.36858198
H	2.49870534	3.45057952	−0.60650898
C	3.66357263	4.34168023	−2.11832565
H	4.04278058	5.13822425	−1.49047805
H	3.98041291	4.32001018	−3.15443651

II(benzaldehyde)

E (TPSSh/def2-TZVP) =	−954.19932633
$G - E$ (TPSSh/def2-TZVP) =	0.15403768
$H - E$ (TPSSh/def2-TZVP) =	0.20497300
E (DLPNO-CCSD(T)tight/def-TZVPP//TPSSh/def2-TZVP) =	−949.94816084
E (DLPNO-CCSD(T)tight/def-QZVPP//TPSSh/def2-TZVP) =	−949.98244457
E (DLPNO-CCSD(T)/CBS//TPSSh/def2-TZVP) =	−952.74128576

N	0.47886098	0.26642108	−0.62067343
C	0.85217303	0.62035771	0.62593526
S	2.07338007	1.80439997	0.41490891
C	2.03480323	1.77775548	−1.31662617
C	1.10558346	0.88007100	−1.70237311
C	−0.56378538	−0.74230888	−0.84939341
C	−1.07120585	−1.27141157	2.86687798
O	−1.77804503	−1.95570326	2.14572351
C	−1.10518086	−1.31446032	4.34182324
C	−1.97734781	−2.16738910	5.02582094
C	−1.98240888	−2.18128246	6.41165041
C	−1.11817271	−1.34526023	7.12122626
C	−0.24807431	−0.49444951	6.44571653
C	−0.24234747	−0.47966175	5.05607727
H	0.82078776	0.61042504	−2.70918437
H	2.65956941	2.38679819	−1.95037294
H	−1.38635558	−0.29627117	−1.41156131
H	−0.14709795	−1.57579012	−1.41795974
H	−0.92416060	−1.09638287	0.11309623
H	−0.34499479	−0.56004181	2.42754943
H	0.42842483	0.17658414	4.51077783
H	0.42063313	0.15238158	7.00155687
H	−1.12523476	−1.35918233	8.20534953
H	−2.65612529	−2.84000404	6.94748501
H	−2.63703566	−2.80529079	4.44932128

TS$_{\text{II-III}}$(benzaldehyde)

E (TPSSh/def2-TZVP) =	−954.19057927
$G - E$ (TPSSh/def2-TZVP) =	0.15599152
$H - E$ (TPSSh/def2-TZVP) =	0.20575845
E (DLPNO-CCSD(T)tight/def-TZVPP//TPSSh/def2-TZVP) =	−949.90814525
E (DLPNO-CCSD(T)tight/def-QZVPP//TPSSh/def2-TZVP) =	−949.94249977
E (DLPNO-CCSD(T)/CBS//TPSSh/def2-TZVP) =	−952.727332899

C	4.55112905	1.18093924	2.20227470
C	4.51753371	0.28452755	1.13819317
C	5.14954697	−0.95219824	1.25853411
C	5.79776428	−1.29901590	2.43889249
C	5.82044438	−0.40407660	3.50614431
C	5.19991727	0.83683282	3.38343336
C	3.79249267	0.66549066	−0.13798015
O	3.37826481	1.84522666	−0.30134822
C	2.23150968	−0.42766315	0.10252226
S	1.81202907	−1.29467535	1.50758842
C	0.12217490	−1.25155545	1.15364758
C	−0.07653160	−0.58297483	−0.00289281
N	1.10900330	−0.13648059	−0.56508100
C	1.14998390	0.64560334	−1.81052224
H	−1.01234849	−0.37116115	−0.49830019
H	−0.62671671	−1.69039927	1.79296933
H	1.37991657	−0.01518804	−2.64670073
H	0.17628272	1.10823326	−1.96155123
H	1.92946506	1.40263622	−1.68132634
H	4.19837936	0.09989053	−1.00227941
H	5.12877670	−1.64464627	0.42126079
H	6.28878785	−2.26192788	2.52694174
H	6.32723837	−0.67061558	4.42685446
H	5.22641112	1.53787952	4.21056116
H	4.06543206	2.14110648	2.07320147

III(benzaldehyde)

E (TPSSh/def2-TZVP) =	−954.19788475
$G - E$ (TPSSh/def2-TZVP) =	0.15693390
$H - E$ (TPSSh/def2-TZVP) =	0.20726027
E (DLPNO-CCSD(T)tight/def-TZVPP//TPSSh/def2-TZVP) =	−949.91339823
E (DLPNO-CCSD(T)tight/def-QZVPP//TPSSh/def2-TZVP) =	−949.94801808
E (DLPNO-CCSD(T)/CBS//TPSSh/def2-TZVP) =	−952.735593433

C	0.07684377	−0.48146120	−0.26732370
C	0.05833184	−0.86711024	1.02430229
S	1.63025858	−0.68604249	1.73100203
C	2.25687139	−0.02531218	0.28305524
N	1.31882008	−0.04302721	−0.68518921
C	3.55876072	0.73123007	0.18629157
O	3.02291403	1.96454282	0.27497562
C	1.56607130	0.58933402	−1.98295864
H	−0.74176624	−0.48283962	−0.97048459
H	−0.77965081	−1.24399059	1.58604956
H	2.23163085	−0.03027179	−2.58380960
H	0.61366202	0.70848474	−2.49472493
H	2.02502055	1.55841965	−1.77295586
C	4.50526318	0.30689165	1.30799830
H	4.05664026	0.47735024	−0.77883413
C	5.16158859	−0.92365980	1.28303711
C	5.99296055	−1.29739087	2.33241113
C	6.17886244	−0.43518788	3.41167786
C	5.53154576	0.79636064	3.43401218
C	4.69382868	1.16618296	2.38487450
H	5.02100254	−1.59170649	0.43739563
H	6.50000020	−2.25577551	2.30950109
H	6.83086482	−0.72304687	4.22882991
H	5.68059156	1.47007459	4.27078591
H	4.17043737	2.11498436	2.36529471

2(benzaldehyde)

E (TPSSh/def2-TZVP) =	−1129.21003927
$G - E$ (TPSSh/def2-TZVP) =	0.27957910
$H - E$ (TPSSh/def2-TZVP) =	0.34951809
E (DLPNO-CCSD(T)tight/def-TZVPP//TPSSh/def2-TZVP) =	−1123.71371019
E (DLPNO-CCSD(T)tight/def-QZVPP//TPSSh/def2-TZVP) =	−1123.75611214
E (DLPNO-CCSD(T)/CBS//TPSSh/def2-TZVP) =	−1127.42890804

C	4.16226311	5.43279136	−0.69607538
C	2.97745597	5.75341710	−0.02107742
C	2.91916698	5.65609562	1.37228967
C	4.02381174	5.20846624	2.08637222
C	5.19748160	4.88650805	1.40911916
C	5.27034228	5.00867139	0.01971667
C	1.77382333	6.16339145	−0.75687030
O	1.62389758	6.04935988	−1.96063033
N	−1.63049592	4.22373434	−2.48185655
C	−2.71375101	3.23700448	−2.48223700
C	−1.94829380	5.34261061	−1.58986613
C	−1.36886892	4.71475638	−3.83814805
C	0.79754575	2.98708247	−1.25780078
N	1.96145179	2.82111334	−1.87424305
C	2.96859911	2.33259219	−1.06434711
C	2.55067801	2.11442259	0.20327393
S	0.89361342	2.53413457	0.36175189
C	2.18036654	3.20304293	−3.28279570
H	−3.65987446	3.66179724	−2.84704465
H	3.95218751	2.17146226	−1.47546835
H	−0.11348799	3.41349235	−1.75389198
H	2.97178646	2.57815325	−3.68931694
H	1.25739907	3.04097031	−3.83196306
H	2.45110558	4.25775000	−3.31178490
H	3.12487283	1.73973672	1.03447748
H	−2.44320774	2.39594228	−3.12373170
H	−2.87305736	2.86801798	−1.46662506
H	−2.87398276	5.85591308	−1.88696592
H	−2.07630800	4.97334259	−0.56922624
H	−1.12937057	6.06245232	−1.61381333
H	−2.24121834	5.23035339	−4.26470094
H	−0.52931412	5.41136046	−3.81362127
H	−1.12297334	3.87297621	−4.48982447
H	0.95871742	6.57363296	−0.12859174
H	2.00485933	5.92975839	1.88933423
H	3.98031568	5.12995945	3.16585497
H	6.06716568	4.55839514	1.96626364
H	6.19843098	4.78578587	−0.49322001
H	4.20147760	5.55200272	−1.77265247

$\mathbf{TS_{2\text{-}IV}}$(benzaldehyde)

E (TPSSh/def2-TZVP) =	−1129.18957367
$G-E$ (TPSSh/def2-TZVP) =	0.28398110
$H-E$ (TPSSh/def2-TZVP) =	0.34686256
E (DLPNO-CCSD(T)tight/def-TZVPP//TPSSh/def2-TZVP) =	−1123.68292838
E (DLPNO-CCSD(T)tight/def-QZVPP//TPSSh/def2-TZVP) =	−1123.72518670
E (DLPNO-CCSD(T)/CBS//TPSSh/def2-TZVP) =	−1127.40556918

N	2.47055984	2.43331119	−1.69541413
C	2.91896436	1.13024398	−1.51131429
C	2.28250091	0.50713159	−0.49809671
S	1.13426108	1.59584267	0.19496814
C	1.51354050	2.87252164	−0.86729242
C	3.05054132	3.29856710	−2.73235456
C	1.18021629	5.06193465	−0.50258674
O	0.82497947	5.60097974	−1.58460717
N	−1.53850151	5.09343462	−2.46214370
C	−2.02417081	6.31708992	−3.15594770
C	−1.34910720	3.96227883	−3.40923385
C	−2.43503571	4.71205683	−1.33796457
H	−3.43100565	4.49125683	−1.72268134
H	3.69204380	0.73485097	−2.15343491
H	−0.53836456	5.32534629	−2.04031056
H	4.08363057	3.53241949	−2.47568230
H	3.01572978	2.78224459	−3.69174950
H	2.46401638	4.21186058	−2.77351109
H	2.42736413	−0.50252920	−0.14886323
H	−2.02381940	3.83140755	−0.84752895
H	−2.48532065	5.54034471	−0.63334555
H	−2.99951519	6.12164070	−3.60235909
H	−2.10026433	7.12301009	−2.42840675
H	−1.30604131	6.58798952	−3.92744300
H	−2.30997665	3.67679185	−3.83792876
H	−0.66887807	4.27933049	−4.19748205
H	−0.91594867	3.12559234	−2.86352250
C	2.52677023	5.28959446	0.06225399
H	0.42431532	4.75547458	0.23023243
C	2.83106622	4.81921522	1.34315230
C	4.08400552	5.04843386	1.89138783
C	5.04065756	5.75373890	1.16299645
C	4.73721548	6.23816968	−0.10750137
C	3.48300401	6.00973576	−0.65790178
H	2.08262701	4.26941602	1.90460100
H	4.31694557	4.68494123	2.88482583
H	6.01906862	5.93555696	1.59141400
H	5.47674796	6.80372128	−0.66166121
H	3.22179548	6.40183952	−1.63323072

IV(benzaldehyde)

E (TPSSh/def2-TZVP) =	−1129.22222003
$G - E$ (TPSSh/def2-TZVP) =	0.28500305
$H - E$ (TPSSh/def2-TZVP) =	0.35094444
E (DLPNO-CCSD(T)tight/def-TZVPP//TPSSh/def2-TZVP) =	−1123.72019208
E (DLPNO-CCSD(T)tight/def-QZVPP//TPSSh/def2-TZVP) =	−1123.76235781
E (DLPNO-CCSD(T)/CBS//TPSSh/def2-TZVP) =	−1127.44791889

N	3.18432265	2.04456122	−1.97031814
C	3.89351787	0.86494392	−1.84205332
C	3.41507521	0.06563945	−0.86341127
S	2.05906690	0.80098778	−0.11227503
C	2.17365213	2.16674710	−1.10859712
C	3.56362112	3.07140405	−2.96217712
C	1.24452826	3.35231668	−0.96825541
O	0.73492798	3.64171026	−2.23126873
N	−1.27149623	5.33534943	−2.05753437
C	−0.92445751	6.50039841	−2.88406555
C	−2.44957466	4.64533390	−2.60423529
C	−1.50695395	5.74656264	−0.66579971
H	−2.33364013	6.46483074	−0.59255200
H	4.72677143	0.67849980	−2.50160293
H	−0.05458205	4.32060803	−2.13649490
H	4.22240586	3.79478862	−2.48272300
H	4.07908916	2.57063397	−3.77800668
H	2.65447916	3.55202025	−3.31040262
H	3.77692305	−0.90386844	−0.56188918
H	−1.75591128	4.86973724	−0.06566724
H	−0.60250156	6.20713634	−0.26612566
H	−1.74531306	7.22817650	−2.91752310
H	−0.04181824	6.98715706	−2.46734428
H	−0.70465081	6.17048420	−3.90025470
H	−3.33025038	5.30015169	−2.61523648
H	−2.23692649	4.32147383	−3.62358451
H	−2.67030730	3.76830121	−1.99442129
C	1.99905683	4.50029274	−0.29286917
H	0.45441138	3.02349102	−0.27595733
C	2.25571062	4.44898176	1.07732199
C	2.93018980	5.49008114	1.70292199
C	3.35117913	6.59134938	0.96137840
C	3.09116709	6.64778303	−0.40386317
C	2.41889647	5.60320608	−1.03225491
H	1.91466020	3.60099382	1.66394694
H	3.11862182	5.44804356	2.76882678
H	3.87216995	7.40603466	1.44926853
H	3.40731039	7.50841836	−0.98137895
H	2.19664719	5.64976655	−2.09094648

5.3 Reactions: Imidazole + Base + Aldehyde

5.3.1 DABCO

I

E (TPSSh/def2-TZVP) =	−650.90392737
$G - E$ (TPSSh/def2-TZVP) =	0.28187263
$H - E$ (TPSSh/def2-TZVP) =	0.33993274
E (DLPNO-CCSD(T)tight/def-TZVPP//TPSSh/def2-TZVP) =	−646.57277666
E (DLPNO-CCSD(T)tight/def-QZVPP//TPSSh/def2-TZVP) =	−646.60133715
E (DLPNO-CCSD(T)/CBS//TPSSh/def2-TZVP) =	−649.73530397

C	−1.98406710	0.71688798	1.31575847
N	−0.67950896	1.18653950	1.83234387
C	0.38959389	0.29977172	1.32197748
C	0.38806742	0.32465051	−0.23985794
N	−0.66905950	1.21634249	−0.71826460
C	−1.96825732	0.73818624	−0.24606327
C	−0.43692038	2.56167329	1.34367974
C	−0.43433779	2.57070462	−0.21887833
H	1.35152012	0.68229866	−0.63301740
H	0.20665147	−0.71375125	1.71471148
H	1.34818314	0.65026343	1.73712081
H	−2.15695713	−0.30124975	1.70229553
H	−2.76913613	1.37062924	1.72834158
H	−2.14324348	−0.26621776	−0.65999698
H	−2.75096917	1.40234077	−0.64215202
H	0.53169736	2.89903574	1.74856042
H	−1.22413253	3.21359056	1.75593829
H	−1.22097749	3.23088874	−0.61371037
H	0.52911327	2.92742289	−0.61280110
H	0.21221112	−0.67945210	−0.65412757
H	−0.67907495	1.16278074	3.72711917
C	−0.67303647	1.12767538	4.85125863
N	−1.15904004	0.13307953	5.61165413
C	−0.96965534	0.44422600	6.94709544
C	−0.35216868	1.66185881	6.98632938
N	−0.17829184	2.06672403	5.67403475
C	0.44741480	3.32237472	5.24778835
H	−0.02715480	2.26679193	7.82789186
H	−1.28667555	−0.21783762	7.74770619
C	−1.78339345	−1.09213842	5.10330997
H	1.49359130	3.34327167	5.58050793
H	0.40555328	3.37883362	4.15487205
H	−0.09976389	4.16927745	5.68192059
H	−2.80147656	−1.17812669	5.50485128
H	−1.82273303	−1.03430983	4.01031594
H	−1.18667861	−1.96231984	5.40713025

2(formaldehyde)

E (TPSSh/def2-TZVP) =	−765.47063461
$G - E$ (TPSSh/def2-TZVP) =	0.30337750
$H - E$ (TPSSh/def2-TZVP) =	0.37252407
E (DLPNO-CCSD(T)tight/def-TZVPP//TPSSh/def2-TZVP) =	−760.50017575
E (DLPNO-CCSD(T)tight/def-QZVPP//TPSSh/def2-TZVP) =	−760.53376760
E (DLPNO-CCSD(T)/CBS//TPSSh/def2-TZVP) =	−764.140007343

N	1.75597334	2.61489029	−1.13920773
C	1.66324913	1.38063017	−1.92333731
C	0.80849524	0.32767245	−1.15812296
N	0.33696155	0.89437277	0.12331604
C	1.51457690	1.27687651	0.93066345
C	2.37526820	2.31707576	0.15500856
C	0.40878463	3.14942991	−0.91942564
C	−0.46174209	2.10560470	−0.15839166
O	1.74255737	−2.50949124	1.26494235
C	2.84173364	−2.93878337	1.52286251
H	3.73612793	−2.29049127	1.51035742
C	−1.14038775	−1.31039551	1.63364039
N	−1.44055304	−2.54209514	1.21455618
C	−2.04859021	−3.23678179	2.24162495
C	−2.11836844	−2.39338265	3.30307834
N	−1.54873950	−1.19974513	2.90226071
C	−1.12663898	−3.08142419	−0.11010249
C	−1.40533419	0.00162424	3.72744228
H	−0.78325805	−0.22417216	4.59258047
H	−2.37649270	−4.25531842	2.12760921
H	−0.62900863	−0.52014571	1.05343167
H	−2.38857243	0.33859389	4.05287138
H	−0.93233723	0.77313657	3.12570312
H	−0.39829120	−3.88310365	−0.00511070
H	−2.04016545	−3.45193168	−0.57286609
H	−0.70164467	−2.28326494	−0.71180017
H	−2.52055450	−2.53312420	4.29135560
H	3.00583227	−3.99953375	1.78333950
H	1.38546353	−0.57213980	−0.92888461
H	−0.07221435	0.03450802	−1.73772233
H	1.15233230	1.68974533	1.87716232
H	2.07386605	0.36462090	1.15149082
H	−1.33378425	1.80318029	−0.74418759
H	−0.81901104	2.50225316	0.79647126
H	2.46572561	3.25103262	0.71355685
H	3.38274529	1.93752960	−0.02896742
H	−0.02546821	3.39124532	−1.89158502
H	0.49883827	4.07791984	−0.35189313
H	2.67577321	1.01241311	−2.10207550
H	1.21867446	1.62329316	−2.89064204

$\mathbf{TS_{2\text{-}IV}}$(formaldehyde)

E (TPSSh/def2-TZVP) =	−765.44231014
$G-E$ (TPSSh/def2-TZVP) =	0.30973120
$H-E$ (TPSSh/def2-TZVP) =	0.37366078
E (DLPNO-CCSD(T)tight/def-TZVPP//TPSSh/def2-TZVP) =	−760.45803242
E (DLPNO-CCSD(T)tight/def-QZVPP//TPSSh/def2-TZVP) =	−760.49149291
E (DLPNO-CCSD(T)/CBS//TPSSh/def2-TZVP) =	−764.108374051

N	1.17805197	2.72415705	−1.10847303
C	1.81348762	1.75483621	−2.00376467
C	1.76862634	0.33140399	−1.38976453
N	1.02159180	0.42988869	−0.08703576
C	1.75939447	1.33459018	0.85931875
C	1.86661187	2.72646590	0.18243319
C	−0.22910194	2.37468091	−0.91782177
C	−0.36576467	0.94644468	−0.32096836
O	2.14917941	−1.95147222	0.55548351
C	1.72138568	−2.38446021	1.62450103
H	1.98729861	−1.92020522	2.58613266
C	−0.52034431	−1.62201580	1.95671901
N	−1.48259464	−2.31829197	1.29325685
C	−2.65957751	−2.39665375	2.01550264
C	−2.43765178	−1.73504909	3.17652486
N	−1.13344875	−1.27568277	3.11983002
C	−1.27857481	−2.93448712	−0.01317583
C	−0.48570315	−0.52897409	4.19092408
H	−0.46485702	−1.12346989	5.10486390
H	−3.53503392	−2.90847238	1.65312018
H	1.00495855	−0.51224251	0.34333023
H	−1.02093247	0.40268971	4.38015212
H	0.53326055	−0.31066744	3.88056200
H	−1.50022931	−4.00074449	0.03939858
H	−1.93044917	−2.47224891	−0.75666174
H	−0.23686667	−2.79810691	−0.29627027
H	−3.08276645	−1.55830879	4.02047535
H	1.25849247	−3.37972365	1.69993121
H	2.74613743	−0.08180830	−1.15101406
H	1.23117119	−0.38778241	−2.00672141
H	1.18392478	1.35062215	1.78455645
H	2.72249503	0.86257346	1.04763854
H	−0.85444649	0.24754357	−0.99913996
H	−0.87998011	0.92016950	0.63863197
H	1.41958128	3.49388561	0.81489390
H	2.91089356	2.99148547	0.01629716
H	−0.73646121	2.43376368	−1.88093546
H	−0.68087850	3.11116070	−0.25262967
H	2.84500796	2.06360203	−2.17380176
H	1.29269230	1.77765442	−2.96136190

IV(formaldehyde)

E (TPSSh/def2-TZVP) =	−765.49730612
$G - E$ (TPSSh/def2-TZVP) =	0.31271710
$H - E$ (TPSSh/def2-TZVP) =	0.37526783
E (DLPNO-CCSD(T)tight/def-TZVPP//TPSSh/def2-TZVP) =	−760.51278390
E (DLPNO-CCSD(T)tight/def-QZVPP//TPSSh/def2-TZVP) =	−760.54614381
E (DLPNO-CCSD(T)/CBS//TPSSh/def2-TZVP) =	−764.168488315

N	1.31421821	2.97606851	−1.04296692
C	1.71239937	2.01872785	−2.08023963
C	1.66661738	0.57054554	−1.51688543
N	1.22150373	0.59990449	−0.10001814
C	2.17452070	1.42114988	0.68259349
C	2.22892208	2.86153933	0.09569650
C	−0.04877606	2.67311360	−0.60402700
C	−0.11743259	1.22550833	−0.03424986
O	1.26616475	−1.97770113	0.55028398
C	0.91908654	−2.21380221	1.88183375
H	1.37518092	−1.50150634	2.57921139
C	−0.57447806	−2.17500964	2.07227288
N	−1.46684061	−2.87709808	1.35523091
C	−2.74231356	−2.56194759	1.77347905
C	−2.62143194	−1.64653010	2.76726987
N	−1.27024275	−1.41400326	2.93740513
C	−1.13414072	−3.86154936	0.31504814
C	−0.70041676	−0.50085522	3.93335957
H	−0.27680446	−1.06575440	4.76296297
H	−3.61382975	−3.01014077	1.32910819
H	1.22663970	−0.97147493	0.35080057
H	−1.50037496	0.13844676	4.29825591
H	0.06806029	0.11444732	3.46986594
H	−1.05573382	−4.85115028	0.76541771
H	−1.93079847	−3.85574736	−0.42552139
H	−0.19220200	−3.56741061	−0.14093420
H	−3.36501413	−1.14179230	3.35878725
H	1.27951405	−3.20905255	2.16323310
H	2.64551629	0.08815373	−1.54223225
H	0.96667839	−0.06114923	−2.06825344
H	1.83558765	1.42474210	1.72283071
H	3.14682611	0.92574270	0.64638927
H	−0.80719257	0.59576232	−0.60201574
H	−0.43761160	1.22201097	1.01239988
H	1.94062707	3.60325223	0.84341946
H	3.23516171	3.10636758	−0.24890996
H	−0.71825950	2.78998161	−1.45840578
H	−0.33655023	3.40728500	0.15141959
H	2.71770941	2.27844124	−2.41656431
H	1.03485019	2.13366425	−2.92847816

2(acetaldehyde)

E (TPSSh/def2-TZVP) =	−804.82127047
$G - E$ (TPSSh/def2-TZVP) =	0.32993036
$H - E$ (TPSSh/def2-TZVP) =	0.40215476
E (DLPNO-CCSD(T)tight/def-TZVPP//TPSSh/def2-TZVP) =	−799.56554713
E (DLPNO-CCSD(T)tight/def-QZVPP//TPSSh/def2-TZVP) =	−799.60102575
E (DLPNO-CCSD(T)/CBS//TPSSh/def2-TZVP) =	−803.410836332

C	0.80690825	2.51860589	−1.59537586
N	2.07188522	2.50540315	−2.02057360
C	2.74573720	1.46670342	−1.41101362
C	1.85314621	0.83348004	−0.60795376
N	0.65179604	1.50363121	−0.74011622
C	2.67120867	3.51605474	−2.89300041
C	−0.59187496	1.17395455	−0.04272476
C	2.35405741	5.42201223	1.01574522
O	2.05154782	4.60447829	0.17129916
C	1.46451683	5.85881926	2.13440006
N	−1.29494420	4.62111269	−2.35421894
C	−0.76936812	5.94381766	−1.95571347
H	−1.36049843	1.87140273	−0.36502355
H	3.78603610	1.26980097	−1.60230336
H	0.03530611	3.25395193	−1.88095092
H	−0.43887254	1.26601513	1.03186489
H	−0.89031857	0.15620679	−0.29065687
H	3.21287815	4.23486978	−2.28006659
H	3.34147595	3.02602525	−3.59652296
H	1.87492866	4.01854494	−3.43618541
H	1.96179569	−0.02691998	0.02893905
H	3.35309007	5.90087479	0.98449486
H	1.96604253	5.67290928	3.09005517
H	1.31518697	6.94230520	2.07407372
H	0.50570894	5.34322055	2.10499636
C	−1.80760822	7.05226613	−2.29810655
H	−0.55055136	5.90526131	−0.88569994
H	0.17548604	6.10136865	−2.48347994
C	−1.57234723	4.63596053	−3.80597021
C	−2.56161104	4.37992709	−1.63298750
C	−3.59190558	5.49679322	−1.97631993
H	−2.93148188	3.39261537	−1.92552359
H	−2.33745111	4.35902792	−0.56207263
C	−2.60686724	5.75197365	−4.13783632
H	−0.62489435	4.80248756	−4.32683262
H	−1.94147127	3.64611622	−4.08729956
H	−4.48785850	5.07815211	−2.43911595
H	−3.89894579	6.03980415	−1.07998267
N	−2.99679521	6.45426344	−2.91169656
H	−2.18972678	6.48391209	−4.83250560
H	−3.50697451	5.33310131	−4.59230679
H	−2.12173627	7.59048540	−1.40109550
H	−1.39275967	7.78056233	−2.99814934

$TS_{2\text{-}IV}$(acetaldehyde)

E (TPSSh/def2-TZVP) =	−804.79112796
$G - E$ (TPSSh/def2-TZVP) =	0.33510386
$H - E$ (TPSSh/def2-TZVP) =	0.40294429
E (DLPNO-CCSD(T)tight/def-TZVPP//TPSSh/def2-TZVP) =	−799.53202955
E (DLPNO-CCSD(T)tight/def-QZVPP//TPSSh/def2-TZVP) =	−799.56731929
E (DLPNO-CCSD(T)/CBS//TPSSh/def2-TZVP) =	−803.381791028

C	0.89823483	2.68010388	−1.02480611
N	2.00104392	2.30856657	−1.74069444
C	2.32580907	0.97340309	−1.57025581
C	1.40736735	0.46799424	−0.71510188
N	0.55904711	1.51525764	−0.39786444
C	2.75105540	3.21261162	−2.60104626
C	−0.57979908	1.38294766	0.49766680
C	1.92293263	5.37320135	0.23721277
O	1.51276154	6.04812027	−0.69248545
C	1.17400928	5.08452555	1.49130561
N	−0.91260636	5.87509206	−1.91828338
C	−1.54207682	7.20705241	−2.19995265
H	−0.26659337	0.93911389	1.44372719
H	3.16671288	0.50792374	−2.05607587
H	−0.04153640	6.00525806	−1.35596977
H	−1.35281057	0.75644166	0.04849647
H	−0.97946857	2.37724937	0.68322858
H	3.81292792	3.16963738	−2.35441707
H	2.62049404	2.93983272	−3.65025518
H	2.38426944	4.22231454	−2.43479119
H	1.29160556	−0.52374732	−0.31152522
H	2.95044108	4.97304732	0.19029010
H	1.19710594	4.00940231	1.68320899
H	1.69812469	5.56876974	2.32384134
H	0.14780367	5.44570951	1.44754831
C	−2.86473863	6.93580833	−2.96778226
H	−1.69635204	7.70161320	−1.24106353
H	−0.82115207	7.78314069	−2.77969735
C	−0.54178607	5.18522785	−3.20324125
C	−1.85582924	4.99869438	−1.14286413
C	−3.08916753	4.73757109	−2.04546323
H	−1.28300584	4.10195863	−0.90127247
H	−2.10710127	5.53616519	−0.22786428
C	−1.83218783	5.06963785	−4.05358194
H	0.23429412	5.78633358	−3.67640434
H	−0.12547494	4.22254354	−2.90488973
H	−3.15339047	3.68106112	−2.30850947
H	−4.00800490	5.01377638	−1.52745437
N	−2.99645264	5.51317620	−3.28383968
H	−1.76238265	5.68330591	−4.95236337
H	−1.98975485	4.03574336	−4.36197252
H	−3.72489511	7.23574973	−2.36849031
H	−2.88582192	7.50530179	−3.89707107

IV(acetaldehyde)

E (TPSSh/def2-TZVP) =	−804.83235733
$G - E$ (TPSSh/def2-TZVP) =	0.34042977
$H - E$ (TPSSh/def2-TZVP) =	0.40477813
E (DLPNO-CCSD(T)tight/def-TZVPP//TPSSh/def2-TZVP) =	−799.55914847
E (DLPNO-CCSD(T)tight/def-QZVPP//TPSSh/def2-TZVP) =	−799.59425408
E (DLPNO-CCSD(T)/CBS//TPSSh/def2-TZVP) =	−803.426371933

C	1.73571591	2.95117670	−0.48049976
N	2.59450209	2.39953934	−1.35931954
C	2.31915255	1.05958576	−1.50641534
C	1.26706131	0.78363527	−0.69807609
N	0.91499650	1.96286243	−0.06818353
C	3.66437623	3.12294645	−2.05942493
C	−0.19949811	2.04428154	0.88495108
C	1.76720047	4.44217784	−0.18959170
O	1.46423623	5.12397893	−1.38088992
C	0.95132008	4.92111721	1.00433454
N	−1.09847915	5.35123931	−2.11561389
C	−1.46286778	6.75125442	−1.78670228
H	0.15392593	2.40898055	1.84575039
H	2.89032462	0.42811361	−2.16410984
H	0.45883503	5.13007933	−1.56370523
H	−0.59693548	1.03964493	1.00352372
H	−0.97747451	2.69609503	0.49522603
H	4.50440230	3.28173363	−1.38301644
H	3.98299639	2.51446097	−2.90190316
H	3.26691831	4.07428475	−2.40554209
H	0.74204620	−0.13635104	−0.50944181
H	2.81995046	4.66196362	0.02342880
H	1.25030838	4.42297486	1.92930750
H	1.13973003	5.98848251	1.11785204
H	−0.11926551	4.78556363	0.84987176
C	−2.81434163	7.11408867	−2.46534638
H	−1.52108174	6.83022695	−0.69819761
H	−0.64678979	7.39204123	−2.12587608
C	−0.99946761	5.21573795	−3.58964470
C	−2.16698301	4.45549279	−1.62656707
C	−3.52296827	4.83914056	−2.29151916
H	−1.87692938	3.42799275	−1.86634412
H	−2.21701159	4.55903794	−0.53821439
C	−2.36281971	5.58075466	−4.24306228
H	−0.19495418	5.87320785	−3.92494886
H	−0.70668889	4.18546451	−3.80740276
H	−3.93570537	3.99952794	−2.85438740
H	−4.25740452	5.13449926	−1.53957580
N	−3.33080389	5.96120500	−3.21026835
H	−2.25475061	6.41784376	−4.93509831
H	−2.77264851	4.73588152	−4.80023145
H	−3.56224018	7.40575397	−1.72535150
H	−2.69287559	7.94343008	−3.16445810

2(acrolein)

E (TPSSh/def2-TZVP) =	−842.91963566
$G - E$ (TPSSh/def2-TZVP) =	0.33538432
$H - E$ (TPSSh/def2-TZVP) =	0.40867036
E (DLPNO-CCSD(T)tight/def-TZVPP//TPSSh/def2-TZVP) =	−837.41965643
E (DLPNO-CCSD(T)tight/def-QZVPP//TPSSh/def2-TZVP) =	−837.45633907
E (DLPNO-CCSD(T)/CBS//TPSSh/def2-TZVP) =	−841.437890221

N	0.85557087	−1.09106881	3.81925376
C	−0.08914223	−0.79715106	2.91957063
N	−1.22789781	−1.36546757	3.32210885
C	−1.00277873	−2.04731634	4.50143055
C	0.30641007	−1.87403716	4.81669177
C	−2.49421690	−1.33289868	2.58733537
C	2.24810980	−0.64451724	3.74507051
C	−0.13509059	−3.90529829	0.11987401
O	−0.57916859	−2.88998193	0.63140687
N	0.50645264	0.72297926	0.33337038
C	1.00541537	2.11155805	0.40916176
C	1.23429303	2.67068903	−1.02693361
N	0.88419213	1.65407535	−2.02425440
C	−0.52332689	1.28216556	−1.85904784
C	−0.75965684	0.71435645	−0.42883336
C	1.49795885	−0.10168565	−0.38899966
C	1.72146173	0.46884392	−1.81968711
H	0.16213826	−4.75676056	0.76007576
H	2.90520383	−1.51135888	3.68773875
H	−1.78470547	−2.58521122	5.00850172
H	0.07331334	−0.20346784	2.00324832
H	2.48821040	−0.05272022	4.62736447
H	2.36228844	−0.03612400	2.85163095
H	−2.66902435	−2.30744269	2.13600012
H	−3.29695742	−1.07418315	3.27571059
H	−2.41852549	−0.58160259	1.80661557
H	0.88484134	−2.22644386	5.65283174
C	0.05176891	−4.10645058	−1.32639368
H	0.27002380	2.70316521	0.96046088
H	1.93356476	2.10416509	0.98788063
H	−1.11984779	−0.31738556	−0.45461891
H	−1.48567714	1.31856084	0.12345827
H	2.42608343	−0.09378410	0.19046460
H	1.12120816	−1.12619628	−0.41310250
H	−0.77226691	0.54368227	−2.62491278
H	−1.13463991	2.16883675	−2.03877384
H	2.76355443	0.75633884	−1.97412899
H	1.46105731	−0.26827390	−2.58296966
H	0.61848921	3.55366623	−1.20946148
H	2.27810418	2.95243131	−1.17987535
C	−0.38161848	−3.22243637	−2.22848768
H	0.54208881	−5.02539286	−1.63014232
H	−0.25176767	−3.38081422	−3.29177294
H	−0.89277792	−2.31989254	−1.91339473

$TS_{2\text{-}IV}$(acrolein)

E (TPSSh/def2-TZVP) =	−842.89138999
$G - E$ (TPSSh/def2-TZVP) =	0.33928487
$H - E$ (TPSSh/def2-TZVP) =	0.40939093
E (DLPNO-CCSD(T)tight/def-TZVPP//TPSSh/def2-TZVP) =	−837.39143775
E (DLPNO-CCSD(T)tight/def-QZVPP//TPSSh/def2-TZVP) =	−837.42794400
E (DLPNO-CCSD(T)/CBS//TPSSh/def2-TZVP) =	−841.410991559

N	0.39940733	−1.12056530	3.69339640
C	−0.15704979	−1.00256436	2.45221027
N	−1.32519486	−1.69906438	2.59717250
C	−1.48722030	−2.21594658	3.87167952
C	−0.38857451	−1.84803011	4.56966088
C	−2.31901729	−1.84504903	1.54441673
C	1.67742021	−0.53437303	4.06845371
C	0.75497294	−3.17429603	0.34340757
O	0.40256002	−2.40418513	−0.54464608
N	0.65556860	0.34596132	−0.57436962
C	1.74763705	0.98000615	0.23615830
C	1.91343816	2.43800666	−0.26475896
N	0.89197205	2.76017956	−1.26269504
C	−0.43724355	2.54788044	−0.68626313
C	−0.64084532	1.05718363	−0.31059608
C	0.99166710	0.40403700	−2.03611414
C	1.06300035	1.90241720	−2.43592614
H	1.43828222	−2.82046664	1.13062756
H	2.31766477	−1.28927359	4.52694494
H	−2.34991478	−2.78934623	4.16619100
H	0.56084817	−0.65822271	−0.30070961
H	1.53156331	0.28690773	4.77240937
H	2.15441758	−0.15959695	3.16655043
H	−2.67550895	−2.87510519	1.51197338
H	−3.16613007	−1.17862450	1.71867579
H	−1.84950208	−1.60231312	0.59497999
H	−0.10520678	−2.04093425	5.59058166
C	0.31257017	−4.56672804	0.44881571
H	1.42098920	0.89910904	1.27126203
H	2.64347953	0.37489559	0.09519147
H	−1.39815819	0.56663797	−0.92240185
H	−0.86103123	0.88735649	0.74287369
H	1.93884910	−0.11934562	−2.16350230
H	0.21603559	−0.14886066	−2.56440106
H	−1.18685795	2.86492785	−1.41181898
H	−0.53267410	3.18159558	0.19607692
H	2.02562695	2.12696728	−2.89592491
H	0.28063127	2.14503403	−3.15567290
H	1.82052386	3.13703015	0.56697750
H	2.89435457	2.58256869	−0.71891803
C	−0.52086322	−5.11218063	−0.44259443
H	0.69616572	−5.13650410	1.28813284
H	−0.84525853	−6.14231330	−0.36281280
H	−0.89084635	−4.53025985	−1.27937111

2(benzaldehyde)

E (TPSSh/def2-TZVP) =	−996.66187934
$G - E$ (TPSSh/def2-TZVP) =	0.38260002
$H - E$ (TPSSh/def2-TZVP) =	0.45887807
E (DLPNO-CCSD(T)tight/def-TZVPP//TPSSh/def2-TZVP) =	−990.13770147
E (DLPNO-CCSD(T)tight/def-QZVPP//TPSSh/def2-TZVP) =	−990.18021973
E (DLPNO-CCSD(T)/CBS//TPSSh/def2-TZVP) =	−994.878189323

N	1.45944027	3.04892606	−1.30812310
C	0.90206839	2.09370888	−2.26928812
C	0.32343283	0.85916978	−1.51641906
N	0.50850100	1.02313268	−0.05885905
C	1.95327660	1.16244866	0.21826306
C	2.52400202	2.39596850	−0.54150538
C	0.40159106	3.48471426	−0.39253468
C	−0.18026784	2.25815760	0.37086715
O	1.78181045	−3.17859763	0.77581376
C	1.96870690	−3.64130886	1.88669368
C	2.46591639	−2.87157823	3.03650526
C	−0.65569430	−1.44723314	1.32686041
N	−1.11361999	−2.54977905	0.72670921
C	−1.51576556	−3.45498840	1.68647881
C	−1.30473914	−2.87183383	2.89531274
N	−0.77334913	−1.62132672	2.64759580
C	−1.05268835	−2.81001478	−0.71435779
C	−0.38928712	−0.64257353	3.66683533
H	−1.26958726	−0.34884076	4.23731352
H	−1.91394524	−4.42095019	1.42917577
H	−0.23685338	−0.55548544	0.82694606
H	0.03222067	0.22394790	3.16488627
H	0.35906347	−1.08381289	4.32294509
H	−1.95466265	−3.33945621	−1.01462986
H	−0.99574854	−1.85791964	−1.23504436
H	−0.16450213	−3.40387254	−0.92518805
H	−1.49245366	−3.22550445	3.89396371
H	1.74273444	−4.70559792	2.09620331
H	0.82893942	−0.06378350	−1.81426315
H	−0.74775991	0.74201137	−1.70666805
H	2.07645663	1.26680550	1.30043622
H	2.44101264	0.23478429	−0.09204849
H	−1.24881424	2.13129467	0.17697697
H	−0.04347982	2.35981630	1.45163765
H	2.94347649	3.12838048	0.15154314
H	3.31311948	2.10035099	−1.23590079
H	−0.37097182	3.98764583	−0.97760707
H	0.82756465	4.21457881	0.29904172
H	1.69619226	1.79836137	−2.95793768
H	0.12768840	2.60242962	−2.84688023
C	2.43400922	−3.44580788	4.31073122
C	2.85868680	−2.71543782	5.41439659
C	3.33541564	−1.41819213	5.24046021
C	3.38603008	−0.84710447	3.96663105
C	2.94733844	−1.56787009	2.86759397
H	2.07720158	−4.46393377	4.43027792
H	2.83431285	−3.15698690	6.40312654
H	3.68192254	−0.85324943	6.09780096
H	3.77842596	0.15508515	3.84052376
H	2.98117953	−1.15110248	1.86895005

$\mathbf{TS_{2\text{-}IV}}$(benzaldehyde)

E (TPSSh/def2-TZVP) =	−996.63090803
$G - E$ (TPSSh/def2-TZVP) =	0.38694413
$H - E$ (TPSSh/def2-TZVP) =	0.45884594
E (DLPNO-CCSD(T)tight/def-TZVPP//TPSSh/def2-TZVP) =	−990.09086083
E (DLPNO-CCSD(T)tight/def-QZVPP//TPSSh/def2-TZVP) =	−990.13309856
E (DLPNO-CCSD(T)/CBS//TPSSh/def2-TZVP) =	−994.843397618

N	0.74828961	2.77179125	−1.12000621
C	0.13667604	1.65407264	−1.84041261
C	0.60942020	0.30079777	−1.24650715
N	1.60676637	0.59897161	−0.16691177
C	2.75921033	1.37312404	−0.73195307
C	2.20336083	2.71797946	−1.27282991
C	0.39808654	2.69302117	0.29857010
C	0.94511465	1.38194992	0.92539052
O	2.39068829	−1.77808209	0.48997023
C	2.00473957	−2.39972063	1.50389251
C	2.35742025	−2.00979072	2.88839573
C	−0.27535077	−2.08553304	1.66947624
N	−1.05315957	−2.78602613	0.80402530
C	−2.36944715	−2.85017146	1.22904821
C	−2.42562104	−2.16593588	2.39621997
N	−1.14162057	−1.70966166	2.64514119
C	−0.54565812	−3.43149193	−0.40313788
C	−0.77308451	−0.91785735	3.81454991
H	−0.91842926	−1.50153679	4.72369701
H	−3.13679602	−3.36484486	0.67594973
H	1.97295505	−0.34116096	0.21284447
H	−1.38926002	−0.01866536	3.86089808
H	0.27515534	−0.64717163	3.72855526
H	−0.54724707	−4.51562107	−0.28096010
H	−1.17507436	−3.16819807	−1.25396132
H	0.46966863	−3.08205861	−0.57643745
H	−3.25301452	−1.96304608	3.05496826
H	1.71978887	−3.45500916	1.40039296
H	1.10955112	−0.33873514	−1.97290718
H	−0.19354206	−0.26541230	−0.77533287
H	3.48307225	1.50241760	0.07255639
H	3.20915795	0.75139286	−1.50540615
H	0.16802201	0.74094791	1.33987662
H	1.69669178	1.55926570	1.69376205
H	2.63754766	3.55946142	−0.73207452
H	2.44651652	2.83469457	−2.32919675
H	−0.68808201	2.73760393	0.38712745
H	0.81386760	3.56300151	0.80775948
H	0.41261386	1.73111903	−2.89255595
H	−0.94763721	1.74585164	−1.76854997
C	2.01573230	−2.85956258	3.94312786
C	2.33861742	−2.52111318	5.24951170
C	3.01214079	−1.32911594	5.51105687
C	3.38023872	−0.49071038	4.46092528
C	3.06054585	−0.83452187	3.15309724
H	1.48437842	−3.78209748	3.73136205
H	2.07333958	−3.18418077	6.06420874
H	3.26678341	−1.06357535	6.53003552
H	3.93201124	0.41940059	4.66465310
H	3.38087423	−0.21243712	2.32561484

IV(benzaldehyde)

E (TPSSh/def2-TZVP) =	−996.67289729
$G - E$ (TPSSh/def2-TZVP) =	0.38867989
$H - E$ (TPSSh/def2-TZVP) =	0.45999418
E (DLPNO-CCSD(T)tight/def-TZVPP//TPSSh/def2-TZVP) =	−990.13656678
E (DLPNO-CCSD(T)tight/def-QZVPP//TPSSh/def2-TZVP) =	−990.17875088
E (DLPNO-CCSD(T)/CBS//TPSSh/def2-TZVP) =	−994.895445396

N	2.35626555	3.30282044	−0.56114818
C	1.38019839	2.96881305	−1.60257608
C	0.64602245	1.64482605	−1.24613505
N	1.13372825	1.14185572	0.06056102
C	2.59033071	0.89169608	−0.03215025
C	3.31920294	2.20526623	−0.43584502
C	1.65824087	3.47813706	0.71507571
C	0.89576237	2.17619222	1.09369886
O	−0.48845116	−0.82088179	0.71296690
C	0.09145886	−2.01330029	1.13917261
C	1.41638651	−1.83527514	1.86892772
C	−0.90161814	−2.71524391	2.03166297
N	−0.80216353	−4.00486043	2.40600375
C	−1.85208959	−4.32565217	3.24070126
C	−2.60511819	−3.20719600	3.36872227
N	−2.00596567	−2.21557645	2.61427616
C	0.27443107	−4.92065301	2.02239991
C	−2.56785642	−0.85739320	2.50255888
H	−1.83433631	−0.12488899	2.82455846
H	−1.96765332	−5.31041479	3.65797739
H	0.23321407	−0.12058790	0.45052248
H	−3.44144282	−0.82697566	3.15008381
H	−2.84411706	−0.65949952	1.47186131
H	1.20886984	−4.60642839	2.48516807
H	0.00364852	−5.91698769	2.36209798
H	0.38314125	−4.92464331	0.93870320
H	−3.51151023	−3.02563428	3.91865993
H	0.27510524	−2.69665716	0.29185964
H	0.82889697	0.86390761	−1.98749092
H	−0.43410963	1.78510171	−1.16510745
H	2.92449265	0.52401158	0.93930164
H	2.74581068	0.09754594	−0.76588973
H	−0.18297059	2.33942102	1.15518854
H	1.23416217	1.76881603	2.04939368
H	4.06262028	2.48573012	0.31327006
H	3.83297768	2.09292691	−1.39268092
H	0.97135565	4.32112900	0.61690360
H	2.39852183	3.73498115	1.47564785
H	1.91057555	2.88035413	−2.55271381
H	0.67572477	3.79856623	−1.68790521
C	2.58854590	−2.35137472	1.32213480
C	3.80935321	−2.15517715	1.96182156
C	3.86097553	−1.43917719	3.15276996
C	2.69055125	−0.92133495	3.70518482
C	1.47307145	−1.12185096	3.06746315
H	2.54881406	−2.89738006	0.38484562
H	4.71750545	−2.55530143	1.52735797
H	4.80976295	−1.28403764	3.65183580
H	2.72933273	−0.36519152	4.63419271
H	0.56482602	−0.71246457	3.49690656

5.3.2 DBU

I

E (TPSSh/def2-TZVP) =	−767.73593556
$G - E$ (TPSSh/def2-TZVP) =	0.34080240
$H - E$ (TPSSh/def2-TZVP) =	0.40595810
E (DLPNO-CCSD(T)tight/def-TZVPP//TPSSh/def2-TZVP) =	−762.58916484
E (DLPNO-CCSD(T)tight/def-QZVPP//TPSSh/def2-TZVP) =	−762.62258409
E (DLPNO-CCSD(T)/CBS//TPSSh/def2-TZVP) =	−766.338072823

C	−2.36922371	0.57997080	0.26609785
N	−1.22668645	0.22415062	−0.58349437
C	0.05268062	0.50085137	−0.19481010
N	0.38485530	1.20195187	0.85376633
C	−0.68884587	1.74545188	1.67824087
C	−1.91199572	0.83106020	1.69680844
C	−1.49102250	−0.60446742	−1.76225445
C	−1.23942123	−2.10294929	−1.51274321
C	−0.02112780	−2.35753825	−0.61738902
C	1.26180285	−1.62107020	−1.02739218
C	1.15367043	−0.07020179	−1.06348645
H	−0.30238068	1.89820692	2.69973713
H	−0.97632471	2.74567257	1.30188299
H	−1.64301882	−0.12645663	2.17356988
H	−2.73359743	1.27206663	2.28019575
H	−3.09369146	−0.24838707	0.23160966
H	−2.87232637	1.47233318	−0.14689447
H	0.99485356	0.27316690	−2.09743190
H	2.09804816	0.38360482	−0.73479356
H	−2.53335579	−0.42750360	−2.06301899
H	−0.86951938	−0.24482806	−2.59450365
H	−2.12446756	−2.55983936	−1.04095898
H	−1.11430165	−2.59954531	−2.48895832
H	0.18296588	−3.43936011	−0.58683042
H	−0.27671773	−2.06928036	0.41746262
H	1.60186095	−1.97748164	−2.01287484
H	2.05302240	−1.90188586	−0.31338815
H	2.05778958	1.54716757	1.39108418
C	3.08266261	1.54788138	1.87309586
N	3.56698621	0.50756323	2.56909307
C	4.83916557	0.80953415	3.02335265
C	5.12460094	2.07159728	2.58360405
N	4.01947981	2.50895254	1.87137839
C	3.87859502	3.81321796	1.21836099
H	6.00982777	2.68665549	2.71667481
H	5.42771619	0.11302345	3.61335889
C	2.82830851	−0.73917850	2.79139703
H	4.67655434	3.94098659	0.47506766
H	2.90247330	3.84611176	0.72148956
H	3.93804776	4.61113941	1.97044030
H	3.38481301	−1.58006905	2.35665674
H	2.69130969	−0.89979588	3.86888343
H	1.85333242	−0.63925620	2.29858594

2(formaldehyde)

E (TPSSh/def2-TZVP) =	−882.30079781
$G-E$ (TPSSh/def2-TZVP) =	0.36280025
$H-E$ (TPSSh/def2-TZVP) =	0.43859561
E (DLPNO-CCSD(T)tight/def-TZVPP//TPSSh/def2-TZVP) =	−876.51241002
E (DLPNO-CCSD(T)tight/def-QZVPP//TPSSh/def2-TZVP) =	−876.55079009
E (DLPNO-CCSD(T)/CBS//TPSSh/def2-TZVP) =	−880.740895775

C	−3.94159388	−3.85059933	−0.01745115
N	−2.64929398	−3.78824312	0.66090885
C	−2.16186246	−4.90041374	1.11894900
N	−2.76297367	−6.11999215	1.04736282
C	−3.97582535	−6.31395673	0.24676613
C	−4.13815613	−5.17492282	−0.74619288
C	−0.80908472	−4.86754528	1.78637838
C	0.30802550	−5.57015606	0.96803793
C	−0.18290938	−6.64230854	−0.01060497
C	−0.95663402	−7.80087960	0.62261267
C	−2.08869574	−7.31881835	1.54561606
C	−1.09966633	−1.28683591	0.28571337
N	−1.43529335	−0.48165936	1.29913368
C	−0.73940635	0.70517266	1.18917292
C	0.03604554	0.60376064	0.07857003
N	−0.20307764	−0.64255179	−0.46895678
C	−2.40201975	−0.84007039	2.34350031
C	0.43635220	−1.18194862	−1.67291820
O	−1.03316943	−4.03823968	−2.61340365
C	−0.98824586	−4.65498648	−3.65009486
H	−1.54307962	−5.60069138	−3.78552364
H	−3.99706001	−3.01502668	−0.72139384
H	−4.74973760	−3.70414739	0.71148997
H	−3.38225948	−5.26024002	−1.53119904
H	−5.12299998	−5.22874867	−1.21451945
H	−3.88701331	−7.27077990	−0.27376998
H	−4.84550611	−6.37951982	0.91004393
H	−0.88810123	−5.31864050	2.77818834
H	−0.55243048	−3.82000426	1.94373840
H	−2.84885125	−8.09272219	1.66199601
H	−1.70153131	−7.11540652	2.54431673
H	−1.36745193	−8.42596778	−0.17573863
H	−0.28706969	−8.43968524	1.20640601
H	0.67708917	−7.04335927	−0.55396010
H	−0.81961474	−6.15715572	−0.75630968
H	1.03821954	−5.99796470	1.66044703
H	0.83815201	−4.81105906	0.38629015
H	−2.71463811	−1.86676705	2.15350562
H	0.72685657	1.29905924	−0.36559487
H	−1.52315079	−2.29915461	0.17291786
H	−1.92171052	−0.76095112	3.31785021
H	−3.25502132	−0.16439768	2.29280865
H	1.49965763	−1.32528525	−1.48169098
H	0.30083817	−0.47606150	−2.49138041
H	−0.03281966	−2.13149441	−1.92241858
H	−0.85558003	1.50638544	1.89802184
H	−0.39221713	−4.30675431	−4.51278221

$\mathbf{TS_{2\text{-}IV}}$(formaldehyde)

E (TPSSh/def2-TZVP) =	−882.28409265
$G - E$ (TPSSh/def2-TZVP) =	0.36816736
$H - E$ (TPSSh/def2-TZVP) =	0.43901577
E (DLPNO-CCSD(T)tight/def-TZVPP//TPSSh/def2-TZVP) =	−876.48976366
E (DLPNO-CCSD(T)tight/def-QZVPP//TPSSh/def2-TZVP) =	−876.52816789
E (DLPNO-CCSD(T)/CBS//TPSSh/def2-TZVP) =	−880.721629408

N	−1.23362982	−0.11310526	0.80158167
C	−0.81917554	−1.14139161	0.01464871
N	−1.11283713	−0.70547242	−1.24128701
C	−1.69410997	0.55043889	−1.23608742
C	−1.77075627	0.92707996	0.06281972
C	−0.81976602	−1.47358081	−2.44635891
C	−1.11244639	−0.10866208	2.25330853
C	1.22017201	−2.51644657	0.11159122
H	1.28119491	−2.26128386	1.18148060
O	0.96917557	−3.65066755	−0.27307605
N	−1.88300413	−4.15578808	0.54369506
C	−1.75222451	−5.41903079	0.91231790
N	−2.80077241	−6.23305394	0.97240336
C	−4.13901094	−5.81922967	0.51933717
C	−4.04262638	−4.64674406	−0.44423858
C	−3.16039446	−3.56313789	0.15546732
C	−0.39983225	−5.94440312	1.28257826
C	0.13889493	−7.01543447	0.29151305
C	−0.92175937	−7.69994027	−0.57499959
C	−1.99995932	−8.46631286	0.19689375
C	−2.60878156	−7.64595865	1.34222087
H	−2.94843246	−2.76706993	−0.55530055
H	−3.63329842	−3.11050223	1.03233113
H	−3.61492412	−4.98152501	−1.39250600
H	−5.04055187	−4.25591049	−0.64458754
H	−4.59847237	−6.68146973	0.03465113
H	−4.74333605	−5.56480041	1.39516335
H	−0.47496116	−6.35402387	2.29275505
H	0.29238152	−5.10579659	1.30992173
H	−3.58516751	−8.03145511	1.63495399
H	−1.97378647	−7.68336841	2.22668121
H	−2.78515761	−8.77645230	−0.49781609
H	−1.58511627	−9.38027587	0.63062679
H	−0.42367091	−8.38726066	−1.26204235
H	−1.40118074	−6.94452559	−1.20741334
H	0.69650539	−7.76047552	0.86354303
H	0.85088944	−6.51782540	−0.36796832
H	−0.69957923	−1.06778575	2.55641149
H	−1.99533494	1.05984272	−2.13582321
H	−1.04186809	−3.58586482	0.44534398
H	−0.44677221	0.69320079	2.57553602
H	−2.09197383	0.03011196	2.71318462
H	−0.13763256	−0.91631082	−3.08973648
H	−1.73998673	−1.67780247	−2.99678277
H	−0.35332443	−2.40961213	−2.14676726
H	−2.15275611	1.82767091	0.51306643
H	1.58510278	−1.73752515	−0.57574967

IV(formaldehyde)

E (TPSSh/def2-TZVP) = −882.33219498
$G - E$ (TPSSh/def2-TZVP) = 0.37301678
$H - E$ (TPSSh/def2-TZVP) = 0.44161486
E (DLPNO-CCSD(T)tight/def-TZVPP//TPSSh/def2-TZVP) = −876.52974844
E (DLPNO-CCSD(T)tight/def-QZVPP//TPSSh/def2-TZVP) = −876.56795299
E (DLPNO-CCSD(T)/CBS//TPSSh/def2-TZVP) = −880.775439413

N	−1.11715238	−0.69313595	0.78039001
C	−0.32016336	−1.05325936	−0.23960305
N	−0.69303373	−0.33370779	−1.31053200
C	−1.73521865	0.49931813	−0.96344217
C	−2.00167857	0.27464603	0.34869460
C	−0.07985092	−0.44122569	−2.63986479
C	−1.09519709	−1.28363346	2.12313145
C	0.71984992	−2.13577710	−0.24892807
H	0.89961454	−2.45256148	0.78541208
O	0.31340540	−3.18630643	−1.08065727
N	−1.80599200	−4.09279998	0.23390324
C	−1.75589450	−5.25451092	0.82566208
N	−2.83188188	−6.01170906	1.14727281
C	−4.17241337	−5.67323035	0.65483697
C	−4.07879702	−4.70662039	−0.51386514
C	−3.12037065	−3.58035296	−0.14631375
C	−0.40399339	−5.81619006	1.18468582
C	−0.00648999	−7.07047275	0.35709760
C	−1.17718770	−7.84397922	−0.25774672
C	−2.19254561	−8.39589997	0.74591763
C	−2.65963271	−7.33497855	1.75430196
H	−2.98633291	−2.89656580	−0.98890951
H	−3.54524138	−2.99168074	0.67906284
H	−3.69654940	−5.22771236	−1.39580342
H	−5.07089718	−4.32102805	−0.75602698
H	−4.66119579	−6.60098164	0.34932342
H	−4.76186017	−5.24363913	1.47234484
H	−0.39366836	−6.04845948	2.25244289
H	0.32932161	−5.02688626	1.02573972
H	−3.61673814	−7.61209646	2.19862866
H	−1.94837551	−7.25103911	2.57591611
H	−3.05119927	−8.79468201	0.19820691
H	−1.76409214	−9.23133338	1.30746708
H	−0.77938675	−8.67012912	−0.85196371
H	−1.69598033	−7.18780410	−0.96448900
H	0.58969962	−7.73322930	0.98977987
H	0.64397418	−6.74489545	−0.45734384
H	−1.17739813	−2.36631421	2.02676248
H	−2.18965091	1.16997990	−1.67144328
H	−0.50092915	−3.60229108	−0.62064825
H	−0.17631936	−1.00789795	2.63849548
H	−1.94866208	−0.89370152	2.67201716
H	0.79982330	0.20021224	−2.69402983
H	−0.81169554	−0.12371519	−3.37880839
H	0.19116367	−1.48194978	−2.80562046
H	−2.73408519	0.70994173	1.00541178
H	1.65858297	−1.72895546	−0.63480385

2(acetaldehyde)

E (TPSSh/def2-TZVP) =	−921.65173499
$G - E$ (TPSSh/def2-TZVP) =	0.38886527
$H - E$ (TPSSh/def2-TZVP) =	0.46821982
E (DLPNO-CCSD(T)tight/def-TZVPP//TPSSh/def2-TZVP) =	−915.58076498
E (DLPNO-CCSD(T)tight/def-QZVPP//TPSSh/def2-TZVP) =	−915.62114161
E (DLPNO-CCSD(T)/CBS//TPSSh/def2-TZVP) =	−920.016010352

C	−2.17771563	−5.20626837	0.79377641
N	−2.51815718	−4.00650842	0.43298178
C	−3.92114369	−3.78086947	0.09581171
C	−4.55375438	−5.00994060	−0.54700623
C	−4.39633335	−6.19414291	0.39307598
N	−3.01863887	−6.27262078	0.88943825
C	−2.50750284	−7.59984233	1.23259381
C	−1.76132892	−8.27102554	0.06940109
C	−0.89529618	−7.28204252	−0.71496022
C	0.01041631	−6.39386519	0.14462130
C	−0.73297507	−5.46508408	1.14514137
C	−0.82739051	−1.63198072	−0.08357191
N	−0.25477366	−1.64955216	−1.29035291
C	0.42427224	−0.46307675	−1.49075414
C	0.25175540	0.27956296	−0.36684413
N	−0.52288262	−0.46995355	0.49656438
C	−0.36896544	−2.76650856	−2.22992559
C	−1.03703287	−0.02718752	1.79283894
O	−3.33980072	0.08547477	−0.50030381
C	−4.42768972	0.40916962	−0.92763424
C	−5.12501408	1.69131753	−0.60589409
H	−4.97552620	−0.27369366	−1.60904726
H	−3.97338736	−2.91842010	−0.57447683
H	−4.47748588	−3.50877563	1.00260991
H	−4.04851790	−5.22189006	−1.49426493
H	−5.61179915	−4.84800151	−0.76297477
H	−4.62326873	−7.12987183	−0.12370916
H	−5.08482360	−6.11042465	1.24124079
H	−0.68626330	−5.88544074	2.15260289
H	−0.23843074	−4.49542957	1.19376194
H	−3.35912156	−8.20456429	1.54810535
H	−1.85332333	−7.51105201	2.10037349
H	−2.47597879	−8.73610676	−0.61577785
H	−1.14688854	−9.07907391	0.47777309
H	−0.27719938	−7.83529938	−1.42669215
H	−1.54851222	−6.63868480	−1.31450928
H	0.72328249	−7.01254714	0.69632697
H	0.60439655	−5.77073579	−0.52963351
H	−1.82250007	0.70849616	1.62824366
H	0.96632917	−0.25731595	−2.39708616
H	−1.44999626	−2.45900792	0.30559411
H	−1.44683651	−0.89117286	2.31022401
H	−0.22250449	0.39978493	2.37549293
H	0.61961617	−3.17657833	−2.43400403
H	−0.82361069	−2.41540925	−3.15546568
H	−1.00103421	−3.52143009	−1.76547518
H	0.60846460	1.26133140	−0.10918827
H	−5.33826880	2.22999334	−1.53532007
H	−6.09624419	1.46877607	−0.15092190
H	−4.52972826	2.31228592	0.06160014

$\mathbf{TS_{2\text{-}IV}}$(acetaldehyde)

E (TPSSh/def2-TZVP) =	−921.63254135
$G-E$ (TPSSh/def2-TZVP) =	0.39270959
$H-E$ (TPSSh/def2-TZVP) =	0.46813783
E (DLPNO-CCSD(T)tight/def-TZVPP//TPSSh/def2-TZVP) =	−915.56059467
E (DLPNO-CCSD(T)tight/def-QZVPP//TPSSh/def2-TZVP) =	−915.60082656
E (DLPNO-CCSD(T)/CBS//TPSSh/def2-TZVP) =	−919.997412262

N	0.09369824	−0.98085900	−1.32793368
C	−0.72753244	−1.49600918	−0.36675732
N	−0.72514398	−0.50747200	0.57483852
C	0.06104275	0.57312826	0.21471081
C	0.58437165	0.27110039	−0.99780795
C	−1.48856190	−0.58056725	1.81122036
C	0.42876280	−1.68685432	−2.55527118
N	−3.49970618	−4.04500251	0.58056532
C	−4.92823173	−4.16100581	0.87180304
C	−5.34780707	−5.61111749	0.69310110
C	−4.43467696	−6.51395881	1.50829137
N	−3.02036164	−6.20728778	1.23396197
C	−2.63677963	−5.03094381	0.74745225
C	−1.20145070	−4.81658231	0.38454401
C	−0.73375626	−5.68543567	−0.81690060
C	−1.59653066	−6.91391983	−1.12190717
C	−1.69761464	−7.93736879	0.01289559
C	−2.03944307	−7.29680055	1.36517440
O	−4.02549788	−1.44495458	−0.42120584
C	−3.43591684	−0.89307990	−1.33178878
C	−3.30260724	0.58641646	−1.47481577
H	−2.97307693	−1.49502099	−2.13514163
H	−5.44975046	−3.49178071	0.19073825
H	−5.11790008	−3.81808329	1.89317189
H	−5.28763253	−5.88207650	−0.36396459
H	−6.38011130	−5.74839197	1.01568611
H	−4.59474144	−7.56166167	1.25110686
H	−4.61762380	−6.39925611	2.58085632
H	−0.60323272	−5.04108300	1.27130463
H	−1.05573163	−3.75367092	0.15859072
H	−2.46573244	−8.02551811	2.05421855
H	−1.14511736	−6.89487745	1.84035569
H	−2.45295541	−8.68340647	−0.24788493
H	−0.75260312	−8.47469395	0.13059315
H	−1.20169874	−7.41090163	−2.01078821
H	−2.60426493	−6.57900234	−1.39138204
H	0.30105749	−5.98722522	−0.63945278
H	−0.72570804	−5.05026189	−1.70550050
H	−2.24686198	0.20357596	1.83950009
H	1.25512425	0.82565875	−1.63237438
H	−3.18846933	−3.16567205	0.16661337
H	−1.97688883	−1.55095879	1.84256121
H	−0.82597285	−0.47553451	2.67156553
H	1.49331901	−1.92583319	−2.58042560
H	0.17783057	−1.07752331	−3.42519752
H	−0.15029962	−2.60693861	−2.57482288
H	0.18549966	1.44311049	0.83739225
H	−2.23906743	0.84866284	−1.50149479
H	−3.72527696	0.89686167	−2.43613929
H	−3.80047267	1.10895344	−0.65970596

IV(acetaldehyde)

E (TPSSh/def2-TZVP) =	−921.66730359
$G - E$ (TPSSh/def2-TZVP) =	0.40076806
$H - E$ (TPSSh/def2-TZVP) =	0.47106129
E (DLPNO-CCSD(T)tight/def-TZVPP//TPSSh/def2-TZVP) =	−915.57513462
E (DLPNO-CCSD(T)tight/def-QZVPP//TPSSh/def2-TZVP) =	−915.61510028
E (DLPNO-CCSD(T)/CBS//TPSSh/def2-TZVP) =	−920.036023359

C	−1.43297280	−0.54708481	0.99045572
N	−0.56591759	−1.14403864	0.21944134
C	−2.64482798	0.08191655	0.35264692
C	0.63117949	−1.69735155	0.84887284
C	1.06201121	−0.88209156	2.06190030
H	1.42704931	−1.72001720	0.09940413
H	0.44804409	−2.73765445	1.15302068
C	−0.09858526	−0.80652356	3.03926939
H	1.33836116	0.12453987	1.73654094
H	1.93058118	−1.32684386	2.55130306
N	−1.33267214	−0.43619662	2.33724503
H	0.09069444	−0.05128450	3.80522327
H	−0.24382538	−1.76501590	3.54965530
C	−2.33188767	0.31639379	3.10154300
H	−3.54189624	−0.32171129	0.82851439
H	−2.66803831	−0.23891715	−0.68678291
C	−2.65218022	1.63486092	0.41774148
C	−2.17388439	1.83705337	2.95066725
H	−2.23347777	0.01412940	4.14523198
H	−3.32477802	0.00287506	2.77962126
H	−1.41065267	2.20431148	3.64276661
H	−3.11658074	2.30689610	3.24637596
C	−1.79042671	2.24686225	1.52664515
H	−1.83401622	3.33563781	1.44546013
H	−0.74543103	1.97333581	1.34681924
H	−3.68609864	1.97638022	0.51499938
H	−2.28463276	2.01416768	−0.53811357
C	0.35366481	−2.65004027	−2.70502342
N	1.66754385	−2.62260301	−3.00084451
C	2.29706021	−3.70877459	−2.43533774
C	1.34475399	−4.41358039	−1.77644712
N	0.14771894	−3.74610476	−1.94958815
C	2.32618817	−1.56176339	−3.77145903
C	−1.10278213	−4.16181791	−1.30268621
C	−0.57221889	−1.52588562	−3.11781113
O	−0.26873417	−0.40210659	−2.32597302
C	−2.05896124	−1.86222683	−3.15490035
H	−1.85546738	−4.39381330	−2.05227660
H	3.35230892	−3.88239971	−2.55219904
H	−0.42910000	−0.65413424	−1.34909261
H	−0.88509957	−5.05351363	−0.72012355
H	−1.44098313	−3.36006108	−0.64729480
H	2.12539508	−1.69390390	−4.83461558
H	3.39611346	−1.63130095	−3.59172399
H	1.94932940	−0.60043814	−3.42695221
H	1.40687977	−5.32105864	−1.20236044
H	−0.26931282	−1.27100200	−4.13925185
H	−2.45912091	−2.03327691	−2.15685246
H	−2.26183869	−2.73362033	−3.78336253
H	−2.57800601	−1.00470105	−3.58309792

2(acrolein)

E (TPSSh/def2-TZVP) =	−959.74886232
$G - E$ (TPSSh/def2-TZVP) =	0.39257215
$H - E$ (TPSSh/def2-TZVP) =	0.47455549
E (DLPNO-CCSD(T)tight/def-TZVPP//TPSSh/def2-TZVP) =	−953.43545989
E (DLPNO-CCSD(T)tight/def-QZVPP//TPSSh/def2-TZVP) =	−953.47705144
E (DLPNO-CCSD(T)/CBS//TPSSh/def2-TZVP) =	−958.038838229

N	−0.68687324	−0.51131034	1.48274714
C	−0.92142185	−1.53197868	0.65269613
N	−0.43286762	−1.20316651	−0.54470153
C	0.11797343	0.06146219	−0.48225442
C	−0.03938443	0.49763919	0.79402796
C	−0.55310236	−2.03679218	−1.74056896
C	−1.08077800	−0.48663337	2.89260978
N	−2.45336846	−3.97603998	1.22850638
C	−2.08674891	−5.21480009	1.10737980
N	−2.92618869	−6.27346560	0.93708083
C	−4.35257111	−6.07022608	0.66302766
C	−4.61057334	−4.63734254	0.22493526
C	−3.88419163	−3.69249754	1.17591880
C	−0.61282249	−5.53385275	1.16408542
C	−0.01685341	−6.05700666	−0.17294442
C	−1.03990696	−6.61682137	−1.16695157
C	−1.81986707	−7.83590034	−0.66775282
C	−2.39640470	−7.62243521	0.73999066
C	−4.56580331	−0.73841584	−1.48594404
C	−5.08749635	0.14904837	−2.53932731
C	−4.30409672	1.01367763	−3.18784632
O	−3.39527602	−0.81426811	−1.15400987
H	−5.32127167	−1.36922263	−0.97967329
H	−4.00870824	−2.65313602	0.86093203
H	−4.31351147	−3.77784185	2.18276691
H	−4.23008864	−4.48600957	−0.78999200
H	−5.68558962	−4.44492804	0.21206430
H	−4.65000579	−6.77277048	−0.11961269
H	−4.93329459	−6.31111298	1.56019748
H	−0.44365185	−6.26260527	1.96051205
H	−0.10374943	−4.62114606	1.47193943
H	−3.20860841	−8.32381209	0.93758378
H	−1.63264140	−7.81351657	1.49429544
H	−2.62572687	−8.05513981	−1.37423856
H	−1.17494598	−8.71948801	−0.64362841
H	−0.52696594	−6.87728696	−2.09643906
H	−1.75101625	−5.82319838	−1.42242007
H	0.74009741	−6.81475019	0.04639176
H	0.51070292	−5.23332444	−0.66206564
H	−0.19936297	−0.33855275	3.51521191
H	0.56897565	0.53318563	−1.33761005
H	−1.44143670	−2.48079302	0.90863061
H	−1.79283238	0.32126244	3.05706865
H	−1.54404845	−1.44179194	3.12905268
H	0.43458233	−2.20232090	−2.16907778
H	−1.20535317	−1.53922510	−2.45566222
H	−0.99656401	−2.98399468	−1.44463467
H	0.25121372	1.41974889	1.26622536
H	−6.14777263	0.07552109	−2.75755861
H	−4.69050177	1.66931285	−3.95811209
H	−3.24853957	1.08355665	−2.94922681

$\mathbf{TS_{2\text{-}IV}}$(acrolein)

E (TPSSh/def2-TZVP) =	−959.73084403
$G-E$ (TPSSh/def2-TZVP) =	0.39833982
$H-E$ (TPSSh/def2-TZVP) =	0.47473429
E (DLPNO-CCSD(T)tight/def-TZVPP//TPSSh/def2-TZVP) =	−953.41289365
E (DLPNO-CCSD(T)tight/def-QZVPP//TPSSh/def2-TZVP) =	−953.45440507
E (DLPNO-CCSD(T)/CBS//TPSSh/def2-TZVP) =	−958.019921999

C	−2.64690772	−4.97797862	0.75949087
N	−3.55129376	−4.03746073	0.56186750
C	−4.98335058	−4.23408525	0.78662999
C	−5.31463879	−5.70042244	0.56197869
C	−4.39532865	−6.57110261	1.40462892
N	−2.98794844	−6.18102013	1.21068727
C	−1.95606287	−7.21637541	1.37843017
C	−1.52575757	−7.82529977	0.03834651
C	−1.39572417	−6.77618216	−1.06963008
C	−0.60501551	−5.52266698	−0.68226070
C	−1.20803442	−4.67712744	0.47608471
O	−4.30843231	−1.44829227	−0.30413648
C	−3.65930102	−0.92243231	−1.20090178
C	−3.34220416	0.51472085	−1.25879148
C	−1.04856767	−1.41067805	−0.50630766
N	−0.04612769	−1.15842086	−1.39587813
C	0.83022778	−0.18647191	−0.94521051
C	0.37425170	0.19802035	0.27118540
N	−0.75979507	−0.55445609	0.51409737
C	0.07618044	−1.83486679	−2.67809349
C	−1.58100438	−0.43707968	1.70924598
H	−3.33593642	−1.51363941	−2.07291359
H	−5.50672630	−3.57568710	0.09731993
H	−5.23373619	−3.92347863	1.80521399
H	−5.18992066	−5.94608800	−0.49566487
H	−6.35242435	−5.89835908	0.83098102
H	−4.48265639	−7.61997447	1.11831229
H	−4.63783915	−6.49137746	2.46844462
H	−0.65019943	−4.84193149	1.40148026
H	−1.13360774	−3.61223776	0.23491672
H	−2.36921879	−7.97197838	2.04627382
H	−1.10448952	−6.76945457	1.89113705
H	−2.24335060	−8.58869958	−0.27376162
H	−0.57202610	−8.33598998	0.19738065
H	−0.92246556	−7.23689549	−1.93947731
H	−2.39619055	−6.47517686	−1.39905729
H	0.41892500	−5.79395170	−0.41476078
H	−0.53833346	−4.87999847	−1.56233642
H	−1.77169166	0.61465405	1.92428477
H	1.68364248	0.14304564	−1.51348118
H	−3.26239474	−3.13275209	0.18890558
H	−2.52650350	−0.93959559	1.51867140
H	−1.08098067	−0.89403769	2.56564983
H	0.98846432	−2.43350475	−2.71020032
H	0.09794866	−1.10683274	−3.49045483
H	−0.78995108	−2.48218539	−2.79459357
H	0.75392674	0.92783218	0.96619575
H	−2.66488876	0.83234661	−2.04461991
C	−3.89753510	1.39207630	−0.42072345
H	−3.68934423	2.45293436	−0.48455177
H	−4.59159440	1.05760483	0.34226116

5.4 Reactions: Catalyst + Amine + Aldehyde

5.4.1 Catalyst 1

I

E (TPSSh/def2-TZVP) =	−633.68726708
$G-E$ (TPSSh/def2-TZVP) =	0.26536103
$H-E$ (TPSSh/def2-TZVP) =	0.32583982
E (DLPNO-CCSD(T)/def-TZVPP//TPSSh/def2-TZVP) =	−629.44604162
E (DLPNO-CCSD(T)/def-QZVPP//TPSSh/def2-TZVP) =	−629.47352609
E (DLPNO-CCSD(T)tight/def-QZVPP//TPSSh/def2-TZVP) =	
E (DLPNO-CCSD(T)/CBS//TPSSh/def2-TZVP) =	−632.521081987

C	1.94126258	1.05687091	−1.45059645
C	1.34126512	0.38540935	−0.39044867
C	0.43612422	1.02142946	0.46942108
C	0.08852051	2.35870668	0.30949696
C	0.68690770	3.02799529	−0.74763173
C	1.59445317	2.39026752	−1.60983635
N	0.03944297	0.06201537	1.40121180
C	0.66288719	−1.08865441	1.12829099
N	1.45109500	−0.93007352	0.06021921
C	2.28947204	−1.96951573	−0.53337219
C	−0.90343029	0.27798692	2.49696073
N	0.38394256	−3.74587577	2.67275226
C	1.70216025	−4.11178143	3.19836965
C	−0.56794114	−3.56164873	3.77103321
C	−0.10409744	−4.76875390	1.74356394
H	−1.86890683	0.57946278	2.09111076
H	−0.52061723	1.05576125	3.15747455
H	−1.01140872	−0.65196399	3.04877854
H	1.98691699	−2.13474850	−1.56722053
H	3.33364462	−1.65942897	−0.49785532
H	2.15871234	−2.88484437	0.03801306
H	−0.60986574	2.85506353	0.97040851
H	0.44879393	4.07076121	−0.91404268
H	2.64093239	0.57100862	−2.11791825
H	2.03464298	2.95640385	−2.42068386
H	0.54846728	−2.03216993	1.69506059
H	1.67645374	−5.05436606	3.76430239
H	2.40773214	−4.23066927	2.37305774
H	2.06512213	−3.32253918	3.85975319
H	−0.22853996	−5.74677733	2.23078372
H	−1.06889924	−4.46226617	1.33454161
H	0.60463256	−4.88741644	0.92091252
H	−0.70800710	−4.48096720	4.35818246
H	−0.20463604	−2.78171239	4.44432146
H	−1.53940667	−3.26517645	3.36857713

II(formaldehyde)

E (TPSSh/def2-TZVP) =	−573.23937270
$G-E$ (TPSSh/def2-TZVP) =	0.16357579
$H-E$ (TPSSh/def2-TZVP) =	0.21632494
E (DLPNO-CCSD(T)/def-TZVPP//TPSSh/def2-TZVP) =	−569.58239033
E (DLPNO-CCSD(T)/def-QZVPP//TPSSh/def2-TZVP) =	−569.60683553
E (DLPNO-CCSD(T)tight/def-QZVPP//TPSSh/def2-TZVP) =	
E (DLPNO-CCSD(T)/CBS//TPSSh/def2-TZVP) =	−572.22538112

N	3.54686759	0.48411631	−2.55912591
C	2.54809347	1.06050528	−1.78242343
C	1.99039706	0.00688807	−1.04661071
N	2.69120685	−1.13164826	−1.42788890
C	3.65089150	−0.86213143	−2.35582172
C	0.94803435	0.21916905	−0.15202037
C	0.48447627	1.52438059	−0.01712241
C	1.04279006	2.57779116	−0.75194596
C	2.08533367	2.36442884	−1.64904994
C	2.42475428	−2.46369612	−0.90879145
C	4.38962910	1.21888894	−3.48222879
H	1.42516939	−2.78956076	−1.20633110
H	2.49067959	−2.45782647	0.18121613
H	3.17470288	−3.13992435	−1.30867581
H	3.78213784	1.70703872	−4.24820223
H	4.97033230	1.97692636	−2.95086523
H	5.06261685	0.50616515	−3.95239846
H	0.51377158	−0.59296810	0.41764326
H	−0.32592990	1.73092050	0.67156513
H	2.51416644	3.18224422	−2.21505650
H	0.65441573	3.58034835	−0.61860373
C	5.61494995	−2.63403550	−2.51146771
O	5.50193175	−3.42854021	−1.60202761
H	6.23275162	−1.72237224	−2.41863522
H	5.19095079	−2.81516211	−3.51303434

$\mathbf{TS_{II\text{-}III}}$(formaldehyde)

E (TPSSh/def2-TZVP) =	−573.23642760
$G-E$ (TPSSh/def2-TZVP) =	0.16624312
$H-E$ (TPSSh/def2-TZVP) =	0.21590029
E (DLPNO-CCSD(T)/def-TZVPP//TPSSh/def2-TZVP) =	−569.56150110
E (DLPNO-CCSD(T)/def-QZVPP//TPSSh/def2-TZVP) =	−569.58609049
E (DLPNO-CCSD(T)tight/def-QZVPP//TPSSh/def2-TZVP) =	
E (DLPNO-CCSD(T)/CBS//TPSSh/def2-TZVP) =	−572.217834737

N	3.44130510	0.52570235	−2.44876428
C	2.43872335	1.13156660	−1.69846098
C	1.86130210	0.10494646	−0.93990889
N	2.55225632	−1.05563872	−1.27317688
C	3.50803921	−0.80636518	−2.19347200
C	0.80474478	0.35159021	−0.07167693
C	0.35187045	1.66391959	0.01536846
C	0.93185243	2.69066866	−0.74082584
C	1.98643367	2.44294123	−1.61374669
C	2.27021176	−2.38196935	−0.73235952
C	4.30357463	1.21485001	−3.39178001
H	1.35560115	−2.77645712	−1.17982087
H	2.14248542	−2.30513514	0.34824464
H	3.12836856	−3.01581490	−0.95996334
H	3.70917077	1.65486003	−4.19529361
H	4.86397890	2.00306781	−2.88477358
H	4.99636466	0.48957748	−3.81044001
H	0.35209885	−0.44022505	0.51149640
H	−0.46922371	1.89775879	0.68208666
H	2.43107747	3.23901480	−2.19795030
H	0.54928941	3.69954740	−0.64465499
C	5.06873179	−2.03901035	−2.40482106
O	5.09052969	−2.86696907	−1.47138461
H	5.77269760	−1.17726678	−2.39470260
H	4.82253162	−2.34756276	−3.43943818

III(formaldehyde)

E (TPSSh/def2-TZVP) =	−573.24559205
$G - E$ (TPSSh/def2-TZVP) =	0.16748633
$H - E$ (TPSSh/def2-TZVP) =	0.21631146
E (DLPNO-CCSD(T)/def-TZVPP//TPSSh/def2-TZVP) =	−569.57283696
E (DLPNO-CCSD(T)/def-QZVPP//TPSSh/def2-TZVP) =	−569.59787054
E (DLPNO-CCSD(T)tight/def-QZVPP//TPSSh/def2-TZVP) =	
E (DLPNO-CCSD(T)/CBS//TPSSh/def2-TZVP) =	−572.230751897

N	3.56164736	0.50211186	−2.34254204
C	2.49470600	1.09381794	−1.67781950
C	1.91374725	0.08115851	−0.90353355
N	2.64854190	−1.07723448	−1.14190590
C	3.63603143	−0.81176310	−2.01327218
C	0.81280689	0.32455363	−0.09368962
C	0.31039671	1.62189073	−0.08826732
C	0.88896308	2.63355556	−0.86497533
C	1.99239028	2.38937893	−1.67631830
C	2.37790039	−2.38509307	−0.53140434
C	4.44658527	1.16079809	−3.28929725
H	1.37022135	−2.69846651	−0.81007888
H	2.44720949	−2.28472345	0.55321702
H	3.14982159	−3.05409593	−0.93924466
H	4.00450349	1.16855931	−4.28746867
H	4.62107870	2.18377509	−2.95730215
H	5.39231270	0.62277161	−3.31372537
H	0.36619599	−0.45885145	0.50480372
H	−0.54844711	1.85494429	0.52894812
H	2.43101732	3.17458787	−2.27886502
H	0.46592223	3.63025237	−0.83549114
C	4.67192048	−1.81327649	−2.55939977
O	4.60370028	−3.03728561	−2.10581329
H	5.64104347	−1.25656144	−2.36212280
H	4.53836446	−1.67679523	−3.67620179

2(formaldehyde)

E (TPSSh/def2-TZVP) =	−748.25420676
$G - E$ (TPSSh/def2-TZVP) =	0.28723389
$H - E$ (TPSSh/def2-TZVP) =	0.35841943
E (DLPNO-CCSD(T)/def-TZVPP//TPSSh/def2-TZVP) =	−743.37372194
E (DLPNO-CCSD(T)/def-QZVPP//TPSSh/def2-TZVP) =	−743.40619857
E (DLPNO-CCSD(T)tight/def-QZVPP//TPSSh/def2-TZVP) =	
E (DLPNO-CCSD(T)/CBS//TPSSh/def2-TZVP) =	−746.923655367

C	1.58653047	2.43294914	−1.85880318
C	1.94609458	1.13492516	−1.47295938
N	0.81825965	0.57597744	−0.87428393
C	−0.16575610	1.47709806	−0.88309330
N	0.25740655	2.60069621	−1.47154223
C	0.74388187	−0.75418608	−0.27755317
C	−0.54523595	3.80424453	−1.66862490
C	0.89092948	1.72606397	3.25498764
O	0.51537305	1.39548999	2.15555625
N	−2.95319813	1.22890595	0.43298150
C	−4.07289605	1.22260630	−0.51075450
C	−2.92385927	−0.01357256	1.20682077
C	−3.03555162	2.38135067	1.33218309
H	1.44131612	−0.81196763	0.55684067
H	−3.83127736	−0.14615541	1.81493954
C	2.48261331	3.28321557	−2.49743760
H	−1.17243016	1.33774369	−0.45320142
H	0.98982547	−1.50266640	−1.03047292
H	−0.26745004	−0.91305559	0.08526623
H	−0.09021956	4.63863137	−1.13490814
H	−0.60389809	4.03360428	−2.73247656
H	−1.54250321	3.61951114	−1.27840681
C	3.22036828	0.62894668	−1.70543476
H	−2.84486252	−0.86590251	0.52780917
H	−2.05976324	−0.00388084	1.87433072
H	−3.93718590	2.35387613	1.96230923
H	−2.15581691	2.39749824	1.97789683
H	−3.06163722	3.30354629	0.74702881
H	−5.04578075	1.14830456	−0.00230352
H	−4.06358887	2.14395266	−1.09724837
H	−3.97350230	0.37414224	−1.19042364
H	0.23744968	2.28480204	3.94864873
H	1.90515713	1.48498704	3.61957201
C	4.11530149	1.47771846	−2.34046814
H	3.50388744	−0.37222703	−1.40844325
H	5.11987948	1.12909272	−2.54325666
C	3.75370798	2.77794858	−2.72916953
H	2.20913116	4.28584344	−2.79916522
H	4.48738007	3.40198151	−3.22327607

$TS_{2\text{-}IV}$(formaldehyde)

E (TPSSh/def2-TZVP) =	−748.22247668
$G - E$ (TPSSh/def2-TZVP) =	0.29380318
$H - E$ (TPSSh/def2-TZVP) =	0.35973218
E (DLPNO-CCSD(T)/def-TZVPP//TPSSh/def2-TZVP) =	−743.32717554
E (DLPNO-CCSD(T)/def-QZVPP//TPSSh/def2-TZVP) =	−743.35960794
E (DLPNO-CCSD(T)tight/def-QZVPP//TPSSh/def2-TZVP) =	
E (DLPNO-CCSD(T)/CBS//TPSSh/def2-TZVP) =	−746.889788548

C	1.61354353	2.38457093	−1.59735273
C	1.95502039	1.03905487	−1.42512928
N	1.22687899	0.60097302	−0.31989759
C	0.45557377	1.59215732	0.19901354
N	0.70133042	2.67568075	−0.58519794
C	1.30804663	−0.74559246	0.22317438
C	0.11784109	3.99063483	−0.36425521
C	−0.08814417	2.05028745	2.48313740
O	−1.26296790	2.40986119	2.55872949
N	−2.76699527	1.32646030	0.59063043
C	−2.69668045	1.23105583	−0.89528113
C	−2.92181833	−0.01628566	1.22647959
C	−3.84204765	2.26335734	1.04268060
H	2.33172017	−0.96710127	0.52925400
H	−3.87714136	−0.44149483	0.92313400
C	2.15964272	3.15154259	−2.62145759
H	−1.87107945	1.70429662	0.95817876
H	0.99392081	−1.47657055	−0.52456245
H	0.65432489	−0.80209400	1.09031662
H	0.90633378	4.72745811	−0.20262924
H	−0.47403612	4.29403544	−1.23067464
H	−0.51438733	3.94312579	0.52015831
C	2.85678510	0.40039024	−2.27014743
H	−2.10240002	−0.64975849	0.89342534
H	−2.88146361	0.11867016	2.30467068
H	−4.80606938	1.86081220	0.73567556
H	−3.77559320	2.35182494	2.12334505
H	−3.67109308	3.23335205	0.58096022
H	−3.63141187	0.81111682	−1.26402307
H	−2.54846838	2.22873710	−1.30235751
H	−1.85709655	0.59367458	−1.16158175
H	0.73320558	2.77709183	2.39882109
H	0.22106608	1.02493538	2.73543331
C	3.40231168	1.16525810	−3.29339674
H	3.12762942	−0.63970030	−2.14052984
H	4.11074821	0.71020522	−3.97408439
C	3.06064328	2.51487505	−3.46549310
H	1.90303095	4.19445883	−2.75727158
H	3.51272964	3.07363469	−4.27524614

IV(formaldehyde)

E (TPSSh/def2-TZVP) =	−748.28025692
$G-E$ (TPSSh/def2-TZVP) =	0.29647221
$H-E$ (TPSSh/def2-TZVP) =	0.36127190
E (DLPNO-CCSD(T)/def-TZVPP//TPSSh/def2-TZVP) =	−743.38471718
E (DLPNO-CCSD(T)/def-QZVPP//TPSSh/def2-TZVP) =	−743.41694442
E (DLPNO-CCSD(T)tight/def-QZVPP//TPSSh/def2-TZVP) =	
E (DLPNO-CCSD(T)/CBS//TPSSh/def2-TZVP) =	−746.951398184

C	1.73068817	2.68799736	−1.34041527
C	1.88989933	1.30206275	−1.43571398
N	1.36262779	0.76273185	−0.26196942
C	0.91095967	1.76530490	0.51722937
N	1.11868859	2.93290191	−0.11413512
C	1.36060507	−0.66842402	0.03313458
C	0.79664372	4.26569202	0.40431423
C	0.23874355	1.64481321	1.86147813
O	−0.99116465	2.30603792	1.87714356
N	−2.95210755	1.01994185	0.58128199
C	−2.53586919	−0.09598281	−0.27310396
C	−3.89035845	0.55945833	1.61630087
C	−3.55904336	2.08683529	−0.22713030
H	2.38383184	−1.01094271	0.18877000
H	−4.80698100	0.14522580	1.17595702
C	2.15353119	3.54456532	−2.35151908
H	−1.69617330	1.76060476	1.37492494
H	0.91761462	−1.20068933	−0.80788151
H	0.77533252	−0.85802612	0.92713233
H	1.71558749	4.76685014	0.70960880
H	0.31237098	4.83534442	−0.38776101
H	0.11078385	4.15633651	1.23892952
C	2.47878340	0.70375574	−2.54460474
H	−3.41202645	−0.21101766	2.22265511
H	−4.15493711	1.39837047	2.26028601
H	−4.45945572	1.73769780	−0.74975058
H	−3.82885097	2.92114607	0.42099537
H	−2.83706045	2.43475616	−0.96837269
H	−3.38392275	−0.53957124	−0.81231626
H	−1.81050194	0.25945620	−1.00884937
H	−2.07684565	−0.87348966	0.34093757
H	0.88839245	2.11004402	2.61034365
H	0.15852032	0.58560093	2.12640454
C	2.89888520	1.55740489	−3.55433503
H	2.61009509	−0.36761484	−2.62067933
H	3.36518451	1.14044332	−4.43775663
C	2.73947719	2.94943306	−3.45921893
H	2.03995805	4.61817104	−2.28077920
H	3.08688593	3.57471838	−4.27170317

II(acetaldehyde)

E (TPSSh/def2-TZVP) =	−612.58492331
$G - E$ (TPSSh/def2-TZVP) =	0.18838098
$H - E$ (TPSSh/def2-TZVP) =	0.24567252
E (DLPNO-CCSD(T)/def-TZVPP//TPSSh/def2-TZVP) =	−608.64924075
E (DLPNO-CCSD(T)/def-QZVPP//TPSSh/def2-TZVP) =	−608.67546961
E (DLPNO-CCSD(T)tight/def-QZVPP//TPSSh/def2-TZVP) =	
E (DLPNO-CCSD(T)/CBS//TPSSh/def2-TZVP) =	−611.497436763

N	3.47156846	0.66804361	−2.48594891
C	2.41378312	1.18965954	−1.75107070
C	1.90333622	0.11227619	−1.01518526
N	2.68907671	−0.98205607	−1.35623286
C	3.66375271	−0.66852470	−2.26000925
C	0.82167442	0.26920392	−0.15634113
C	0.26847080	1.54268131	−0.05785145
C	0.77918413	2.61953648	−0.79300024
C	1.86188922	2.46182849	−1.65367906
C	2.49051278	−2.31739414	−0.81835660
C	4.28745084	1.44471150	−3.39767113
H	1.50006477	−2.68921267	−1.09245519
H	2.57546102	−2.29995881	0.27076982
H	3.25789996	−2.96714795	−1.22951414
H	3.66813781	1.89111960	−4.17976658
H	4.81258082	2.24056047	−2.86298086
H	5.00947715	0.76842503	−3.84886931
H	0.42401212	−0.56117473	0.41391104
H	−0.57540546	1.70510885	0.60196018
H	2.25371625	3.29788986	−2.22001681
H	0.32161707	3.59611161	−0.68900182
C	5.77769282	−3.00889711	−2.52790550
O	5.45356730	−3.81172052	−1.68025989
C	6.95608725	−2.08608803	−2.40934325
H	5.20761347	−2.92596421	−3.47177800
H	6.59656533	−1.05783459	−2.49799192
H	7.46734503	−2.22857252	−1.45801751
H	7.64715988	−2.26744340	−3.23984168

TS$_{\mathbf{II\text{-}III}}$(acetaldehyde)

E (TPSSh/def2-TZVP) =	−612.57781484
$G - E$ (TPSSh/def2-TZVP) =	0.19318545
$H - E$ (TPSSh/def2-TZVP) =	0.24515324
E (DLPNO-CCSD(T)/def-TZVPP//TPSSh/def2-TZVP) =	−608.61632060
E (DLPNO-CCSD(T)/def-QZVPP//TPSSh/def2-TZVP) =	−608.64265902
E (DLPNO-CCSD(T)tight/def-QZVPP//TPSSh/def2-TZVP) =	
E (DLPNO-CCSD(T)/CBS//TPSSh/def2-TZVP) =	−611.484735907

N	3.55942383	0.70104696	−2.29452473
C	2.38896647	1.18880153	−1.72267534
C	1.91899999	0.16546065	−0.88932075
N	2.82837583	−0.87920639	−1.01172101
C	3.83022459	−0.55595336	−1.85771597
C	0.75751343	0.31678545	−0.14147231
C	0.08068361	1.52590412	−0.26320798
C	0.54984344	2.54754529	−1.09893600
C	1.71493532	2.39788421	−1.84431567
C	2.73450169	−2.15990632	−0.31846542
C	4.39722622	1.44329457	−3.21923710
H	1.69234116	−2.48075793	−0.31056368
H	3.09112754	−2.05539396	0.70824127
H	3.33769384	−2.87531862	−0.88099419
H	3.81733088	1.74177903	−4.09482601
H	4.80024474	2.33379367	−2.73208418
H	5.21494654	0.79862077	−3.53000587
H	0.39391343	−0.46867171	0.50870844
H	−0.82959967	1.68236144	0.30273309
H	2.07607540	3.19023822	−2.48810337
H	−0.00598744	3.47497659	−1.16455678
C	4.85906038	−1.95939871	−2.76895572
O	4.19520309	−3.02295509	−2.71494876
C	6.20726640	−1.86487719	−2.06169184
H	4.85590909	−1.38650544	−3.72596807
H	6.60626796	−0.84564387	−2.06943063
H	6.12024586	−2.20914719	−1.02968061
H	6.91030936	−2.52417672	−2.58114980

III(acetaldehyde)

E (TPSSh/def2-TZVP) =	−612.58545928
$G - E$ (TPSSh/def2-TZVP) =	0.19513982
$H - E$ (TPSSh/def2-TZVP) =	0.24695297
E (DLPNO-CCSD(T)/def-TZVPP//TPSSh/def2-TZVP) =	−608.61954627
E (DLPNO-CCSD(T)/def-QZVPP//TPSSh/def2-TZVP) =	−608.64620378
E (DLPNO-CCSD(T)tight/def-QZVPP//TPSSh/def2-TZVP) =	
E (DLPNO-CCSD(T)/CBS//TPSSh/def2-TZVP) =	−611.493608361

N	3.46879881	0.77069907	−2.42248100
C	2.31705915	1.22513576	−1.78776289
C	1.96604143	0.23868607	−0.85762947
N	2.91273913	−0.76781947	−0.96706682
C	3.81626363	−0.44791166	−1.93160892
C	0.85217344	0.38353709	−0.04060862
C	0.10678466	1.55077275	−0.18734921
C	0.46208971	2.53635537	−1.11356157
C	1.57836597	2.39007128	−1.93434920
C	2.79723672	−2.09183556	−0.36414830
C	4.15646615	1.51929486	−3.46043686
H	1.74510759	−2.27199821	−0.14584742
H	3.37702181	−2.15178458	0.55805033
H	3.15387818	−2.79875113	−1.11783431
H	3.45279015	1.75652289	−4.25997622
H	4.56462107	2.44370094	−3.04672121
H	4.96226678	0.91025007	−3.85896810
H	0.57512516	−0.37198297	0.68315462
H	−0.76802006	1.69905134	0.43393616
H	1.85238764	3.15258525	−2.65240784
H	−0.14130210	3.43204949	−1.19437398
C	4.81112315	−1.42007824	−2.53747544
O	3.97263562	−2.32733273	−3.06557234
C	5.86498518	−1.93376645	−1.53550136
H	5.39467655	−0.83452551	−3.28897101
H	6.49185618	−1.12202971	−1.14715988
H	5.40882600	−2.46208404	−0.69688617
H	6.50042229	−2.64352596	−2.06952397

2(acetaldehyde)

E (TPSSh/def2-TZVP) =	−787.60564660
$G - E$ (TPSSh/def2-TZVP) =	0.31484731
$H - E$ (TPSSh/def2-TZVP) =	0.38814307
E (DLPNO-CCSD(T)/def-TZVPP//TPSSh/def2-TZVP) =	−782.43688793
E (DLPNO-CCSD(T)/def-QZVPP//TPSSh/def2-TZVP) =	−782.47114036
E (DLPNO-CCSD(T)tight/def-QZVPP//TPSSh/def2-TZVP) =	
E (DLPNO-CCSD(T)/CBS//TPSSh/def2-TZVP) =	−786.198903781

N	0.25488799	2.64628134	−1.12712989
C	1.62330427	2.43714711	−1.28062757
C	1.81964790	1.05150384	−1.21358141
N	0.56092341	0.48661889	−1.02266796
C	−0.34423258	1.46550070	−0.95764027
C	0.28586922	−0.93364891	−0.83355479
C	−0.40685014	3.94541805	−1.07309670
C	1.16511886	1.85365119	2.76833562
C	2.63806909	2.06262855	2.61976829
O	0.39488957	1.69428316	1.84343452
N	−3.17538892	1.12477319	0.21513490
C	−3.31505789	2.34712109	1.00956934
C	−4.34116452	0.93070543	−0.64991318
C	−2.99038270	−0.02524072	1.10270409
H	0.76739172	−1.27771103	0.08192338
H	−3.83830760	−0.15764996	1.79144278
C	2.68333483	3.31919674	−1.45853633
H	−1.41220485	1.32625592	−0.72158320
H	0.66430107	−1.49459256	−1.68759224
H	−0.78900206	−1.06947785	−0.75387160
H	−0.05407912	4.49678086	−0.20137575
H	−0.18627649	4.50429891	−1.98208765
H	−1.47823179	3.78366479	−0.99418424
C	3.08494749	0.48518133	−1.32136796
H	−2.89649220	−0.93664445	0.50762090
H	−2.08259509	0.11514570	1.69334523
H	−4.17416656	2.29936846	1.69552210
H	−2.40871452	2.50176881	1.59879443
H	−3.46373464	3.20149983	0.34515170
H	−5.27247017	0.82234960	−0.07391868
H	−4.44725191	1.78723755	−1.31829369
H	−4.20249438	0.03136887	−1.25302403
H	0.78216027	1.84929242	3.80772157
H	2.91608288	3.01307464	3.08781208
H	3.17047078	1.28337399	3.17562555
H	2.94049646	2.05405080	1.57356705
C	4.14373501	1.36562330	−1.49479210
H	3.24213867	−0.58433081	−1.27099865
H	5.14845056	0.97221373	−1.58259270
C	3.94692125	2.75446355	−1.56199903
H	2.53726231	4.39015116	−1.51171694
H	4.80427654	3.40085179	−1.70006898

$\mathbf{TS_{2\text{-}IV}}$(acetaldehyde)

E (TPSSh/def2-TZVP) =	−787.57121973
$G - E$ (TPSSh/def2-TZVP) =	0.31899388
$H - E$ (TPSSh/def2-TZVP) =	0.38889545
E (DLPNO-CCSD(T)/def-TZVPP//TPSSh/def2-TZVP) =	−782.40202110
E (DLPNO-CCSD(T)/def-QZVPP//TPSSh/def2-TZVP) =	−782.43626435
E (DLPNO-CCSD(T)tight/def-QZVPP//TPSSh/def2-TZVP) =	
E (DLPNO-CCSD(T)/CBS//TPSSh/def2-TZVP) =	−786.16618656

N	0.91069408	2.60040444	−0.92885834
C	2.02370003	2.34555325	−1.72650484
C	2.32717046	0.99522185	−1.53020471
N	1.37763883	0.52475691	−0.62640430
C	0.49238084	1.49273637	−0.24667571
C	1.34076617	−0.84483182	−0.14726930
C	0.27697313	3.90412914	−0.83603372
C	−0.06264763	2.63031518	2.55158881
C	0.41210834	1.37328921	3.19227619
O	−1.22765462	2.90317091	2.30718389
N	−3.05071388	1.38469639	1.00576623
C	−4.35586852	1.51608483	1.71599625
C	−3.07220442	2.04692580	−0.33365336
C	−2.61348802	−0.03680526	0.87965613
H	2.30062890	−1.11648719	0.29673104
H	−3.32792323	−0.57062274	0.25427479
C	2.77666278	3.14589271	−2.57907357
H	−2.32813902	1.88479787	1.58150459
H	1.12198839	−1.53437373	−0.96562766
H	0.56323354	−0.91780811	0.60952693
H	1.01143442	4.65958485	−0.54926107
H	−0.16053005	4.18782957	−1.79611779
H	−0.49933965	3.85309126	−0.07678258
C	3.39607406	0.38614891	−2.17889409
H	−1.62262186	−0.02366435	0.42734157
H	−2.58057080	−0.48237531	1.87239478
H	−5.12680072	1.01094962	1.13592818
H	−4.26579301	1.05971447	2.69986173
H	−4.59168343	2.57353006	1.81729420
H	−3.82301684	1.55796771	−0.95321994
H	−3.32042146	3.09750336	−0.19721847
H	−2.07740464	1.93872162	−0.76319043
H	0.71331989	3.38763143	2.34846447
H	1.25764045	0.97385705	2.62865566
H	0.78514743	1.62335024	4.19307588
H	−0.38059590	0.63176083	3.27774057
C	4.14932471	1.18452053	−3.03166326
H	3.63516413	−0.65974787	−2.03325704
H	4.99032536	0.75076009	−3.55805990
C	3.84554508	2.53887287	−3.22788836
H	2.54621238	4.19204648	−2.73636363
H	4.45799233	3.12473859	−3.90182083

IV(acetaldehyde)

E (TPSSh/def2-TZVP) =	−787.61392681
$G - E$ (TPSSh/def2-TZVP) =	0.32342584
$H - E$ (TPSSh/def2-TZVP) =	0.39074413
E (DLPNO-CCSD(T)/def-TZVPP//TPSSh/def2-TZVP) =	−782.42773411
E (DLPNO-CCSD(T)/def-QZVPP//TPSSh/def2-TZVP) =	−782.46170771
E (DLPNO-CCSD(T)tight/def-QZVPP//TPSSh/def2-TZVP) =	
E (DLPNO-CCSD(T)/CBS//TPSSh/def2-TZVP) =	−786.211483096

N	1.12864458	3.05876335	−0.18496994
C	1.69678466	2.61451577	−1.37247961
C	1.90442392	1.24098314	−1.22081551
N	1.44558750	0.90787626	0.05628738
C	0.99054356	2.02334095	0.66598982
C	1.52421848	−0.45541083	0.58622346
C	0.76006517	4.45118780	0.08560830
C	0.36587599	2.23232623	2.03708740
C	0.44656502	1.08187772	3.03370123
O	−0.94968402	2.69941487	1.85837361
N	−2.63600756	1.07638169	0.51307446
C	−3.89072145	1.78461727	0.81282887
C	−2.31546450	1.18371945	−0.91406232
C	−2.73511593	−0.32876931	0.91989829
H	2.41152447	−0.57172032	1.20788777
H	−3.54909691	−0.85007434	0.39785420
C	2.04043020	3.28543491	−2.54218812
H	−1.51994785	1.99835000	1.39040360
H	1.58524539	−1.13297857	−0.26141210
H	0.63428349	−0.68814893	1.15846658
H	1.64039246	5.01010407	0.40495383
H	0.36847352	4.88146281	−0.83445337
H	−0.01461180	4.46417889	0.84694016
C	2.46913257	0.47140759	−2.23365809
H	−1.79976968	−0.84375316	0.69015283
H	−2.91839321	−0.38507483	1.99381659
H	−4.73809658	1.35467421	0.26244429
H	−4.09355004	1.72065620	1.88221801
H	−3.78639904	2.83492467	0.53845195
H	−3.09677235	0.74022922	−1.54615987
H	−2.20728463	2.23598633	−1.18229287
H	−1.37452624	0.66796541	−1.11800846
H	0.92629925	3.07213456	2.46458145
H	1.46570431	0.71111295	3.16281118
H	0.10032864	1.46635099	3.99277753
H	−0.21055281	0.25758227	2.75566868
C	2.80986877	1.13826957	−3.40101669
H	2.64694674	−0.59025377	−2.12612050
H	3.25427080	0.58057055	−4.21539065
C	2.59943711	2.51835914	−3.55279679
H	1.88919782	4.35014787	−2.66007624
H	2.88557622	2.99540437	−4.48141832

5.4.2 Catalyst 2

I

E (TPSSh/def2-TZVP) =	−937.18356053
$G - E$ (TPSSh/def2-TZVP) =	0.22112365
$H - E$ (TPSSh/def2-TZVP) =	0.28048425
E (DLPNO-CCSD(T)/def-TZVPP//TPSSh/def2-TZVP) =	−932.86474587
E (DLPNO-CCSD(T)/def-QZVPP//TPSSh/def2-TZVP) =	−932.89904107
E (DLPNO-CCSD(T)tight/def-QZVPP//TPSSh/def2-TZVP) =	
E (DLPNO-CCSD(T)/CBS//TPSSh/def2-TZVP) =	−935.721803669

N	1.57007090	−1.17846917	0.22851097
C	1.41057937	0.07244796	−0.37567116
C	0.24131095	0.70646863	0.06880092
S	−0.58317312	−0.30882386	1.21729723
C	0.60701630	−1.49975912	1.07646616
C	−0.11714412	1.96764216	−0.40357511
C	0.72430356	2.56751670	−1.32585726
C	1.89354008	1.93171365	−1.76926032
C	2.25514438	0.67867425	−1.30383927
C	2.71170142	−2.05820512	−0.05661108
H	2.71544641	−2.30444585	−1.11810534
H	3.63397247	−1.54296974	0.21050609
H	2.60392468	−2.96183507	0.53613864
H	−1.01869098	2.45940346	−0.06253655
H	0.47379446	3.54733391	−1.71205736
H	3.15830217	0.19313704	−1.64957688
H	2.52733642	2.43088502	−2.49092523
H	0.58839935	−2.46412362	1.64093774
N	0.43486456	−4.05295336	2.67305384
C	1.59130893	−4.94773338	2.57822371
C	0.26225542	−3.58254816	4.05133071
C	−0.77883627	−4.72929994	2.20371533
H	1.47511650	−5.84241740	3.20664806
H	1.71838556	−5.27447810	1.54359213
H	2.49132434	−4.41967343	2.90128478
H	−1.01880125	−5.61448756	2.80975056
H	−1.62470513	−4.04035837	2.25413534
H	−0.64394963	−5.04630019	1.16775854
H	0.07840642	−4.41070940	4.75039910
H	1.16102451	−3.05253462	4.37285207
H	−0.58781465	−2.89853830	4.10125060

II(formaldehyde)

E (TPSSh/def2-TZVP) =	−876.74630145
$G - E$ (TPSSh/def2-TZVP) =	0.12170411
$H - E$ (TPSSh/def2-TZVP) =	0.17156278
E (DLPNO-CCSD(T)/def-TZVPP//TPSSh/def2-TZVP) =	−873.01455175
E (DLPNO-CCSD(T)/def-QZVPP//TPSSh/def2-TZVP) =	−873.04580689
E (DLPNO-CCSD(T)tight/def-QZVPP//TPSSh/def2-TZVP) =	
E (DLPNO-CCSD(T)/CBS//TPSSh/def2-TZVP) =	−875.437977879

N	3.13641517	−0.48628569	−2.24073555
C	2.19447943	0.37703831	−1.66209118
C	1.31711207	−0.31596321	−0.81943138
S	1.76921948	−2.00351135	−0.84025132
C	3.07875209	−1.78949004	−1.93151285
C	0.30233507	0.35775370	−0.14281578
C	0.18575402	1.72857728	−0.32610766
C	1.06408994	2.41820481	−1.16994396
C	2.07654204	1.75356368	−1.84746534
C	4.15091626	0.02770339	−3.16442461
H	3.66273364	0.45281632	−4.04378269
H	4.74066333	0.80016123	−2.66736660
H	4.79528535	−0.79791209	−3.45000988
H	−0.37747511	−0.17583225	0.50977188
H	−0.59582077	2.27221180	0.19093480
H	2.75390077	2.28990474	−2.49977822
H	0.95288908	3.48822947	−1.29698921
C	5.17939962	−3.33671545	−2.51701680
O	6.08510039	−2.64501288	−2.93168955
H	4.44817494	−3.81775383	−3.18842779
H	5.08097919	−3.59513695	−1.44793431

TS$_{\text{II-III}}$(formaldehyde)

E (TPSSh/def2-TZVP) =	−876.74183341
$G-E$ (TPSSh/def2-TZVP) =	0.12461751
$H-E$ (TPSSh/def2-TZVP) =	0.17102600
E (DLPNO-CCSD(T)/def-TZVPP//TPSSh/def2-TZVP) =	−872.98980510
E (DLPNO-CCSD(T)/def-QZVPP//TPSSh/def2-TZVP) =	−873.02119245
E (DLPNO-CCSD(T)tight/def-QZVPP//TPSSh/def2-TZVP) =	
E (DLPNO-CCSD(T)/CBS//TPSSh/def2-TZVP) =	−875.427524728

N	3.12292180	−0.37464933	−2.25885963
C	2.17204146	0.46194989	−1.65887898
C	1.32097469	−0.25359965	−0.80735651
S	1.78658405	−1.93454988	−0.83758505
C	3.06359535	−1.66887444	−1.95014444
C	0.30347771	0.39174560	−0.10834053
C	0.15382858	1.75994097	−0.28411398
C	0.99991559	2.47158398	−1.14249922
C	2.01583843	1.83462836	−1.84029432
C	4.12028878	0.14368028	−3.20671477
H	3.63995863	0.32019689	−4.17046599
H	4.52078576	1.07907875	−2.81630768
H	4.91523966	−0.60301961	−3.27814124
H	−0.35232211	−0.16047305	0.55292337
H	−0.63080759	2.28350027	0.24845023
H	2.66393368	2.38710069	−2.50792302
H	0.85927022	3.53833168	−1.26651253
C	4.58919898	−2.84748897	−2.24975305
O	5.64136169	−2.17077602	−2.33521366
H	4.16294974	−3.35119696	−3.14025781
H	4.38099493	−3.42771046	−1.32724918

2(formaldehyde)

E (TPSSh/def2-TZVP) =	−1051.75145772
$G - E$ (TPSSh/def2-TZVP) =	0.24493653
$H - E$ (TPSSh/def2-TZVP) =	0.31307628
E (DLPNO-CCSD(T)/def-TZVPP//TPSSh/def2-TZVP) =	−1046.79331820
E (DLPNO-CCSD(T)/def-QZVPP//TPSSh/def2-TZVP) =	−1046.83259694
E (DLPNO-CCSD(T)tight/def-QZVPP//TPSSh/def2-TZVP) =	
E (DLPNO-CCSD(T)/CBS//TPSSh/def2-TZVP) =	−1050.12564418

C	3.76266910	−0.49545554	−0.40617258
C	3.48668785	0.65495832	−1.12622166
C	2.14677032	0.98477082	−1.31938437
C	1.11853450	0.18361245	−0.80335311
C	1.40223774	−0.97376128	−0.08106135
C	2.73532340	−1.29939988	0.10886753
S	−0.42778550	0.87072325	−1.21285286
C	0.31696801	2.15214251	−2.02592397
N	1.63492644	2.08698239	−2.00875605
C	2.49306955	3.10762847	−2.62099826
N	−1.33341035	4.42932768	−3.13323319
C	−2.25345746	4.60422268	−2.00702874
C	−0.54393505	5.64496615	−3.34025879
C	−2.06405545	4.06768895	−4.35077295
O	1.09372696	4.41414686	−0.07507434
C	1.02092695	5.04990047	0.94963041
H	−2.96488200	5.42578988	−2.17558912
H	−0.24520975	2.98473924	−2.50646312
H	3.08031033	3.58451650	−1.83808726
H	3.14320127	2.63246977	−3.35505312
H	1.85653730	3.84353728	−3.10205379
H	−2.82446388	3.68550786	−1.85422218
H	−1.67990135	4.81819537	−1.10313536
H	−1.17582671	6.51640980	−3.56690034
H	0.03327882	5.85972296	−2.43871824
H	0.14148971	5.49778031	−4.17804001
H	−2.77762993	4.84807707	−4.65309022
H	−1.35726760	3.91091397	−5.16769231
H	−2.61794368	3.14186390	−4.18289027
H	1.82453553	5.02563767	1.70641515
H	0.14695238	5.68574965	1.17743441
H	0.61050409	−1.59508546	0.31661933
H	2.98816885	−2.19263570	0.66582410
H	4.28269334	1.27212739	−1.52160903
H	4.79348527	−0.78010977	−0.23838932

$TS_{2\text{-}IV}$(formaldehyde)

E (TPSSh/def2-TZVP) =	−1051.72716182
$G-E$ (TPSSh/def2-TZVP) =	0.24932577
$H-E$ (TPSSh/def2-TZVP) =	0.31456491
E (DLPNO-CCSD(T)/def-TZVPP//TPSSh/def2-TZVP) =	−1046.77136602
E (DLPNO-CCSD(T)/def-QZVPP//TPSSh/def2-TZVP) =	−1046.81062398
E (DLPNO-CCSD(T)tight/def-QZVPP//TPSSh/def2-TZVP) =	
E (DLPNO-CCSD(T)/CBS//TPSSh/def2-TZVP) =	−1050.10385503

N	1.99683425	1.94464292	−1.81079969
C	2.29605580	0.67755756	−1.28269838
C	1.30772355	0.25121833	−0.38875435
S	0.09016898	1.49814350	−0.29908774
C	0.86449389	2.54876769	−1.41828847
C	2.90742594	2.57895378	−2.76532744
C	1.22950266	5.45849805	−0.26818573
O	0.65996034	6.04197280	−1.16801037
N	−1.49799885	5.11249857	−2.58508576
C	−2.20743389	6.38809939	−2.90052737
C	−0.90867451	4.48350546	−3.80597966
C	−2.38181830	4.15053921	−1.86163856
H	−3.23366274	3.91276861	−2.49733318
C	3.40670500	−0.11896207	−1.55906323
H	−0.69828781	5.35929362	−1.95245209
H	3.89404918	2.69830224	−2.31442798
H	2.99413480	1.96508537	−3.66354256
H	2.49614875	3.55236507	−3.01665451
C	1.40420864	−0.98230320	0.25186158
H	−1.80016099	3.25675575	−1.64867860
H	−2.72204776	4.61480151	−0.93782020
H	−3.03125246	6.17474015	−3.57988187
H	−2.58478767	6.81594061	−1.97377438
H	−1.50080628	7.07263484	−3.36497741
H	−1.71758488	4.18864029	−4.47307313
H	−0.26439494	5.21287956	−4.29319288
H	−0.32992213	3.62186909	−3.47567020
H	2.26700110	5.71570751	−0.00401287
H	0.74098048	4.66921868	0.31904791
C	2.50905339	−1.77371910	−0.02442937
H	0.63981414	−1.31273665	0.94337145
H	2.60848535	−2.73748135	0.45914847
C	3.49783117	−1.34613076	−0.91935057
H	4.17498307	0.20353078	−2.25003976
H	4.34956774	−1.98493779	−1.11652509

IV(formaldehyde)

E (TPSSh/def2-TZVP) =	−1051.77920303
$G - E$ (TPSSh/def2-TZVP) =	0.25344103
$H - E$ (TPSSh/def2-TZVP) =	0.31575951
E (DLPNO-CCSD(T)/def-TZVPP//TPSSh/def2-TZVP) =	−1046.80813458
E (DLPNO-CCSD(T)/def-QZVPP//TPSSh/def2-TZVP) =	−1046.84722460
E (DLPNO-CCSD(T)tight/def-QZVPP//TPSSh/def2-TZVP) =	
E (DLPNO-CCSD(T)/CBS//TPSSh/def2-TZVP) =	−1050.1548848

N	2.34666689	2.41154509	−1.03819079
C	2.56992266	1.04053671	−1.19926286
C	1.49393838	0.27986678	−0.72076156
S	0.26144438	1.33426694	−0.09755516
C	1.18491746	2.71244583	−0.47061081
C	3.34050330	3.41530719	−1.45583435
C	0.70111239	4.12288367	−0.20830448
O	0.70094596	4.87275624	−1.38274361
N	−1.63802570	4.85061207	−2.62022048
C	−1.28967047	5.23728943	−3.99631434
C	−2.31106524	3.54641132	−2.60831485
C	−2.48360811	5.87971247	−1.99604730
H	−3.42288191	6.01846864	−2.54693758
C	3.69535243	0.42903434	−1.74841484
H	−0.22366681	4.81609209	−1.84236569
H	4.19835491	3.36187993	−0.78530150
H	3.64820140	3.18631272	−2.47501487
H	2.87474698	4.39500110	−1.43408320
C	1.50969695	−1.11205148	−0.78094652
H	−2.71934977	5.58516755	−0.97222234
H	−1.94241077	6.82591488	−1.97544321
H	−2.18420083	5.33859000	−4.62433589
H	−0.75959617	6.18998186	−3.97936744
H	−0.63800217	4.47805725	−4.43125274
H	−3.23941103	3.56156270	−3.19442342
H	−1.64639838	2.79043820	−3.03100135
H	−2.55727786	3.27180649	−1.58076284
H	1.37826060	4.58178940	0.52242842
H	−0.28530856	4.05677592	0.26510009
C	2.62909782	−1.71591997	−1.32985619
H	0.67928936	−1.69904694	−0.41100577
H	2.67279458	−2.79590058	−1.39028361
C	3.70658538	−0.95492097	−1.80539119
H	4.53346484	1.00896572	−2.11121362
H	4.56780412	−1.45916761	−2.22464912

II(acetaldehyde)

E (TPSSh/def2-TZVP) =	−916.09255802
$G - E$ (TPSSh/def2-TZVP) =	0.14666531
$H - E$ (TPSSh/def2-TZVP) =	0.20095889
E (DLPNO-CCSD(T)/def-TZVPP//TPSSh/def2-TZVP) =	−912.08083833
E (DLPNO-CCSD(T)/def-QZVPP//TPSSh/def2-TZVP) =	−912.11386137
E (DLPNO-CCSD(T)tight/def-QZVPP//TPSSh/def2-TZVP) =	
E (DLPNO-CCSD(T)/CBS//TPSSh/def2-TZVP) =	−914.710296583

N	3.20973204	−0.35967779	−2.03732537
C	2.20262103	0.47659402	−1.53342300
C	1.27667474	−0.24252605	−0.76789759
S	1.77594519	−1.91658397	−0.75839520
C	3.16734821	−1.66920792	−1.74107076
C	0.19260745	0.40199090	−0.17542367
C	0.05562415	1.77045391	−0.36312638
C	0.98331593	2.48666279	−1.12812538
C	2.06545589	1.85128317	−1.72111563
C	4.28405436	0.19217452	−2.86577939
H	3.85614828	0.68077004	−3.74356988
H	4.85475157	0.92260085	−2.28810056
H	4.92435947	−0.62761200	−3.17743694
H	−0.52489919	−0.15244240	0.41674472
H	−0.78029408	2.29111948	0.08854232
H	2.78108154	2.40892707	−2.31185378
H	0.85587754	3.55430133	−1.26048298
C	5.18976804	−3.43055930	−3.25020926
O	5.61594824	−2.66627663	−4.08924658
C	4.20781581	−4.53073981	−3.52837117
H	5.54176396	−3.36912137	−2.20327570
H	3.36051294	−4.42990248	−2.84611570
H	3.87045820	−4.49831380	−4.56365804
H	4.68309467	−5.49622455	−3.32077309

III(acetaldehyde)

E (TPSSh/def2-TZVP) =	−916.08310179
$G - E$ (TPSSh/def2-TZVP) =	0.15129805
$H - E$ (TPSSh/def2-TZVP) =	0.20029221
E (DLPNO-CCSD(T)/def-TZVPP//TPSSh/def2-TZVP) =	−912.04502193
E (DLPNO-CCSD(T)/def-QZVPP//TPSSh/def2-TZVP) =	−912.07815541
E (DLPNO-CCSD(T)tight/def-QZVPP//TPSSh/def2-TZVP) =	
E (DLPNO-CCSD(T)/CBS//TPSSh/def2-TZVP) =	−914.694539384

N	3.01317128	−0.36205123	−2.26633390
C	2.11442976	0.50175773	−1.62718926
C	1.28472875	−0.18772044	−0.73374537
S	1.69888882	−1.88125659	−0.77892796
C	2.93098847	−1.65312509	−1.94978045
C	0.31868184	0.48690286	0.00894449
C	0.19682797	1.85794866	−0.16640637
C	1.02062547	2.54359541	−1.06646226
C	1.98611353	1.87744469	−1.80741231
C	3.99193543	0.12194918	−3.25012431
H	3.49391885	0.27400893	−4.20894543
H	4.40717509	1.06463717	−2.89483436
H	4.77863801	−0.63559866	−3.31237190
H	−0.31994006	−0.04555937	0.70243900
H	−0.54853636	2.40388999	0.39904592
H	2.61637825	2.41036167	−2.50719859
H	0.90235619	3.61318034	−1.18935799
C	4.38882885	−2.86325342	−2.24791704
O	5.45158489	−2.19033940	−2.33551333
C	3.89339754	−3.65935717	−3.45293800
H	4.19004137	−3.40491096	−1.29627581
H	2.89869978	−4.08262130	−3.28485289
H	3.87588072	−3.03128144	−4.34598405
H	4.59997457	−4.47642957	−3.62914184

2(acetaldehyde)

E (TPSSh/def2-TZVP) =	−1091.10259378
$G - E$ (TPSSh/def2-TZVP) =	0.27172271
$H - E$ (TPSSh/def2-TZVP) =	0.34274544
E (DLPNO-CCSD(T)/def-TZVPP//TPSSh/def2-TZVP) =	−1085.85676294
E (DLPNO-CCSD(T)/def-QZVPP//TPSSh/def2-TZVP) =	−1085.89783074
E (DLPNO-CCSD(T)tight/def-QZVPP//TPSSh/def2-TZVP) =	
E (DLPNO-CCSD(T)/CBS//TPSSh/def2-TZVP) =	−1089.40013173

C	4.33657572	0.21698652	−1.38301629
C	3.68910013	1.13138007	−2.19735800
C	2.32910051	1.33821874	−1.97606623
C	1.64340262	0.64820180	−0.96677010
C	2.29864396	−0.27478732	−0.15385616
C	3.65110883	−0.47798545	−0.37585341
S	−0.02397439	1.14650126	−0.93869198
C	0.23888589	2.21689380	−2.22019157
N	1.48104343	2.20857894	−2.66270243
C	1.95651943	3.11277131	−3.71462895
C	1.98985468	4.85325582	0.30934795
O	1.61735890	4.45565747	−0.77566614
C	2.93267188	4.11565095	1.20433020
N	−1.76476818	4.37006290	−2.86700310
C	−2.83681244	4.06468288	−3.81771587
C	−2.29699088	4.50214196	−1.50862853
C	−1.05302254	5.59123134	−3.25259488
H	−3.01788385	5.32838406	−1.42432775
H	−0.53178377	2.92314821	−2.60214585
H	2.67067968	3.80844910	−3.27604514
H	2.42416000	2.52755246	−4.50555490
H	1.10334066	3.65779882	−4.10638811
H	−2.80395734	3.57779075	−1.22169180
H	−1.47462502	4.68821310	−0.81461261
H	−1.70807530	6.47469827	−3.23231126
H	−0.22017197	5.75670542	−2.56635370
H	−0.66524920	5.48284094	−4.26810643
H	−3.59435824	4.86119905	−3.85694450
H	−2.41458254	3.94055321	−4.81657248
H	−3.32929865	3.13485914	−3.52660147
H	1.62274497	5.82516233	0.69322742
H	2.44582044	3.93415971	2.16867886
H	3.25167670	3.17433078	0.75861934
H	3.80088163	4.74875123	1.41572605
H	1.77061295	−0.81249098	0.62283004
H	4.18832513	−1.18818308	0.23988941
H	4.22204086	1.66518619	−2.97314652
H	5.39341929	0.03354830	−1.52891711

TS$_{2\text{-IV}}$(acetaldehyde)

E (TPSSh/def2-TZVP) =	−1091.08011615
$G - E$ (TPSSh/def2-TZVP) =	0.27763685
$H - E$ (TPSSh/def2-TZVP) =	0.34378972
E (DLPNO-CCSD(T)/def-TZVPP//TPSSh/def2-TZVP) =	−1085.82769054
E (DLPNO-CCSD(T)/def-QZVPP//TPSSh/def2-TZVP) =	−1085.86870627
E (DLPNO-CCSD(T)tight/def-QZVPP//TPSSh/def2-TZVP) =	
E (DLPNO-CCSD(T)/CBS//TPSSh/def2-TZVP) =	−1089.37626431

C	1.26577157	2.79965453	−1.01107075
N	2.17063034	2.27847856	−1.84950724
C	2.51913581	0.92780541	−1.68401666
C	1.81509754	0.34827885	−0.62236311
S	0.77976873	1.56852163	0.07649745
C	2.79214125	3.08915850	−2.89900794
C	1.15513135	5.30511503	−0.46591844
C	2.57603495	5.39289491	−0.01651107
O	0.74999522	5.85849734	−1.49410088
N	−1.60192649	5.07453506	−2.37975372
C	−2.31672794	4.32162364	−1.30941010
C	−2.38388227	6.25571380	−2.84283496
C	−1.23191764	4.17587912	−3.51024973
H	−3.25508391	3.93629902	−1.70760344
C	3.43373203	0.18703244	−2.43123433
H	−0.67591421	5.43753484	−1.96435075
H	3.86960000	3.14458153	−2.73563413
H	2.60053484	2.64016246	−3.87513058
H	2.35773373	4.08382923	−2.85351161
C	2.00629434	−0.98937239	−0.28217887
H	−1.67830432	3.50215542	−0.98453216
H	−2.51514474	4.99718532	−0.47923247
H	−3.31911498	5.91633153	−3.28721629
H	−2.58649398	6.89704581	−1.98716976
H	−1.79255602	6.79800397	−3.57799613
H	−2.13817295	3.76853905	−3.95759408
H	−0.67647933	4.75431732	−4.24588684
H	−0.60833591	3.37678070	−3.11328339
H	0.44004745	4.91433156	0.27026602
H	2.85834987	4.52457073	0.57791854
H	3.25143941	5.52895677	−0.85986401
H	2.65431814	6.27772303	0.62834527
C	2.91580414	−1.72558721	−1.02654907
H	1.46357832	−1.43893172	0.53940892
H	3.08494392	−2.76682547	−0.78211434
C	3.62087911	−1.14345111	−2.08756642
H	3.98415394	0.63076672	−3.25093358
H	4.32655471	−1.74212893	−2.64967535

IV(acetaldehyde)

E (TPSSh/def2-TZVP) =	−1091.11940124
$G - E$ (TPSSh/def2-TZVP) =	0.28026231
$H - E$ (TPSSh/def2-TZVP) =	0.34516359
E (DLPNO-CCSD(T)/def-TZVPP//TPSSh/def2-TZVP) =	−1085.86028718
E (DLPNO-CCSD(T)/def-QZVPP//TPSSh/def2-TZVP) =	−1085.90110552
E (DLPNO-CCSD(T)tight/def-QZVPP//TPSSh/def2-TZVP) =	
E (DLPNO-CCSD(T)/CBS//TPSSh/def2-TZVP) =	−1089.42039538

C	1.62684194	3.17549294	−0.72456015
N	2.37588064	2.70915270	−1.71716182
C	2.48278589	1.31317298	−1.75070836
C	1.75564282	0.70970147	−0.71619744
S	0.97371548	1.93181687	0.23947912
C	3.06509539	3.55104645	−2.71185228
C	1.33098665	4.63684130	−0.42295087
C	2.54493604	5.31653674	0.21073105
O	0.95351731	5.31855656	−1.58327782
N	−1.52218778	4.65748165	−2.34487632
C	−2.43835160	4.54458925	−1.20272755
C	−1.92297174	5.77054889	−3.21975159
C	−1.47605205	3.39990417	−3.09888140
H	−3.46916772	4.34592637	−1.52528607
C	3.20737294	0.54697298	−2.66160668
H	−0.00121296	5.04965475	−1.84788154
H	4.13658552	3.52408046	−2.51340592
H	2.86029816	3.14154519	−3.70003694
H	2.66825777	4.55828789	−2.63970403
C	1.72563602	−0.67487261	−0.56543798
H	−2.11565918	3.72588107	−0.55595273
H	−2.42395223	5.47491723	−0.63350703
H	−2.92759853	5.61558395	−3.63454665
H	−1.91513159	6.69870766	−2.64773493
H	−1.21108602	5.85830225	−4.04143574
H	−2.45590017	3.13823720	−3.52011287
H	−0.75994709	3.49430693	−3.91710924
H	−1.15835654	2.59022001	−2.43805389
H	0.51772273	4.62419984	0.31598415
H	2.85814605	4.79921187	1.11939227
H	3.38201793	5.35009025	−0.48764764
H	2.26463408	6.33956794	0.46165784
C	2.44428984	−1.43356607	−1.47521012
H	1.16363737	−1.13998042	0.23386245
H	2.44254280	−2.51231731	−1.38450167
C	3.17582326	−0.82963400	−2.50725058
H	3.78180452	1.00486359	−3.45565998
H	3.73135504	−1.45109695	−3.19784009

5.4.3 Catalyst 3

I

E (TPSSh/def2-TZVP) =	−1207.88901992
$G - E$ (TPSSh/def2-TZVP) =	0.33729246
$H - E$ (TPSSh/def2-TZVP) =	0.41063707
E (DLPNO-CCSD(T)/def-TZVPP//TPSSh/def2-TZVP) =	−1201.81842352
E (DLPNO-CCSD(T)/def-QZVPP//TPSSh/def2-TZVP) =	−1201.86458523
E (DLPNO-CCSD(T)tight/def-QZVPP//TPSSh/def2-TZVP) =	
E (DLPNO-CCSD(T)/CBS//TPSSh/def2-TZVP) =	−1205.96904173

C	2.57843601	−1.68014713	−3.14150239
N	3.44084404	−0.89050331	−2.51255377
C	4.40021196	−0.29264611	−3.32866035
C	4.24702383	−0.66363673	−4.63480660
S	2.90553648	−1.74745527	−4.78538159
C	3.30371517	−0.62629585	−1.06411088
C	2.15574908	0.30749348	−0.75577442
C	1.64873255	1.19687626	−1.70040702
C	0.59730296	2.04570612	−1.36775753
C	0.05038700	2.01337825	−0.09001327
C	0.55895874	1.12978638	0.85795631
C	1.60632848	0.27892383	0.52622058
C	5.42447522	0.62991285	−2.76051351
C	5.07766233	−0.29189014	−5.82007975
C	6.18880497	−1.30755922	−6.09939457
O	6.88787019	−0.80883456	−7.22269237
H	4.25623398	−0.22079628	−0.72363559
H	3.16007952	−1.58929482	−0.57429731
H	6.09800441	0.10484446	−2.07802550
H	6.02603993	1.04921580	−3.56452373
H	4.95751087	1.45556490	−2.21807757
H	4.45277206	−0.20605703	−6.71169448
H	5.53122457	0.68758502	−5.65585012
H	6.84110060	−1.39654110	−5.22014951
H	5.74918521	−2.29425452	−6.29891539
H	7.59093439	−1.42553027	−7.46266221
H	1.99572474	−0.41129463	1.26748859
H	0.13627768	1.09794865	1.85471052
H	−0.76961210	2.67253207	0.16677087
H	0.20637640	2.73124024	−2.10972550
H	2.05843487	1.23067163	−2.70395897
H	1.71483321	−2.16783547	−2.62880209
N	0.12313857	−2.83420416	−1.85707686
C	−0.28482036	−3.91518719	−2.75766518
C	0.31717447	−3.33997621	−0.49726654
C	−0.86000581	−1.74687450	−1.87118054
H	−1.24344346	−4.36372715	−2.45938872
H	−0.39249582	−3.52472380	−3.77230279
H	0.47616548	−4.69843193	−2.75880768
H	−0.60246552	−3.77829332	−0.08261112
H	1.09276378	−4.10902340	−0.49882990
H	0.62663275	−2.51956453	0.15284009
H	−1.85234091	−2.08531993	−1.53946575
H	−0.52525318	−0.94427597	−1.21304846
H	−0.95241433	−1.35313542	−2.88551839

II(formaldehyde)

E (TPSSh/def2-TZVP) =	−1147.44316636
$G - E$ (TPSSh/def2-TZVP) =	0.23538145
$H - E$ (TPSSh/def2-TZVP) =	0.30185033
E (DLPNO-CCSD(T)/def-TZVPP//TPSSh/def2-TZVP) =	−1141.96120918
E (DLPNO-CCSD(T)/def-QZVPP//TPSSh/def2-TZVP) =	−1142.00441979
E (DLPNO-CCSD(T)tight/def-QZVPP//TPSSh/def2-TZVP) =	
E (DLPNO-CCSD(T)/CBS//TPSSh/def2-TZVP) =	−1145.67661177

C	1.50414291	1.51007631	−1.77092886
C	2.17444794	0.53635512	−1.03120971
C	1.83493573	0.34476431	0.30654772
C	0.83784019	1.11218410	0.90045049
C	0.17667437	2.08549369	0.15924250
C	0.51295740	2.28186607	−1.17757914
C	3.23143207	−0.32640772	−1.67846331
N	4.13181690	0.45188004	−2.54658755
C	5.07966749	1.33800967	−2.00719150
C	5.74311039	2.01014688	−2.98202678
S	5.13341205	1.47716293	−4.52482557
C	3.99956154	0.37541903	−3.88284978
C	5.27209355	1.44609307	−0.53143503
C	6.84986450	3.00560621	−2.83771836
C	8.22475709	2.36485800	−3.00031588
O	9.19068036	3.39980598	−2.83270458
C	1.97495603	−0.95827976	−4.92751757
O	1.26350324	−1.38704277	−4.04283136
H	3.82758850	−0.83343155	−0.91714118
H	2.77017712	−1.07614145	−2.32011205
H	2.79147637	−1.55330330	−5.36994194
H	1.78766283	0.01643900	−5.41147384
H	5.60717447	0.49598065	−0.10430341
H	6.02910718	2.19711797	−0.30849041
H	4.34382124	1.72799010	−0.02927412
H	6.74717954	3.80067653	−3.58050550
H	6.80063631	3.48103552	−1.85475950
H	8.35564895	1.57611613	−2.24787426
H	8.30466626	1.90502140	−3.99337908
H	10.06868901	3.02379299	−2.96829798
H	2.35467896	−0.41074184	0.88755598
H	0.58206730	0.95259171	1.94162520
H	−0.59734438	2.68816592	0.62000700
H	−0.00297156	3.03524088	−1.76164645
H	1.75922817	1.65654619	−2.81406820

TS$_{\text{II-III}}$(formaldehyde)

E (TPSSh/def2-TZVP) =	−1147.44063891
$G-E$ (TPSSh/def2-TZVP) =	0.23798350
$H-E$ (TPSSh/def2-TZVP) =	0.30132042
E (DLPNO-CCSD(T)/def-TZVPP//TPSSh/def2-TZVP) =	−1141.94031817
E (DLPNO-CCSD(T)/def-QZVPP//TPSSh/def2-TZVP) =	−1141.98366474
E (DLPNO-CCSD(T)tight/def-QZVPP//TPSSh/def2-TZVP) =	
E (DLPNO-CCSD(T)/CBS//TPSSh/def2-TZVP) =	−1145.66799333

C	1.45611728	1.69745405	−1.68751058
C	2.09758188	0.67500042	−0.98760653
C	1.74582163	0.44265376	0.34184486
C	0.76749329	1.21298211	0.96203535
C	0.13597323	2.23367900	0.25951042
C	0.48440627	2.47340404	−1.06684113
C	3.13523390	−0.19467085	−1.65204043
N	4.05076541	0.59852390	−2.49653369
C	5.07765851	1.37864394	−1.94554401
C	5.73200019	2.09296795	−2.89726618
S	5.02291049	1.73992265	−4.44778653
C	3.86375446	0.66618395	−3.81705018
C	5.33713651	1.35487913	−0.47728761
C	6.90496315	3.00662961	−2.73857437
C	8.22604058	2.30631977	−3.04420496
O	9.25834038	3.26848801	−2.85120518
C	2.82021510	−0.73062296	−4.76119259
O	2.78375033	−1.77079493	−4.06395794
H	3.73156586	−0.71530252	−0.90189681
H	2.69341831	−0.94238083	−2.32501308
H	3.47791043	−0.68355591	−5.65148595
H	1.91699322	−0.10315815	−4.89133216
H	5.67705425	0.36765841	−0.15093449
H	6.11209354	2.07708936	−0.22418527
H	4.43342403	1.60205552	0.08480153
H	6.80838088	3.87121361	−3.40007068
H	6.94147231	3.39117816	−1.71643844
H	8.35279346	1.44742014	−2.37243954
H	8.21860289	1.93327870	−4.07621763
H	10.10440929	2.85899706	−3.06865070
H	2.24173388	−0.34902075	0.89460320
H	0.50416042	1.01908706	1.99549234
H	−0.62229988	2.83928956	0.74186543
H	−0.00508853	3.26553421	−1.62177553
H	1.71950904	1.88483279	−2.72275994

III(formaldehyde)

E (TPSSh/def2-TZVP) =	−1147.45080304
$G - E$ (TPSSh/def2-TZVP) =	0.24030331
$H - E$ (TPSSh/def2-TZVP) =	0.30315494
E (DLPNO-CCSD(T)/def-TZVPP//TPSSh/def2-TZVP) =	−1141.94455006
E (DLPNO-CCSD(T)/def-QZVPP//TPSSh/def2-TZVP) =	−1141.98823800
E (DLPNO-CCSD(T)tight/def-QZVPP//TPSSh/def2-TZVP) =	
E (DLPNO-CCSD(T)/CBS//TPSSh/def2-TZVP) =	−1145.67884888

C	1.44668568	1.52229981	−1.66555308
C	2.16581388	0.54778588	−0.97118812
C	1.96717426	0.37921698	0.39774975
C	1.05814375	1.18169183	1.08014447
C	0.35163255	2.16317543	0.39269372
C	0.54819421	2.32917561	−0.97648414
C	3.14703229	−0.33671000	−1.70345448
N	4.11528592	0.42228736	−2.51604708
C	5.02164932	1.33548547	−1.96208905
C	5.76055928	1.96962576	−2.90501361
S	5.32391434	1.39535860	−4.49295544
C	4.11007098	0.37185766	−3.86448147
C	5.11440613	1.50691962	−0.48339761
C	6.83851623	2.98923807	−2.72734973
C	8.23079794	2.41842616	−2.98031784
O	9.15811812	3.47871170	−2.76830130
C	3.04687974	−0.23577458	−4.72486136
O	2.16154940	0.77597021	−4.58702070
H	3.70579958	−0.95386950	−0.99618764
H	2.63024437	−0.99942368	−2.39501041
H	2.74013472	−1.23881270	−4.35256115
H	3.45419701	−0.40295643	−5.74443840
H	5.23345830	0.54264440	0.01784616
H	5.97907462	2.12081164	−0.23712849
H	4.21804248	1.98077535	−0.07903890
H	6.67987171	3.83266511	−3.40521744
H	6.80323012	3.38956723	−1.71237140
H	8.41787727	1.58327364	−2.29263527
H	8.29711932	2.03511267	−4.00681127
H	10.04580878	3.15285999	−2.95941592
H	2.53026255	−0.37808254	0.93523626
H	0.90757056	1.04413804	2.14477460
H	−0.35313690	2.79434315	0.92207271
H	−0.00992749	3.08642004	−1.51526705
H	1.59095497	1.60546104	−2.74152930

2(formaldehyde)

E (TPSSh/def2-TZVP) =	−1322.45554077
$G - E$ (TPSSh/def2-TZVP) =	0.36083260
$H - E$ (TPSSh/def2-TZVP) =	0.43754577
E (DLPNO-CCSD(T)/def-TZVPP//TPSSh/def2-TZVP) =	−1315.74561204
E (DLPNO-CCSD(T)/def-QZVPP//TPSSh/def2-TZVP) =	−1315.79684587
E (DLPNO-CCSD(T)tight/def-QZVPP//TPSSh/def2-TZVP) =	
E (DLPNO-CCSD(T)/CBS//TPSSh/def2-TZVP) =	−1320.37084144

C	1.83505272	1.13863245	−4.60156867
C	2.37057327	2.29078542	−4.02811614
C	2.70769631	3.36960186	−4.84462763
C	2.51375572	3.29679102	−6.21958602
C	1.97388326	2.14684253	−6.78755747
C	1.63428214	1.06913461	−5.97607215
C	2.64093469	2.39374512	−2.54635386
N	1.68696092	1.59753379	−1.74133722
C	2.04156713	0.49900969	−0.95911689
C	0.95409397	−0.05042587	−0.34283275
S	−0.47063484	0.83893459	−0.76374911
C	0.39568226	1.89588924	−1.73561173
C	3.45777974	0.04187261	−0.86735770
C	0.89414306	−1.20834521	0.60051316
C	0.98577453	−0.77541085	2.06541834
O	0.93685346	−1.96894655	2.82367069
N	−1.00266139	4.03819849	−3.32067269
C	−2.26410399	4.16588951	−2.59101857
C	−0.29808731	5.31888511	−3.36492222
C	−1.22696604	3.51740231	−4.67083697
O	1.48150907	4.28854981	−0.11439928
C	1.50533218	5.13135570	0.75016741
H	−2.95276572	4.87875970	−3.06842306
H	−0.04893153	2.72839001	−2.32458149
H	3.63765099	2.03001691	−2.30011120
H	2.56567013	3.42275026	−2.19746522
H	−2.76047152	3.19358134	−2.54448837
H	−2.06349337	4.50912822	−1.57356950
H	−0.88598605	6.09889044	−3.87191912
H	−0.08390455	5.65101284	−2.34736481
H	0.64308074	5.19373048	−3.90231299
H	−1.86558549	4.18316613	−5.27041892
H	−0.26873354	3.39989259	−5.17792207
H	−1.71162105	2.54086605	−4.60806339
H	2.45456770	5.55498189	1.12352100
H	0.57769425	5.51901842	1.20807034
H	4.09194401	0.80394356	−0.40721105
H	3.51356562	−0.85474867	−0.25310392
H	3.86515332	−0.19657831	−1.85297256
H	−0.03407842	−1.76660490	0.46035448
H	1.71281792	−1.89843892	0.38700324
H	1.92202751	−0.22580027	2.23261185
H	0.14952938	−0.10568715	2.30801394
H	0.96529688	−1.75251345	3.76403477
H	3.12463783	4.26953156	−4.40478432
H	2.77758769	4.14081837	−6.84521084
H	1.81664204	2.09168623	−7.85770854
H	1.21389137	0.17121593	−6.41249133
H	1.56453498	0.29080835	−3.98142846

$\mathbf{TS_{2\text{-}IV}}$ (formaldehyde)

E (TPSSh/def2-TZVP) =	−1322.43462495
$G-E$ (TPSSh/def2-TZVP) =	0.36773688
$H-E$ (TPSSh/def2-TZVP) =	0.44549908
E (DLPNO-CCSD(T)/def-TZVPP//TPSSh/def2-TZVP) =	−1315.70868185
E (DLPNO-CCSD(T)/def-QZVPP//TPSSh/def2-TZVP) =	−1315.75971744
E (DLPNO-CCSD(T)tight/def-QZVPP//TPSSh/def2-TZVP) =	
E (DLPNO-CCSD(T)/CBS//TPSSh/def2-TZVP) =	−1320.34942631

N	1.90100551	1.80337817	−1.33162837
C	2.01170017	0.49186299	−0.84687035
C	0.90878535	0.12926350	−0.13532716
S	−0.18844717	1.47507248	−0.09918933
C	0.78869405	2.49910978	−1.03760484
C	2.92884687	2.42240125	−2.19315615
C	1.07824301	4.85671173	−0.67953107
O	1.11366947	5.44680097	−1.75588371
N	−0.83452885	4.28318249	−3.32250993
C	−0.69221680	5.41445937	−4.28830653
C	−1.00623039	2.97483377	−4.01732119
C	−1.93643779	4.54011805	−2.34915957
H	−2.87686800	4.60001334	−2.89458699
C	3.23661365	−0.32897138	−1.08392934
H	0.03755499	4.26383124	−2.76276810
H	3.86179826	1.87883179	−2.04400417
C	2.53733634	2.43044187	−3.65102256
H	3.07485994	3.44342617	−1.84662812
C	0.63200494	−1.15748717	0.57340951
H	−1.96094170	3.71883589	−1.63654570
H	−1.72805171	5.47901690	−1.84150244
H	−1.62943997	5.52791381	−4.83089591
H	−0.45463437	6.31047529	−3.72073738
H	0.11872572	5.17567860	−4.97189421
H	−1.94065940	3.00096228	−4.57647694
H	−0.16323211	2.82520344	−4.68721521
H	−1.03029436	2.19082981	−3.26432133
H	1.99768366	4.53841575	−0.16354419
H	0.16523909	4.80963719	−0.06683624
H	4.10321934	0.09732261	−0.57045920
H	3.08464411	−1.33514495	−0.69646391
H	3.48198142	−0.40996941	−2.14537923
C	1.04105207	−1.11460377	2.04595025
H	−0.43144190	−1.40096402	0.51746007
H	1.16972060	−1.97412909	0.08606537
O	0.72984060	−2.39142480	2.57747327
H	2.11401037	−0.89466189	2.12705134
H	0.48992768	−0.31725296	2.56189021
H	0.95630172	−2.40805323	3.51560652
C	2.64315460	3.61492860	−4.37964388
C	2.34815135	3.63338585	−5.74145868
C	1.93419914	2.46992320	−6.38275548
C	1.81270606	1.28700317	−5.65594181
C	2.11283755	1.26818316	−4.29881014
H	2.95978623	4.52319815	−3.87861528
H	2.45541539	4.55461189	−6.30275506
H	1.71343518	2.48111062	−7.44326195
H	1.49215888	0.37724094	−6.14936396
H	2.00926818	0.34220358	−3.74450793

IV(formaldehyde)

E (TPSSh/def2-TZVP) =	−1322.48365074
$G - E$ (TPSSh/def2-TZVP) =	0.36854445
$H - E$ (TPSSh/def2-TZVP) =	0.44602162
E (DLPNO-CCSD(T)/def-TZVPP//TPSSh/def2-TZVP) =	−1315.75897601
E (DLPNO-CCSD(T)/def-QZVPP//TPSSh/def2-TZVP) =	−1315.80992934
E (DLPNO-CCSD(T)tight/def-QZVPP//TPSSh/def2-TZVP) =	
E (DLPNO-CCSD(T)/CBS//TPSSh/def2-TZVP) =	−1320.40143916

N	2.07700385	2.01600809	−1.07868622
C	2.17897973	0.64078773	−0.85252775
C	1.15126302	0.17168760	−0.08834022
S	0.09206162	1.47339036	0.33057417
C	1.02753279	2.60489951	−0.50629743
C	3.02447449	2.73141713	−1.95860404
C	0.71524974	4.08757716	−0.54259948
O	0.86160059	4.64333357	−1.80570541
N	−1.28347536	4.44157307	−3.35005608
C	−0.71533562	4.78948878	−4.66159426
C	−1.88523558	3.10492030	−3.38747684
C	−2.26961308	5.44617581	−2.92973240
H	−3.10494853	5.51508053	−3.63921557
C	3.32649503	−0.13450295	−1.40430107
H	0.00542060	4.51313000	−2.36218845
H	4.01427955	2.32569687	−1.74243919
C	2.68321313	2.58165225	−3.42627806
H	3.00631755	3.78072690	−1.67757204
C	0.90312872	−1.21681131	0.40508086
H	−2.66702452	5.17867264	−1.94904014
H	−1.78449725	6.42027803	−2.86021018
H	−1.48841961	4.81186484	−5.44126545
H	−0.24758167	5.77253503	−4.59970018
H	0.04305597	4.05464265	−4.93193860
H	−2.71107921	3.04894075	−4.10964728
H	−1.12437018	2.37679669	−3.67106703
H	−2.27428575	2.85128524	−2.39914336
H	1.41452679	4.58092973	0.14514773
H	−0.28902782	4.21814801	−0.11876617
H	4.26174683	0.14929618	−0.91375220
H	3.16848708	−1.19713387	−1.23121385
H	3.43670623	0.02918244	−2.47815879
C	1.47186377	−1.45330765	1.80660314
H	−0.16834525	−1.42847398	0.42487229
H	1.35561508	−1.93423587	−0.28226952
O	1.17254441	−2.80004540	2.11760762
H	2.55409585	−1.26660019	1.80502984
H	1.00668267	−0.75867573	2.51920198
H	1.49590402	−3.00380925	3.00423595
C	3.37658662	3.38024745	−4.33617504
C	3.12857319	3.26924032	−5.69811325
C	2.18579044	2.35765978	−6.16688576
C	1.48837984	1.56520108	−5.26360310
C	1.73401990	1.67890660	−3.89720135
H	4.10823171	4.09681328	−3.97855056
H	3.67170760	3.89652180	−6.39438269
H	1.99579446	2.26875890	−7.22940500
H	0.75051328	0.85535515	−5.61826194
H	1.17304929	1.05595096	−3.20941762

II(acetaldehyde)

E (TPSSh/def2-TZVP) =	−1186.78951011
$G-E$ (TPSSh/def2-TZVP) =	0.26042599
$H-E$ (TPSSh/def2-TZVP) =	0.33123462
E (DLPNO-CCSD(T)/def-TZVPP//TPSSh/def2-TZVP) =	−1181.02754878
E (DLPNO-CCSD(T)/def-QZVPP//TPSSh/def2-TZVP) =	−1181.07254535
E (DLPNO-CCSD(T)tight/def-QZVPP//TPSSh/def2-TZVP) =	
E (DLPNO-CCSD(T)/CBS//TPSSh/def2-TZVP) =	−1184.94930132

C	4.56775120	4.12703156	−0.56217368
N	3.95317479	4.02266075	−1.82145432
C	3.71623402	5.14083124	−2.53721525
S	4.31328219	6.42123621	−1.57348384
C	4.84454478	5.41844373	−0.25007001
C	3.47669327	2.72960736	−2.32598058
C	2.21352515	2.26739139	−1.63609577
C	1.29432222	3.17930724	−1.11997457
C	0.11930317	2.73277564	−0.52461571
C	−0.15182701	1.37082103	−0.44193752
C	0.76173120	0.45576789	−0.95623963
C	1.93857169	0.90329984	−1.54560710
C	1.92365953	4.67563894	−5.01500196
C	0.75743351	5.33812693	−4.34001218
C	5.53139537	5.95584618	0.96458027
C	7.01273793	6.22169094	0.71592793
O	7.55939842	6.72133095	1.93441792
C	4.85005961	2.90706973	0.24999863
O	2.01009481	3.48186172	−5.20770380
H	4.26703772	1.98591282	−2.20284497
H	3.29799389	2.86865581	−3.39161520
H	2.71508761	5.36093412	−5.37190184
H	1.12812941	5.99484494	−3.54994163
H	0.23393863	5.96496178	−5.07143520
H	0.07195257	4.59526247	−3.93368345
H	5.26471770	3.18920974	1.21723430
H	3.94101535	2.32468696	0.41951502
H	5.57559948	2.25674975	−0.24837397
H	5.06158384	6.88820028	1.28823328
H	5.43845946	5.24841156	1.79273878
H	7.50889520	5.29052076	0.41251927
H	7.12729395	6.94935159	−0.09728306
H	8.48983505	6.93050447	1.78899601
H	2.65038408	0.18669623	−1.94303197
H	0.56035821	−0.60754748	−0.89479076
H	−1.06755961	1.02409725	0.02256908
H	−0.58673655	3.45218490	−0.12555649
H	1.49921815	4.24126578	−1.18606003

TS$_{\text{II-III}}$(acetaldehyde)

E (TPSSh/def2-TZVP) = −1186.78163786
$G-E$ (TPSSh/def2-TZVP) = 0.26510069
$H-E$ (TPSSh/def2-TZVP) = 0.33067079
E (DLPNO-CCSD(T)/def-TZVPP//TPSSh/def2-TZVP) = −1180.99479395
E (DLPNO-CCSD(T)/def-QZVPP//TPSSh/def2-TZVP) = −1181.03971653
E (DLPNO-CCSD(T)tight/def-QZVPP//TPSSh/def2-TZVP) =
E (DLPNO-CCSD(T)/CBS//TPSSh/def2-TZVP) = −1184.93576224

C	4.33630168	4.02652940	−0.59960606
N	3.69933497	3.91594673	−1.84651862
C	3.52388431	5.04229387	−2.54367947
S	4.14875634	6.32944847	−1.62734558
C	4.65278927	5.31523435	−0.30862870
C	3.23120558	2.61209940	−2.38590653
C	2.18501653	1.96735120	−1.51417961
C	1.00577811	2.65560410	−1.22359350
C	0.03357452	2.05974733	−0.42934586
C	0.22151106	0.77254122	0.06883151
C	1.38579189	0.07582555	−0.23732992
C	2.36394231	0.67505973	−1.02471444
C	2.29314027	5.12942050	−4.06214170
C	1.98243498	6.62316909	−4.05372201
C	5.35183465	5.85517123	0.89861627
C	6.83409267	6.11018620	0.64116210
O	7.38517177	6.61200570	1.85475735
C	4.62021462	2.82557729	0.23868251
O	1.36562132	4.30340726	−3.85408912
H	4.10328491	1.96496373	−2.49883337
H	2.79498457	2.84462386	−3.35874148
H	3.07363480	4.88639384	−4.81536980
H	2.89046594	7.23330414	−4.09709757
H	1.37951848	6.84050886	−4.94027647
H	1.39702321	6.88845133	−3.17163709
H	5.28814085	3.09731803	1.05515969
H	3.70542664	2.40237749	0.65723099
H	5.10600564	2.04199822	−0.34808213
H	4.88871111	6.79202645	1.21865969
H	5.25486209	5.15435195	1.73078541
H	7.32325320	5.17448388	0.33994243
H	6.95025104	6.83319577	−0.17635671
H	8.31744335	6.81313523	1.70931120
H	3.27665188	0.13474533	−1.25720498
H	1.53457024	−0.93031516	0.13765888
H	−0.54105686	0.31091183	0.68564576
H	−0.88204752	2.59691007	−0.20992323
H	0.84825159	3.63418454	−1.66385487

2(acetaldehyde)

E (TPSSh/def2-TZVP) =	−1361.80666741
$G - E$ (TPSSh/def2-TZVP) =	0.38740988
$H - E$ (TPSSh/def2-TZVP) =	0.46720855
E (DLPNO-CCSD(T)/def-TZVPP//TPSSh/def2-TZVP) =	−1354.81088446
E (DLPNO-CCSD(T)/def-QZVPP//TPSSh/def2-TZVP) =	−1354.86387650
E (DLPNO-CCSD(T)tight/def-QZVPP//TPSSh/def2-TZVP) =	
E (DLPNO-CCSD(T)/CBS//TPSSh/def2-TZVP) =	−1359.64612313

C	1.24027178	1.57702535	−5.65100040
C	2.00252570	2.29276863	−4.72558894
C	2.12321113	3.67601273	−4.85660618
C	1.49884765	4.33597440	−5.90957629
C	0.74552503	3.61688397	−6.83223771
C	0.61154290	2.23717118	−6.69922677
C	2.74438238	1.57775035	−3.63686289
N	1.91113672	1.26939126	−2.43825960
C	0.68046798	1.71570760	−2.25617371
S	0.07045507	1.24860069	−0.76135367
C	1.53295896	0.42519860	−0.35020053
C	2.43241759	0.54344524	−1.36833529
C	3.82997449	0.03129251	−1.41096038
C	1.70780160	−0.22678136	0.98287905
C	2.17168167	0.76405464	2.05312928
O	2.31084545	0.01157046	3.24326788
N	−1.54620078	3.71272580	−3.06462793
C	−0.97972067	5.05684262	−2.96642549
C	−2.20257639	3.52101867	−4.35866875
C	−2.49413776	3.48069629	−1.97581561
O	3.10605643	3.58967728	−1.12972495
C	3.20089444	4.61846550	−0.49344619
C	2.17102278	5.11393684	0.47066047
H	−3.04606207	4.21447878	−4.50253736
H	0.10535263	2.34959484	−2.93431680
H	3.13021760	0.62114903	−3.99110389
H	3.57490403	2.18003187	−3.26869969
H	−1.47916992	3.67874044	−5.15830915
H	−2.58187519	2.49939518	−4.42626641
H	−3.35032100	4.17254311	−2.01285075
H	−2.87891772	2.46003873	−2.03321529
H	−1.99232390	3.61742202	−1.01458195
H	−1.75141627	5.84101215	−3.01968830
H	−0.45102686	5.16030430	−2.01539173
H	−0.27041771	5.21124084	−3.78012210
H	4.09734928	5.25848418	−0.61144757
H	2.62906643	5.24271869	1.45707897
H	1.83311790	6.10835601	0.15976252
H	1.32541081	4.43017618	0.53225016
H	4.05290875	−0.51028870	−0.49396713
H	3.98673513	−0.64732046	−2.25293738
H	4.53913986	0.85813125	−1.49991072
H	0.77189844	−0.68315009	1.31255647
H	2.44240629	−1.03050646	0.90618901
H	3.12140738	1.22296455	1.74722951
H	1.42709333	1.56467085	2.16462798
H	2.60029662	0.59180698	3.95833543
H	2.72221677	4.23047409	−4.14253662
H	1.61117757	5.40810866	−6.01776690
H	0.26894071	4.12931746	−7.65929543
H	0.03080389	1.67523575	−7.42042996
H	1.15476707	0.49881750	−5.56285131

$\mathbf{TS_{2\text{-}IV}}$(acetaldehyde)

E (TPSSh/def2-TZVP) =	−1361.78281820
$G-E$ (TPSSh/def2-TZVP) =	0.39228825
$H-E$ (TPSSh/def2-TZVP) =	0.47455564
E (DLPNO-CCSD(T)/def-TZVPP//TPSSh/def2-TZVP) =	−1354.78370814
E (DLPNO-CCSD(T)/def-QZVPP//TPSSh/def2-TZVP) =	−1354.83654054
E (DLPNO-CCSD(T)tight/def-QZVPP//TPSSh/def2-TZVP) =	
E (DLPNO-CCSD(T)/CBS//TPSSh/def2-TZVP) =	−1359.62508545

C	0.90560688	2.64630917	−0.99810222
N	1.97587072	2.06929534	−1.58107198
C	2.29791062	0.74165858	−1.25367490
C	1.42638500	0.23085090	−0.34388799
S	0.26300314	1.46482852	0.04700725
C	2.71079790	2.80628495	−2.61294071
C	1.14494562	5.45502739	−0.55702377
C	2.47133950	5.50175017	0.12178716
O	0.95841864	5.79974406	−1.71601194
N	−1.18877178	4.70649522	−3.04702985
C	−1.80387369	3.35993069	−2.86843848
C	−2.11635601	5.79684356	−2.62281244
C	−0.71837036	4.91069539	−4.45126951
H	−2.69403050	3.30344735	−3.49447252
C	3.47408182	0.06157135	−1.87243133
H	−0.34646919	4.80022524	−2.43550409
H	3.76048531	2.50777559	−2.57568216
C	2.15133835	2.59895555	−4.00335366
H	2.65222786	3.86111187	−2.35003693
C	1.42594338	−1.12118057	0.29358921
H	−1.07533453	2.61075194	−3.16441126
H	−2.06136416	3.22946055	−1.82061248
H	−2.98813123	5.79014108	−3.27517895
H	−2.41670094	5.61669344	−1.59251328
H	−1.58796863	6.74433500	−2.69690311
H	−1.58432042	4.90530806	−5.11159981
H	−0.20411666	5.86768529	−4.50223154
H	−0.03736025	4.10336426	−4.70934709
H	0.29745305	5.14567648	0.07514789
H	2.42947599	6.25568731	0.91635782
H	2.66344213	4.54265441	0.60991966
H	3.26902177	5.75166887	−0.57594479
H	3.49691325	−0.98493056	−1.57236258
H	3.43335869	0.10130914	−2.96403134
H	4.41441019	0.51937392	−1.55161715
C	2.13811525	−1.13124897	1.64547584
H	0.40186726	−1.47223161	0.44055264
H	1.91773414	−1.84288487	−0.36310433
O	2.06487000	−2.46532696	2.12308172
H	3.17954563	−0.80656757	1.51807705
H	1.64388238	−0.43074762	2.33148800
H	2.48856364	−2.51448539	2.98881027
C	2.60762702	3.42020395	−5.03709275
C	2.11641327	3.26989628	−6.32837569
C	1.15917990	2.29529959	−6.60386720
C	0.70380120	1.47158325	−5.58051059
C	1.19770169	1.62219948	−4.28572421
H	3.34887943	4.18482074	−4.82855357
H	2.48122462	3.91261897	−7.12070887
H	0.77905188	2.17484135	−7.61100107
H	−0.03117364	0.70263609	−5.78804580
H	0.83999186	0.96938279	−3.49741560

IV(acetaldehyde)

E (TPSSh/def2-TZVP) =	−1361.82391477
$G - E$ (TPSSh/def2-TZVP) =	0.39472214
$H - E$ (TPSSh/def2-TZVP) =	0.47240456
E (DLPNO-CCSD(T)/def-TZVPP//TPSSh/def2-TZVP) =	−1354.81226296
E (DLPNO-CCSD(T)/def-QZVPP//TPSSh/def2-TZVP) =	−1354.86495719
E (DLPNO-CCSD(T)tight/def-QZVPP//TPSSh/def2-TZVP) =	
E (DLPNO-CCSD(T)/CBS//TPSSh/def2-TZVP) =	−1359.66734333

C	1.33985973	2.87931226	−0.69713015
N	2.24965284	2.26491530	−1.45281154
C	2.40958483	0.89889805	−1.19375389
C	1.57376348	0.46355841	−0.20965518
S	0.61454869	1.78245540	0.36802611
C	2.97659707	2.92188034	−2.55952107
C	1.00758769	4.36602035	−0.68712665
C	2.02720643	5.11005487	0.18172079
O	1.00006758	4.90803032	−1.96981657
N	−1.34515612	4.80315828	−3.20255818
C	−2.07424230	3.53712457	−3.07706291
C	−2.15037352	5.92103168	−2.69273173
C	−0.95608761	5.04032699	−4.60094283
H	−3.00744023	3.54435352	−3.65668882
C	3.41593856	0.09822820	−1.94751407
H	0.07538377	4.80447785	−2.40861535
H	4.00205362	2.55072262	−2.52264227
C	2.34691716	2.64402406	−3.90826786
H	2.97541454	3.98952924	−2.36399720
C	1.44790115	−0.90222058	0.38347922
H	−1.44538781	2.72339160	−3.43974719
H	−2.32096475	3.35973462	−2.02822861
H	−3.07478955	6.05144835	−3.27107171
H	−2.41521983	5.73348911	−1.65066843
H	−1.56661651	6.84039901	−2.74854014
H	−1.83463492	5.11975279	−5.25518817
H	−0.38611944	5.96805810	−4.66157606
H	−0.32904500	4.21782248	−4.94511175
H	0.02146339	4.44342342	−0.20788536
H	1.73680309	6.15989369	0.22184435
H	2.05866276	4.70704656	1.19572226
H	3.02435288	5.04412534	−0.25745289
H	3.31905861	−0.95296415	−1.68347555
H	3.27684535	0.19924051	−3.02573908
H	4.43319392	0.41364621	−1.69954333
C	2.31356157	−1.08171503	1.63302164
H	0.40843527	−1.10787183	0.64898768
H	1.74004018	−1.65022249	−0.35616814
O	2.09475038	−2.41144500	2.06247736
H	3.36799940	−0.90276159	1.38353249
H	2.01620903	−0.35317647	2.39954773
H	2.61009640	−2.57923259	2.86144955
C	2.90178872	3.27415876	−5.02303673
C	2.37604755	3.05207079	−6.28912549
C	1.29188291	2.19396594	−6.45713234
C	0.73430045	1.56722562	−5.34978770
C	1.25711744	1.79498447	−4.07871776
H	3.74545336	3.94509076	−4.90056595
H	2.81450995	3.54869086	−7.14610720
H	0.88590659	2.01669992	−7.44541422
H	−0.10998107	0.89876431	−5.47024769
H	0.80394132	1.29932124	−3.22754643

5.4.4 Catalyst 4

I

E (TPSSh/def2-TZVP) =	−1175.61372237
$G - E$ (TPSSh/def2-TZVP) =	0.42853516
$H - E$ (TPSSh/def2-TZVP) =	0.50459062
E (DLPNO-CCSD(T)/def-TZVPP//TPSSh/def2-TZVP) =	−1169.47874737
E (DLPNO-CCSD(T)/def-QZVPP//TPSSh/def2-TZVP) =	−1169.52441946
E (DLPNO-CCSD(T)tight/def-QZVPP//TPSSh/def2-TZVP) =	
E (DLPNO-CCSD(T)/CBS//TPSSh/def2-TZVP) =	−1173.70701756

C	0.63298250	1.59545435	3.84305988
N	0.05390841	1.56971121	2.64800744
C	−1.30566737	1.25301558	2.67983961
C	−1.71731876	1.03754924	3.95858352
S	−0.44027767	1.23312124	5.08339363
C	0.90298744	1.83435876	1.43707525
C	1.15554934	0.54369683	0.65991463
C	2.17836718	0.79304035	−0.45615580
C	1.71344545	1.95543419	−1.33676425
C	1.44003026	3.21937826	−0.52114581
C	0.37616817	2.97654227	0.56162858
C	2.41966792	−0.48004147	−1.26349321
C	−0.01207038	4.23257881	1.37144780
C	1.15808408	4.84725170	2.14437349
C	−2.18247491	1.19801703	1.47397568
C	−0.68254572	5.27917149	0.47839592
H	1.85030171	2.15502605	1.87408430
H	−2.71300825	0.77921227	4.28171243
H	−3.13686736	0.75463163	1.75415080
H	−2.37416487	2.19989567	1.08513513
H	−1.74538903	0.59748055	0.67717070
H	1.51792390	−0.23148116	1.34193247
H	0.22636995	0.17666516	0.21375116
H	−0.52238552	2.62769314	0.04225557
H	3.12324188	1.08876640	0.01942779
H	1.10494329	4.02242408	−1.17958756
H	2.37026625	3.55929966	−0.05284523
H	2.46737204	2.16132416	−2.10071763
H	0.80070976	1.65863140	−1.86827245
H	3.17154806	−0.31088500	−2.03690225
H	2.76843783	−1.29622195	−0.62592903
H	1.49907178	−0.80556770	−1.75655140
H	−0.76188414	3.91739709	2.10937287
H	−1.08531320	6.09390155	1.08332630
H	0.02666310	5.71490228	−0.22863728
H	−1.50699663	4.84538063	−0.09430316
H	0.81073045	5.69288980	2.74117099
H	1.62483572	4.13440263	2.82986629
H	1.93028306	5.21846309	1.46678621
H	1.70874205	1.82750474	3.97302448
N	3.61868202	2.14930812	4.22361258
C	4.43185377	2.36272232	3.02445534
C	3.73370663	3.29292168	5.13339963
C	4.01947135	0.91189749	4.89882451
H	5.50046290	2.46975328	3.26208729
H	4.31678210	1.51236639	2.34873568
H	4.10191856	3.26989332	2.51418948
H	4.76528326	3.43910421	5.48524201
H	3.41077253	4.20121292	4.62186414
H	3.09427171	3.13370410	6.00433133
H	5.06856129	0.93922289	5.22709548
H	3.39125801	0.75364746	5.77832875
H	3.89296510	0.06653383	4.21907891

II(formaldehyde)

E (TPSSh/def2-TZVP) = −1115.16228817
$G - E$ (TPSSh/def2-TZVP) = 0.32844724
$H - E$ (TPSSh/def2-TZVP) = 0.39568856
E (DLPNO-CCSD(T)/def-TZVPP//TPSSh/def2-TZVP) = −1109.61023688
E (DLPNO-CCSD(T)/def-QZVPP//TPSSh/def2-TZVP) = −1109.65272863
E (DLPNO-CCSD(T)tight/def-QZVPP//TPSSh/def2-TZVP) =
E (DLPNO-CCSD(T)/CBS//TPSSh/def2-TZVP) = −1113.40799965

C	1.52148865	−0.67169433	0.20330394
S	−0.04468465	−0.03120760	0.53492811
C	0.18604080	0.02142238	2.22496171
N	1.42950149	−0.44408729	2.46137683
C	2.20626130	−0.84039830	1.35966907
C	1.86998955	−0.46119840	3.88257041
C	−1.34920338	1.00256769	4.13231749
O	−0.75162235	1.05083834	5.18946428
H	−1.37927269	1.85742350	3.43558927
C	3.60551603	−1.35871448	1.46141636
H	1.88275979	−0.88933331	−0.78960288
C	2.30758910	−1.85260080	4.33555310
C	2.87452478	0.64630541	4.26206014
H	0.94888439	−0.21930078	4.41195916
H	−1.97954689	0.14266553	3.85163543
H	4.06421728	−1.33415734	0.47293513
H	4.21321727	−0.75255637	2.13403786
H	3.62943243	−2.39006207	1.81974882
C	2.44313632	−1.89114631	5.85925321
H	1.57207792	−2.58932333	3.99716732
H	3.27263121	−2.12594553	3.89956513
C	3.00865373	0.58001530	5.79392149
H	3.86069491	0.41641440	3.83590675
C	2.42624776	2.04122553	3.74291089
C	2.87097835	−3.27445830	6.34199384
C	3.42490781	−0.80264053	6.29442734
H	1.46193077	−1.65519807	6.29030530
H	3.73879971	1.31999490	6.12686974
H	2.04603429	0.85401935	6.23980103
H	3.50849042	−0.79034838	7.38585616
H	4.42175922	−1.05042168	5.90479695
H	2.94885282	−3.30283133	7.43200052
H	2.15463885	−4.04112691	6.03408330
H	3.84907670	−3.54259882	5.92934119
C	2.83680423	3.20002676	4.65689110
C	2.96236744	2.32101663	2.33442798
H	1.33209239	2.03029915	3.70158445
H	2.51026540	4.14486120	4.21454920
H	2.38633262	3.12481845	5.64708462
H	3.92441396	3.25063884	4.77479130
H	2.61595420	3.29622544	1.98236663
H	4.05781092	2.34212025	2.34313701
H	2.64561414	1.57796119	1.60285329

$\mathbf{TS_{II\text{-}III}}$(formaldehyde)

E (TPSSh/def2-TZVP) =	−1115.16333671
$G - E$ (TPSSh/def2-TZVP) =	0.33045367
$H - E$ (TPSSh/def2-TZVP) =	0.39509526
E (DLPNO-CCSD(T)/def-TZVPP//TPSSh/def2-TZVP) =	−1109.59699821
E (DLPNO-CCSD(T)/def-QZVPP//TPSSh/def2-TZVP) =	−1109.63951803
E (DLPNO-CCSD(T)tight/def-QZVPP//TPSSh/def2-TZVP) =	
E (DLPNO-CCSD(T)/CBS//TPSSh/def2-TZVP) =	−1113.40406966

S	1.58229093	0.11878705	−0.65852374
C	0.08716432	0.43569419	0.08982587
N	0.15652854	0.05383749	1.36947704
C	1.37450511	−0.50441241	1.79357296
C	2.26858084	−0.53965084	0.77669619
C	−1.07969496	0.20487945	2.19724530
C	−1.44558745	1.29784388	−0.82432733
O	−2.34853253	1.67630243	−0.04115426
H	−0.78313041	2.04250945	−1.31022762
C	1.64468546	−0.96376826	3.18958259
H	3.27878347	−0.91385818	0.82456806
C	−1.75903883	−1.14598544	2.41053028
C	−0.87127991	0.97051260	3.50918615
H	−1.73129201	0.80103999	1.55031317
H	−1.60727102	0.41770519	−1.47908599
H	2.58475199	−1.51529088	3.20214137
H	1.73719092	−0.11830760	3.87454031
H	0.85757520	−1.61754429	3.56539057
C	−2.27015967	1.19505200	4.10350828
H	−0.33058249	0.33094327	4.21695839
C	−0.05194113	2.26626738	3.33149683
C	−3.13746820	−0.94039018	3.04741522
H	−1.86384060	−1.65210124	1.44645715
H	−1.15785142	−1.79534746	3.05871683
C	−3.83756313	−2.27548977	3.28708250
H	−3.73481520	−0.35054548	2.34147531
H	−2.18644681	1.74664436	5.04358787
H	−2.85713503	1.81235117	3.41670741
C	−2.99712455	−0.12964133	4.33884385
H	−3.98740747	0.05689634	4.76608379
H	−2.44254682	−0.72611150	5.07606284
H	−4.83329042	−2.12517050	3.71237517
H	−3.94946440	−2.83623836	2.35520017
H	−3.26455522	−2.89386974	3.98588916
C	0.40054505	2.81927549	4.68514871
H	0.85561913	1.99444889	2.77796659
C	−0.78281136	3.33778454	2.51697277
H	1.05396815	3.68456271	4.54771134
H	−0.45257759	3.14502540	5.28633989
H	0.95071343	2.06907884	5.26238785
H	−0.09751843	4.15525753	2.27719091
H	−1.19553821	2.94596399	1.58321379
H	−1.61000625	3.76322582	3.09219342

2(formaldehyde)

E (TPSSh/def2-TZVP) =	−1290.17998024
$G - E$ (TPSSh/def2-TZVP) =	0.45150856
$H - E$ (TPSSh/def2-TZVP) =	0.53721925
E (DLPNO-CCSD(T)/def-TZVPP//TPSSh/def2-TZVP) =	−1283.40482795
E (DLPNO-CCSD(T)/def-QZVPP//TPSSh/def2-TZVP) =	−1283.45545622
E (DLPNO-CCSD(T)tight/def-QZVPP//TPSSh/def2-TZVP) =	
E (DLPNO-CCSD(T)/CBS//TPSSh/def2-TZVP) =	−1288.10870537

C	4.64649326	2.97477880	−4.53717773
C	4.20430745	4.02413232	−3.51411386
C	3.55285603	3.33473287	−2.30890266
C	2.41277658	2.43592239	−2.78291173
C	2.87372640	1.33814773	−3.74784087
C	3.49577087	2.05837492	−4.95529724
N	1.61816998	1.91498474	−1.62064285
C	2.02197925	0.97623550	−0.66881938
C	1.04837112	0.78803495	0.26148975
S	−0.33143862	1.75493520	−0.05327016
C	0.40181767	2.40366089	−1.41718554
C	3.32803343	0.25619781	−0.70577381
C	1.75875273	0.33259286	−4.10669702
C	2.31013554	−0.83153226	−4.93311575
C	5.36141687	4.91435659	−3.06797067
C	0.55844658	0.97564598	−4.80628416
N	−0.87428774	4.61536921	−3.11486606
C	−1.67782517	5.17728573	−2.02613247
C	0.14284336	5.57607406	−3.54491852
C	−1.72963073	4.22651417	−4.23820464
O	1.38688363	5.03258805	−0.02494736
C	1.62538110	5.83920335	0.84190133
H	−2.20854113	6.09105617	−2.33232430
H	−0.04893592	3.16711935	−2.07772096
H	1.68762465	3.06259654	−3.30527963
H	1.08332514	0.11906477	1.10618656
H	−2.42179932	4.44376370	−1.70626784
H	−1.02663629	5.41483317	−1.18299505
H	−0.30057165	6.51393975	−3.91227305
H	0.80070277	5.80845272	−2.70552613
H	0.73591479	5.14212316	−4.35297540
H	−2.28855600	5.08162228	−4.64692961
H	−1.11702200	3.80225025	−5.03519673
H	−2.44635983	3.47226822	−3.90782053
H	0.85601598	6.54110809	1.20991213
H	2.62450880	5.91372586	1.30721726
H	3.45581361	−0.28744347	0.22919318
H	3.35328158	−0.46547286	−1.52443556
H	4.16830737	0.93925350	−0.82398503
H	3.16504727	4.07163827	−1.60206061
H	4.31324505	2.74409792	−1.78866188
H	3.68252432	0.77628251	−3.26855712
H	3.44112679	4.65699746	−3.98683329
H	3.85240021	1.31700833	−5.67234852
H	2.72654237	2.64893653	−5.46507552
H	5.06515798	3.47041476	−5.41690550
H	5.45354207	2.37167060	−4.10269651
H	5.80643039	5.43038959	−3.92126244
H	5.02748631	5.67013157	−2.35245228
H	6.14541508	4.31933975	−2.59030304
H	1.39722721	−0.09243350	−3.16084109
H	1.55043796	−1.60643162	−5.05362025
H	2.60860027	−0.50643323	−5.93218851
H	3.18089318	−1.28523267	−4.45150888
H	−0.22046567	0.22992231	−4.97744255
H	0.11409012	1.78151888	−4.21631159
H	0.83932091	1.38612545	−5.77891832

$\mathbf{TS_{2\text{-}IV}}$(formaldehyde)

E (TPSSh/def2-TZVP) =	−1290.15204700
$G - E$ (TPSSh/def2-TZVP) =	0.45780472
$H - E$ (TPSSh/def2-TZVP) =	0.53711781
E (DLPNO-CCSD(T)/def-TZVPP//TPSSh/def2-TZVP) =	−1283.36477160
E (DLPNO-CCSD(T)/def-QZVPP//TPSSh/def2-TZVP) =	−1283.41546703
E (DLPNO-CCSD(T)tight/def-QZVPP//TPSSh/def2-TZVP) =	
E (DLPNO-CCSD(T)/CBS//TPSSh/def2-TZVP) =	−1288.0801762

N	1.93602352	2.32424336	−1.46021208
C	1.96065855	1.14704045	−0.69287589
C	0.98461314	1.16241068	0.24866278
S	0.09036575	2.62620031	0.14917000
C	0.99046694	3.23602436	−1.16326249
C	2.94969181	2.69639378	−2.49545783
C	1.23744996	5.61391980	−1.69999347
O	0.69313162	5.85535626	−2.77257724
N	−1.62911604	4.39654419	−2.95285233
C	−1.79541587	5.03933439	−4.29415652
C	−2.13715950	2.99366397	−2.94202081
C	−2.26559363	5.22286075	−1.88189152
H	−3.33359764	5.28958073	−2.08242240
C	2.90529736	0.00878485	−0.91044098
H	−0.61427478	4.40242605	−2.74401842
C	4.27914425	3.05614111	−1.83058295
C	3.11168109	1.68257328	−3.63458912
H	2.53014749	3.60760621	−2.92363910
H	0.77701843	0.38967308	0.97177632
H	−2.09102714	4.73928682	−0.92293738
H	−1.80298178	6.20641549	−1.90362937
H	−2.86018467	5.11527144	−4.50878156
H	−1.32788846	6.01896260	−4.25331073
H	−1.30002071	4.41894654	−5.03718609
H	−3.20410489	3.01106216	−3.15869928
H	−1.60528655	2.42737387	−3.70204653
H	−1.95269356	2.56592319	−1.95971754
H	0.73209134	5.78169432	−0.73611486
H	2.31769253	5.42081441	−1.62981157
H	2.81205808	−0.68759487	−0.07802373
H	2.66812867	−0.53136122	−1.82944383
H	3.94341235	0.33467668	−0.96640293
C	5.26651384	3.62837498	−2.85275629
H	4.09744394	3.77839247	−1.02860182
H	4.72500341	2.17115564	−1.36593875
C	4.07995709	2.30382048	−4.65397385
H	3.60362806	0.78778437	−3.24214558
C	1.76294217	1.23921585	−4.23938403
C	6.60328130	3.95909966	−2.19365961
C	5.42614953	2.64917965	−4.01793644
H	4.83454367	4.55654182	−3.25145020
H	4.22611738	1.61104981	−5.48533538
H	3.63833005	3.21524065	−5.07167538
H	6.09540667	3.07526106	−4.77006385
H	5.90602634	1.73254580	−3.65246493
H	7.29627333	4.39554185	−2.91615414
H	6.47727490	4.67086036	−1.37389156
H	7.06755392	3.05556647	−1.78730640
C	1.02269388	2.37937909	−4.94423782
C	1.93959295	0.04610829	−5.18107450
H	1.13491060	0.90305155	−3.40441606
H	0.96963823	−0.33007726	−5.51469658
H	2.50958366	0.32034450	−6.07164760
H	2.46498696	−0.77436933	−4.68515461
H	0.04183949	2.03531418	−5.28525492
H	0.88578613	3.24423159	−4.28746773
H	1.56599884	2.72107546	−5.82755453

IV(formaldehyde)

E (TPSSh/def2-TZVP) =	−1290.20616892
$G - E$ (TPSSh/def2-TZVP) =	0.46080700
$H - E$ (TPSSh/def2-TZVP) =	0.53826327
E (DLPNO-CCSD(T)/def-TZVPP//TPSSh/def2-TZVP) =	−1283.41673168
E (DLPNO-CCSD(T)/def-QZVPP//TPSSh/def2-TZVP) =	−1283.46721394
E (DLPNO-CCSD(T)tight/def-QZVPP//TPSSh/def2-TZVP) =	
E (DLPNO-CCSD(T)/CBS//TPSSh/def2-TZVP) =	−1288.13643166

N	2.09112761	2.33994879	−1.39765679
C	2.00620395	1.08380112	−0.79019103
C	1.07460303	1.07858873	0.20013550
S	0.33339556	2.60703018	0.37932416
C	1.27191134	3.25360519	−0.86798171
C	3.06575581	2.75378267	−2.47315663
C	1.16559882	4.70692966	−1.28009963
O	0.76521385	4.85883623	−2.60679699
N	−1.86696145	4.59835489	−2.95263666
C	−2.03032859	5.04203798	−4.34583460
C	−2.30522952	3.20672181	−2.80159745
C	−2.61088516	5.48031832	−2.04346073
H	−3.68630393	5.47924606	−2.26580736
C	2.83424288	−0.09887748	−1.16636805
H	−0.25115353	4.73437113	−2.68591733
C	4.41782418	3.05896946	−1.82574157
C	3.18914107	1.76843018	−3.64334780
H	2.62935539	3.67231676	−2.86477844
H	0.80505969	0.24418686	0.82720975
H	−2.47056803	5.14520358	−1.01388429
H	−2.23410010	6.49930953	−2.13960042
H	−3.08270733	5.01004636	−4.65728131
H	−1.66295935	6.06400266	−4.44336569
H	−1.44757536	4.39456390	−5.00170570
H	−3.36534743	3.08185237	−3.05999155
H	−1.70847348	2.56668233	−3.45302266
H	−2.16344600	2.89025376	−1.76624385
H	0.48717357	5.19565893	−0.56988725
H	2.14925043	5.17115435	−1.15515251
H	2.67677541	−0.87912862	−0.42285497
H	2.54596593	−0.49258704	−2.14179551
H	3.89690139	0.14231872	−1.19004823
C	5.38196905	3.66693503	−2.85161584
H	4.28103944	3.74594637	−0.98462228
H	4.85364741	2.13871118	−1.42100907
C	4.11728016	2.44146045	−4.66869281
H	3.71916083	0.87899567	−3.29236506
C	1.83692551	1.31182596	−4.23395400
C	6.74235033	3.94540422	−2.21711582
C	5.48952902	2.74823917	−4.07074892
H	4.94988994	4.61964672	−3.18560788
H	4.22241685	1.78743411	−5.53683658
H	3.65882746	3.37029600	−5.02316591
H	6.13039179	3.21234850	−4.82466592
H	5.97950640	1.81259349	−3.77336039
H	7.41813162	4.40770478	−2.93943973
H	6.65247746	4.61852630	−1.36082920
H	7.20567965	3.01683727	−1.87091007
C	1.12793188	2.40377162	−5.03911741
C	2.01796230	0.04864297	−5.07977420
H	1.18313407	1.04670834	−3.39190121
H	1.05254220	−0.31817334	−5.43442984
H	2.63678731	0.24654396	−5.95810580
H	2.49465638	−0.75508742	−4.51079088
H	0.12914017	2.06450497	−5.32474188
H	1.02761531	3.33054712	−4.47135165
H	1.67035483	2.62321320	−5.96130429

II(acetaldehyde)

E (TPSSh/def2-TZVP) =	−1154.50870336
$G - E$ (TPSSh/def2-TZVP) =	0.35386921
$H - E$ (TPSSh/def2-TZVP) =	0.42505141
E (DLPNO-CCSD(T)/def-TZVPP//TPSSh/def2-TZVP) =	−1148.67638664
E (DLPNO-CCSD(T)/def-QZVPP//TPSSh/def2-TZVP) =	−1148.72059779
E (DLPNO-CCSD(T)tight/def-QZVPP//TPSSh/def2-TZVP) =	
E (DLPNO-CCSD(T)/CBS//TPSSh/def2-TZVP) =	−1152.68107244

S	1.95312440	−0.54446384	−0.42885714
C	0.36934911	−0.22817726	0.13370357
N	0.41485461	−0.36481328	1.47893345
C	1.64172600	−0.70370963	2.07423312
C	2.61036502	−0.84176620	1.13780008
C	−0.85125406	−0.09489975	2.21051186
C	−2.31170584	0.93326500	−1.06134022
C	−1.58774128	2.06102520	−1.73580485
O	−3.06231644	1.07186242	−0.11829798
C	1.84519443	−0.87070239	3.54710228
H	3.64330337	−1.09284875	1.32239513
C	−1.28054260	−1.26058406	3.09840029
C	−0.89247390	1.26569728	2.93611522
H	−1.57143334	−0.02254960	1.39606273
H	−2.13537652	−0.06792579	−1.49488195
H	−1.88438312	2.10176353	−2.78972068
H	−1.80718735	3.01121343	−1.25003071
H	−0.51527636	1.85267176	−1.70614571
H	2.91447174	−0.86597124	3.75883066
H	1.38264979	−0.06212982	4.11448135
H	1.43348102	−1.81565224	3.90819299
C	−2.34198813	1.44585158	3.41906900
H	−0.25338874	1.22604903	3.82874536
C	−0.38709480	2.41715583	2.02089188
C	−2.72121539	−1.05562108	3.57320032
H	−1.19119742	−2.19506496	2.53527347
H	−0.63955765	−1.34466884	3.98038189
H	−2.42685924	2.38224757	3.97445459
H	−2.99543972	1.52860664	2.54448346
C	−2.81726654	0.28848884	4.29672061
C	−3.18275297	−2.21347345	4.45414076
H	−3.36492514	−1.00882832	2.68573576
H	−3.84908799	0.46666004	4.61616992
H	−2.20788467	0.24221227	5.20972646
H	−4.21800187	−2.07321857	4.77615939
H	−3.12038967	−3.16649677	3.92153742
H	−2.56019261	−2.28847542	5.35175591
C	−1.14372297	3.73655185	2.19541541
C	1.11039905	2.67144250	2.22124598
H	−0.53552957	2.08967115	0.98709606
H	−0.72349275	4.48825017	1.52140188
H	−2.20424496	3.63532359	1.96164911
H	−1.04982067	4.12251827	3.21558924
H	1.46136995	3.45372527	1.54284728
H	1.30527153	3.00742766	3.24560838
H	1.71353527	1.78176540	2.03715596

$\mathbf{TS_{II\text{-}III}}$(acetaldehyde)

E (TPSSh/def2-TZVP) =	−1154.50426943
$G-E$ (TPSSh/def2-TZVP) =	0.35729526
$H-E$ (TPSSh/def2-TZVP) =	0.42435322
E (DLPNO-CCSD(T)/def-TZVPP//TPSSh/def2-TZVP) =	−1148.65041768
E (DLPNO-CCSD(T)/def-QZVPP//TPSSh/def2-TZVP) =	−1148.69464992
E (DLPNO-CCSD(T)tight/def-QZVPP//TPSSh/def2-TZVP) =	
E (DLPNO-CCSD(T)/CBS//TPSSh/def2-TZVP) =	−1152.67096047

C	1.18757323	0.07256131	−0.60372343
N	0.03595570	−0.04368030	0.06843649
C	0.13674204	−0.40862353	1.42116624
C	1.43167045	−0.56972484	1.78453877
S	2.47728996	−0.26083223	0.45322597
C	−1.23570864	0.16506015	−0.69042445
C	1.37608127	0.51325529	−2.48572918
O	0.38374164	1.15912155	−2.92265711
C	2.73885082	1.20256570	−2.45081134
C	−1.03394856	−0.56984619	2.33566951
H	1.79267943	−0.84294114	2.76327663
C	−1.90616798	−1.17137333	−1.00060315
C	−2.20042544	1.16796561	−0.04675610
H	−0.88164235	0.58785124	−1.63813705
H	1.47625470	−0.56037179	−2.76347039
H	3.04396950	1.38051202	−3.48582292
H	2.66563735	2.16715731	−1.94593362
H	3.50865072	0.58984396	−1.96936988
H	−0.69392672	−1.03551527	3.26066582
H	−1.47986744	0.39525573	2.58449322
H	−1.81066119	−1.19815622	1.89974722
C	−3.32555272	1.39502469	−1.06822172
H	−2.66766657	0.70722582	0.83168210
C	−1.50837205	2.46587411	0.42087916
C	−3.06553351	−0.95674425	−1.97946571
H	−1.16967329	−1.85248682	−1.43705767
H	−2.29011637	−1.64519539	−0.08856893
H	−4.04537640	2.11453371	−0.66921051
H	−2.90227932	1.83034863	−1.97840118
C	−3.76827553	−2.27399531	−2.29744663
C	−4.03533926	0.08590052	−1.41627236
H	−2.63674293	−0.55298400	−2.90482634
H	−4.83509966	0.27332022	−2.13967319
H	−4.51383201	−0.32201961	−0.51543385
H	−4.57487978	−2.12377291	−3.01980039
H	−3.07041426	−3.00354226	−2.71721548
H	−4.20566931	−2.70820605	−1.39235404
C	−2.43122226	3.27996410	1.33114330
H	−0.64538861	2.16467858	1.02814543
C	−0.97935817	3.32174190	−0.73349891
H	−0.36699672	4.13798282	−0.34034093
H	−0.37613077	2.74492731	−1.44024253
H	−1.80562816	3.77305887	−1.29021671
H	−1.90583517	4.15104321	1.73081065
H	−3.30491954	3.64621569	0.78530616
H	−2.78989313	2.68435335	2.17702700

III(acetaldehyde)

E (TPSSh/def2-TZVP) =	−1154.51165367
$G - E$ (TPSSh/def2-TZVP) =	0.35933695
$H - E$ (TPSSh/def2-TZVP) =	0.42612379
E (DLPNO-CCSD(T)/def-TZVPP//TPSSh/def2-TZVP) =	−1148.65174109
E (DLPNO-CCSD(T)/def-QZVPP//TPSSh/def2-TZVP) =	−1148.69634165
E (DLPNO-CCSD(T)tight/def-QZVPP//TPSSh/def2-TZVP) =	
E (DLPNO-CCSD(T)/CBS//TPSSh/def2-TZVP) =	−1152.67883575

N	1.23064317	0.11688910	−0.49617056
C	0.02299023	0.21644505	0.20004831
C	0.19704976	0.06758510	1.53268505
S	1.84751021	−0.23388393	1.93269707
C	2.30991261	−0.10928737	0.29027714
C	3.74227478	−0.06309693	−0.15650723
C	4.70781125	−0.72191690	0.83375944
O	3.82063399	1.28782183	−0.23632694
C	1.44261898	0.26938206	−1.96270365
C	−1.30502869	0.42041008	−0.45243964
H	−0.56965110	0.11063591	2.28829159
H	2.49991202	0.52806747	−2.02991926
C	1.18524589	−1.06600882	−2.66223172
C	0.67127662	1.42192990	−2.62313573
H	3.84463122	−0.61627839	−1.12039648
H	5.72387207	−0.52553142	0.48694511
H	4.60592896	−0.27013734	1.82475163
H	4.56084264	−1.80520930	0.90538402
H	−1.46212188	−0.27945591	−1.27514039
H	−2.08279719	0.25109236	0.29205359
H	−1.41893939	1.43373045	−0.84021782
C	1.58123704	−1.00848897	−4.13962308
H	1.74748825	−1.85313667	−2.15092802
H	0.12192374	−1.32597634	−2.58359082
C	1.14479965	1.48017449	−4.08425294
H	−0.39043928	1.15723161	−2.65989533
C	0.79766272	2.77309264	−1.88385577
H	0.64227337	2.30570278	−4.59455630
H	2.21741303	1.69694362	−4.10937058
C	1.29009547	−2.33361757	−4.83966319
C	0.87736094	0.16782297	−4.81952849
H	2.66240714	−0.82137248	−4.18355296
H	1.20596189	0.24408347	−5.86077504
H	−0.20301963	−0.02749548	−4.84241898
H	1.60158089	−2.30077103	−5.88689257
H	1.81544915	−3.16158099	−4.35605167
H	0.21834826	−2.55527233	−4.81549974
C	−0.29538596	3.73935425	−2.34961147
H	0.63022590	2.57665150	−0.81844655
C	2.18350154	3.41110220	−2.00516496
H	−0.26380458	4.66240504	−1.76584371
H	−0.16334447	4.01195710	−3.40054117
H	−1.29567124	3.30613319	−2.24171327
H	2.22024132	4.32029414	−1.39902250
H	2.96297107	2.73842328	−1.63767533
H	2.39397163	3.69910259	−3.03951111

2(acetaldehyde)

E (TPSSh/def2-TZVP) =	−1329.52738254
$G - E$ (TPSSh/def2-TZVP) =	0.47834323
$H - E$ (TPSSh/def2-TZVP) =	0.56685051
E (DLPNO-CCSD(T)/def-TZVPP//TPSSh/def2-TZVP) =	−1322.46274587
E (DLPNO-CCSD(T)/def-QZVPP//TPSSh/def2-TZVP) =	−1322.51516135
E (DLPNO-CCSD(T)tight/def-QZVPP//TPSSh/def2-TZVP) =	
E (DLPNO-CCSD(T)/CBS//TPSSh/def2-TZVP) =	−1327.37937383

C	4.13488932	4.20174380	−4.91219785
C	4.22832282	4.52054954	−3.41971123
C	3.79771297	3.28391555	−2.62658026
C	2.38678298	2.87679921	−3.03988114
C	2.26076369	2.49434376	−4.52719193
C	2.73738049	3.73112386	−5.31236898
N	1.81374830	1.85512486	−2.10320552
C	0.62087488	2.08447889	−1.56957558
S	0.11460468	0.83435447	−0.56832564
C	1.56025745	−0.03382149	−0.87432118
C	2.38220160	0.63665094	−1.72443121
C	3.70048291	0.13837549	−2.21497139
C	0.82124389	2.04024799	−4.89850151
C	0.62811575	0.53084037	−4.70671051
C	5.62819741	4.97090199	−3.01141544
C	0.41005286	2.39847833	−6.33057582
N	−1.27443944	4.37441557	−2.34159266
C	−2.38945236	3.84067810	−3.12549230
C	−1.73139998	4.80135421	−1.01763421
C	−0.63046485	5.48424021	−3.04386860
O	1.81041062	4.76343816	−0.40906748
C	2.15301216	5.71686936	0.25934034
C	3.47193496	5.84484606	0.94959866
H	−2.46891275	5.61594401	−1.07620271
H	0.01447463	2.97557474	−1.80965349
H	1.73539878	3.73640215	−2.87355941
H	1.74115262	−0.99777728	−0.42690800
H	−2.19684777	3.95795809	−0.50226279
H	−0.87405924	5.14013393	−0.43487034
H	−1.31483782	6.33264793	−3.19720979
H	0.22734125	5.82796372	−2.46338793
H	−0.28491968	5.14422784	−4.02244306
H	−3.18268263	4.58729535	−3.28110827
H	−2.02783874	3.51504457	−4.10255637
H	−2.82275594	2.98219764	−2.60803881
H	1.46289361	6.57545531	0.38605080
H	3.98982738	6.73286026	0.57076804
H	3.30647449	6.02003090	2.01773816
H	4.08737272	4.95857203	0.80407691
H	4.52820474	0.64803858	−1.71945123
H	3.77586142	−0.92579840	−1.99563589
H	3.80400313	0.27515628	−3.29122790
H	3.81167225	3.48724477	−1.55383532
H	4.51044573	2.48352374	−2.83584316
H	2.95497958	1.67442401	−4.74902786
H	3.51971849	5.32920110	−3.19568698
H	2.71965255	3.50316936	−6.37856565
H	2.02270651	4.54766865	−5.15298710
H	4.40636283	5.08148401	−5.50177252
H	4.86879492	3.42288979	−5.15465600
H	5.92764831	5.86293493	−3.56571012
H	5.67498229	5.20390933	−1.94427510
H	6.36318776	4.18716211	−3.21718772
H	0.13214254	2.56563844	−4.22415127
H	−0.40261007	0.24738263	−4.93013460
H	1.27795042	−0.02050769	−5.39255103
H	0.84899260	0.18764990	−3.69513234
H	−0.60124834	2.03242901	−6.52149685
H	0.41411955	3.47391204	−6.51157204
H	1.07242872	1.92693902	−7.06178581

$\mathbf{TS_{2\text{-}IV}}$(acetaldehyde)

E (TPSSh/def2-TZVP) =	−1329.49693383
$G-E$ (TPSSh/def2-TZVP) =	0.48391091
$H-E$ (TPSSh/def2-TZVP) =	0.56515436
E (DLPNO-CCSD(T)/def-TZVPP//TPSSh/def2-TZVP) =	−1322.43011781
E (DLPNO-CCSD(T)/def-QZVPP//TPSSh/def2-TZVP) =	−1322.48249328
E (DLPNO-CCSD(T)tight/def-QZVPP//TPSSh/def2-TZVP) =	
E (DLPNO-CCSD(T)/CBS//TPSSh/def2-TZVP) =	−1327.35167722

C	1.11271814	2.54518414	−1.22735706
N	2.15660034	2.03341774	−1.91763894
C	2.48245408	0.68023730	−1.71414565
C	1.64510650	0.11357626	−0.81191285
S	0.50669252	1.27786975	−0.25772836
C	2.86305039	2.94945033	−2.85684447
C	1.21680894	5.31169723	−0.34828265
C	2.67136299	5.51800731	−0.10225613
O	0.60814032	5.79267045	−1.29322784
N	−1.70644440	4.62015540	−2.14251809
C	−2.55967363	5.35415040	−1.16032394
C	−1.71446655	5.30066649	−3.47309723
C	−2.10468508	3.18710270	−2.25276511
H	−3.58690626	5.35972277	−1.52148086
C	3.59403932	−0.04162029	−2.40800013
H	−0.72730133	4.70281811	−1.78388393
C	4.35829358	3.03903614	−2.55537684
C	2.56091767	2.70377523	−4.34966815
H	2.41137621	3.91410353	−2.61508764
H	1.66586717	−0.91020936	−0.47311282
H	−2.50134897	4.84339643	−0.20127198
H	−2.17854156	6.36832989	−1.06781833
H	−2.73629532	5.32248895	−3.84893098
H	−1.33057872	6.30956600	−3.34159412
H	−1.07313666	4.74236506	−4.15058991
H	−3.11466793	3.14067548	−2.65897145
H	−1.40057290	2.67889224	−2.90520416
H	−2.07082176	2.73801645	−1.26379402
H	0.68141275	4.73536725	0.42339353
H	2.78503570	6.14125837	0.79256269
H	3.14140955	4.55806260	0.12472923
H	3.15578531	6.00156215	−0.94847657
H	4.56521590	0.20372511	−1.97377042
H	3.43949275	−1.11434054	−2.29712972
H	3.62934029	0.18707724	−3.47302313
C	5.00138503	4.18591068	−3.33809753
H	4.50498017	3.17499450	−1.47954388
H	4.86580358	2.11356189	−2.83548609
C	3.23159040	3.85504592	−5.11998522
H	3.03815549	1.77151408	−4.67410453
C	1.03463367	2.59127408	−4.61588886
C	6.49436382	4.28889761	−3.03794557
C	4.72579198	3.97858096	−4.82732490
H	4.51696881	5.12335526	−3.03207844
H	3.08180551	3.71017799	−6.19094079
H	2.73429797	4.79692500	−4.85500726
H	5.14659383	4.80592390	−5.40545346
H	5.24117758	3.06874282	−5.16005086
H	6.94681536	5.11868987	−3.58548942
H	6.67568602	4.44838621	−1.97182871
H	7.01169383	3.37127565	−3.33345459
C	0.59879300	3.12510502	−5.98442689
C	0.52970844	1.15089420	−4.47178544
H	0.54885286	3.20333974	−3.84245037
H	−0.54557497	1.09673149	−4.66602106
H	1.02034437	0.50639340	−5.20689351
H	0.71126593	0.73088325	−3.48286611
H	−0.48083255	2.99654765	−6.10577020
H	0.83019863	4.18307559	−6.11774299
H	1.07916591	2.57156793	−6.79565133

IV(acetaldehyde)

E (TPSSh/def2-TZVP) =	−1329.54164010
$G - E$ (TPSSh/def2-TZVP) =	0.48807603
$H - E$ (TPSSh/def2-TZVP) =	0.56652789
E (DLPNO-CCSD(T)/def-TZVPP//TPSSh/def2-TZVP) =	−1322.46183474
E (DLPNO-CCSD(T)/def-QZVPP//TPSSh/def2-TZVP) =	−1322.51401119
E (DLPNO-CCSD(T)tight/def-QZVPP//TPSSh/def2-TZVP) =	
E (DLPNO-CCSD(T)/CBS//TPSSh/def2-TZVP) =	−1327.39765939

C	1.54206629	2.71777741	−0.92789967
N	2.32384689	2.22139176	−1.89067668
C	2.49374521	0.83222876	−1.82787363
C	1.80531238	0.29935133	−0.78584313
S	0.95448543	1.49682523	0.08695032
C	3.01363196	3.13647416	−2.86895320
C	1.17463879	4.17361928	−0.65609756
C	2.35023068	4.95227459	−0.06448529
O	0.70207504	4.81339976	−1.80573635
N	−1.94409617	4.53794247	−2.07271800
C	−2.54900047	5.56972227	−1.21832741
C	−2.20970052	4.82653548	−3.48903421
C	−2.44836950	3.20706351	−1.71685961
H	−3.63886780	5.61345957	−1.34551825
C	3.28101387	0.02786490	−2.80793814
H	−0.30679825	4.65893757	−1.89827433
C	4.50284407	3.18305834	−2.51353746
C	2.71155886	2.86270910	−4.35660704
H	2.57002730	4.10734258	−2.65597928
H	1.75342743	−0.74211144	−0.51244151
H	−2.32856687	5.35111436	−0.17215720
H	−2.12464323	6.54161811	−1.47196033
H	−3.28675180	4.84436608	−3.70258246
H	−1.78296820	5.79657016	−3.74657167
H	−1.74256781	4.06015100	−4.10839879
H	−3.53171572	3.12379876	−1.87815615
H	−1.94669632	2.45343506	−2.32604936
H	−2.24167451	3.00837266	−0.66350270
H	0.38970696	4.11832983	0.11136548
H	1.98959539	5.93941898	0.22511044
H	2.75770015	4.44982500	0.81501301
H	3.14359854	5.08174848	−0.80049567
H	4.27622406	0.43354547	−2.97753971
H	3.38533198	−0.98375729	−2.41775849
H	2.76504348	−0.02898529	−3.76691973
C	5.22151627	4.26229457	−3.32387902
H	4.60846563	3.37224362	−1.44112432
H	4.98413339	2.22276818	−2.71597478
C	3.48999089	3.94107564	−5.13914130
H	3.11778912	1.89028533	−4.65305367
C	1.19224169	2.90608772	−4.68353364
C	6.70629698	4.30937968	−2.97358242
C	4.97895831	3.99413831	−4.80715349
H	4.77025923	5.23215895	−3.07440052
H	3.36672443	3.75187679	−6.20526718
H	3.03587121	4.91873494	−4.93624653
H	5.45943922	4.76802326	−5.41141977
H	5.45379211	3.04299498	−5.07997301
H	7.21272148	5.09356508	−3.53998956
H	6.85863038	4.50923852	−1.90993284
H	7.19068897	3.35775857	−3.21184970
C	0.91521864	3.19711942	−6.16397885
C	0.44924948	1.61375413	−4.31901886
H	0.77066767	3.72716275	−4.09423752
H	−0.60058555	1.69437072	−4.61041644
H	0.86339747	0.76214737	−4.86737676
H	0.46783560	1.37585847	−3.25546478
H	−0.15995741	3.14808838	−6.35156980
H	1.25677649	4.18612426	−6.46801077
H	1.39375971	2.45492236	−6.81029771

5.4.5 Catalyst 5

I

E (TPSSh/def2-TZVP) =	−958.52837138
$G - E$ (TPSSh/def2-TZVP) =	0.37549765
$H - E$ (TPSSh/def2-TZVP) =	0.44946543
E (DLPNO-CCSD(T)/def-TZVPP//TPSSh/def2-TZVP) =	−952.23980240
E (DLPNO-CCSD(T)/def-QZVPP//TPSSh/def2-TZVP) =	−952.28104638
E (DLPNO-CCSD(T)tight/def-QZVPP//TPSSh/def2-TZVP) =	
E (DLPNO-CCSD(T)/CBS//TPSSh/def2-TZVP) =	−956.818861119

C	3.17107344	0.90747646	−0.50023268
C	3.36918008	2.08593764	−1.20981681
C	4.48899693	2.29039871	−2.00565861
C	5.45247595	1.29207495	−2.06943097
C	5.28057686	0.10780125	−1.35869064
C	4.14030134	−0.08384103	−0.58301711
N	2.38188432	3.11775893	−1.11521028
C	2.57231371	4.43593468	−1.15437049
N	1.33981250	4.97285494	−1.04843991
C	0.46782404	3.92884587	−0.94254284
N	1.05078427	2.76505918	−0.97372425
C	0.63355168	6.25482748	−0.83115968
C	−0.81233308	5.74179552	−1.08106583
O	−0.79271234	4.29133728	−0.82979970
C	0.87711256	6.85796873	0.57359010
C	0.44009152	5.89217941	1.68157788
C	2.36341704	7.18753698	0.73770043
C	0.06754417	8.16156287	0.65106256
H	0.92851277	6.96845085	−1.60090956
H	−1.54251481	6.16673097	−0.40050145
H	−1.12697553	5.88251383	−2.11485604
H	2.27772926	0.77603575	0.09507712
H	4.00273271	−1.00849540	−0.03687020
H	6.03018686	−0.67129014	−1.41858548
H	6.32763045	1.43323099	−2.69141218
H	4.59289154	3.19855676	−2.58318212
H	0.25373883	8.64746197	1.61014468
H	0.35850059	8.85816111	−0.13994127
H	−1.00850272	7.98831559	0.57851119
H	0.61664147	6.35433891	2.65416331
H	−0.62238603	5.64640306	1.62372236
H	1.01138634	4.96007436	1.65631913
H	2.52836380	7.67035755	1.70241171
H	2.98433912	6.28878547	0.71579275
H	2.70658048	7.87300922	−0.04093404
H	3.52686430	4.96422488	−1.31332609
N	5.12791471	6.09883057	−1.94301960
C	6.06395271	5.40607508	−2.83487070
C	4.50406939	7.21325273	−2.66108341
C	5.84832158	6.58787078	−0.76202556
H	6.85766164	6.07798508	−3.19364576
H	6.53169845	4.57684257	−2.30214346
H	5.52849843	5.01896303	−3.70479391
H	6.66612868	7.27152632	−1.03404018
H	5.16115012	7.12057602	−0.10388389
H	6.27147016	5.74135770	−0.21844661
H	5.24532469	7.93920183	−3.02668999
H	3.94826111	6.83033280	−3.52012618
H	3.81454793	7.73944790	−1.99832578

II(formaldehyde)

E (TPSSh/def2-TZVP) =	−898.08959302
$G-E$ (TPSSh/def2-TZVP) =	0.27388358
$H-E$ (TPSSh/def2-TZVP) =	0.34014289
E (DLPNO-CCSD(T)/def-TZVPP//TPSSh/def2-TZVP) =	−892.39203386
E (DLPNO-CCSD(T)/def-QZVPP//TPSSh/def2-TZVP) =	−892.43011978
E (DLPNO-CCSD(T)tight/def-QZVPP//TPSSh/def2-TZVP) =	
E (DLPNO-CCSD(T)/CBS//TPSSh/def2-TZVP) =	−896.534061447

C	0.06260730	−0.19087534	0.12093179
N	0.01476042	0.24478695	1.41826670
C	1.27526487	0.38095376	1.91773577
N	2.20164469	0.09189416	1.06492889
N	1.40618951	−0.26810117	−0.04322492
C	−0.98732882	0.49968412	2.45605964
C	2.06730750	−0.67208076	−1.22960275
C	−1.06417531	1.03335038	−2.17962317
O	−1.71433307	2.02002152	−1.92645148
C	−2.14477769	1.43599210	2.05346814
H	−1.41393465	−0.45925927	2.76651103
O	1.32202081	0.75420799	3.19491557
H	0.02741631	1.07279006	−2.34473957
H	−1.52755120	0.03837081	−2.30554285
C	1.36591173	−1.40475564	−2.18593858
C	2.00740523	−1.78357785	−3.35753015
C	3.34224545	−1.45037097	−3.57226169
C	4.03472325	−0.73271048	−2.60240627
C	3.40414765	−0.33767602	−1.42835745
H	0.33562464	−1.67281992	−1.99146681
H	1.46271689	−2.35421911	−4.10068223
H	3.83994344	−1.75437978	−4.48532677
H	5.07516466	−0.47253553	−2.75838145
H	3.92985655	0.21984277	−0.66579852
C	−0.06018628	1.05659804	3.57336292
H	−0.23423375	0.59389601	4.54162817
H	−0.13714877	2.14036600	3.65933827
C	−2.94817470	1.75406586	3.32319796
C	−1.62606952	2.72908822	1.41714663
C	−3.04723424	0.68951586	1.06267320
H	−2.47060929	3.37486601	1.16816325
H	−0.97324613	3.28727888	2.09469162
H	−1.08506940	2.52899172	0.49259004
H	−3.83226529	2.33945729	3.06165698
H	−3.28755718	0.83836309	3.81729084
H	−2.36769845	2.33920313	4.04260692
H	−3.87064488	1.33435067	0.74812291
H	−2.48923263	0.38873039	0.17645176
H	−3.47044464	−0.20601097	1.52846664

TS$_{\text{II-III}}$(formaldehyde)

E (TPSSh/def2-TZVP) =	−898.09105755
$G - E$ (TPSSh/def2-TZVP) =	0.27762547
$H - E$ (TPSSh/def2-TZVP) =	0.33988256
E (DLPNO-CCSD(T)/def-TZVPP//TPSSh/def2-TZVP) =	−892.37040134
E (DLPNO-CCSD(T)/def-QZVPP//TPSSh/def2-TZVP) =	−892.40853042
E (DLPNO-CCSD(T)tight/def-QZVPP//TPSSh/def2-TZVP) =	
E (DLPNO-CCSD(T)/CBS//TPSSh/def2-TZVP) =	−896.52953282

N	0.08050696	−0.13403726	0.26264848
C	0.07189727	−0.16809669	1.62031037
N	1.24390050	−0.00531389	2.15032868
N	2.02733026	0.13553012	0.99058966
C	1.34836513	0.04701328	−0.17422996
C	3.42566343	0.30934300	1.11677157
C	−1.25921812	−0.31536301	−0.29390240
C	1.71858081	−0.78527478	−1.88030154
O	0.70919749	−1.45760958	−2.19567422
H	−1.20624121	−1.10592249	−1.04354313
C	−1.83329419	0.96817867	−0.93852434
O	−1.13147286	−0.38373218	2.13964652
H	2.62265257	−1.29243691	−1.47967644
H	1.98973603	0.12722984	−2.44774841
C	4.16705486	0.65950238	−0.01071927
C	5.54280596	0.80733451	0.10371089
C	6.17386988	0.62087574	1.33046349
C	5.41815609	0.28273872	2.44873940
C	4.04151071	0.12152770	2.35092222
H	3.66474472	0.82805210	−0.95490909
H	6.12152096	1.07899806	−0.77106049
H	7.24695394	0.74340265	1.41458999
H	5.90132315	0.13841693	3.40787975
H	3.44081533	−0.14520878	3.20908411
C	−1.97872486	−0.78375746	1.00126456
H	−2.07194624	−1.86916026	1.04276571
H	−2.94703867	−0.31678053	1.15656228
C	−3.22903532	0.62607822	−1.47671465
C	−1.92049990	2.11385777	0.07623834
C	−0.93330869	1.38298480	−2.10851079
H	−3.65829689	1.49854563	−1.97435340
H	−3.17634568	−0.18729442	−2.20515475
H	−3.91847656	0.33059522	−0.68031605
H	−2.31630481	3.00766580	−0.41144054
H	−2.57971477	1.87685681	0.91581067
H	−0.93459576	2.36484111	0.47586676
H	−1.38799669	2.22133103	−2.64255888
H	0.04841971	1.70427214	−1.75250234
H	−0.78221456	0.55137600	−2.79931378

III(formaldehyde)

E (TPSSh/def2-TZVP) =	−898.09549259
$G - E$ (TPSSh/def2-TZVP) =	0.27919127
$H - E$ (TPSSh/def2-TZVP) =	0.34097137
E (DLPNO-CCSD(T)/def-TZVPP//TPSSh/def2-TZVP) =	−892.37560933
E (DLPNO-CCSD(T)/def-QZVPP//TPSSh/def2-TZVP) =	−892.41399249
E (DLPNO-CCSD(T)tight/def-QZVPP//TPSSh/def2-TZVP) =	
E (DLPNO-CCSD(T)/CBS//TPSSh/def2-TZVP) =	−896.535960355

N	0.05298317	−0.19596113	0.24147838
C	0.02303195	−0.07206217	1.59089171
N	1.18427960	0.16912426	2.12013817
N	1.99850201	0.18114696	0.97752858
C	1.33148736	−0.07164304	−0.17083542
C	3.38687940	0.41239364	1.11585613
C	1.77546213	−0.59720570	−1.57743320
O	0.87375351	−1.44184362	−2.01970011
C	−1.26679084	−0.49945145	−0.31185237
H	−1.13561329	−1.34582982	−0.98799834
C	−1.90254536	0.68725929	−1.07453605
O	−1.18050854	−0.27345676	2.11186415
H	1.97608032	0.31974568	−2.19558716
H	2.79325426	−1.01369624	−1.36047291
C	4.11837100	0.83865344	0.00936175
C	5.48756015	1.03671362	0.14283476
C	6.11360590	0.82928524	1.36712778
C	5.36406693	0.42066655	2.46759625
C	3.99780879	0.20566150	2.35020276
H	3.62015331	1.03760506	−0.93101686
H	6.06128928	1.36688082	−0.71468273
H	7.18002694	0.99211604	1.46625448
H	5.84592861	0.26198570	3.42493547
H	3.40035601	−0.11464259	3.19237733
C	−1.97492252	−0.87355514	1.01827041
H	−1.97880105	−1.95036277	1.18906919
H	−2.97957841	−0.47195854	1.10975419
C	−3.23707554	0.18747508	−1.64406562
C	−2.13627943	1.89234577	−0.15536581
C	−0.98173782	1.09704077	−2.22999805
H	−3.72624938	0.99000541	−2.20056610
H	−3.07717041	−0.65110308	−2.32636481
H	−3.92691768	−0.13493162	−0.85796404
H	−2.55248617	2.71864631	−0.73626293
H	−2.83699937	1.67415668	0.65503224
H	−1.19943115	2.24155352	0.28735801
H	−1.50468298	1.80077285	−2.88280964
H	−0.08715230	1.60058818	−1.85349710
H	−0.65543239	0.22396329	−2.79923749

2(formaldehyde)

E (TPSSh/def2-TZVP) =	−1073.09869435
$G - E$ (TPSSh/def2-TZVP) =	0.39830690
$H - E$ (TPSSh/def2-TZVP) =	0.48195269
E (DLPNO-CCSD(T)/def-TZVPP//TPSSh/def2-TZVP) =	−1066.16835024
E (DLPNO-CCSD(T)/def-QZVPP//TPSSh/def2-TZVP) =	−1066.21448919
E (DLPNO-CCSD(T)tight/def-QZVPP//TPSSh/def2-TZVP) =	
E (DLPNO-CCSD(T)/CBS//TPSSh/def2-TZVP) =	−1071.22422325

O	2.96719422	0.74812261	−1.64949355
C	2.29539891	1.14653041	−0.58846393
N	0.96403725	1.33533615	−0.81407788
C	0.60056667	0.95347497	−2.19314845
C	2.03905517	0.87708772	−2.78509728
C	0.42935113	1.75002527	0.34882150
N	1.44609012	1.78217443	1.20639118
N	2.65214007	1.40475986	0.63704571
C	1.39878603	2.16112143	2.58241353
C	0.25208594	1.88402619	3.31899686
C	0.20104849	2.28702709	4.64756489
C	1.28781322	2.93729577	5.22562652
C	2.43437381	3.18466428	4.47542879
C	2.49901913	2.79965263	3.14228005
C	−0.22183539	−0.35225560	−2.28497424
C	−1.54557792	−0.16753018	−1.53405112
C	−0.51767089	−0.58852967	−3.77353840
C	0.54736364	−1.54058994	−1.69686101
N	−2.22477548	3.16007829	0.80376710
C	−2.69970688	3.55291806	−0.52429190
C	−3.26934985	2.42952782	1.52541922
C	−1.80420432	4.34466732	1.55838739
O	−0.02675424	−0.94864298	1.70609826
C	0.28132659	−1.78698965	2.51909759
H	−3.54921905	4.24970213	−0.47110638
H	−0.59996687	2.10650994	0.54145784
H	0.04183478	1.76942489	−2.65358075
H	−1.89150350	4.04333470	−1.07262973
H	−3.01870688	2.66748774	−1.07643317
H	−4.17844807	3.03381975	1.66188211
H	−3.53295426	1.52647969	0.97177880
H	−2.89864050	2.14197831	2.51074610
H	−2.62863642	5.05965973	1.69552655
H	−1.43697074	4.04341042	2.54012076
H	−0.99727039	4.85046075	1.02340042
H	0.08664316	−1.64823925	3.59729665
H	0.77307643	−2.72983601	2.22184865
H	2.19484414	0.00775935	−3.41547747
H	2.31436080	1.78677324	−3.31769583
H	3.38001313	2.98477299	2.54266669
H	3.28239574	3.68434779	4.92657096
H	1.24527575	3.24220677	6.26385474
H	−0.68349448	2.07639319	5.23617357
H	−0.56251085	1.33412656	2.86678161
H	−1.13275150	−1.48302008	−3.88439218
H	−1.06532579	0.25165490	−4.20955751
H	0.39270544	−0.74556459	−4.35741612
H	−0.05750350	−2.44402250	−1.79494904
H	1.48971680	−1.72197355	−2.21931016
H	0.75086178	−1.39558104	−0.63418227
H	−2.17053908	−1.05165101	−1.67247996
H	−1.38855312	−0.04670187	−0.46039734
H	−2.09853035	0.69468177	−1.91598439

$\mathbf{TS_{2\text{-}IV}}$(formaldehyde)

E (TPSSh/def2-TZVP) =	−1073.07090064
$G - E$ (TPSSh/def2-TZVP) =	0.40666852
$H - E$ (TPSSh/def2-TZVP) =	0.48107492
E (DLPNO-CCSD(T)/def-TZVPP//TPSSh/def2-TZVP) =	−1066.13026506
E (DLPNO-CCSD(T)/def-QZVPP//TPSSh/def2-TZVP) =	−1066.17643908
E (DLPNO-CCSD(T)tight/def-QZVPP//TPSSh/def2-TZVP) =	
E (DLPNO-CCSD(T)/CBS//TPSSh/def2-TZVP) =	−1071.19514106

C	−0.85177244	4.34087531	0.04211424
C	0.15835630	3.94198922	−0.83263172
C	0.66388843	4.83247626	−1.77712625
C	0.15246177	6.12325390	−1.83942267
C	−0.85126616	6.53391858	−0.96892802
C	−1.34684699	5.63762619	−0.02759431
N	0.68140851	2.61581237	−0.78228224
N	1.54088607	2.24142571	−1.82319242
C	1.81076781	1.02314849	−1.48680659
N	1.19838688	0.63315801	−0.33055960
C	0.43657260	1.66456411	0.15336529
C	1.44192438	−0.80549207	−0.10270978
C	2.60397455	−1.01703935	−1.10720815
O	2.53509007	0.08204930	−2.07297242
O	−1.32105961	2.22090125	2.63677842
C	−0.10721344	2.33583589	2.48631854
N	−3.00091370	1.44538134	0.67295552
C	−4.04942526	2.44411288	1.04807458
C	−2.78316064	1.40878746	−0.80301900
C	−3.31734304	0.09357418	1.22428542
H	−4.25732017	−0.24967941	0.79492002
H	−2.13082421	1.75385388	1.15572140
H	0.55705040	−1.35279882	−0.44542281
C	1.75085413	−1.24113520	1.34200732
H	−2.50811226	−0.58302176	0.95974454
H	−3.39263438	0.17636499	2.30581816
H	−5.00597203	2.11258925	0.64720312
H	−4.08360756	2.50949678	2.13270577
H	−3.77306401	3.40662406	0.62336753
H	−3.71579548	1.11520091	−1.28318623
H	−2.47890684	2.39965894	−1.13323607
H	−1.99847153	0.68863220	−1.01921038
H	0.34918769	3.28043482	2.15982311
H	0.58695940	1.57033053	2.85522834
H	3.57693910	−0.95477108	−0.61935582
H	2.52359855	−1.94279529	−1.66909276
H	1.44572968	4.50897377	−2.44857835
H	0.54975721	6.81222155	−2.57463654
H	−1.24053235	7.54299169	−1.01985040
H	−2.11984661	5.94812203	0.66573168
H	−1.23593997	3.66153616	0.78939590
C	2.28531805	−2.68151327	1.29284298
C	2.78839430	−0.32295847	1.99540914
C	0.43928560	−1.23725044	2.13958904
H	2.41216380	−3.06070896	2.30858989
H	1.58932358	−3.34666047	0.77342834
H	3.25730809	−2.74201868	0.79729028
H	2.98241633	−0.65662304	3.01644163
H	3.74079110	−0.33910520	1.46018747
H	2.44438527	0.71208329	2.03420837
H	0.62878873	−1.48747129	3.18534474
H	−0.05194006	−0.26421433	2.10444466
H	−0.25012966	−1.98649516	1.73805609

IV(formaldehyde)

E (TPSSh/def2-TZVP) =	−1073.11819453
$G - E$ (TPSSh/def2-TZVP) =	0.40849523
$H - E$ (TPSSh/def2-TZVP) =	0.48230281
E (DLPNO-CCSD(T)/def-TZVPP//TPSSh/def2-TZVP) =	−1066.17526348
E (DLPNO-CCSD(T)/def-QZVPP//TPSSh/def2-TZVP) =	−1066.22130636
E (DLPNO-CCSD(T)tight/def-QZVPP//TPSSh/def2-TZVP) =	
E (DLPNO-CCSD(T)/CBS//TPSSh/def2-TZVP) =	−1071.24638804

C	−1.05449913	4.51168528	−0.06257212
C	0.13695640	4.09704317	−0.64677363
C	0.98757991	4.98677128	−1.29158470
C	0.63919136	6.33076144	−1.32982795
C	−0.53895226	6.76913416	−0.73126855
C	−1.38399336	5.86102851	−0.10083765
N	0.49900415	2.70823481	−0.62566271
N	0.93250436	2.13010819	−1.80601539
C	1.18203749	0.91054001	−1.42743088
N	0.96615583	0.69097895	−0.09685870
C	0.51394268	1.86370559	0.41183559
C	1.16111505	−0.75532499	0.22254675
C	1.84879571	−1.18259840	−1.10466152
O	1.55768905	−0.15054885	−2.10603563
O	−1.03983168	1.34684597	2.14369793
C	0.06302092	2.14358202	1.81154728
N	−3.17675005	1.27184873	0.47230367
C	−4.22080658	2.27845265	0.71047570
C	−2.86206193	1.18431861	−0.95798870
C	−3.62087373	−0.03826616	0.97406555
H	−4.52811248	−0.38088033	0.45896225
H	−1.80260308	1.47172735	1.48751287
H	0.16537493	−1.19761041	0.30389146
C	1.98588623	−1.11733464	1.48119054
H	−2.82892954	−0.77278521	0.81900455
H	−3.82477292	0.03352194	2.04264276
H	−5.16484169	2.00545903	0.21981205
H	−4.39548383	2.37195175	1.78280080
H	−3.89814809	3.24369917	0.31724881
H	−3.73452556	0.87859644	−1.55134610
H	−2.52004343	2.15361049	−1.32390981
H	−2.07117835	0.44670548	−1.11172989
H	−0.12280074	3.21697110	1.90510233
H	0.87667150	1.89203211	2.49643208
H	2.93206290	−1.21823914	−1.00242050
H	1.47330020	−2.12264490	−1.49658356
H	1.89509850	4.62663367	−1.75774741
H	1.29215893	7.03632706	−1.82781502
H	−0.80323295	7.81870232	−0.76403659
H	−2.30871594	6.19827395	0.35049500
H	−1.71948529	3.79335270	0.39810159
C	2.46175835	−2.57341063	1.30845317
C	3.19581105	−0.19087048	1.63514138
C	1.09731600	−1.07769705	2.73136314
H	2.93034221	−2.90369765	2.23671100
H	1.62267752	−3.24512931	1.10671597
H	3.20146358	−2.68772014	0.51320522
H	3.80959533	−0.53042889	2.47077642
H	3.82980661	−0.18585838	0.74420210
H	2.89668824	0.83911755	1.84320737
H	1.69745043	−1.32487167	3.60910013
H	0.62571701	−0.11188059	2.89671444
H	0.29711817	−1.81832268	2.65412913

II(acetaldehyde)

E (TPSSh/def2-TZVP) =	−937.44077938
$G - E$ (TPSSh/def2-TZVP) =	0.29973194
$H - E$ (TPSSh/def2-TZVP) =	0.36976102
E (DLPNO-CCSD(T)/def-TZVPP//TPSSh/def2-TZVP) =	−931.45901841
E (DLPNO-CCSD(T)/def-QZVPP//TPSSh/def2-TZVP) =	−931.49881205
E (DLPNO-CCSD(T)tight/def-QZVPP//TPSSh/def2-TZVP) =	
E (DLPNO-CCSD(T)/CBS//TPSSh/def2-TZVP) =	−935.810239096

C	0.19777474	−0.80713246	−0.04208139
N	0.07170860	−0.43136435	1.18809825
N	1.26543365	0.30844208	1.33895720
C	2.08169122	0.37046724	0.25671080
N	1.34568820	−0.36729698	−0.62707154
C	1.53272851	0.89224661	2.60187131
C	1.42031045	−0.77863754	−2.02840654
C	4.68077281	−0.86434791	−0.26403425
C	4.83115472	−1.26878897	1.17315588
O	4.36937912	−1.62557426	−1.15711287
H	2.35771781	−1.30922733	−2.19658187
C	1.32682687	0.40228541	−3.02187615
O	−0.57418540	−1.53821301	−0.84298998
H	5.89549870	−1.23349924	1.43473733
H	4.31470275	−0.55408264	1.81731079
H	4.45142831	−2.27703199	1.33451860
H	4.92200088	0.19120383	−0.48827449
C	2.62293825	1.75177294	2.74030191
C	2.89263594	2.31405811	3.98031066
C	2.08272241	2.03318887	5.07823255
C	0.99474175	1.18102609	4.92415139
C	0.71301856	0.60501360	3.69035386
H	3.23295482	1.96904040	1.87355209
H	3.73867499	2.98334336	4.08617885
H	2.29612299	2.47864689	6.04257058
H	0.35553903	0.95717369	5.77039603
H	−0.12915939	−0.05924904	3.55776463
C	0.22209138	−1.77080293	−2.05420607
H	0.55722088	−2.80773909	−2.02306438
H	−0.44534226	−1.62087098	−2.89873627
C	1.31825407	−0.17289886	−4.44462661
C	0.06296932	1.23277795	−2.77587582
C	2.57202781	1.28199369	−2.85104614
H	1.34115773	0.64151407	−5.17241382
H	2.19554927	−0.80288507	−4.61684947
H	0.42310170	−0.76749537	−4.64716849
H	0.00493516	2.04481764	−3.50445738
H	−0.84675128	0.63346359	−2.87493211
H	0.07200677	1.67842577	−1.77866576
H	2.53453998	2.11833506	−3.55411046
H	2.63728963	1.67881597	−1.83602985
H	3.48027853	0.70593515	−3.04723101

$\mathbf{TS_{II\text{-}III}}$(acetaldehyde)

E (TPSSh/def2-TZVP) =	−937.41970327
$G - E$ (TPSSh/def2-TZVP) =	0.30506238
$H - E$ (TPSSh/def2-TZVP) =	0.36948043
E (DLPNO-CCSD(T)/def-TZVPP//TPSSh/def2-TZVP) =	−931.40857580
E (DLPNO-CCSD(T)/def-QZVPP//TPSSh/def2-TZVP) =	−931.44845511
E (DLPNO-CCSD(T)tight/def-QZVPP//TPSSh/def2-TZVP) =	
E (DLPNO-CCSD(T)/CBS//TPSSh/def2-TZVP) =	−935.78227786

N	−0.06657725	−0.10246255	0.11080867
N	−0.12041495	−0.25813349	1.50139560
C	1.05249041	−0.10977016	2.15048456
N	1.91043348	0.17884516	1.11584311
C	1.19498065	0.13096624	−0.04619001
C	−1.38218514	−0.55634470	2.07702487
C	3.36002338	0.27698061	0.81537714
C	1.07609875	0.59254144	3.89468229
O	1.57751958	1.75060099	3.80573724
C	1.75929495	−0.46851381	4.75804333
C	4.20293378	1.38115427	1.49805388
H	3.81026597	−0.69332170	1.04300615
O	1.91187450	0.24397408	−1.15418050
H	1.69467911	−0.13883150	5.79922048
H	1.27396035	−1.44431724	4.67007351
H	2.81279109	−0.57640062	4.50310703
H	−0.02754417	0.51608966	3.99009349
C	−2.53364490	−0.02968329	1.49949678
C	−3.76688354	−0.31926522	2.06853566
C	−3.85023764	−1.12267727	3.20255664
C	−2.69174564	−1.65064947	3.76324068
C	−1.45235594	−1.37745436	3.19788745
H	−2.44971080	0.59280912	0.61926577
H	−4.66655425	0.09090012	1.62555636
H	−4.81547025	−1.34246010	3.64277388
H	−2.74949065	−2.28793247	4.63744398
H	−0.54606237	−1.80705204	3.60233897
C	3.28310103	0.49074299	−0.72599996
H	3.92192284	−0.19308048	−1.28008843
H	3.51526450	1.51882669	−0.99593885
C	4.48463574	1.02260604	2.96007893
C	5.55203712	1.42246822	0.75448228
C	3.50000864	2.74051254	1.42984559
H	5.25009388	1.69614130	3.35480747
H	3.58338643	1.15169798	3.56026728
H	4.86439246	−0.00090501	3.04860850
H	4.15299130	3.50437301	1.85890482
H	3.27751706	3.04296210	0.40158659
H	2.57841497	2.71689287	2.01483085
H	6.21304449	2.12754100	1.26189268
H	6.04153313	0.44308308	0.76482300
H	5.46165189	1.75226395	−0.28386177

III(acetaldehyde)

E (TPSSh/def2-TZVP) =	−937.42720333
$G - E$ (TPSSh/def2-TZVP) =	0.30673279
$H - E$ (TPSSh/def2-TZVP) =	0.37096278
E (DLPNO-CCSD(T)/def-TZVPP//TPSSh/def2-TZVP) =	−931.41509725
E (DLPNO-CCSD(T)/def-QZVPP//TPSSh/def2-TZVP) =	−931.45507751
E (DLPNO-CCSD(T)tight/def-QZVPP//TPSSh/def2-TZVP) =	
E (DLPNO-CCSD(T)/CBS//TPSSh/def2-TZVP) =	−935.791647366

N	−0.01303105	−0.16461568	−0.10691802
C	−0.21643885	−0.61486134	1.14771101
N	0.99306270	−0.41236402	1.75970121
C	1.80477903	0.18478156	0.83871417
N	1.26156844	0.38347721	−0.31693293
C	−1.55589901	−0.89765946	1.79143637
C	−1.55198299	−2.16923961	2.65832241
C	1.68121213	−0.48002914	3.07138631
C	−0.93159922	−0.16641959	−1.18855579
O	−1.73057135	0.28260221	2.41385269
C	1.25942806	0.59504776	4.12087862
H	1.55069062	−1.47382300	3.50121528
O	3.03417210	0.42304814	1.28111791
H	−2.51654234	−2.22980147	3.16635742
H	−1.41579540	−3.07137052	2.05099502
H	−0.77518493	−2.13787845	3.42442523
H	−2.26639496	−1.08561078	0.95909630
C	−1.05976860	0.98372731	−1.96000672
C	−1.95620299	0.98908609	−3.02031390
C	−2.72029563	−0.14045724	−3.30156310
C	−2.57434300	−1.28771578	−2.52863946
C	−1.67004269	−1.31088695	−1.47330627
H	−0.46108493	1.85253757	−1.72132641
H	−2.06468967	1.88305045	−3.62261445
H	−3.42241354	−0.12870757	−4.12661272
H	−3.15474250	−2.17445472	−2.75332753
H	−1.52200820	−2.20883409	−0.88764636
C	3.12749011	−0.34239361	2.52490318
H	3.54639785	−1.31888381	2.27215394
H	3.80078128	0.19781000	3.17848405
C	0.03359764	0.10632512	4.90358172
C	2.41389377	0.77628794	5.12336123
C	0.93557173	1.92816093	3.43785661
H	0.72878460	2.68300628	4.20057081
H	1.77345107	2.28926765	2.83318503
H	0.04366606	1.81912618	2.81620471
H	−0.19293629	0.82547853	5.69559316
H	−0.83432832	0.03335801	4.24265194
H	0.23549746	−0.85981647	5.38029862
H	2.04272711	1.33523710	5.98425066
H	2.78878624	−0.18410678	5.49408666
H	3.25224444	1.34152404	4.70858237

2(acetaldehyde)

E (TPSSh/def2-TZVP) =	−1112.44988595
$G - E$ (TPSSh/def2-TZVP) =	0.42577766
$H - E$ (TPSSh/def2-TZVP) =	0.50789928
E (DLPNO-CCSD(T)/def-TZVPP//TPSSh/def2-TZVP) =	−1105.23305862
E (DLPNO-CCSD(T)/def-QZVPP//TPSSh/def2-TZVP) =	−1105.28102188
E (DLPNO-CCSD(T)tight/def-QZVPP//TPSSh/def2-TZVP) =	
E (DLPNO-CCSD(T)/CBS//TPSSh/def2-TZVP) =	−1110.49836598

C	3.27097140	3.68291285	−3.43299384
C	2.26445239	3.90229288	−2.49995678
C	1.57705357	5.10907537	−2.42509951
C	1.89332188	6.11205270	−3.33293007
C	2.89137612	5.91022576	−4.28227545
C	3.58121654	4.70155915	−4.32513851
N	1.92259320	2.85474147	−1.59260071
C	0.72243706	2.60118124	−1.07818015
N	0.90055349	1.53153001	−0.28213479
C	2.21837189	1.19299140	−0.36882692
N	2.89974633	1.97164468	−1.15930789
O	2.54949692	0.14115016	0.35281417
C	1.28026035	−0.43972291	0.82154363
C	0.19305698	0.66644152	0.68238212
C	−0.19470515	1.38008434	1.99760648
C	−1.14911534	2.53737381	1.68124811
C	−0.92674327	0.34534991	2.86508934
C	1.04147761	1.91578698	2.72844652
N	−1.88861828	3.68848014	−2.19870656
C	−1.60118275	3.78879507	−3.63274663
C	−2.36136756	4.97140002	−1.67314372
C	−2.88085082	2.63926888	−1.95824137
O	1.52545981	4.80548794	0.80190509
C	2.42045487	5.60056075	1.00557393
C	3.23054710	5.66798368	2.25802337
H	−0.70291140	0.26288030	0.20873577
H	−3.82548557	2.83284993	−2.48770172
H	−0.22561646	3.09971035	−1.35277636
H	−2.48910248	1.67864406	−2.30196560
H	−3.09552869	2.57464290	−0.89035196
H	−3.30200125	5.29123695	−2.14563545
H	−2.52400342	4.88791016	−0.59708866
H	−1.60798903	5.73969644	−1.85516788
H	−2.50033905	4.03482390	−4.21659658
H	−0.85184765	4.56236381	−3.80401240
H	−1.20802106	2.83575176	−3.99366454
H	2.68312527	6.34066453	0.22314546
H	4.28657545	5.51633434	2.00912438
H	3.15717810	6.67506241	2.68176506
H	2.90406578	4.92816574	2.98681694
H	0.84082057	5.26372575	−1.64748267
H	1.36973088	7.05913310	−3.28617248
H	3.13847926	6.69840542	−4.98259060
H	4.36179251	4.54634366	−5.05929865
H	3.79396664	2.73631041	−3.45196365
H	1.07382759	−1.30236896	0.18883894
H	1.44082288	−0.75303155	1.84789272
H	−1.25500970	0.81593812	3.79323879
H	−1.81179385	−0.04639760	2.35593345
H	−0.28543975	−0.49700219	3.13650381
H	−1.50347616	2.98295191	2.61258267
H	−0.65218751	3.32654519	1.11392316
H	−2.02331525	2.18691585	1.12619204
H	0.72939074	2.38814672	3.66188601
H	1.74742060	1.12219160	2.98512234
H	1.55455368	2.67396320	2.13414280

$\mathbf{TS_{2\text{-}IV}}$(acetaldehyde)

E (TPSSh/def2-TZVP) =	−1112.41894423
$G - E$ (TPSSh/def2-TZVP) =	0.43020551
$H - E$ (TPSSh/def2-TZVP) =	0.51167133
E (DLPNO-CCSD(T)/def-TZVPP//TPSSh/def2-TZVP) =	−1105.18655194
E (DLPNO-CCSD(T)/def-QZVPP//TPSSh/def2-TZVP) =	−1105.23441054
E (DLPNO-CCSD(T)tight/def-QZVPP//TPSSh/def2-TZVP) =	
E (DLPNO-CCSD(T)/CBS//TPSSh/def2-TZVP) =	−1110.46404619

C	4.32417840	4.40017679	−2.10032653
C	3.00407014	4.01332455	−2.30357405
C	2.19033276	4.67999064	−3.21292105
C	2.69490560	5.77917365	−3.89870157
C	4.00786087	6.18910050	−3.68901761
C	4.82160051	5.49333187	−2.79871429
N	2.48729293	2.89893825	−1.57997570
C	1.33908393	2.86040206	−0.87604266
N	1.35118675	1.56410171	−0.42916201
C	2.48446745	0.94891312	−0.88425750
N	3.24052161	1.71791036	−1.59942482
O	2.60078658	−0.32168925	−0.53185249
C	1.31298144	−0.69054886	0.05397741
C	0.56600980	0.63091204	0.40358045
C	0.53261458	0.97640011	1.91088715
C	−0.35798728	2.20433473	2.11176299
C	−0.10211990	−0.20174758	2.66691009
C	1.94537463	1.24309510	2.44039650
N	−2.71994648	4.49131729	−1.41869549
C	−3.33474884	3.21064226	−0.98077777
C	−2.29726214	4.43003114	−2.84212721
C	−3.62381934	5.64511324	−1.16297242
O	−0.70999106	4.78276387	0.13410696
C	0.46943134	4.88305722	−0.28487472
C	1.57478846	5.22571164	0.67078267
H	−0.45863603	0.58595392	0.02910490
H	−4.53948186	5.53066755	−1.74403594
H	−1.80572575	4.63791841	−0.79074161
H	−3.85532396	5.67587921	−0.10002267
H	−3.11091894	6.56091407	−1.45112299
H	−3.16645428	4.26198884	−3.47882329
H	−1.82249087	5.37315727	−3.10781431
H	−1.58692993	3.61290453	−2.95898574
H	−4.24001707	3.01955604	−1.55792711
H	−2.61473198	2.40964690	−1.13974327
H	−3.57349267	3.28374356	0.07844559
H	0.64906931	5.14895020	−1.33007794
H	2.55784007	5.08480384	0.22445878
H	1.46004318	6.28779812	0.91782652
H	1.48836358	4.65897970	1.59685437
H	1.18769146	4.31410301	−3.39813083
H	2.07053154	6.30034091	−4.61433357
H	4.40216107	7.03977858	−4.23099312
H	5.84835351	5.80219854	−2.64632936
H	4.94501538	3.84263136	−1.41105404
H	0.77538070	−1.27839816	−0.69043719
H	1.52930676	−1.30214568	0.92367491
H	−0.28513852	0.09243238	3.70210300
H	−1.06383481	−0.48519349	2.22821405
H	0.53916967	−1.08497842	2.69116913
H	−0.35995224	2.49969423	3.16311994
H	−0.02999271	3.05524928	1.51885509
H	−1.39112493	1.97930374	1.82790990
H	1.90773089	1.43105956	3.51503220
H	2.60867828	0.38928960	2.27748558
H	2.39238640	2.11827493	1.96326470

IV(acetaldehyde)

E (TPSSh/def2-TZVP) =	−1112.46013430
$G - E$ (TPSSh/def2-TZVP) =	0.43256154
$H - E$ (TPSSh/def2-TZVP) =	0.51391837
E (DLPNO-CCSD(T)/def-TZVPP//TPSSh/def2-TZVP) =	−1105.23503279
E (DLPNO-CCSD(T)/def-QZVPP//TPSSh/def2-TZVP) =	−1105.28277746
E (DLPNO-CCSD(T)tight/def-QZVPP//TPSSh/def2-TZVP) =	
E (DLPNO-CCSD(T)/CBS//TPSSh/def2-TZVP) =	−1110.51454499

C	4.27494330	4.62043245	−1.82199469
C	3.00601909	4.22734813	−2.22652699
C	2.34861848	4.83597109	−3.28793161
C	2.97584122	5.88664186	−3.94783171
C	4.24340603	6.30136239	−3.55015565
C	4.89367785	5.66695021	−2.49496202
N	2.35960593	3.14881487	−1.52953313
C	1.33295635	3.24548740	−0.67922263
N	1.06495418	1.97241599	−0.31851705
C	1.93251704	1.17572901	−1.01470128
N	2.76329632	1.83859458	−1.76408806
O	1.73516927	−0.11519434	−0.82971887
C	0.43604091	−0.21126327	−0.14168166
C	0.23593013	1.13986280	0.59042223
C	0.71823873	1.12517653	2.06323209
C	0.72156917	2.54063465	2.64289270
C	−0.29631099	0.27002991	2.83908057
C	2.12568400	0.52898074	2.19683241
N	−2.65732306	3.97571651	−1.57632995
C	−3.23894538	2.63684446	−1.42584231
C	−2.22503479	4.19295816	−2.96176205
C	−3.62333803	5.00655614	−1.16729126
O	−0.67617923	4.11199217	0.23906792
C	0.53254093	4.49283114	−0.35377649
C	1.29957216	5.48672796	0.51625136
H	−0.79464314	1.48308057	0.53933861
H	−4.52903401	4.98170137	−1.78773414
H	−1.40193514	4.07037032	−0.47430059
H	−3.90331515	4.84500406	−0.12586535
H	−3.16107822	5.99023708	−1.25711432
H	−3.06230015	4.11784737	−3.66897056
H	−1.78407092	5.18698502	−3.05380137
H	−1.47840794	3.44272588	−3.23251974
H	−4.13048232	2.50413904	−2.05337968
H	−2.49983426	1.88624163	−1.71528413
H	−3.51967504	2.47856597	−0.38333088
H	0.35774855	4.97166299	−1.32637871
H	2.24953163	5.75983250	0.05329276
H	0.68848363	6.38291388	0.62221997
H	1.48558734	5.07578998	1.50676594
H	1.37401750	4.48201861	−3.60178929
H	2.47969700	6.37098747	−4.77949485
H	4.73038476	7.11706804	−4.07001556
H	5.88390340	5.98563536	−2.19469155
H	4.76517988	4.11185497	−1.00145325
H	−0.31899786	−0.38453126	−0.90956934
H	0.49828698	−1.06212509	0.52782072
H	−0.00723343	0.22930210	3.89042367
H	−1.29941445	0.70101976	2.78269634
H	−0.34246141	−0.75926106	2.47438091
H	0.92428458	2.49077407	3.71439428
H	1.51502032	3.14041383	2.19245124
H	−0.22846289	3.05140989	2.48622614
H	2.42563922	0.55429980	3.24589403
H	2.17899759	−0.51006846	1.86589639
H	2.86437136	1.10699979	1.63435648

5.5 Reactions: Precursor + Amine + Formaldehyde + Chloride

ion pair with ammonium

E (TPSSh/def2-TZVP) =	−635.41723218
$G - E$ (TPSSh/def2-TZVP) =	0.09971747
$H - E$ (TPSSh/def2-TZVP) =	0.13921225
E (DLPNO-CCSD(T)tight/def-TZVPP//TPSSh/def2-TZVP) =	−633.44884175
E (DLPNO-CCSD(T)tight/def-QZVPP//TPSSh/def2-TZVP) =	−633.47076994
E (DLPNO-CCSD(T)/CBS//TPSSh/def2-TZVP) =	−634.65213833

N	0.00836424	−0.08260777	0.00584754
C	−0.03393613	0.01779993	1.47643389
C	1.38079376	0.01772472	−0.52412818
C	−0.70119137	−1.27394009	−0.49583873
H	−0.59733445	0.88457860	−0.42248693
H	−0.70582848	−1.24077550	−1.58432687
H	−0.21229632	−2.18885152	−0.15008441
H	−1.72897633	−1.24067741	−0.13741311
H	−1.07597876	0.02377035	1.79271531
H	0.49333756	−0.82250273	1.93631875
H	0.42999804	0.95756543	1.77226509
H	1.33172451	0.02382035	−1.61200550
H	1.81440775	0.95742423	−0.18520930
H	1.99003491	−0.82266330	−0.18032287
Cl	−1.39898994	2.16516372	−0.98878768

5.5.1 Imidazolium

ion pair

E (TPSSh/def2-TZVP) =	−765.82197523
$G - E$ (TPSSh/def2-TZVP) =	0.10544015
$H - E$ (TPSSh/def2-TZVP) =	0.14914537
E (DLPNO-CCSD(T)tight/def-TZVPP//TPSSh/def2-TZVP) =	−763.08599791
E (DLPNO-CCSD(T)tight/def-QZVPP//TPSSh/def2-TZVP) =	−763.11354410
E (DLPNO-CCSD(T)/CBS//TPSSh/def2-TZVP) =	−764.824044939

C	1.16957166	−0.11660493	−0.52666979
N	−0.06665257	0.00546759	−0.02797475
C	0.00539928	0.18975364	1.34223084
C	1.32443512	0.17802458	1.66729709
N	2.02613419	−0.01226238	0.49290903
C	−1.28118525	−0.05006586	−0.83800316
C	3.48328404	−0.08875063	0.32258266
H	1.52505398	−0.25343372	−1.59082509
H	1.81582561	0.28801239	2.61816619
H	−0.87183586	0.31153287	1.95338883
H	3.66999683	−0.19877613	−0.75244192
H	3.93309492	0.82864206	0.70146515
H	3.86257531	−0.94934859	0.87337262
H	−1.89864858	−0.89024749	−0.52017314
H	−1.83735252	0.88109809	−0.73052222
H	−0.98369187	−0.18285405	−1.87565955
Cl	2.79069773	−0.36179343	−3.06183080

2

E (TPSSh/def2-TZVP) =	−1054.97068480
$G-E$ (TPSSh/def2-TZVP) =	0.24417037
$H-E$ (TPSSh/def2-TZVP) =	0.31154082
E (DLPNO-CCSD(T)tight/def-TZVPP//TPSSh/def2-TZVP) =	−1050.35328741
E (DLPNO-CCSD(T)tight/def-QZVPP//TPSSh/def2-TZVP) =	−1050.39325698
E (DLPNO-CCSD(T)/CBS//TPSSh/def2-TZVP) =	−1053.49186091

C	2.00732339	2.39502761	−1.22923115
C	2.27440852	1.40735441	−0.33836423
N	1.05845212	0.87228038	0.04333032
C	0.07702106	1.54739852	−0.56000032
N	0.63673824	2.44280916	−1.38327192
C	0.85650781	−0.16008204	1.05916308
C	−0.11208081	3.50338610	−2.04965723
C	0.00033016	2.53068350	3.71136361
O	0.04467444	1.31408746	3.70946746
N	−2.95250886	0.96793753	−0.00052014
C	−3.55039198	2.22808557	−0.43893057
C	−3.62822278	−0.17340055	−0.60121272
C	−2.96639127	0.88572091	1.46126329
H	1.65439672	−0.89604708	0.97018334
H	−4.69337156	−0.22988502	−0.31696255
H	2.66174323	3.05991604	−1.76397178
H	−0.99086444	1.34746306	−0.43689032
H	−0.10218086	−0.63871975	0.87330612
H	0.84758105	0.29611204	2.05169037
H	0.53760682	3.98051985	−2.78075421
H	−0.97525711	3.07197856	−2.55355107
H	−0.43738687	4.20679617	−1.27845603
H	3.20610083	1.03431665	0.04756666
H	−3.56917190	−0.10650943	−1.69065118
H	−3.14231078	−1.09866193	−0.28145314
H	−3.99474698	0.89622164	1.86046537
H	−2.48535883	−0.04083208	1.78251610
H	−2.40894819	1.73165125	1.86474906
H	−4.61077206	2.30593304	−0.14410583
H	−2.99167332	3.05393175	0.00616665
H	−3.49694848	2.29652988	−1.52959104
H	−0.91279267	3.07932041	3.98793766
H	0.88791518	3.15108389	3.51601370
Cl	−0.63185888	3.67571451	1.26995964

$TS_{2\text{-}IV}$

E (TPSSh/def2-TZVP) =	−1054.94131998
$G - E$ (TPSSh/def2-TZVP) =	0.24711614
$H - E$ (TPSSh/def2-TZVP) =	0.31177166
E (DLPNO-CCSD(T)tight/def-TZVPP//TPSSh/def2-TZVP) =	−1050.32250344
E (DLPNO-CCSD(T)tight/def-QZVPP//TPSSh/def2-TZVP) =	−1050.36238583
E (DLPNO-CCSD(T)/CBS//TPSSh/def2-TZVP) =	−1053.46683521

C	1.98756201	2.18112587	−1.49849601
C	2.25342856	0.96250120	−0.97143563
N	1.16295806	0.64236580	−0.18248128
C	0.20282515	1.61849342	−0.18946575
N	0.74952532	2.55723734	−1.01218041
C	1.04369496	−0.60599622	0.55487801
C	0.09469620	3.81360934	−1.36498744
C	−0.19939536	1.87165824	2.66996198
O	−0.80224387	0.86517819	2.99651363
N	−2.90345394	1.48007750	0.55711032
C	−3.52078516	2.07850926	−0.65968454
C	−2.58479834	0.04326989	0.36592230
C	−3.76232686	1.69282708	1.76106231
H	2.01627700	−0.88060411	0.96540018
H	−3.51224574	−0.49416456	0.16614359
H	2.56749080	2.80607400	−2.15638108
H	−2.01342064	2.01392210	0.69729038
H	0.69644009	−1.41005581	−0.09924242
H	0.34071749	−0.46427235	1.37185288
H	0.83447270	4.61507745	−1.36156568
H	−0.34444299	3.73731355	−2.36310250
H	−0.67510432	4.02909859	−0.61880495
H	3.10950587	0.31748351	−1.07652728
H	−1.89429630	−0.04265152	−0.46908488
H	−2.11804790	−0.32348440	1.27651801
H	−4.71462533	1.18875546	1.59119723
H	−3.23968731	1.27008577	2.61660031
H	−3.87252463	2.76925542	1.88302177
H	−4.47951456	1.59182526	−0.83990818
H	−3.62406152	3.14546268	−0.46459769
H	−2.84335440	1.91323585	−1.49612760
H	−0.70932187	2.82276645	2.43464241
H	0.90723397	1.87215029	2.61861599
Cl	−2.11264717	4.40327947	1.20257002

IV

E (TPSSh/def2-TZVP) =	−1054.99533338
$G - E$ (TPSSh/def2-TZVP) =	0.25171876
$H - E$ (TPSSh/def2-TZVP) =	0.31403483
E (DLPNO-CCSD(T)tight/def-TZVPP//TPSSh/def2-TZVP) =	−1050.37163411
E (DLPNO-CCSD(T)tight/def-QZVPP//TPSSh/def2-TZVP) =	−1050.41136485
E (DLPNO-CCSD(T)/CBS//TPSSh/def2-TZVP) =	−1053.52339813

N	1.60644713	2.91424422	−0.60313419
C	2.53086208	2.14965006	−1.28542758
C	2.39675208	0.88131172	−0.83815455
N	1.38806105	0.87960048	0.10974484
C	0.86853439	2.11519843	0.19082938
C	0.78490758	−0.35150215	0.62198517
C	−0.17474670	2.65362717	1.13706909
O	−0.77516789	1.68135262	1.93317992
C	1.19587874	4.26411973	−0.96782925
C	−3.98502330	0.79059487	2.35864009
N	−3.26098610	1.03852434	1.11364581
C	−3.22885539	−0.16141993	0.27084054
C	−3.85094185	2.16091128	0.37414393
Cl	−1.05428169	1.74350211	−1.82938140
H	1.51815194	−1.14769800	0.50333721
H	−4.24471664	−0.48434389	−0.00585709
H	3.18981200	2.57738456	−2.01933926
H	−1.68599125	1.44239918	1.54655303
H	−0.10453273	−0.55978003	0.02587086
H	0.51461957	−0.22399800	1.66407294
H	1.06774481	4.87137842	−0.07197375
H	1.97116183	4.70286988	−1.59210538
H	0.25508738	4.18101682	−1.51997714
H	2.91853255	−0.02099001	−1.10123316
H	−2.65616704	0.06594749	−0.63065327
H	−2.74693877	−0.97322262	0.82154699
H	−5.03699716	0.52147410	2.17475595
H	−3.50641932	−0.02646048	2.90251357
H	−3.95726682	1.68732349	2.98078830
H	−4.90059738	1.95804301	0.10958568
H	−3.81881425	3.05759207	0.99802466
H	−3.26670890	2.32131479	−0.53421396
H	−0.89666051	3.19440424	0.51710933
H	0.33797255	3.37711101	1.79016268

5.5.2 Triazolium

ion pair

E (TPSSh/def2-TZVP) =	−781.85569411
$G - E$ (TPSSh/def2-TZVP) =	0.09321696
$H - E$ (TPSSh/def2-TZVP) =	0.13685242
E (DLPNO-CCSD(T)tight/def-TZVPP//TPSSh/def2-TZVP) =	−779.07410607
E (DLPNO-CCSD(T)tight/def-QZVPP//TPSSh/def2-TZVP) =	−779.10217783
E (DLPNO-CCSD(T)/CBS//TPSSh/def2-TZVP) =	−780.85023814

N	1.15511378	−0.10253142	−0.51889083
C	−0.10204242	−0.03080321	−0.04897147
N	0.01520776	0.15724977	1.25852378
N	1.32110236	0.20870910	1.66306837
C	1.99069734	0.04617410	0.55420613
C	−1.06583254	0.30694066	2.22272834
C	1.49336796	−0.29679997	−1.93802423
H	−1.00105509	−0.07878115	−0.74926451
H	3.06579282	0.03276043	0.48697325
H	0.54820675	−0.24026890	−2.49202521
H	2.17659172	0.49263726	−2.24790669
H	1.95848889	−1.27386136	−2.06364120
H	−1.01340569	−0.50297841	2.94881637
H	−0.96146392	1.26600604	2.72736746
H	−2.00403091	0.26641442	1.67408087
Cl	−1.89015180	−0.07156735	−2.43861444

2

E (TPSSh/def2-TZVP) =	−1070.99595285
$G - E$ (TPSSh/def2-TZVP) =	0.22859979
$H - E$ (TPSSh/def2-TZVP) =	0.29912246
E (DLPNO-CCSD(T)tight/def-TZVPP//TPSSh/def2-TZVP) =	−1066.33289985
E (DLPNO-CCSD(T)tight/def-QZVPP//TPSSh/def2-TZVP) =	−1066.37355905
E (DLPNO-CCSD(T)/CBS//TPSSh/def2-TZVP) =	−1069.50988717

C	0.51349306	2.51362771	−1.08224420
N	1.78914153	2.34155148	−1.50315263
C	2.38623873	1.51517447	−0.58766132
N	1.60568898	1.25459583	0.41851466
N	0.45591546	1.94892025	0.12973903
C	2.25355112	2.66084089	−2.84890313
C	−0.76797924	1.66662550	0.85790247
N	−1.64413889	4.51720052	−2.22233792
C	−2.86215864	3.75530032	−1.94632511
C	−1.27010795	4.38574297	−3.63096794
C	−1.80880969	5.91820713	−1.85726990
C	1.61281115	5.74879895	−0.46525470
O	1.82115222	5.81876928	−1.64918853
Cl	−0.70232451	0.88398409	−2.60120449
H	−0.49383248	1.33927453	1.85738123
H	−3.72111535	4.12872117	−2.52943637
H	3.39370882	1.14861006	−0.69147877
H	−0.20339464	3.23679751	−1.47128424
H	−1.31448651	0.88859550	0.32041477
H	−1.37020773	2.57275594	0.90606024
H	3.28631573	2.33035857	−2.94178837
H	1.61089450	2.12380063	−3.54962064
H	2.19653589	3.73606070	−3.00748691
H	−2.68593527	2.70622977	−2.19102547
H	−3.11074650	3.84167015	−0.88481144
H	−2.62934825	6.40184144	−2.41506189
H	−2.03439839	5.99641578	−0.79006098
H	−0.88574265	6.46215938	−2.06716580
H	−2.04126964	4.80163391	−4.30128937
H	−0.33617746	4.92520603	−3.80667429
H	−1.12946549	3.32754335	−3.85960712
H	2.19223863	5.07236904	0.19192699
H	0.82955647	6.35552018	0.02644414

$\mathbf{TS_{2\text{-}IV}}$

E (TPSSh/def2-TZVP) =	−1070.97381493
$G - E$ (TPSSh/def2-TZVP) =	0.23730083
$H - E$ (TPSSh/def2-TZVP) =	0.30089159
E (DLPNO-CCSD(T)tight/def-TZVPP//TPSSh/def2-TZVP) =	−1066.30379252
E (DLPNO-CCSD(T)tight/def-QZVPP//TPSSh/def2-TZVP) =	−1066.34447705
E (DLPNO-CCSD(T)/CBS//TPSSh/def2-TZVP) =	−1069.48867674

N	1.49232067	2.35740079	−1.40355636
C	1.04956827	1.07780717	−1.19633387
N	0.30220239	1.00944188	−0.13059036
N	0.26780799	2.31640172	0.30218494
C	0.97847756	3.18041211	−0.44551721
C	−0.47268003	2.62399758	1.50868472
C	2.35973818	2.77076003	−2.50682895
Cl	−0.17690438	1.36219217	−4.14222240
C	1.43576869	5.58033332	−0.68820845
O	1.07407514	5.93628795	−1.80186075
C	−1.15180405	4.57645915	−3.78888770
N	−1.40522681	4.23332083	−2.35014326
C	−1.93796736	5.39731997	−1.58896569
C	−2.29281491	3.03206787	−2.22661503
H	−0.01355357	2.12792843	2.36468562
H	−3.26053319	3.29153931	−2.65512365
H	1.26189253	0.25721663	−1.85953089
H	−0.49341485	3.99011536	−1.93276189
H	−1.50186540	2.27672828	1.40979182
H	−0.44626898	3.70225944	1.65137351
H	3.38186544	2.44344561	−2.30961931
H	1.96439434	2.31962514	−3.42014219
H	2.32619429	3.85526246	−2.57770712
H	−1.81119560	2.21503896	−2.78004068
H	−2.39826907	2.79604391	−1.16888902
H	−2.90525144	5.66474108	−2.01169367
H	−2.05598282	5.10431130	−0.54657920
H	−1.22385699	6.21254802	−1.67789982
H	−2.10188613	4.88397907	−4.22575123
H	−0.42992222	5.38962062	−3.80845209
H	−0.76622038	3.66902149	−4.26735519
H	2.47096912	5.27254100	−0.47978836
H	0.81027956	5.73319336	0.20718775

IV

E (TPSSh/def2-TZVP) =	−1071.02202442
$G - E$ (TPSSh/def2-TZVP) =	0.23967992
$H - E$ (TPSSh/def2-TZVP) =	0.30182826
E (DLPNO-CCSD(T)tight/def-TZVPP//TPSSh/def2-TZVP) =	−1066.34933500
E (DLPNO-CCSD(T)tight/def-QZVPP//TPSSh/def2-TZVP) =	−1066.39000155
E (DLPNO-CCSD(T)/CBS//TPSSh/def2-TZVP) =	−1069.53993112

N	1.81899544	2.37532807	−1.38893080
C	1.47701532	1.06578873	−1.16574967
N	0.73676079	0.94592779	−0.09443551
N	0.52949995	2.23656340	0.30607174
C	1.17749090	3.10949229	−0.46507062
C	−0.30413447	2.47076200	1.47276011
C	2.56501825	2.85402889	−2.55645938
Cl	−0.01835221	1.12369967	−3.58046765
C	1.17588477	4.61029423	−0.36518197
O	0.84171591	5.22738601	−1.56865748
C	−1.41917487	4.44625824	−3.90220998
N	−1.60106147	4.61368256	−2.44999440
C	−2.39973321	5.80495271	−2.15870075
C	−2.22500103	3.41101604	−1.88131343
H	−1.18195900	1.83466298	1.38467865
H	−3.24449284	3.27144730	−2.26989789
H	1.81649822	0.25232225	−1.77926953
H	−0.10086152	4.93726697	−1.86716025
H	−0.60053157	3.51616698	1.50013698
H	0.24699325	2.21560670	2.37834340
H	3.59746354	2.51293051	−2.48078046
H	2.05589064	2.42006308	−3.42379352
H	2.50891173	3.93795635	−2.57947784
H	−1.62532051	2.53688592	−2.15031740
H	−2.28794526	3.51789525	−0.79422983
H	−3.40534649	5.73615466	−2.59944788
H	−2.50213620	5.92311541	−1.07725192
H	−1.89938526	6.68734865	−2.56164696
H	−2.39032624	4.38636317	−4.41470159
H	−0.86694763	5.30336254	−4.29345690
H	−0.86337766	3.52329720	−4.08318347
H	2.18422612	4.93585522	−0.08667162
H	0.51134160	4.89339720	0.46244082

5.5.3 Thiazolium

ion pair

E (TPSSh/def2-TZVP) =	−1069.32023414
$G - E$ (TPSSh/def2-TZVP) =	0.06223355
$H - E$ (TPSSh/def2-TZVP) =	0.10314597
E (DLPNO-CCSD(T)tight/def-TZVPP//TPSSh/def2-TZVP) =	−1066.50476018
E (DLPNO-CCSD(T)tight/def-QZVPP//TPSSh/def2-TZVP) =	−1066.53895606
E (DLPNO-CCSD(T)/CBS//TPSSh/def2-TZVP) =	−1068.02617107

C	2.26123944	−0.01259223	0.68439554
S	1.51152069	−0.07379136	−0.86621426
C	−0.03064170	0.08604524	−0.19478685
N	0.03999082	0.17679896	1.12911208
C	1.32102973	0.12536939	1.64591010
C	−1.17070868	0.31935902	1.96506122
H	−1.05734388	0.06563868	−0.70242029
H	3.32919743	−0.08142853	0.81111751
H	1.46549389	0.19309212	2.71302687
H	−1.14809699	−0.45233120	2.73365351
H	−1.16256833	1.30954753	2.42035040
H	−2.03525632	0.19700489	1.29957724
Cl	−2.94991510	−0.08520351	−0.84262906

2

E (TPSSh/def2-TZVP) =	−1358.46191354
$G - E$ (TPSSh/def2-TZVP) =	0.19874719
$H - E$ (TPSSh/def2-TZVP) =	0.26552581
E (DLPNO-CCSD(T)tight/def-TZVPP//TPSSh/def2-TZVP) =	−1353.76068310
E (DLPNO-CCSD(T)tight/def-QZVPP//TPSSh/def2-TZVP) =	−1353.80715454
E (DLPNO-CCSD(T)/CBS//TPSSh/def2-TZVP) =	−1356.68515765

S	0.10389284	1.87174375	0.39518314
C	0.33530781	2.36364585	−1.26290901
N	1.67558754	2.32319538	−1.54204356
C	2.40782643	1.56329655	−0.64385990
C	1.72595703	1.23737913	0.46678691
C	2.16068243	2.69711462	−2.86439205
C	1.50858451	5.24366991	−0.28532926
O	1.83669271	5.72380252	−1.34160696
N	−1.65008098	4.58103139	−2.38037957
C	−1.73417733	5.96174040	−1.92168843
C	−2.90723057	3.87089058	−2.15806197
C	−1.26260350	4.51971903	−3.78678724
Cl	−0.62748872	0.90999866	−2.59201089
H	−3.74422321	4.32395287	−2.71681849
H	3.42969857	1.31190272	−0.88300606
H	−0.27094455	3.18947965	−1.63640849
H	3.24563781	2.61241143	−2.87372146
H	1.71566219	2.03167112	−3.60745789
H	1.88180972	3.72947997	−3.07160478
H	2.06874813	0.65866080	1.30738927
H	−2.79217586	2.83140013	−2.46814461
H	−3.15360294	3.89449044	−1.09374995
H	−2.49552565	6.54079952	−2.47286499
H	−1.99999135	5.97797773	−0.86171659
H	−0.76583779	6.44878782	−2.05134999
H	−2.00570575	5.00229744	−4.44466729
H	−0.30607343	5.03033986	−3.91975917
H	−1.15580542	3.47481526	−4.08348623
H	2.21996276	4.68434673	0.35057123
H	0.47849756	5.33774675	0.10432128

$\mathbf{TS_{2\text{-}IV}}$

E (TPSSh/def2-TZVP) =	−1358.44487442
$G - E$ (TPSSh/def2-TZVP) =	0.20525697
$H - E$ (TPSSh/def2-TZVP) =	0.26689046
E (DLPNO-CCSD(T)tight/def-TZVPP//TPSSh/def2-TZVP) =	−1353.74569017
E (DLPNO-CCSD(T)tight/def-QZVPP//TPSSh/def2-TZVP) =	−1353.79261475
E (DLPNO-CCSD(T)/CBS//TPSSh/def2-TZVP) =	−1356.67356154

N	1.70549866	2.44989364	−1.25300080
C	1.88719914	1.22525982	−0.63631065
C	1.09295389	1.06244840	0.44256894
S	0.11910073	2.47452772	0.61837880
C	0.76700842	3.26750949	−0.75105940
C	2.38806223	2.75361757	−2.51622778
Cl	−0.49929157	1.39680213	−3.35693582
C	1.57340357	5.76257287	−0.92393976
O	1.36399185	6.24014943	−2.02040781
C	−1.30412294	4.56753616	−3.84676070
N	−1.59169155	4.29610341	−2.40585023
C	−1.75563658	5.55174457	−1.62678553
C	−2.77348815	3.39675936	−2.24836996
H	−3.64087131	3.90071928	−2.67611278
H	2.59573528	0.52311072	−1.04801507
H	−0.75586640	3.78559469	−2.02526260
H	3.43327786	2.45164719	−2.44034287
H	1.86182459	2.20787112	−3.30419174
H	2.31662887	3.82332737	−2.69442140
H	1.03567552	0.20713884	1.09548999
H	−2.52737436	2.46282607	−2.75823832
H	−2.92493523	3.21850683	−1.18462792
H	−2.66326357	6.05163679	−1.96426298
H	−1.84442690	5.29369755	−0.57267245
H	−0.88412525	6.18128841	−1.79508032
H	−2.17101153	5.06777499	−4.27974525
H	−0.42659147	5.20951941	−3.89734315
H	−1.10726008	3.59806969	−4.30844971
H	2.51714827	5.24620645	−0.68003305
H	0.88365701	5.90570203	−0.07493968

IV

E (TPSSh/def2-TZVP) =	−1358.49005897
$G - E$ (TPSSh/def2-TZVP) =	0.20875980
$H - E$ (TPSSh/def2-TZVP) =	0.26828362
E (DLPNO-CCSD(T)tight/def-TZVPP//TPSSh/def2-TZVP) =	−1353.78393163
E (DLPNO-CCSD(T)tight/def-QZVPP//TPSSh/def2-TZVP) =	−1353.83084308
E (DLPNO-CCSD(T)/CBS//TPSSh/def2-TZVP) =	−1356.71959342

C	1.40889394	2.53232029	−0.62304296
N	1.26979866	1.23202891	−0.38981741
C	0.93012021	0.91554277	0.91207443
C	0.89202128	2.00629468	1.71486086
S	1.16539364	3.42868460	0.79460064
C	1.36661039	0.17423169	−1.41117667
C	1.71209277	3.17072125	−1.95741272
O	0.87284745	2.73568480	−2.97998173
C	−2.33805202	1.87877276	−3.07141754
N	−1.73144248	3.12176018	−2.56542774
C	−2.17669194	3.37314763	−1.18800660
C	−2.05780207	4.25267230	−3.43553514
Cl	−1.60386712	−0.04411328	−0.06354167
H	−3.25786219	3.57266031	−1.15206751
H	0.72714847	−0.11051759	1.16529078
H	2.24693328	−0.43149142	−1.19394557
H	0.43903743	−0.40115571	−1.31185882
H	1.43221272	0.64137013	−2.38824035
H	0.64213950	2.04802673	2.76093667
H	−1.96399679	2.49069976	−0.57882996
H	−1.65254385	4.24728335	−0.79209651
H	−3.14340159	4.42330951	−3.48667353
H	−1.58089236	5.15840695	−3.05408419
H	−1.68516556	4.05733588	−4.44271026
H	−3.42851838	1.98442833	−3.16687501
H	−1.91932044	1.65133803	−4.05408565
H	−2.12061876	1.06894254	−2.37130738
H	2.74177898	2.91737246	−2.23514382
H	1.67948292	4.25958010	−1.80550848
H	−0.11523510	2.90499404	−2.73898916

5.6 Reactions: Precursor + Amine + Formaldehyde + Bromide

ion pair with ammonium

E (TPSSh/def2-TZVP) =	−2749.26329632
$G - E$ (TPSSh/def2-TZVP) =	0.10011132
$H - E$ (TPSSh/def2-TZVP) =	0.14097300
E (DLPNO-CCSD(T)tight/def-TZVPP//TPSSh/def2-TZVP) =	−2746.36309585
E (DLPNO-CCSD(T)tight/def-QZVPP//TPSSh/def2-TZVP) =	−2746.42110890
E (DLPNO-CCSD(T)/CBS//TPSSh/def2-TZVP) =	−2748.16198575

N	0.01343136	−0.09091109	0.00948514
C	−0.03160945	0.01576014	1.48419378
C	1.38878538	0.01587415	−0.52450389
C	−0.69863890	−1.28574450	−0.49403735
H	−0.55746787	0.81500531	−0.39418985
H	−0.70197262	−1.24899032	−1.58193674
H	−0.20395836	−2.19404867	−0.14440330
H	−1.72535983	−1.24911419	−0.13434201
H	−1.07474939	0.02119288	1.79519435
H	0.49777739	−0.82390808	1.93880399
H	0.43010049	0.95812491	1.77342467
H	1.33439038	0.02073752	−1.61166365
H	1.81507666	0.95854555	−0.18607373
H	1.99408244	−0.82343287	−0.17642596
Br	−1.48575868	2.28673826	−1.05054844

5.6.1 Imidazolium

ion pair

E (TPSSh/def2-TZVP) =	−2879.67115144
$G - E$ (TPSSh/def2-TZVP) =	0.10412108
$H - E$ (TPSSh/def2-TZVP) =	0.14947528
E (DLPNO-CCSD(T)tight/def-TZVPP//TPSSh/def2-TZVP) =	−2876.00169074
E (DLPNO-CCSD(T)tight/def-QZVPP//TPSSh/def2-TZVP) =	−2876.06489512
E (DLPNO-CCSD(T)/CBS//TPSSh/def2-TZVP) =	−2878.33527539

C	1.16695732	−0.11917678	−0.51336195
N	−0.06945190	0.00535562	−0.01870731
C	0.00305355	0.19470293	1.35044740
C	1.32207268	0.18323303	1.67614575
N	2.02520112	−0.01187108	0.50322488
C	−1.28317409	−0.05207390	−0.83150953
C	3.48300719	−0.09021125	0.33774125
H	1.49759047	−0.25634408	−1.57241821
H	1.81341201	0.29639262	2.62662376
H	−0.87439573	0.31942094	1.96059561
H	3.67796471	−0.19585032	−0.73554303
H	3.93239020	0.82495998	0.72184644
H	3.85841979	−0.95424082	0.88544945
H	−1.89931471	−0.89288040	−0.51332249
H	−1.83943232	0.87888051	−0.72452990
H	−0.98412318	−0.18495250	−1.86880373
Br	2.83652489	−0.36695050	−3.23656638

2

E (TPSSh/def2-TZVP) =	−3168.82033101
$G - E$ (TPSSh/def2-TZVP) =	0.24281225
$H - E$ (TPSSh/def2-TZVP) =	0.31137571
E (DLPNO-CCSD(T)tight/def-TZVPP//TPSSh/def2-TZVP) =	−3163.26804549
E (DLPNO-CCSD(T)tight/def-QZVPP//TPSSh/def2-TZVP) =	−3163.34415579
E (DLPNO-CCSD(T)/CBS//TPSSh/def2-TZVP) =	−3167.00492047

C	2.00051776	2.37083056	−1.33217492
C	2.28761702	1.41178532	−0.41634278
N	1.08179288	0.88994895	0.01019909
C	0.08609502	1.54691130	−0.59088165
N	0.62691079	2.41450387	−1.45610285
C	0.90251767	−0.10386402	1.06762533
C	−0.13545120	3.44994605	−2.14557862
C	−0.11472393	2.38820849	3.89320042
O	−0.05357731	1.17719569	3.79482716
N	−2.93624975	1.00496376	0.00942392
C	−3.54448032	2.25389050	−0.44590272
C	−3.59722058	−0.14990541	−0.58269256
C	−2.95710385	0.93730745	1.47184661
H	1.71680187	−0.82438285	1.00786233
H	−4.66290970	−0.21437571	−0.30303753
H	2.64260399	3.01874156	−1.90148084
H	−0.98039053	1.35284322	−0.43970426
H	−0.04516402	−0.61182255	0.90416602
H	0.88376495	0.38645610	2.04278828
H	0.49217129	3.88622878	−2.92003859
H	−1.01930325	3.00407548	−2.59810543
H	−0.42793484	4.19556057	−1.40069933
H	3.22809322	1.05223894	−0.03903360
H	−3.53323126	−0.09372489	−1.67243469
H	−3.10309753	−1.06631501	−0.25056272
H	−3.98742404	0.94029859	1.86561035
H	−2.46535845	0.02159406	1.80651335
H	−2.41277619	1.79413157	1.87054905
H	−4.60536965	2.32760376	−0.15263708
H	−2.99464795	3.09188397	−0.01242819
H	−3.49085487	2.30906848	−1.53714263
H	−1.06172578	2.90819894	4.10797119
H	0.77929862	3.02817721	3.83876076
Br	−0.50162906	3.83991927	1.34320409

$\mathbf{TS_{2\text{-}IV}}$

E (TPSSh/def2-TZVP) =	−3168.78949559
$G - E$ (TPSSh/def2-TZVP) =	0.24614933
$H - E$ (TPSSh/def2-TZVP) =	0.31183542
E (DLPNO-CCSD(T)tight/def-TZVPP//TPSSh/def2-TZVP) =	−3163.23612743
E (DLPNO-CCSD(T)tight/def-QZVPP//TPSSh/def2-TZVP) =	−3163.31200054
E (DLPNO-CCSD(T)/CBS//TPSSh/def2-TZVP) =	−3166.97799768

C	1.98859937	2.11943942	−1.57344398
C	2.24218380	0.89686562	−1.04992949
N	1.19634430	0.62793936	−0.18478205
C	0.27641948	1.64079248	−0.13941426
N	0.80139787	2.54884882	−1.00934215
C	1.08262297	−0.60343953	0.58149475
C	0.17714169	3.82858907	−1.32876242
C	−0.19294044	1.87309467	2.65115808
O	−0.83226849	0.89079720	2.98313565
N	−2.94749891	1.50237542	0.56789994
C	−3.58184880	2.06611282	−0.65760624
C	−2.53562233	0.08837764	0.37369673
C	−3.83967786	1.64869916	1.75830357
H	2.05679300	−0.86989144	0.99415174
H	−3.42693825	−0.50192611	0.15912874
H	2.54537902	2.71327761	−2.27852455
H	−2.10456954	2.09750402	0.73907172
H	0.73158648	−1.42183282	−0.05252312
H	0.38224069	−0.44234790	1.39705868
H	0.92664317	4.61960577	−1.27925086
H	−0.24407329	3.79691517	−2.33668040
H	−0.60622295	4.03038770	−0.59518666
H	3.06263776	0.21681961	−1.20625675
H	−1.83084922	0.04995981	−0.45243087
H	−2.05881076	−0.25270036	1.28859351
H	−4.75173039	1.08161140	1.56907687
H	−3.30259773	1.25890613	2.62039489
H	−4.03026379	2.71318328	1.88584890
H	−4.50534385	1.52071322	−0.85215736
H	−3.75666950	3.12453149	−0.46657903
H	−2.88365550	1.94609030	−1.48446567
H	−0.66949846	2.84088743	2.41940235
H	0.91274676	1.84023123	2.61000469
Br	−2.24112630	4.59099233	1.25414304

IV

E (TPSSh/def2-TZVP) =	−3168.84588763
$G - E$ (TPSSh/def2-TZVP) =	0.25073419
$H - E$ (TPSSh/def2-TZVP) =	0.31399938
E (DLPNO-CCSD(T)tight/def-TZVPP//TPSSh/def2-TZVP) =	−3163.28701621
E (DLPNO-CCSD(T)tight/def-QZVPP//TPSSh/def2-TZVP) =	−3163.36280723
E (DLPNO-CCSD(T)/CBS//TPSSh/def2-TZVP) =	−3167.0371161

N	1.63928761	2.92431480	−0.58204958
C	2.57438092	2.16088971	−1.24923618
C	2.41666469	0.88767430	−0.82227824
N	1.38317266	0.88289047	0.09701449
C	0.87752586	2.12328039	0.18628772
C	0.75658835	−0.35038171	0.57654502
C	−0.20554653	2.65712056	1.08881933
O	−0.78053137	1.69190599	1.91204916
C	1.26070730	4.28804071	−0.93047182
C	−3.96931089	0.80246763	2.39676376
N	−3.26993678	1.03115300	1.13308790
C	−3.24888746	−0.18524067	0.31339468
C	−3.88314537	2.13579560	0.38511262
Br	−1.06325816	1.72227551	−2.08676705
H	1.48904931	−1.14934919	0.47413773
H	−4.26818204	−0.51939702	0.06556676
H	3.25450639	2.59131784	−1.96186857
H	−1.69905264	1.44409994	1.54780980
H	−0.11260441	−0.54688896	−0.05343025
H	0.45327236	−0.22995124	1.61024707
H	1.09561977	4.87180679	−0.02521628
H	2.06966685	4.73371151	−1.50494891
H	0.34679406	4.23352718	−1.52990278
H	2.93462712	−0.01645370	−1.08641061
H	−2.70016389	0.02454361	−0.60709888
H	−2.74856290	−0.98296326	0.86791853
H	−5.02276257	0.52522537	2.23678164
H	−3.47620325	−0.00220607	2.94595838
H	−3.93421608	1.71066494	3.00152428
H	−4.93691534	1.92195249	0.14838912
H	−3.84142620	3.04515656	0.98946780
H	−3.32416657	2.28334581	−0.54125278
H	−0.93711941	3.14046303	0.43198908
H	0.26284064	3.43068908	1.71718708

5.6.2 Triazolium

ion pair

E (TPSSh/def2-TZVP) =	−2895.70928287
$G - E$ (TPSSh/def2-TZVP) =	0.09392702
$H - E$ (TPSSh/def2-TZVP) =	0.13810044
E (DLPNO-CCSD(T)tight/def-TZVPP//TPSSh/def2-TZVP) =	−2891.98511694
E (DLPNO-CCSD(T)tight/def-QZVPP//TPSSh/def2-TZVP) =	−2892.04880472
E (DLPNO-CCSD(T)/CBS//TPSSh/def2-TZVP) =	−2894.3639664

N	1.20677703	−0.26446894	−0.53285353
C	−0.00631618	−0.51445838	0.02089491
N	0.01652812	0.06164953	1.23067213
N	1.12856326	0.85159070	1.38000100
C	1.82143685	0.63103306	0.30375082
C	−1.13896644	0.25589726	2.08822179
C	1.54590821	−0.56283448	−1.91848674
H	−0.67622587	−1.29639865	−0.28128603
H	2.77995957	1.07409704	0.09182938
H	0.80148970	−0.07231116	−2.55172480
H	2.54389675	−0.17916972	−2.11995274
H	1.53028379	−1.64036581	−2.07983248
H	−1.62980139	−0.70258596	2.25285634
H	−0.78434199	0.66487852	3.03033145
H	−1.82552591	0.94323711	1.58750247
Br	−1.62707851	0.92950986	−1.46349798

2

E (TPSSh/def2-TZVP) =	−3184.84596915
$G - E$ (TPSSh/def2-TZVP) =	0.22869794
$H - E$ (TPSSh/def2-TZVP) =	0.29896567
E (DLPNO-CCSD(T)tight/def-TZVPP//TPSSh/def2-TZVP) =	−3179.24856096
E (DLPNO-CCSD(T)tight/def-QZVPP//TPSSh/def2-TZVP) =	−3179.32528492
E (DLPNO-CCSD(T)/CBS//TPSSh/def2-TZVP) =	−3183.02344393

N	1.72351511	2.30509247	−1.56229304
C	2.35040239	1.51878958	−0.63340664
N	1.61210510	1.32490939	0.42020073
N	0.46204888	2.02021109	0.14686270
C	0.47684534	2.52968277	−1.08882931
C	−0.73078264	1.80337514	0.94671939
Br	−0.92076350	0.68130842	−2.56328096
C	2.13497247	2.55796608	−2.93922161
C	1.76803118	5.76503495	−0.52641897
O	1.90669021	5.71203050	−1.72129571
C	−1.37565133	4.34431198	−3.65629318
N	−1.67649919	4.50646550	−2.23360311
C	−1.73366175	5.91709672	−1.87162396
C	−2.92739476	3.82686094	−1.89610748
H	−0.41434402	1.51331792	1.94507008
H	−3.78601085	4.25249461	−2.44246330
H	3.34561291	1.12823522	−0.76362164
H	−0.25777696	3.22986750	−1.49296846
H	−1.31990147	1.01583876	0.47021549
H	−1.30838618	2.72585993	0.98196149
H	3.15972651	2.21195473	−3.05869713
H	1.45839857	1.99595291	−3.58771670
H	2.08100060	3.62552431	−3.14170135
H	−2.83184004	2.76695053	−2.13848551
H	−3.12025291	3.93361426	−0.82503257
H	−2.54574190	6.45025427	−2.39522181
H	−1.90519404	6.01366715	−0.79586640
H	−0.78763825	6.39907455	−2.12554520
H	−2.14886639	4.80314388	−4.29519994
H	−0.41880726	4.82309747	−3.87885278
H	−1.30998112	3.27843517	−3.88333649
H	2.26258419	5.04414316	0.15265585
H	1.13660809	6.53584515	−0.04592348

$\mathbf{TS_{2\text{-}IV}}$

E (TPSSh/def2-TZVP) =	−3184.82278975
$G - E$ (TPSSh/def2-TZVP) =	0.23598741
$H - E$ (TPSSh/def2-TZVP) =	0.30080451
E (DLPNO-CCSD(T)tight/def-TZVPP//TPSSh/def2-TZVP) =	−3179.21652745
E (DLPNO-CCSD(T)tight/def-QZVPP//TPSSh/def2-TZVP) =	−3179.29320521
E (DLPNO-CCSD(T)/CBS//TPSSh/def2-TZVP) =	−3182.99984738

N	1.43877415	2.29438556	−1.44134784
C	0.98665751	1.03066616	−1.16380541
N	0.30129968	1.01436307	−0.05469743
N	0.31740898	2.33425207	0.33557543
C	0.99802972	3.15586633	−0.48191585
C	−0.34814141	2.69729035	1.57051749
Br	−0.40990362	1.08126633	−4.23400273
C	2.25978532	2.65573443	−2.59773488
C	1.49372841	5.46731103	−0.76755247
O	1.08075481	5.82015265	−1.86925315
C	−1.17893790	4.54892793	−3.78507063
N	−1.39833230	4.32514980	−2.31767442
C	−1.93899191	5.54284205	−1.65129117
C	−2.25971400	3.12476150	−2.07171973
H	0.14909384	2.21933631	2.41530512
H	−3.24920935	3.33814993	−2.47450702
H	1.14758379	0.18319020	−1.80812349
H	−0.47203966	4.14577769	−1.90016789
H	−1.38750612	2.36866738	1.53988864
H	−0.29351777	3.77887635	1.67516841
H	3.28478159	2.31849677	−2.43656350
H	1.82177238	2.17809270	−3.47796006
H	2.23849824	3.73769860	−2.70505751
H	−1.80487643	2.27194237	−2.59179490
H	−2.31550712	2.96077196	−0.99698415
H	−2.90846624	5.76913332	−2.09245863
H	−2.05350151	5.33457708	−0.58834178
H	−1.22944900	6.35188021	−1.80457901
H	−2.13816773	4.82081262	−4.22481309
H	−0.45617506	5.35480200	−3.88755981
H	−0.81268779	3.60724226	−4.20870831
H	2.53679324	5.15706352	−0.60832744
H	0.93010027	5.66987947	0.15839817

IV

E (TPSSh/def2-TZVP) =	−3184.87180723
$G - E$ (TPSSh/def2-TZVP) =	0.23845637
$H - E$ (TPSSh/def2-TZVP) =	0.30176553
E (DLPNO-CCSD(T)tight/def-TZVPP//TPSSh/def2-TZVP) =	−3179.26499407
E (DLPNO-CCSD(T)tight/def-QZVPP//TPSSh/def2-TZVP) =	−3179.34155240
E (DLPNO-CCSD(T)/CBS//TPSSh/def2-TZVP) =	−3183.05257212

N	1.78624909	2.23683949	−1.41363516
C	1.40646105	0.94749431	−1.13865779
N	0.68033760	0.88899150	−0.05269856
N	0.52369491	2.19747802	0.30689228
C	1.18639674	3.02269845	−0.50339825
C	−0.28524023	2.49758732	1.47654342
Br	−0.26932310	0.94814492	−3.64459399
C	2.53133710	2.65282913	−2.60552912
C	1.25150031	4.52510300	−0.44283464
O	0.94225414	5.12729200	−1.65961427
C	−1.47811691	4.53509513	−3.88705208
N	−1.57979184	4.72749558	−2.43040910
C	−2.26698220	5.98159480	−2.11799990
C	−2.27019416	3.58412775	−1.81879959
H	0.26286288	2.23275779	2.38098037
H	−3.31087381	3.51412378	−2.16735039
H	1.70895145	0.10199482	−1.72862772
H	−0.03274849	4.92024015	−1.91977236
H	−1.19526892	1.90553052	1.41032459
H	−0.52717615	3.55713084	1.48487345
H	3.54263795	2.25104324	−2.54761872
H	1.98238243	2.23907078	−3.45877047
H	2.53891001	3.73755490	−2.64527387
H	−1.74851582	2.66266733	−2.09115297
H	−2.28312942	3.71057040	−0.73216819
H	−3.29352828	5.99057000	−2.51316679
H	−2.31089703	6.11621137	−1.03457331
H	−1.71597784	6.81732278	−2.55289869
H	−2.47329188	4.53766060	−4.35456575
H	−0.88587023	5.34733162	−4.31348007
H	−0.99535690	3.57553517	−4.08467357
H	2.27508601	4.80966157	−0.17461717
H	0.60405253	4.85856595	0.37932139

5.6.3 Thiazolium

ion pair

E (TPSSh/def2-TZVP) =	−3183.17510775
$G - E$ (TPSSh/def2-TZVP) =	0.06298018
$H - E$ (TPSSh/def2-TZVP) =	0.10459884
E (DLPNO-CCSD(T)tight/def-TZVPP//TPSSh/def2-TZVP) =	−3179.41383194
E (DLPNO-CCSD(T)tight/def-QZVPP//TPSSh/def2-TZVP) =	−3179.48365184
E (DLPNO-CCSD(T)/CBS//TPSSh/def2-TZVP) =	−3181.53981299

C	1.24125526	0.39078919	1.57197572
C	2.09612688	0.28167379	0.54041315
S	1.27448908	−0.27752408	−0.88522449
C	−0.25581101	−0.14283611	−0.08259198
N	−0.05494354	0.01952936	1.25563039
C	−1.18627150	0.21506952	2.15334425
Br	−1.38516990	1.86698155	−0.83693679
H	−1.09204503	−0.72474111	−0.43104408
H	3.15035844	0.49962709	0.52955098
H	1.46210312	0.71665401	2.57643757
H	−1.88663075	−0.61219294	2.03981710
H	−0.82025851	0.24095810	3.17787570
H	−1.68485755	1.15167363	1.88852149

2

E (TPSSh/def2-TZVP) =	−3472.31112703
$G - E$ (TPSSh/def2-TZVP) =	0.19794112
$H - E$ (TPSSh/def2-TZVP) =	0.26539163
E (DLPNO-CCSD(T)tight/def-TZVPP//TPSSh/def2-TZVP) =	−3466.67468303
E (DLPNO-CCSD(T)tight/def-QZVPP//TPSSh/def2-TZVP) =	−3466.75758414
E (DLPNO-CCSD(T)/CBS//TPSSh/def2-TZVP) =	−3470.19723775

C	0.34000216	2.42835491	−1.22777661
N	1.63292896	2.31594921	−1.60162846
C	2.40060888	1.55484744	−0.74144451
C	1.75504228	1.22224399	0.39333913
S	0.14158665	1.85709773	0.37943956
C	2.05369962	2.70172801	−2.94605724
C	1.57497026	5.29213453	−0.17171567
O	1.94061240	5.65307068	−1.26154253
N	−1.64051047	4.53037050	−2.43855050
C	−2.92119975	3.87834947	−2.16814454
C	−1.31971956	4.46070168	−3.86264923
C	−1.64274701	5.91072549	−1.97059142
Br	−0.83592683	0.75257868	−2.82953883
H	−3.75590503	4.37817758	−2.68832600
H	3.40916852	1.29838728	−1.02521294
H	−0.33016563	3.18190234	−1.65226653
H	3.11251407	2.47871485	−3.05636626
H	1.45422632	2.12370492	−3.65503989
H	1.88847554	3.76915334	−3.08246609
H	2.12461852	0.63386964	1.21580761
H	−2.86563393	2.83751036	−2.49015747
H	−3.12190553	3.90531728	−1.09405812
H	−2.39736525	6.52725525	−2.48866604
H	−1.86738312	5.93255707	−0.90096206
H	−0.65920258	6.35543232	−2.13511136
H	−2.06996061	4.98253207	−4.48060713
H	−0.34849944	4.92966563	−4.03774781
H	−1.27345450	3.41314528	−4.16504733
H	2.22294009	4.69005924	0.49325702
H	0.57192697	5.54239021	0.22080625

$TS_{2\text{-}IV}$

E (TPSSh/def2-TZVP) = −3472.29297906
$G - E$ (TPSSh/def2-TZVP) = 0.20559839
$H - E$ (TPSSh/def2-TZVP) = 0.26772353
E (DLPNO-CCSD(T)tight/def-TZVPP//TPSSh/def2-TZVP) = −3466.65054957
E (DLPNO-CCSD(T)tight/def-QZVPP//TPSSh/def2-TZVP) = −3466.73338763
E (DLPNO-CCSD(T)/CBS//TPSSh/def2-TZVP) = −3470.18086598

N	1.59574883	2.48510902	−1.32026801
C	1.32610690	1.18913598	−0.90553645
C	0.68737330	1.15477236	0.28362041
S	0.40569461	2.77391204	0.81357152
C	1.16289561	3.48518577	−0.54174806
Br	−0.50893971	0.95115392	−3.66987750
C	2.30872125	2.72244306	−2.58658664
C	1.58488736	5.64208237	−1.11682121
O	1.15532671	5.86503599	−2.25241562
C	−1.27116790	4.45982445	−3.77421372
N	−1.41243416	4.42332901	−2.28160930
C	−1.82406725	5.74686584	−1.73444025
C	−2.33768005	3.32946753	−1.84827651
H	−3.32898738	3.56077815	−2.23685935
H	1.59235693	0.36379270	−1.54607329
H	−0.47462600	4.22140524	−1.89997939
H	3.34490084	2.40055838	−2.47271727
H	1.79671877	2.14269453	−3.35924661
H	2.26300831	3.78471824	−2.81054253
H	0.35228051	0.28526447	0.82449781
H	−1.95848620	2.38902122	−2.26541748
H	−2.35103277	3.30748737	−0.75986676
H	−2.80428560	5.99183067	−2.14079691
H	−1.87907454	5.67017101	−0.64940787
H	−1.07213217	6.47513993	−2.02842692
H	−2.24070148	4.73078845	−4.19161930
H	−0.51621244	5.20546654	−4.01076018
H	−0.97189603	3.45723937	−4.09586645
H	2.64485094	5.40533036	−0.93637258
H	1.04011480	5.96952701	−0.21529359

IV

E (TPSSh/def2-TZVP) =	−3472.34008677
$G-E$ (TPSSh/def2-TZVP) =	0.20755646
$H-E$ (TPSSh/def2-TZVP) =	0.26825082
E (DLPNO-CCSD(T)tight/def-TZVPP//TPSSh/def2-TZVP) =	−3466.69994554
E (DLPNO-CCSD(T)tight/def-QZVPP//TPSSh/def2-TZVP) =	−3466.78270664
E (DLPNO-CCSD(T)/CBS//TPSSh/def2-TZVP) =	−3470.23225179

N	1.80815051	2.38886380	−1.34623309
C	1.54334074	1.07643287	−1.00220636
C	0.92844857	0.97128657	0.20063267
S	0.58175480	2.53040374	0.82471870
C	1.34123510	3.28846651	−0.48661066
Br	−0.34255665	0.86142669	−3.37644946
C	2.47108838	2.70406422	−2.62347811
C	1.44053399	4.78968723	−0.62388313
O	1.01610061	5.26153407	−1.86263302
C	−1.53498876	4.45947823	−3.85274249
N	−1.54626627	4.78831847	−2.41762800
C	−2.19809599	6.07807244	−2.18376382
C	−2.21115263	3.71912523	−1.66109823
H	−3.27377059	3.64160768	−1.93390496
H	1.82825721	0.28919987	−1.67888419
H	0.02730192	5.01913175	−2.02432202
H	3.50019610	2.34793543	−2.57254037
H	1.89585407	2.16971558	−3.38827745
H	2.42331319	3.77583534	−2.78428828
H	0.59333483	0.07554161	0.69440007
H	−1.72310689	2.76711383	−1.88411852
H	−2.14536165	3.93742809	−0.59181899
H	−3.24594215	6.06848394	−2.51833972
H	−2.17571467	6.31187595	−1.11698063
H	−1.66333802	6.86009962	−2.72569441
H	−2.55621728	4.43579631	−4.25961237
H	−0.95965173	5.21737373	−4.38841216
H	−1.07695593	3.47742242	−3.98820103
H	2.49072708	5.07637196	−0.49487328
H	0.88381509	5.22485682	0.21883631

5.7 Reactions: Precursor + Amine + Formaldehyde + Iodide

ion pair with ammonium

E (TPSSh/def2-TZVP) =	−472.81108445
$G - E$ (TPSSh/def2-TZVP) =	0.09973178
$H - E$ (TPSSh/def2-TZVP) =	0.14159598
E (DLPNO-CCSD(T)tight/def-TZVPP//TPSSh/def2-TZVP) =	−254.98992630 − 470.60946081
E (DLPNO-CCSD(T)tight/def-QZVPP//TPSSh/def2-TZVP) =	−257.95530345 − 470.61904337
E (DLPNO-CCSD(T)/CBS//TPSSh/def2-TZVP) =	598.185463868

N	0.02127694	−0.10393860	0.01506502
C	−0.02608801	0.00962992	1.49156211
C	1.39752312	0.00964553	−0.52179800
C	−0.68983087	−1.30258796	−0.48774132
H	−0.53920183	0.77704432	−0.38127017
H	−0.69221755	−1.26601535	−1.57553998
H	−0.19042609	−2.20587564	−0.13440467
H	−1.71629341	−1.26579952	−0.12765157
H	−1.06995277	0.01202310	1.79976833
H	0.50699611	−0.82698684	1.94550735
H	0.43152220	0.95550863	1.77538212
H	1.34005100	0.01262420	−1.60870082
H	1.81788906	0.95526642	−0.18454235
H	2.00306448	−0.82726191	−0.17094710
I	−1.60018339	2.44255269	−1.13171196

5.7.1 Imidazolium

ion pair

E (TPSSh/def2-TZVP) =	−603.22701325
$G - E$ (TPSSh/def2-TZVP) =	0.10482352
$H - E$ (TPSSh/def2-TZVP) =	0.15004895
E (DLPNO-CCSD(T)tight/def-TZVPP//TPSSh/def2-TZVP) =	−254.98992630 − 600.24767215
E (DLPNO-CCSD(T)tight/def-QZVPP//TPSSh/def2-TZVP) =	−257.95530345 − 600.26231311
E (DLPNO-CCSD(T)/CBS//TPSSh/def2-TZVP) =	763.005756598

C	1.17164833	−0.66867042	−0.32023383
N	−0.03711981	−0.17575283	−0.00207867
C	0.11994092	0.71870093	1.04139758
C	1.42305449	0.68235126	1.41079840
N	2.05756806	−0.23422262	0.59174518
C	−1.19169434	−0.26819287	−0.88681583
C	3.49805313	−0.39891986	0.44264864
H	1.36116481	−1.42003655	−1.06269549
H	1.95186524	1.21118371	2.18325819
H	−0.70728884	1.28536711	1.42944113
H	3.80702807	0.16176043	−0.44548560
H	3.98600939	−0.01848238	1.33734119
H	3.73520278	−1.45482631	0.31899299
H	−1.38338115	−1.31223104	−1.13117580
H	−2.05579441	0.15075269	−0.37581617
H	−0.95927575	0.29393039	−1.79710794
I	1.88972108	1.02568237	−2.68690196

2

E (TPSSh/def2-TZVP) =	−892.36315867
$G - E$ (TPSSh/def2-TZVP) =	0.24010716
$H - E$ (TPSSh/def2-TZVP) =	0.31086185
E (DLPNO-CCSD(T)tight/def-TZVPP//TPSSh/def2-TZVP) =	−254.98992630 − 887.50664788
E (DLPNO-CCSD(T)tight/def-QZVPP//TPSSh/def2-TZVP) =	−257.95530345 − 887.53441124
E (DLPNO-CCSD(T)/CBS//TPSSh/def2-TZVP) =	1128.02885757

C	2.06036190	2.35048421	−0.98354887
C	2.32264851	1.10199798	−0.52419577
N	1.13211484	0.58691622	−0.04525677
C	0.14883072	1.46884149	−0.29397500
N	0.71302516	2.58531737	−0.78436638
C	0.87371505	−0.81111415	0.27649409
C	−0.05854059	3.68554794	−1.35242677
C	−0.07362393	3.34371370	2.39611526
O	−0.85053245	4.13846899	1.93226051
N	−2.82407196	1.07268158	0.67816040
C	−3.47238873	1.96437486	−0.28411129
C	−3.10926808	−0.32368559	0.34937450
C	−3.24104603	1.38694010	2.03913027
H	1.80960926	−1.28385629	0.56732613
H	−4.18852282	−0.54587544	0.40049833
H	2.70490210	3.08881335	−1.42583138
H	−0.88863996	1.35594518	0.02575325
H	0.45125389	−1.28554844	−0.61482742
H	0.16203045	−0.86695822	1.09847286
H	0.62873026	4.48304176	−1.62636671
H	−0.58087567	3.30127868	−2.23346564
H	−0.76708072	4.05242319	−0.61261273
H	3.23880584	0.54002650	−0.49133621
H	−2.74600855	−0.53515916	−0.65832698
H	−2.59482310	−0.97662156	1.05985002
H	−4.32473610	1.24186189	2.18881988
H	−2.71450936	0.73708144	2.74376512
H	−2.99922624	2.42759798	2.26444485
H	−4.57003080	1.85859464	−0.26826582
H	−3.22492477	2.99986682	−0.03848973
H	−3.10742022	1.73093480	−1.28670001
H	−0.41387242	2.40419739	2.87092909
H	1.02005040	3.51197086	2.37119676
I	−0.84782887	0.27598994	−2.94940982

$\mathbf{TS_{2\text{-}IV}}$

E (TPSSh/def2-TZVP) =	-892.33678763
$G - E$ (TPSSh/def2-TZVP) =	0.24533792
$H - E$ (TPSSh/def2-TZVP) =	0.31181236
E (DLPNO-CCSD(T)tight/def-TZVPP//TPSSh/def2-TZVP) =	$-254.98992630 - 887.47155142$
E (DLPNO-CCSD(T)tight/def-QZVPP//TPSSh/def2-TZVP) =	$-257.95530345 - 887.49926417$
E (DLPNO-CCSD(T)/CBS//TPSSh/def2-TZVP) =	1127.9750117

C	1.92434088	2.24955677	−1.20288500
C	2.22605012	1.05709329	−0.63601928
N	1.21527064	0.79324072	0.26660510
C	0.25519282	1.76064832	0.26575235
N	0.74493474	2.66737942	−0.62521142
C	1.05855260	−0.47380162	0.95497549
C	−0.01754321	3.81777407	−1.08005915
C	−0.19536473	3.22844010	2.33350421
O	−1.24245637	3.84033461	2.23581239
N	−2.78384485	0.90051809	0.24880077
C	−3.43389026	2.07648963	−0.39868790
C	−3.42482367	−0.37396356	−0.18559010
C	−2.77590963	1.02368624	1.73096953
H	2.02763991	−0.83213417	1.30415461
H	−4.46716479	−0.35411803	0.13461398
H	2.43159234	2.81465195	−1.96542666
H	−1.79014123	0.91165814	−0.10130341
H	0.61655425	−1.20812381	0.27672206
H	0.40235963	−0.32153729	1.81039931
H	0.66557171	4.61337304	−1.37852796
H	−0.63424971	3.51762412	−1.93124574
H	−0.64608514	4.16676244	−0.26261048
H	3.04548454	0.38124850	−0.80951874
H	−3.31981227	−0.44572815	−1.26748598
H	−2.89693932	−1.20168890	0.28446100
H	−3.80225922	0.93437874	2.08764448
H	−2.17111242	0.21581070	2.13838824
H	−2.36085090	1.99148533	2.00314419
H	−4.48969227	2.08339482	−0.12822290
H	−2.94082735	2.97490981	−0.03418987
H	−3.28973467	1.96653822	−1.47353952
H	0.76298039	3.65083685	1.99033366
H	−0.12031172	2.27394774	2.88208232
I	−1.04513882	0.32829488	−2.70778358

IV

E (TPSSh/def2-TZVP) = −892.39789305
$G - E$ (TPSSh/def2-TZVP) = 0.24997004
$H - E$ (TPSSh/def2-TZVP) = 0.31397039
E (DLPNO-CCSD(T)tight/def-TZVPP//TPSSh/def2-TZVP) = −254.98992630 − 887.53486270
E (DLPNO-CCSD(T)tight/def-QZVPP//TPSSh/def2-TZVP) = −257.95530345 − 887.56233738
E (DLPNO-CCSD(T)/CBS//TPSSh/def2-TZVP) = 1128.05262543

N	1.87298945	2.58965059	−1.33654074
C	2.82742915	1.78671778	−0.74092489
C	2.34182138	1.40189412	0.46151930
N	1.09666435	1.97887230	0.59181385
C	0.79649057	2.65051794	−0.53602892
C	0.11005582	1.63250567	1.60740403
C	−0.48255505	3.43116953	−0.70098370
O	−0.50650638	4.23122187	−1.84091300
C	1.93923152	2.98485381	−2.74515337
C	−2.77205411	4.55534982	−4.26322915
N	−2.23703606	3.32201695	−3.68603174
C	−3.30438498	2.52994632	−3.06201791
C	−1.54069073	2.52465571	−4.70172432
I	−0.48557679	−0.06315778	−1.81593135
H	0.62761320	1.17520502	2.44752085
H	−4.07556412	2.24948758	−3.79554590
H	3.75464693	1.55912930	−1.23471037
H	−0.59498957	0.92426549	1.16102952
H	−0.40694013	2.53024513	1.94508334
H	2.98595043	2.95716624	−3.04272384
H	1.36108103	2.25852403	−3.32020159
H	1.52520918	3.97939863	−2.86479502
H	2.75809466	0.77106768	1.22591722
H	−2.86865755	1.62520363	−2.63327437
H	−3.77471138	3.11987588	−2.27194954
H	−3.51257037	4.34948394	−5.05114259
H	−3.24924412	5.14644061	−3.47935307
H	−1.95661334	5.13969860	−4.69409257
H	−2.21817265	2.23628645	−5.51970505
H	−0.72132159	3.11326489	−5.12111426
H	−1.14136944	1.62457249	−4.22985235
H	−0.57769869	4.06895440	0.19149969
H	−1.29514280	2.69514721	−0.68458053
H	−1.14140582	3.82197418	−2.52496966

5.7.2 Triazolium

ion pair

E (TPSSh/def2-TZVP) =	-619.26052368
$G - E$ (TPSSh/def2-TZVP) =	0.09308137
$H - E$ (TPSSh/def2-TZVP) =	0.13794602
E (DLPNO-CCSD(T)tight/def-TZVPP//TPSSh/def2-TZVP) =	$-254.98992630 - 616.23289211$
E (DLPNO-CCSD(T)tight/def-QZVPP//TPSSh/def2-TZVP) =	$-257.95530345 - 616.24816195$
E (DLPNO-CCSD(T)/CBS//TPSSh/def2-TZVP) =	783.356360219

N	1.22092472	−0.25953375	−0.52228975
C	0.01847584	−0.53438172	0.04137383
N	0.02229687	0.07286701	1.23544813
N	1.11136105	0.89288177	1.36676645
C	1.81090658	0.66912227	0.29407042
C	−1.13440203	0.24623544	2.09584963
C	1.57570839	−0.58608330	−1.89755492
H	−0.64239605	−1.32633100	−0.25487806
H	2.75632058	1.13463521	0.07184216
H	0.83666106	−0.11097704	−2.55053326
H	2.57500951	−0.20463084	−2.09606951
H	1.56235980	−1.66660379	−2.03559878
H	−1.58793883	−0.72487294	2.28984872
H	−0.78998200	0.69576135	3.02301425
H	−1.84837961	0.89381433	1.57816939
I	−1.80033889	0.98739699	−1.60103270

2

E (TPSSh/def2-TZVP) =	-908.39762941
$G - E$ (TPSSh/def2-TZVP) =	0.22775418
$H - E$ (TPSSh/def2-TZVP) =	0.29879089
E (DLPNO-CCSD(T)tight/def-TZVPP//TPSSh/def2-TZVP) =	$-254.98992630 - 903.49654420$
E (DLPNO-CCSD(T)tight/def-QZVPP//TPSSh/def2-TZVP) =	$-257.95530345 - 903.52493079$
E (DLPNO-CCSD(T)/CBS//TPSSh/def2-TZVP) =	1148.38869946

N	1.71729239	2.31616565	−1.58787597
C	2.36069403	1.58456037	−0.62779450
N	1.62788718	1.42580127	0.43678363
N	0.46648800	2.08715257	0.13746058
C	0.47038953	2.54553349	−1.11834817
C	−0.71835244	1.89335775	0.95485739
I	−1.02100930	0.42717114	−2.61688163
C	2.12905476	2.53404850	−2.97005736
C	1.88978655	5.76801436	−0.47147754
O	1.91812176	5.64125042	−1.66855909
C	−1.45640641	4.34585358	−3.67552468
N	−1.71243123	4.48269659	−2.24153213
C	−1.69840931	5.88440607	−1.84165061
C	−2.98210256	3.84863630	−1.88639423
H	−0.39159284	1.63726373	1.95902712
H	−3.83526453	4.32190529	−2.40083774
H	3.36156349	1.20434808	−0.74401562
H	−0.28287664	3.20834130	−1.55304393
H	−1.30753882	1.08664112	0.50906230
H	−1.30018710	2.81369886	0.96380681
H	3.15677197	2.19363075	−3.07857988
H	1.45998823	1.94709294	−3.60559173
H	2.06497269	3.59502396	−3.20119894
H	−2.94002364	2.79118809	−2.15292327
H	−3.14326406	3.93770978	−0.80875002
H	−2.50164358	6.46550730	−2.32605751
H	−1.83386490	5.95871575	−0.75897548
H	−0.73996342	6.33234671	−2.11012347
H	−2.22834787	4.84838064	−4.28195229
H	−0.48838166	4.79385522	−3.91310417
H	−1.43539738	3.28488464	−3.93229426
H	2.29305580	4.99324925	0.20871685
H	1.45601831	6.66459353	0.00991750

$TS_{2\text{-}IV}$

E (TPSSh/def2-TZVP) =	−908.37655601
$G - E$ (TPSSh/def2-TZVP) =	0.23360134
$H - E$ (TPSSh/def2-TZVP) =	0.29998625
E (DLPNO-CCSD(T)tight/def-TZVPP//TPSSh/def2-TZVP) =	−254.98992630 − 903.47205588
E (DLPNO-CCSD(T)tight/def-QZVPP//TPSSh/def2-TZVP) =	−257.95530345 − 903.50039767
E (DLPNO-CCSD(T)/CBS//TPSSh/def2-TZVP) =	1148.35056693

N	1.76871199	2.38666379	−1.43191023
C	2.13098217	1.29287556	−0.69919577
N	1.43612589	1.20814760	0.39756159
N	0.60126566	2.29418006	0.30775891
C	0.73384985	3.03379866	−0.80870729
C	−0.43601169	2.43609180	1.30818431
I	−1.07092735	0.79802942	−2.88060795
C	2.22569269	2.68735268	−2.78038706
C	1.72253964	5.52676660	−0.78289840
O	1.49726577	6.09756382	−1.82907459
C	−1.25426541	4.43343894	−3.73449690
N	−1.76639569	4.17431247	−2.35685378
C	−1.68913360	5.38986629	−1.50372755
C	−3.15322226	3.62610496	−2.39289966
H	−0.01243474	2.24797664	2.29352309
H	−3.80000311	4.37318158	−2.85408618
H	2.89963650	0.59630180	−0.99038013
H	−1.12807646	3.43794876	−1.95800235
H	−1.23567842	1.72017090	1.10878086
H	−0.82288639	3.45184707	1.25435538
H	3.23663097	2.30201842	−2.91104536
H	1.54460609	2.20901671	−3.48942925
H	2.22458743	3.76665092	−2.92293148
H	−3.12485046	2.69302009	−2.95459449
H	−3.47001562	3.42503892	−1.37112392
H	−2.41522282	6.11484582	−1.87170288
H	−1.93207651	5.10804892	−0.48110992
H	−0.68345050	5.79750069	−1.56387235
H	−1.91432074	5.15175080	−4.22067371
H	−0.24778779	4.83754027	−3.64881142
H	−1.24228512	3.47487446	−4.25340407
H	2.64111687	4.93738722	−0.62372097
H	1.06597014	5.62904740	0.09832453

IV

E (TPSSh/def2-TZVP) =	−908.42271269
$G - E$ (TPSSh/def2-TZVP) =	0.23712401
$H - E$ (TPSSh/def2-TZVP) =	0.30169480
E (DLPNO-CCSD(T)tight/def-TZVPP//TPSSh/def2-TZVP) =	−254.98992630 − 903.51348568
E (DLPNO-CCSD(T)tight/def-QZVPP//TPSSh/def2-TZVP) =	−257.95530345 − 903.54165978
E (DLPNO-CCSD(T)/CBS//TPSSh/def2-TZVP) =	1148.39842846

C	1.20439381	2.12915667	−1.00667950
N	1.11060218	0.82140015	−0.70817834
C	0.75924115	0.74104206	0.61569715
N	0.72492311	1.92461669	1.17028471
N	0.96856086	2.77768967	0.13387902
C	1.15046976	−0.31377825	−1.63402802
C	0.94959344	4.20688280	0.40114350
C	1.53248805	2.71546967	−2.35494199
O	0.75745831	2.18581269	−3.38181020
C	−2.55749527	1.85512209	−3.74067520
N	−1.77250672	3.02268795	−3.30717194
C	−2.23764323	3.48647530	−1.99375519
C	−1.84895586	4.09703058	−4.29865030
I	−2.24401259	−0.12850163	−0.25785705
H	1.83942635	4.48623038	0.96533900
H	−3.28054582	3.83225378	−2.03710988
H	0.58169658	−0.18753053	1.12707123
H	0.05813651	4.42247081	0.98589033
H	0.92020878	4.74825553	−0.54060820
H	1.91927494	−1.01069665	−1.30237216
H	0.15594352	−0.77303611	−1.59704804
H	1.35787079	0.06021011	−2.63135528
H	−2.17306760	2.66315367	−1.27771376
H	−1.61414869	4.32338550	−1.66593179
H	−2.88274205	4.44426868	−4.44205532
H	−1.24096388	4.94339150	−3.97051991
H	−1.46337562	3.73829903	−5.25448408
H	−3.61240708	2.12359293	−3.89474301
H	−2.14767707	1.47875499	−4.67998234
H	−2.49708437	1.08048511	−2.97357878
H	2.58337183	2.48980334	−2.56996068
H	1.45335714	3.80772753	−2.27585398
H	−0.21596523	2.50902094	−3.29135100

5.7.3 Thiazolium

ion pair

E (TPSSh/def2-TZVP) =	−906.72625212
$G - E$ (TPSSh/def2-TZVP) =	0.06205038
$H - E$ (TPSSh/def2-TZVP) =	0.10445225
E (DLPNO-CCSD(T)tight/def-TZVPP//TPSSh/def2-TZVP) =	−254.98992630 − 903.66051604
E (DLPNO-CCSD(T)tight/def-QZVPP//TPSSh/def2-TZVP) =	−257.95530345 − 903.68196821
E (DLPNO-CCSD(T)/CBS//TPSSh/def2-TZVP) =	1150.2301941

C	2.03613749	−0.29599132	0.55082066
S	1.38640940	0.37871256	−0.90852922
C	−0.14241163	0.61250302	−0.14423246
N	−0.02336022	0.43683279	1.19837839
C	1.15483989	−0.18837646	1.56244881
C	−1.19234319	0.51515297	2.06479181
H	−0.85679183	1.31071611	−0.54378283
H	3.01983582	−0.73287334	0.57652965
H	1.27808137	−0.52256917	2.58062854
H	−0.87081554	0.41133665	3.09895670
H	−1.68222098	1.47883527	1.92932159
H	−1.88203604	−0.28734412	1.78010950
I	−1.85138354	−1.34942597	−0.94928714

2

E (TPSSh/def2-TZVP) =	−1195.86279145
$G - E$ (TPSSh/def2-TZVP) =	0.19665111
$H - E$ (TPSSh/def2-TZVP) =	0.26521197
E (DLPNO-CCSD(T)tight/def-TZVPP//TPSSh/def2-TZVP) =	−254.98991745 − 1190.92219816
E (DLPNO-CCSD(T)tight/def-QZVPP//TPSSh/def2-TZVP) =	−257.95530345 − 1190.95676641
E (DLPNO-CCSD(T)/CBS//TPSSh/def2-TZVP) =	1515.25962862

S	0.34713489	1.89689038	0.54041308
C	0.48726435	2.36924986	−1.10163954
N	1.76078293	2.21856335	−1.52235775
C	2.55929408	1.51163589	−0.64646116
C	1.95606635	1.25555399	0.53198326
C	2.13131272	2.52046519	−2.90183558
C	1.86954589	5.25155709	−0.16159649
O	2.16137678	5.51504382	−1.30031092
N	−1.53134589	4.42687647	−2.30123613
C	−1.43966800	5.80481313	−1.83405024
C	−2.82321872	3.83759168	−1.95116215
C	−1.30342303	4.34666821	−3.74283192
I	−0.86566188	0.41315052	−2.70084138
H	−3.66297198	4.38142416	−2.41575825
H	3.55534783	1.23216124	−0.95166447
H	−0.20703955	3.08121962	−1.56103174
H	3.18659592	2.29487821	−3.03646399
H	1.50889479	1.89522915	−3.55131739
H	1.95339416	3.57598946	−3.09874226
H	2.35635827	0.72025130	1.37612669
H	−2.84204114	2.79698518	−2.27811078
H	−2.95350026	3.86738074	−0.86646076
H	−2.19849314	6.45587267	−2.30074114
H	−1.59126873	5.83306992	−0.75179450
H	−0.45025096	6.20542222	−2.06337802
H	−2.06514091	4.90460327	−4.31301117
H	−0.32390001	4.76958464	−3.97833507
H	−1.32427027	3.29930577	−4.04896361
H	2.49389407	4.58512368	0.46346169
H	0.96083849	5.66023120	0.31854472

$TS_{2\text{-}IV}$

E (TPSSh/def2-TZVP) =	−1195.84335824
$G-E$ (TPSSh/def2-TZVP) =	0.20498324
$H-E$ (TPSSh/def2-TZVP) =	0.26770900
E (DLPNO-CCSD(T)tight/def-TZVPP//TPSSh/def2-TZVP) =	−254.98992630 − 1190.89615347
E (DLPNO-CCSD(T)tight/def-QZVPP//TPSSh/def2-TZVP) =	−257.95530345 − 1190.93061811
E (DLPNO-CCSD(T)/CBS//TPSSh/def2-TZVP) =	1515.21832539

N	1.61145858	2.46327410	−1.33403149
C	1.43520052	1.16315843	−0.88448745
C	0.81253641	1.11546450	0.31321593
S	0.42488134	2.72226285	0.80741693
C	1.11664400	3.44700741	−0.57292656
I	−0.63150103	0.66312983	−3.70133978
C	2.28754668	2.72007612	−2.61610039
C	1.54325430	5.55543008	−1.07789058
O	1.13030112	5.82302463	−2.21511367
C	−1.26975413	4.45664196	−3.77040182
N	−1.42750990	4.48963304	−2.28003760
C	−1.85044951	5.83756295	−1.80464236
C	−2.35343664	3.41601144	−1.80332934
H	−3.34342120	3.62530722	−2.20695190
H	1.75696505	0.34075933	−1.50315428
H	−0.49010596	4.31568913	−1.88078925
H	3.33573478	2.43218928	−2.52583163
H	1.78262325	2.11910469	−3.37768564
H	2.20093814	3.77943401	−2.84280449
H	0.53941724	0.23911605	0.87727196
H	−1.98052409	2.45524472	−2.17479479
H	−2.37242610	3.44013423	−0.71515727
H	−2.82037532	6.06414730	−2.24497863
H	−1.93114846	5.81171606	−0.71881453
H	−1.08930516	6.55006868	−2.11244017
H	−2.23245668	4.71059676	−4.21303583
H	−0.50757457	5.18620137	−4.03224769
H	−0.97491587	3.44084879	−4.05007035
H	2.60342554	5.31884684	−0.89365006
H	1.00323968	5.88744920	−0.17454725

IV

E (TPSSh/def2-TZVP) = −1195.89124237
$G - E$ (TPSSh/def2-TZVP) = 0.20655852
$H - E$ (TPSSh/def2-TZVP) = 0.26821711
E (DLPNO-CCSD(T)tight/def-TZVPP//TPSSh/def2-TZVP) = −254.98992630 − 1190.94869459
E (DLPNO-CCSD(T)tight/def-QZVPP//TPSSh/def2-TZVP) = −257.95530345 − 1190.98305002
E (DLPNO-CCSD(T)/CBS//TPSSh/def2-TZVP) = 1515.2846374

N	1.88371277	2.35085680	−1.36279670
C	1.66774351	1.04158165	−0.97468550
C	1.05363065	0.95299381	0.23024995
S	0.64258485	2.51626090	0.79756108
C	1.37722910	3.26012690	−0.53506915
I	−0.47448646	0.64006826	−3.39575482
C	2.52666712	2.64895259	−2.65333924
C	1.43754116	4.75991386	−0.71203274
O	1.01094806	5.18883251	−1.96497986
C	−1.66911187	4.48612507	−3.83088360
N	−1.59364760	4.83466895	−2.40286600
C	−2.17831079	6.15443193	−2.15800981
C	−2.26276583	3.80676002	−1.59534899
H	−3.33757021	3.75846612	−1.82273254
H	1.99059882	0.24195534	−1.61944189
H	0.00798125	4.99074737	−2.09067819
H	3.55865565	2.30089882	−2.61238131
H	1.94946273	2.09902191	−3.40579469
H	2.46644301	3.71662269	−2.83483234
H	0.74640349	0.06154531	0.74933838
H	−1.81629884	2.83341201	−1.81156612
H	−2.14520282	4.04563834	−0.53507370
H	−3.24105411	6.18549654	−2.43987006
H	−2.09249969	6.40202856	−1.09762357
H	−1.63875068	6.90485155	−2.73834410
H	−2.71054843	4.48514495	−4.18316020
H	−1.10162219	5.21864922	−4.40822973
H	−1.24466574	3.49109195	−3.97802564
H	2.47996975	5.07410960	−0.58307337
H	0.86504635	5.20295148	0.11548646

5.8 Reactions: Precursor + Amine + Formaldehyde + Tetrafluoroborate

ion pair with ammonium

E (TPSSh/def2-TZVP) =	−599.80868305
$G - E$ (TPSSh/def2-TZVP) =	0.11492041
$H - E$ (TPSSh/def2-TZVP) =	0.16290738
E (DLPNO-CCSD(T)tight/def-TZVPP//TPSSh/def2-TZVP) =	−596.79880869
E (DLPNO-CCSD(T)tight/def-QZVPP//TPSSh/def2-TZVP) =	−596.82510431
E (DLPNO-CCSD(T)/CBS//TPSSh/def2-TZVP) =	−599.060737958

C	−0.26969175	0.11746493	1.46155664
N	−0.17741525	0.02295089	−0.02313500
C	−0.62289868	−1.30179001	−0.52932567
C	1.19366259	0.36646287	−0.50388718
F	−1.89553238	1.78825873	−0.84577993
H	−0.85752449	0.74361657	−0.40698440
H	−0.58350818	−1.28651878	−1.61676373
H	0.03557792	−2.07729702	−0.13832502
H	−1.64610085	−1.47463537	−0.20082206
H	−1.29320755	−0.10394128	1.75834802
H	0.41951755	−0.60042779	1.90650299
H	−0.01756692	1.13705198	1.74156999
H	1.18892621	0.36188445	−1.59074586
H	1.42924179	1.36702928	−0.15282839
H	1.89202215	−0.37337583	−0.11082562
B	−1.00138504	2.95341464	−0.95097770
F	−1.74000789	4.08118532	−1.15383220
F	−0.28949573	2.97992617	0.27193274
F	−0.11277952	2.66625126	−1.99026962

5.8.1 Imidazolium

ion pair

E (TPSSh/def2-TZVP) =	−730.22751607
$G - E$ (TPSSh/def2-TZVP) =	0.11741861
$H - E$ (TPSSh/def2-TZVP) =	0.16981329
E (DLPNO-CCSD(T)tight/def-TZVPP//TPSSh/def2-TZVP) =	−726.44557935
E (DLPNO-CCSD(T)tight/def-QZVPP//TPSSh/def2-TZVP) =	−726.47681317
E (DLPNO-CCSD(T)/CBS//TPSSh/def2-TZVP) =	−729.246245634

C	1.04433189	0.42673119	−0.63039152
N	−0.17989901	0.26392749	−0.12876445
C	−0.07044498	−0.21926123	1.16113837
C	1.25608440	−0.34509225	1.42526150
N	1.93237744	0.06705388	0.29471157
C	−1.41728720	0.49649818	−0.87818961
C	3.37647403	−0.01050609	0.05692629
H	1.29044159	0.73800883	−1.63457564
H	1.77092356	−0.69990491	2.30034081
H	−0.93482707	−0.43728767	1.76277947
H	3.54542223	0.17099036	−1.00206845
H	3.88892349	0.72809331	0.67343141
H	3.71363947	−1.01563772	0.30285945
H	−2.13754450	−0.26960538	−0.59774347
H	−1.81272857	1.48522952	−0.64448711
H	−1.18234357	0.40373807	−1.93658175
F	2.51898635	0.12847747	−3.08090627
B	1.67928226	−1.02440321	−3.22681983
F	1.99706815	−1.72654498	−4.36111416
F	1.83731309	−1.80812009	−2.05798771
F	0.33606394	−0.55368977	−3.26050690

2

E (TPSSh/def2-TZVP) =	−1019.36160104
$G - E$ (TPSSh/def2-TZVP) =	0.25148483
$H - E$ (TPSSh/def2-TZVP) =	0.33039865
E (DLPNO-CCSD(T)tight/def-TZVPP//TPSSh/def2-TZVP) =	−1013.70508555
E (DLPNO-CCSD(T)tight/def-QZVPP//TPSSh/def2-TZVP) =	−1013.74937501
E (DLPNO-CCSD(T)/CBS//TPSSh/def2-TZVP) =	−1017.90279461

N	0.44311850	2.74487693	−0.90990201
C	1.79642842	2.47861489	−0.94996521
C	1.95281850	1.19039777	−0.55285803
N	0.69580517	0.69663320	−0.27763689
C	−0.20901169	1.64784506	−0.51688772
C	0.37346661	−0.70001333	0.01779845
B	−0.78516949	0.55386044	−3.43145062
C	−0.20589153	3.96144551	−1.39506123
C	−0.07154769	1.80045302	3.10859151
O	−0.85681546	0.96540792	2.74055835
C	−3.90223486	1.99271104	−1.36592771
N	−3.30096601	1.59052784	−0.09407004
C	−3.79251076	2.41153016	1.00454838
C	−3.54745764	0.17274543	0.16418687
H	1.26393498	−1.17559265	0.42410600
H	−4.62452231	−0.04921743	0.25013866
H	2.51251082	3.21193316	−1.27470097
H	−1.29543463	1.56811802	−0.40267395
H	0.05923705	−1.17833039	−0.90825296
H	−0.42541787	−0.72548616	0.75417965
H	0.41087473	4.81932785	−1.13149804
H	−0.33732681	3.87808663	−2.47249462
H	−1.18012639	4.04520512	−0.91941353
H	2.83289591	0.57823207	−0.47224700
H	−3.13069156	−0.41405521	−0.65455545
H	−3.06340179	−0.11241704	1.10126351
H	−4.88537242	2.32871118	1.13458282
H	−3.31155193	2.10383647	1.93519087
H	−3.55488673	3.46175758	0.81361271
H	−4.99713037	1.85936788	−1.35899940
H	−3.68646160	3.04697477	−1.55377785
H	−3.46773179	1.40470045	−2.17229302
H	−0.20473249	2.34785649	4.06064338
H	0.82357292	2.06422756	2.51216084
F	−1.45650039	−0.18105940	−2.40815565
F	−1.08448111	0.03528032	−4.67399919
F	−1.20397756	1.90765073	−3.33302383
F	0.60174327	0.49569109	−3.16817408

$TS_{2\text{-}IV}$

E (TPSSh/def2-TZVP) =	−1019.33977856
$G-E$ (TPSSh/def2-TZVP) =	0.25806240
$H-E$ (TPSSh/def2-TZVP) =	0.33122474
E (DLPNO-CCSD(T)tight/def-TZVPP//TPSSh/def2-TZVP) =	−1013.66969228
E (DLPNO-CCSD(T)tight/def-QZVPP//TPSSh/def2-TZVP) =	−1013.71388073
E (DLPNO-CCSD(T)/CBS//TPSSh/def2-TZVP) =	−1017.88031481

N	0.54306543	2.84852115	−0.51075401
C	1.77149743	2.53774534	−1.05752777
C	1.93977223	1.20662820	−0.87736210
N	0.80714050	0.75631932	−0.23258301
C	−0.09294620	1.75222571	−0.01155469
C	0.56779713	−0.64179169	0.09089581
B	−1.07375383	0.59308166	−3.18555993
C	−0.08850883	4.14917237	−0.63645573
C	−0.52929418	1.77838021	2.59938580
O	−1.60693650	1.33661197	2.94277108
C	−3.83327679	1.51824509	−1.65052934
N	−3.08825596	1.43432171	−0.35972258
C	−3.46016587	2.55987065	0.53931430
C	−3.31032929	0.11856087	0.30668469
H	1.43952233	−1.05560769	0.60081247
H	−4.37675396	0.00733245	0.50380142
H	2.39799164	3.26906056	−1.53829145
H	−2.04394044	1.50656410	−0.51317803
H	0.35963548	−1.19695798	−0.82247157
H	−0.29640871	−0.69289986	0.74867559
H	0.66017814	4.93465093	−0.52895822
H	−0.57282343	4.23056276	−1.61111475
H	−0.82970061	4.25210886	0.15421742
H	2.73973030	0.55099252	−1.17380066
H	−2.95589184	−0.65307357	−0.37117753
H	−2.75135507	0.11171621	1.24012809
H	−4.52869823	2.49950706	0.74553466
H	−2.89247632	2.47039135	1.46268905
H	−3.23542878	3.49457917	0.02926714
H	−4.89897830	1.49750551	−1.41776389
H	−3.56005071	2.44329683	−2.14937091
H	−3.54771548	0.67845336	−2.27546606
H	−0.36449779	2.85551343	2.42499954
H	0.37182104	1.14604733	2.54119761
F	−1.47934547	−0.24655045	−2.09166674
F	−1.94220414	0.38200446	−4.25044115
F	−1.19784181	1.94283875	−2.74084534
F	0.24644289	0.31430838	−3.50546117

5.8.2 Triazolium

ion pair

E (TPSSh/def2-TZVP) =	−746.25913333
$G - E$ (TPSSh/def2-TZVP) =	0.10563567
$H - E$ (TPSSh/def2-TZVP) =	0.15779626
E (DLPNO-CCSD(T)tight/def-TZVPP//TPSSh/def2-TZVP) =	−742.43253191
E (DLPNO-CCSD(T)tight/def-QZVPP//TPSSh/def2-TZVP) =	−742.46448058
E (DLPNO-CCSD(T)/CBS//TPSSh/def2-TZVP) =	−745.270976445

N	1.39511258	0.93943283	−0.86861396
C	1.09680867	−0.09742109	−0.07652129
N	0.54574310	0.39975401	1.01567752
N	0.47712509	1.76050507	0.96630085
C	1.00606015	2.06048056	−0.18874900
C	0.12620576	−0.33728637	2.20382286
C	2.13508315	0.84696260	−2.13377019
H	1.32308147	−1.13277911	−0.28514336
H	1.13355178	3.06231211	−0.56276543
H	1.44691138	0.96725400	−2.97004610
H	2.89184685	1.62818531	−2.14547413
H	2.61834600	−0.12770792	−2.15437057
H	0.49487796	0.20076746	3.07377845
H	−0.96133484	−0.39731407	2.22991149
H	0.58485291	−1.32204478	2.15188960
F	3.76138475	−0.31810264	0.31835791
B	3.57874233	−1.72504658	0.27623532
F	4.75680828	−2.40002324	0.44840512
F	2.61954850	−2.06762417	1.26884452
F	2.98355313	−2.02814098	−0.99382563

2

E (TPSSh/def2-TZVP) =	−1035.40211721
$G - E$ (TPSSh/def2-TZVP) =	0.24277251
$H - E$ (TPSSh/def2-TZVP) =	0.31882654
E (DLPNO-CCSD(T)tight/def-TZVPP//TPSSh/def2-TZVP) =	−1029.70238222
E (DLPNO-CCSD(T)tight/def-QZVPP//TPSSh/def2-TZVP) =	−1029.74729809
E (DLPNO-CCSD(T)/CBS//TPSSh/def2-TZVP) =	−1033.93681823

C	0.83046085	2.67475160	−1.65064821
N	2.03110662	2.31707576	−2.12828386
C	2.66286826	1.64504893	−1.11874429
N	1.90340212	1.54321689	−0.06390391
N	0.75996483	2.18905573	−0.42207082
C	2.59451443	2.66091094	−3.43822110
C	−0.30493066	2.38803058	0.55161443
C	0.14880818	6.20965315	0.89547971
O	−0.81905384	5.50077987	1.05535519
C	−2.67779830	3.57288119	−2.97607237
N	−1.40530479	4.28355007	−3.03011109
C	−0.94389329	4.42839359	−4.40833582
C	−1.50744758	5.59563845	−2.38423189
B	2.63467870	5.44763604	−1.33456407
H	−0.59123814	1.41963284	0.95750417
H	−3.47711310	4.10243255	−3.52047747
H	3.66405683	1.25712103	−1.19670636
H	0.05634072	3.27007674	−2.16164965
H	−1.14116857	2.86250919	0.04617308
H	0.05684759	3.04724137	1.33689746
H	2.99657262	1.75563514	−3.89197605
H	1.79818991	3.06865899	−4.05232474
H	3.36329398	3.41641912	−3.29135774
H	−2.56546949	2.57825612	−3.41555445
H	−2.99181392	3.46475714	−1.93545578
H	−2.25777355	6.23272814	−2.88024448
H	−1.78747467	5.46908097	−1.33728379
H	−0.53499979	6.08420964	−2.42950119
H	−1.64965563	5.00900085	−5.02475591
H	0.01998632	4.93948287	−4.40811187
H	−0.83109276	3.44039513	−4.86447504
H	0.29445958	6.81250016	−0.01508824
H	0.93706322	6.30098012	1.66122302
F	3.85080317	4.81558566	−1.64154204
F	2.04511644	4.77839835	−0.22266427
F	2.80137533	6.78911836	−1.04329069
F	1.75175738	5.29106876	−2.44291889

$\mathbf{TS_{2\text{-}IV}}$

E (TPSSh/def2-TZVP) =	−1035.37935797
$G - E$ (TPSSh/def2-TZVP) =	0.24701040
$H - E$ (TPSSh/def2-TZVP) =	0.32020043
E (DLPNO-CCSD(T)tight/def-TZVPP//TPSSh/def2-TZVP) =	−1029.67438363
E (DLPNO-CCSD(T)tight/def-QZVPP//TPSSh/def2-TZVP) =	−1029.71926951
E (DLPNO-CCSD(T)/CBS//TPSSh/def2-TZVP) =	−1033.91538254

C	0.86911247	2.68935018	−0.96155725
N	2.10131725	2.23968519	−1.36113309
C	2.27344474	0.94683957	−0.95166476
N	1.23372569	0.51050487	−0.30100411
N	0.39617260	1.60117391	−0.32048767
C	3.10211512	3.02189884	−2.09039142
C	−0.88617505	1.48830155	0.35154072
C	0.36187199	4.82656501	0.64374478
O	−0.83972094	5.01365994	0.69460942
C	−2.00491437	3.95491873	−2.11675864
N	−1.01668283	4.91163120	−2.68191204
C	−0.88903250	4.74676073	−4.16133920
C	−1.35995895	6.32448225	−2.32350967
B	2.15661890	6.16086272	−3.44002086
H	−0.74089808	0.96017823	1.29245301
H	−2.97465986	4.15954837	−2.57032105
H	3.16211420	0.36685108	−1.14167002
H	−0.08466774	4.71557913	−2.26596231
H	−1.59141595	0.92814140	−0.26582617
H	−1.26170057	2.49100630	0.54087409
H	3.98952519	2.40227967	−2.21421167
H	2.71727178	3.32296380	−3.06125459
H	3.35080313	3.91829439	−1.52770487
H	−1.67905253	2.94449423	−2.35063706
H	−2.04328055	4.10273056	−1.04128357
H	−2.32879119	6.55678023	−2.76596486
H	−1.40407959	6.38720305	−1.23805213
H	−0.58014132	6.96680918	−2.72494718
H	−1.87248567	4.90840819	−4.60244338
H	−0.16721606	5.46964867	−4.53016761
H	−0.53997851	3.73678045	−4.36541159
H	1.01614567	5.37809813	−0.04542391
H	0.86154735	4.13531857	1.34600723
F	3.42660090	6.61501582	−3.16832309
F	1.55558905	5.67200237	−2.21671272
F	1.31454182	7.17111464	−3.93670106
F	2.17223745	5.08330488	−4.35011072

IV

E (TPSSh/def2-TZVP) =	−1035.43311364
$G - E$ (TPSSh/def2-TZVP) =	0.25065040
$H - E$ (TPSSh/def2-TZVP) =	0.32147107
E (DLPNO-CCSD(T)tight/def-TZVPP//TPSSh/def2-TZVP) =	−1029.72162769
E (DLPNO-CCSD(T)tight/def-QZVPP//TPSSh/def2-TZVP) =	−1029.76631073
E (DLPNO-CCSD(T)/CBS//TPSSh/def2-TZVP) =	−1033.97020548

N	1.43475465	2.92833685	−0.27226454
C	2.50485446	2.62371029	−0.99904858
N	2.47907845	1.28238969	−1.14394691
C	1.34611433	0.84118843	−0.52468796
N	0.69220251	1.82099112	0.02561865
C	3.57371235	3.50093857	−1.59377635
O	3.41895149	4.85421684	−1.30822969
C	3.43029294	0.47642514	−1.91281656
C	0.89385346	4.23356853	0.09576129
F	0.91035462	0.86732180	−3.57065290
B	1.20121130	2.13324283	−4.10656541
F	0.89053432	3.12257033	−3.12488228
F	2.60875247	2.20991895	−4.34231925
F	0.50681380	2.38378959	−5.26977188
N	2.50373840	6.24334060	−3.40778511
C	3.15285811	7.54483606	−3.23523347
C	1.04436170	6.37423092	−3.31871720
C	2.88501032	5.64140677	−4.69327042
H	0.47977944	4.14144903	1.09721448
H	0.65424666	7.04428378	−4.09901604
H	1.04097097	−0.19082212	−0.51150848
H	0.10884371	4.48791938	−0.61553390
H	1.69900940	4.95859228	0.05343731
H	4.41574370	0.53880684	−1.45010456
H	3.07786534	−0.55134080	−1.89755894
H	3.44641799	0.83915426	−2.93923609
H	0.59179824	5.38992920	−3.42990122
H	0.78045780	6.78972696	−2.34330894
H	2.85271771	8.25607452	−4.01919123
H	2.88269019	7.96105588	−2.26281152
H	4.23610092	7.41788013	−3.27268812
H	2.59007371	6.28425211	−5.53570692
H	3.96823281	5.50780832	−4.71969990
H	2.40499428	4.67062879	−4.79980093
H	3.56131552	3.28560130	−2.66924882
H	4.53398812	3.14551519	−1.19277534
H	3.04599178	5.33069964	−2.13385029

5.8.3 Thiazolium

ion pair

E (TPSSh/def2-TZVP) =	−1033.72001006
$G - E$ (TPSSh/def2-TZVP) =	0.07377079
$H - E$ (TPSSh/def2-TZVP) =	0.12420900
E (DLPNO-CCSD(T)tight/def-TZVPP//TPSSh/def2-TZVP) =	−1029.86136463
E (DLPNO-CCSD(T)tight/def-QZVPP//TPSSh/def2-TZVP) =	−1029.89953538
E (DLPNO-CCSD(T)/CBS//TPSSh/def2-TZVP) =	−1032.44411666

C	1.06864752	−0.20959902	1.39041766
C	2.05323208	−0.08166842	0.47244671
S	1.45500725	0.67408827	−0.95130062
C	−0.07733481	0.81501608	−0.27224440
N	−0.13071006	0.31173111	0.94953842
C	−1.38251983	0.23007518	1.72480647
F	−1.81938643	−1.41603008	−0.63093697
B	−2.36930531	−0.60205647	−1.65698438
F	−2.81460761	0.61627820	−1.02683440
F	−1.32834129	−0.24712536	−2.54389442
F	−3.40845294	−1.22672998	−2.29572917
H	−0.95509174	1.19665067	−0.78151078
H	3.07800716	−0.40370382	0.55391604
H	1.12256897	−0.66122510	2.36816721
H	−1.56028646	−0.81556974	1.96541219
H	−1.27313843	0.83136533	2.62726926
H	−2.18932607	0.58786615	1.09107619

2

E (TPSSh/def2-TZVP) =	−1322.86203101
$G - E$ (TPSSh/def2-TZVP) =	0.21056663
$H - E$ (TPSSh/def2-TZVP) =	0.28507693
E (DLPNO-CCSD(T)tight/def-TZVPP//TPSSh/def2-TZVP) =	−1317.12982140
E (DLPNO-CCSD(T)tight/def-QZVPP//TPSSh/def2-TZVP) =	−1317.18100850
E (DLPNO-CCSD(T)/CBS//TPSSh/def2-TZVP) =	−1321.10846481

N	0.80403139	1.86547416	−0.61520775
C	0.34003178	2.11209525	−1.82994571
S	1.12273258	1.18015057	−2.99187246
C	2.10721445	0.43543848	−1.80059532
C	1.81738147	0.92972818	−0.57743272
C	0.35415509	2.59169221	0.58194970
F	1.53175398	4.38124507	−3.21718147
B	2.27764127	4.62559728	−2.03993605
F	1.34127608	4.81907966	−0.97180496
F	3.03585405	3.47608631	−1.73153303
F	3.07550691	5.74215538	−2.17448072
C	−1.64753443	3.75472536	−4.20410836
N	−1.88810548	3.95576820	−2.77670574
C	−1.68080677	5.35927994	−2.41386549
C	−3.22214626	3.50598636	−2.39713604
C	0.04715788	6.31443850	0.69952624
O	−0.84729152	5.60983437	1.10410189
H	1.16932958	3.22727620	0.91838899
H	−4.01724878	4.06114614	−2.92227621
H	2.86223217	−0.28797355	−2.05828503
H	−0.45683076	2.84507850	−2.05656215
H	0.07357881	1.86600595	1.34465772
H	−0.48924080	3.21854371	0.31544862
H	2.28754493	0.69685061	0.36435136
H	−3.33420245	2.44446934	−2.63323173
H	−3.36067376	3.64485120	−1.32281447
H	−2.37200648	6.02366526	−2.95855871
H	−1.84741439	5.48143226	−1.34293883
H	−0.65376451	5.63662184	−2.64741131
H	−2.35188370	4.32930041	−4.82763298
H	−0.63001620	4.06778281	−4.44060201
H	−1.76553609	2.69518637	−4.45015453
H	−0.06659245	6.95482396	−0.19104884
H	1.02668944	6.36579970	1.20536110

$TS_{2\text{-}IV}$

E (TPSSh/def2-TZVP) = −1322.84173153
$G - E$ (TPSSh/def2-TZVP) = 0.21594162
$H - E$ (TPSSh/def2-TZVP) = 0.28654004
E (DLPNO-CCSD(T)tight/def-TZVPP//TPSSh/def2-TZVP) = −1317.10348452
E (DLPNO-CCSD(T)tight/def-QZVPP//TPSSh/def2-TZVP) = −1317.15462139
E (DLPNO-CCSD(T)/CBS//TPSSh/def2-TZVP) = −1321.08848473

C	0.62279752	2.66731952	−0.79229180
S	2.02172000	2.31746955	−1.70684936
C	2.29832204	0.78716697	−0.95428131
C	1.34214088	0.56789813	−0.02709732
N	0.43698425	1.62019786	0.03831244
C	−0.67955587	1.59554536	0.98745438
C	0.22861453	4.98364215	0.50963286
O	−0.96190154	5.18860260	0.66434604
B	1.83159066	5.33543400	−3.81669379
N	−1.33579228	4.48341834	−2.66976832
C	−1.32248609	4.03394613	−4.09481988
C	−2.30127940	3.69324427	−1.86112538
C	−1.60653718	5.95151630	−2.56430525
H	−0.30840844	1.35731309	1.98516186
H	−3.30074606	3.86316790	−2.26199629
H	3.12120593	0.13775961	−1.20789686
H	−0.37912635	4.33209236	−2.28901864
H	−1.41201908	0.84372628	0.68674445
H	−1.13668464	2.58130624	0.99343680
H	1.22440851	−0.28455836	0.62609495
H	−2.03379063	2.64207807	−1.93675473
H	−2.23565344	4.02892315	−0.82958090
H	−2.59006161	6.14553670	−2.99267813
H	−1.58660877	6.21608720	−1.50868422
H	−0.82724425	6.46969709	−3.11802991
H	−2.33314965	4.13906478	−4.48955918
H	−0.61599933	4.65014067	−4.64379218
H	−1.00496600	2.99385656	−4.12253277
H	0.80350302	5.41311378	−0.32214696
H	0.81203358	4.41210294	1.25445463
F	3.11158801	5.82525210	−3.72759852
F	0.95392290	6.25377501	−4.43450600
F	1.77104327	4.10908401	−4.49212559
F	1.31068852	5.10680064	−2.47872411

5.9 Reactions: Precursor + Amine + Formaldehyde + Triflate

ion pair with ammonium

E (TPSSh/def2-TZVP) =	−1136.95998270
$G - E$ (TPSSh/def2-TZVP) =	0.12365940
$H - E$ (TPSSh/def2-TZVP) =	0.17791233
E (DLPNO-CCSD(T)tight/def-TZVPP//TPSSh/def2-TZVP) =	−1132.48131249
E (DLPNO-CCSD(T)tight/def-QZVPP//TPSSh/def2-TZVP) =	−1132.52840537
E (DLPNO-CCSD(T)/CBS//TPSSh/def2-TZVP) =	−1135.57243923

C	0.21517665	0.17722058	1.31418818
N	−0.17975382	0.09572152	−0.11843464
C	0.90157298	0.60078801	−1.00776949
C	−0.62106529	−1.26891953	−0.49897321
O	−2.26458194	1.62684351	−0.38710013
S	−1.86024067	3.02977100	−0.09718191
O	−0.48723697	3.09849198	0.38848348
C	−1.74628102	3.74187356	−1.80771560
O	−2.85649851	3.81494473	0.57530199
H	−1.03947612	0.75192952	−0.24422458
H	−0.94942880	−1.25129683	−1.53666185
H	0.20686312	−1.96834378	−0.37816317
H	−1.45285128	−1.55667474	0.14148674
H	−0.62576521	−0.15235411	1.92194736
H	1.08058423	−0.46427998	1.48540390
H	0.44111053	1.21775502	1.53498466
H	0.54205041	0.58024132	−2.03464993
H	1.11753538	1.62589276	−0.71714855
H	1.77998974	−0.03695757	−0.89976206
F	−1.32298786	5.01021913	−1.77621154
F	−0.86332065	3.03177714	−2.55117657
F	−2.92674889	3.70434378	−2.43629908

5.9.1 Imidazolium

ion pair

E (TPSSh/def2-TZVP) =	−1267.37342391
$G - E$ (TPSSh/def2-TZVP) =	0.12614246
$H - E$ (TPSSh/def2-TZVP) =	0.18544956
E (DLPNO-CCSD(T)tight/def-TZVPP//TPSSh/def2-TZVP) =	−1262.12340980
E (DLPNO-CCSD(T)tight/def-QZVPP//TPSSh/def2-TZVP) =	−1262.17551676
E (DLPNO-CCSD(T)/CBS//TPSSh/def2-TZVP) =	−1265.75258969

N	2.06119455	−0.25648588	0.12884224
C	1.20455447	0.39508026	−0.65887427
N	0.01214613	0.40508802	−0.06442391
C	0.10005709	−0.28566651	1.12793937
C	1.38604925	−0.70145416	1.25196626
C	−1.20981611	0.90635218	−0.69772904
C	3.44676006	−0.55990696	−0.22350241
O	0.67126668	0.86734348	−3.44368962
S	0.43517752	−0.56070923	−3.70274324
O	−0.53682966	−0.86305144	−4.72165490
C	2.06177480	−1.09709385	−4.42107245
O	0.36068756	−1.35402313	−2.47399083
H	1.39399538	0.80788855	−1.64563002
H	1.87664092	−1.26298387	2.02704993
H	−0.74978424	−0.41928846	1.77348876
H	3.53732098	−1.62263671	−0.44558412
H	3.70719376	0.01120811	−1.11021231
H	4.09985974	−0.28660879	0.60444403
H	−1.85869369	0.06057702	−0.92045867
H	−1.70319184	1.60925312	−0.02736560
H	−0.92718068	1.38797792	−1.63105231
F	2.08340904	−2.41199338	−4.67881126
F	2.35259594	−0.44435133	−5.55277843
F	3.07064234	−0.84538796	−3.54044020

2

E (TPSSh/def2-TZVP) =	−1556.51248719
$G - E$ (TPSSh/def2-TZVP) =	0.26091711
$H - E$ (TPSSh/def2-TZVP) =	0.34610494
E (DLPNO-CCSD(T)tight/def-TZVPP//TPSSh/def2-TZVP) =	−1549.38374158
E (DLPNO-CCSD(T)tight/def-QZVPP//TPSSh/def2-TZVP) =	−1549.44881913
E (DLPNO-CCSD(T)/CBS//TPSSh/def2-TZVP) =	−1554.41325897

N	0.42138711	2.49572027	−1.08068851
C	1.75624132	2.28890189	−1.34799643
C	2.08293627	1.07568677	−0.83331226
N	0.93728131	0.56484655	−0.26066279
C	−0.06570027	1.43169814	−0.43591075
C	0.77402427	−0.78788639	0.27267488
C	−0.39407623	3.60369384	−1.58330247
C	0.21929102	3.56392513	2.43132457
O	−0.76932626	3.86234535	1.81240265
C	−3.08143437	0.98391469	2.09293528
N	−2.93791821	0.94006546	0.64366808
C	−3.37158677	−0.35157726	0.10952402
C	−3.67959286	2.03057972	0.01143628
O	−0.99322205	−1.04622145	−2.24641138
H	1.73351763	−1.12619252	0.65906707
H	−4.43654821	−0.54300258	0.32334610
H	2.33934422	2.99864141	−1.90623482
H	−1.09660195	1.28367341	−0.09085833
H	0.41531035	−1.42910107	−0.53285133
H	0.04097719	−0.76108615	1.07633322
H	0.27675915	4.35467837	−1.99424304
H	−1.05385498	3.20811944	−2.35544399
H	−0.96404795	4.02498285	−0.75839922
H	3.00529078	0.52384278	−0.85211233
H	−3.20653230	−0.37140675	−0.96607538
H	−2.78057632	−1.15059125	0.56278639
H	−4.12721540	0.84127960	2.41516002
H	−2.47847050	0.19196823	2.54598912
H	−2.73595019	1.94987078	2.46667130
H	−4.76124854	1.96577396	0.21714990
H	−3.31450005	2.98610848	0.39593848
H	−3.52031383	1.98650212	−1.06618060
H	1.14371636	3.22151487	1.92670766
H	0.25382829	3.61462023	3.53583935
S	−1.00324981	0.10903119	−3.13790932
C	−1.67406821	−0.57648987	−4.72492931
O	−1.98145752	1.13669209	−2.78136697
O	0.30967998	0.62090096	−3.49991299
F	−1.74574886	0.37672506	−5.66971333
F	−0.89294523	−1.56343264	−5.19429914
F	−2.90986937	−1.07670073	−4.55117669

$\mathbf{TS_{2\text{-}IV}}$

E (TPSSh/def2-TZVP) = −1556.49138536
$G - E$ (TPSSh/def2-TZVP) = 0.26783803
$H - E$ (TPSSh/def2-TZVP) = 0.34698406
E (DLPNO-CCSD(T)tight/def-TZVPP//TPSSh/def2-TZVP) = −1549.34963290
E (DLPNO-CCSD(T)tight/def-QZVPP//TPSSh/def2-TZVP) = −1549.41452828
E (DLPNO-CCSD(T)/CBS//TPSSh/def2-TZVP) = −1554.39214078

N	0.55797675	2.61031417	−0.99227300
C	1.82958313	2.30264147	−1.42546510
C	2.17046184	1.14400029	−0.81207609
N	1.09229228	0.79156235	−0.02764465
C	0.06190989	1.67736230	−0.13058545
C	0.99044912	−0.46661968	0.69328524
C	−0.22400055	3.72793581	−1.49974827
C	−0.11692750	3.10392871	2.13432375
O	−1.24723138	3.43530414	2.42455818
C	−2.72870734	0.55106178	1.61187700
N	−2.86669661	0.93139298	0.18142045
C	−3.74120445	−0.03162223	−0.54681787
C	−3.36577502	2.32698528	0.03224901
O	−1.12742500	−0.80548060	−1.68880218
H	1.97980723	−0.77168777	1.03440861
H	−4.71907434	−0.02353958	−0.06326241
H	2.36061728	2.90206546	−2.14406166
H	−1.89094950	0.94288463	−0.21701442
H	0.56292416	−1.22537975	0.03674099
H	0.34197117	−0.32198769	1.55608830
H	0.45045693	4.52688054	−1.80694436
H	−0.82587098	3.39311291	−2.34504398
H	−0.87434772	4.09135185	−0.70587913
H	3.05733680	0.53947423	−0.89023056
H	−3.80635107	0.28803770	−1.58179768
H	−3.28448878	−1.01535464	−0.50299039
H	−3.71756854	0.55280998	2.07008068
H	−2.29796726	−0.44731986	1.65782067
H	−2.08712419	1.27827808	2.10227192
H	−4.37934564	2.37881091	0.42994739
H	−2.70590025	2.98525634	0.59216600
H	−3.34881183	2.56346642	−1.02878792
H	0.54672185	3.74594219	1.53156293
H	0.34376236	2.18098820	2.52761540
S	−1.09549686	0.11203871	−2.82916659
C	−1.95463566	−0.85893298	−4.15723282
O	−1.97702056	1.27232031	−2.66370075
O	0.21035006	0.39318533	−3.38137714
F	−2.05345151	−0.15328943	−5.29367717
F	−1.30333709	−1.99905778	−4.43053387
F	−3.20900323	−1.18881604	−3.77248908

IV

E (TPSSh/def2-TZVP) =	−1556.54944643
$G - E$ (TPSSh/def2-TZVP) =	0.27189158
$H - E$ (TPSSh/def2-TZVP) =	0.34928794
E (DLPNO-CCSD(T)tight/def-TZVPP//TPSSh/def2-TZVP) =	−1549.40985191
E (DLPNO-CCSD(T)tight/def-QZVPP//TPSSh/def2-TZVP) =	−1549.47459239
E (DLPNO-CCSD(T)/CBS//TPSSh/def2-TZVP) =	−1554.45344563

N	0.81043014	2.84093047	−0.73646972
C	1.82049244	2.82671692	−1.68015397
C	2.46297320	1.64219645	−1.56665147
N	1.83845415	0.94715445	−0.55420164
C	0.81942496	1.67890059	−0.06828083
C	2.14299084	−0.42681751	−0.15490043
C	−0.21465346	3.88810020	−0.69546076
C	−0.08989575	1.17728956	1.02094486
O	−1.01638076	2.12076323	1.46542873
C	−4.37525162	1.93025900	1.70871659
N	−3.48957732	1.42350506	0.65908691
C	−3.59814187	−0.03774268	0.55173419
C	−3.79594045	2.05514337	−0.62998000
O	−0.82319087	−0.92281711	−1.29547546
H	2.98831874	−0.76510626	−0.74766068
H	−4.62097980	−0.34268715	0.28436173
H	1.96311929	3.64750383	−2.35921395
H	−1.93900065	1.84855957	1.13060678
H	1.27541807	−1.05272597	−0.36571200
H	2.39925649	−0.45482791	0.90491672
H	0.24563913	4.81033585	−1.04613466
H	−1.02540362	3.59204910	−1.36023300
H	−0.57682139	3.99449483	0.32022472
H	3.27010937	1.21414573	−2.13192850
H	−2.90588634	−0.40413922	−0.20588975
H	−3.34306479	−0.48659482	1.51419998
H	−5.43112794	1.70488027	1.49506502
H	−4.10582510	1.47476274	2.66326537
H	−4.25927258	3.01261806	1.79209383
H	−4.82454233	1.83188807	−0.95047027
H	−3.69332964	3.13827277	−0.53002502
H	−3.09682220	1.69656348	−1.38485749
H	0.55455141	0.86910326	1.85720709
H	−0.56578367	0.27394536	0.61903712
S	−0.61222460	−0.14352831	−2.51741369
C	−1.83886157	−0.87700457	−3.69904802
O	−1.04722138	1.24707629	−2.41315021
O	0.67444684	−0.35032452	−3.15611856
F	−1.79606069	−0.25835550	−4.88939738
F	−1.60451065	−2.18220372	−3.90065330
F	−3.09357304	−0.75428822	−3.21874791

5.9.2 Triazolium

ion pair

E (TPSSh/def2-TZVP) =	−1283.40575166
$G - E$ (TPSSh/def2-TZVP) =	0.11483824
$H - E$ (TPSSh/def2-TZVP) =	0.17335621
E (DLPNO-CCSD(T)tight/def-TZVPP//TPSSh/def2-TZVP) =	−1278.11114324
E (DLPNO-CCSD(T)tight/def-QZVPP//TPSSh/def2-TZVP) =	−1278.16390570
E (DLPNO-CCSD(T)/CBS//TPSSh/def2-TZVP) =	−1281.77781222

C	0.46624343	2.05209316	−0.32786468
N	1.11120428	1.03450182	−0.97466570
C	1.41760929	0.12557800	−0.03857291
N	0.94150090	0.58954531	1.10457365
N	0.34084528	1.80627639	0.94740955
C	1.57105655	0.99136573	−2.36785005
C	1.05918349	−0.01378353	2.42581157
O	3.99275897	0.61995747	−0.49479248
S	4.46767993	−0.71944702	−0.85470279
O	5.57898550	−0.78481932	−1.76661425
C	5.12898651	−1.34868754	0.76201504
O	3.36001139	−1.65188112	−1.11910939
H	1.97321736	−0.79525218	−0.23202838
H	0.10607455	2.94072727	−0.81849709
H	0.77594960	1.36258621	−3.01205402
H	2.47227108	1.59538139	−2.45360945
H	1.81458566	−0.04025307	−2.60853691
H	1.63403890	0.65499991	3.06380717
H	0.06275102	−0.16425841	2.83769163
H	1.58164000	−0.96039547	2.31582943
F	5.57506316	−2.60618722	0.66555841
F	6.12471799	−0.58265588	1.22360987
F	4.14411619	−1.34021289	1.70313777

2

E (TPSSh/def2-TZVP) =	−1572.54412348
$G-E$ (TPSSh/def2-TZVP) =	0.25210931
$H-E$ (TPSSh/def2-TZVP) =	0.33441404
E (DLPNO-CCSD(T)tight/def-TZVPP//TPSSh/def2-TZVP) =	−1565.37395300
E (DLPNO-CCSD(T)tight/def-QZVPP//TPSSh/def2-TZVP) =	−1565.43974926
E (DLPNO-CCSD(T)/CBS//TPSSh/def2-TZVP) =	−1570.43878915

N	1.41504061	1.36562496	0.33794772
N	0.43329796	2.22705089	−0.05158695
C	0.64826154	2.71056386	−1.26636060
N	1.78342775	2.13929620	−1.69780911
C	2.22504352	1.33551257	−0.68410950
C	−0.68256561	2.51107760	0.84305394
C	2.44172882	2.36547233	−2.98876330
N	−1.32257632	4.47626098	−2.73277285
C	−1.47913466	5.77356557	−2.06766953
C	−0.82468185	4.65593937	−4.09793516
C	−2.57606384	3.73183487	−2.72938508
O	2.11398200	5.00066576	−1.87700710
S	2.55338307	6.30600208	−2.39555776
O	1.50979825	7.04880372	−3.07881889
O	3.40395891	7.04862358	−1.49112774
C	3.69098631	5.81207096	−3.78062539
F	4.69619353	5.02432207	−3.33692208
F	3.02523329	5.09913902	−4.72314207
F	4.23648779	6.87039456	−4.38481280
C	1.32939827	5.94518977	0.64617849
O	0.49962052	5.18064506	1.08567349
H	−1.24522908	1.59188792	1.00120898
H	−3.37105998	4.24859384	−3.29133278
H	3.13151280	0.75577468	−0.73275043
H	0.00412649	3.42908375	−1.81243505
H	−1.29845081	3.27379652	0.37665467
H	−0.28392865	2.89434447	1.77760453
H	2.54893135	1.40853253	−3.49936513
H	1.82896735	3.04695843	−3.56722466
H	3.40817146	2.83154618	−2.81712501
H	−2.42466024	2.74721673	−3.18037612
H	−2.92337136	3.59730168	−1.70161558
H	−2.27882922	6.37197641	−2.53256717
H	−1.72652452	5.61821396	−1.01502392
H	−0.54244900	6.32357329	−2.14098088
H	−1.54715013	5.19586453	−4.73050736
H	0.10396104	5.22526744	−4.06803191
H	−0.64545832	3.67640744	−4.55162618
H	1.04759536	6.84863224	0.08056249
H	2.40979261	5.79486417	0.79679279

$\mathbf{TS_{2\text{-}IV}}$

E (TPSSh/def2-TZVP) =	−1572.52753789
$G - E$ (TPSSh/def2-TZVP) =	0.25528952
$H - E$ (TPSSh/def2-TZVP) =	0.33562411
E (DLPNO-CCSD(T)tight/def-TZVPP//TPSSh/def2-TZVP) =	−1565.35512915
E (DLPNO-CCSD(T)tight/def-QZVPP//TPSSh/def2-TZVP) =	−1565.42080345
E (DLPNO-CCSD(T)/CBS//TPSSh/def2-TZVP) =	−1570.42466875

N	0.29564687	1.76406261	−0.06020116
C	0.71137473	2.64665090	−0.99265947
N	1.80778651	1.99262966	−1.49303814
C	1.96911824	0.79621333	−0.84963579
N	1.04764225	0.61684340	0.05107158
C	2.66284747	2.50105236	−2.56240240
C	−0.85118896	1.91115272	0.81865647
C	0.67340513	5.08503633	0.64289791
O	−0.50739911	5.36409164	0.69705568
O	1.82965157	5.57191183	−2.30027788
S	2.38010889	6.72946659	−3.03299316
O	3.19237102	7.61695516	−2.24069544
C	3.57203854	5.90818794	−4.19703471
F	2.92972940	5.00389701	−4.97599355
O	1.41071335	7.33561103	−3.93727451
F	4.16520898	6.79210498	−5.00440882
F	4.53196201	5.24207603	−3.52831444
N	−0.91505432	5.04177645	−2.55452651
C	−0.67537396	4.77846059	−4.00396361
C	−1.98829646	4.17146973	−2.00700667
C	−1.21774519	6.48684670	−2.30804018
H	−0.56435087	1.60732299	1.82430624
H	−2.91224712	4.39114859	−2.54168608
H	2.76415004	0.09790674	−1.05648177
H	−0.02381887	4.81118349	−2.07250545
H	−1.67242000	1.27883494	0.47588265
H	−1.15086847	2.95601892	0.81763735
H	3.70691356	2.30912511	−2.31632916
H	2.41769923	2.01167931	−3.50684084
H	2.49624712	3.57153604	−2.63732133
H	−1.69393947	3.13445689	−2.14370308
H	−2.09608318	4.39302260	−0.94845767
H	−2.17849881	6.71022013	−2.77221390
H	−1.26080432	6.63469152	−1.23133943
H	−0.42504503	7.07898250	−2.75981853
H	−1.60769740	4.94891966	−4.54189960
H	0.10034072	5.46027248	−4.34532975
H	−0.35764888	3.74297660	−4.11194243
H	1.35075557	5.52558289	−0.10355894
H	1.12436223	4.38233459	1.36869953

IV

E (TPSSh/def2-TZVP) =	−1572.58364359
$G - E$ (TPSSh/def2-TZVP) =	0.26022112
$H - E$ (TPSSh/def2-TZVP) =	0.33721675
E (DLPNO-CCSD(T)tight/def-TZVPP//TPSSh/def2-TZVP) =	−1565.40025921
E (DLPNO-CCSD(T)tight/def-QZVPP//TPSSh/def2-TZVP) =	−1565.46570739
E (DLPNO-CCSD(T)/CBS//TPSSh/def2-TZVP) =	−1570.48034769

N	0.95049941	1.80774189	−0.91337446
C	1.37926106	2.95994174	−0.41018098
N	2.72133320	2.84110170	−0.31395614
C	3.04462925	1.61259660	−0.81559922
N	1.98368287	0.95478458	−1.17822097
C	3.65043401	3.88101110	0.13402271
C	−0.38166493	1.43177519	−1.37866382
C	0.63031715	4.20256175	−0.01231244
O	−0.75247404	4.08661343	−0.11712922
O	1.39560610	3.48444309	−3.31294436
S	2.60957668	4.29237222	−3.23044630
O	3.84330824	3.54461911	−3.05939021
C	2.74375105	5.03648861	−4.92363975
F	1.64394103	5.76311429	−5.20775678
O	2.47827639	5.46259043	−2.35923672
F	3.80682587	5.84796467	−5.01623087
F	2.86031923	4.08611451	−5.86230982
N	−1.64906388	5.53459733	−2.18238395
C	−1.72623244	4.68976415	−3.38115794
C	−2.98310271	5.97784177	−1.77193788
C	−0.77198786	6.68869359	−2.42468406
H	−0.49513280	0.36553130	−1.19842566
H	−3.46655079	6.58649281	−2.55061471
H	4.05601868	1.25705846	−0.90710216
H	−1.06635080	4.63199464	−0.92477249
H	−0.43242389	1.64658817	−2.44550422
H	−1.11200010	2.01760774	−0.83176781
H	3.49635775	4.07556688	1.19585962
H	4.65934979	3.51666975	−0.03838262
H	3.48605034	4.77629239	−0.46538668
H	−3.60980712	5.10729905	−1.56934720
H	−2.90416340	6.57260956	−0.86028206
H	−1.16446457	7.32465455	−3.23176373
H	−0.70628980	7.28580563	−1.51269399
H	0.22488308	6.34264964	−2.69503367
H	−2.15510908	5.24098804	−4.23088756
H	−0.72663959	4.34338415	−3.64153699
H	−2.36375757	3.82808791	−3.17032617
H	1.04347233	5.00323816	−0.63998910
H	0.91119989	4.41922243	1.02877338

5.9.3 Thiazolium

ion pair

E (TPSSh/def2-TZVP) =	−1570.86869794
$G - E$ (TPSSh/def2-TZVP) =	0.08415985
$H - E$ (TPSSh/def2-TZVP) =	0.13979096
E (DLPNO-CCSD(T)tight/def-TZVPP//TPSSh/def2-TZVP) =	−1565.54075734
E (DLPNO-CCSD(T)tight/def-QZVPP//TPSSh/def2-TZVP) =	−1565.59970907
E (DLPNO-CCSD(T)/CBS//TPSSh/def2-TZVP) =	−1568.95261036

C	1.04922494	−0.39895904	1.11323639
C	1.69148051	−0.56594117	−0.06416328
S	0.84003746	0.23239405	−1.33018062
C	−0.34245703	0.75964170	−0.25338034
N	−0.09345312	0.36473268	0.98522882
C	−1.03637439	0.60964040	2.09033141
O	−3.09026384	1.58830249	−0.28710576
S	−3.55417624	0.21190569	−0.53033449
O	−4.90626168	−0.07875561	−0.13263492
C	−3.56255315	0.13110362	−2.38592570
O	−2.53824404	−0.78557753	−0.18232107
H	−1.23851331	1.33517522	−0.50226974
H	2.60160273	−1.11158403	−0.24947496
H	1.32333894	−0.78533029	2.08214452
H	−1.50573919	−0.33711124	2.35102370
H	−0.48584412	1.02668618	2.93272088
H	−1.79886505	1.29656167	1.73239150
F	−3.88663712	−1.09038201	−2.82827009
F	−4.41468613	1.01217890	−2.92211964
F	−2.32321515	0.42045033	−2.86882259

2

E (TPSSh/def2-TZVP) =	−1860.00842916
$G - E$ (TPSSh/def2-TZVP) =	0.21919726
$H - E$ (TPSSh/def2-TZVP) =	0.30073122
E (DLPNO-CCSD(T)tight/def-TZVPP//TPSSh/def2-TZVP) =	−1852.80458107
E (DLPNO-CCSD(T)tight/def-QZVPP//TPSSh/def2-TZVP) =	−1852.87666017
E (DLPNO-CCSD(T)/CBS//TPSSh/def2-TZVP) =	−1857.61459552

S	−0.13369950	1.91225322	0.32626427
C	0.30369999	2.63017553	−1.13035044
N	1.29004341	1.97668244	−1.72097520
C	1.71709603	0.86062104	−1.03208099
C	1.05273749	0.68841413	0.13179478
C	1.85455879	2.36080702	−3.02450896
C	1.73488151	5.85712317	−0.58402002
O	2.24480457	5.09222598	−1.36160904
N	−1.60064849	4.74471210	−2.29833897
C	−1.80446265	6.05254493	−1.68950659
C	−2.76445021	3.87925825	−2.08264716
C	−1.31856311	4.87298790	−3.72944002
O	0.41978578	−0.38969777	−3.43639227
S	−0.89086770	0.22375150	−3.24734537
O	−1.44850447	0.09233022	−1.90914607
O	−1.01410546	1.56170162	−3.82490115
C	−2.01637385	−0.80505202	−4.30213964
F	−1.63754206	−0.77206893	−5.59071710
F	−2.01505520	−2.08754574	−3.90414086
F	−3.28130761	−0.35316891	−4.23392415
H	−3.67564414	4.30022355	−2.53699142
H	2.47006094	0.22758678	−1.47073725
H	−0.21126139	3.49932625	−1.56402456
H	2.89253417	2.65484847	−2.87535735
H	1.75609727	1.50148840	−3.68500903
H	1.27685203	3.19295763	−3.40956479
H	1.16432638	−0.11032796	0.84494917
H	−2.56864061	2.89943297	−2.51848948
H	−2.93941965	3.76728807	−1.00930814
H	−2.67768042	6.57933547	−2.10973284
H	−1.96205718	5.93973658	−0.61365010
H	−0.92335946	6.67802578	−1.85556912
H	−2.14056618	5.37510204	−4.26547331
H	−0.40893222	5.46359402	−3.86771816
H	−1.17810976	3.87917839	−4.15420502
H	0.95229573	5.53219872	0.12791966
H	2.02196325	6.92448216	−0.54197631

$TS_{2\text{-}IV}$

E (TPSSh/def2-TZVP) =	−1859.99763004
$G - E$ (TPSSh/def2-TZVP) =	0.22550750
$H - E$ (TPSSh/def2-TZVP) =	0.30208212
E (DLPNO-CCSD(T)tight/def-TZVPP//TPSSh/def2-TZVP) =	−1852.78442297
E (DLPNO-CCSD(T)tight/def-QZVPP//TPSSh/def2-TZVP) =	−1852.85624037
E (DLPNO-CCSD(T)/CBS//TPSSh/def2-TZVP) =	−1857.60387445

C	0.64873162	3.09895256	−1.04200863
N	1.48505269	2.14289197	−1.48306994
C	1.67138551	1.02724069	−0.68203193
C	0.97918637	1.10155858	0.47345804
S	0.09949730	2.58225997	0.49132070
C	2.17646487	2.24921493	−2.77459832
C	1.76952658	5.58050032	−1.23059629
O	1.15056252	6.36297489	−1.91914711
N	−1.71939815	4.29666682	−2.47513582
C	−2.96889406	3.50541915	−2.67590123
C	−1.26268006	4.92392075	−3.75030989
C	−1.89678836	5.30582893	−1.39437139
O	−0.69590299	1.87097771	−3.93937639
H	−3.77931115	4.20878837	−2.87053473
H	2.28374815	0.21432627	−1.03892537
H	−0.93977666	3.66682130	−2.15902960
H	3.24633077	2.38039171	−2.59907515
H	1.98560559	1.34458809	−3.34846835
H	1.76834160	3.10582882	−3.30077439
H	0.92873089	0.36465634	1.25789469
H	−2.81477280	2.83524000	−3.51419923
H	−3.15762364	2.92094391	−1.77983245
H	−2.70360282	5.97965620	−1.68125728
H	−2.15807799	4.78109674	−0.47707767
H	−0.96673647	5.85589483	−1.27461088
H	−2.05098471	5.58585834	−4.10905118
H	−0.35495306	5.48837895	−3.54725523
H	−1.07426331	4.11811566	−4.45510375
H	2.58818493	4.96422300	−1.63934198
H	1.60310959	5.49471875	−0.14371479
S	−0.90642318	0.64758530	−3.15702516
C	−2.15118985	−0.27774584	−4.17617013
O	0.23550056	−0.24708660	−3.10807564
O	−1.60012923	0.87251823	−1.89641765
F	−2.50228122	−1.43227659	−3.59369098
F	−3.27591106	0.45963208	−4.33271853
F	−1.67822078	−0.55465515	−5.40036536

IV

E (TPSSh/def2-TZVP) =	−1860.04298562
$G - E$ (TPSSh/def2-TZVP) =	0.22918934
$H - E$ (TPSSh/def2-TZVP) =	0.30352816
E (DLPNO-CCSD(T)tight/def-TZVPP//TPSSh/def2-TZVP) =	−1852.82612420
E (DLPNO-CCSD(T)tight/def-QZVPP//TPSSh/def2-TZVP) =	−1852.89775926
E (DLPNO-CCSD(T)/CBS//TPSSh/def2-TZVP) =	−1857.65094794

S	0.79519383	3.23571416	−0.05610059
C	1.14844977	1.94650724	−1.09246995
N	1.23112828	0.80515193	−0.41617060
C	1.00571630	0.92968293	0.93847962
C	0.77261281	2.20653601	1.31233206
C	1.35842116	2.11122786	−2.58223382
O	0.57570335	1.25636009	−3.35072742
C	1.45211295	−0.51435055	−1.03588693
C	−1.77815269	2.67803530	−5.17595518
N	−1.88324040	2.17834842	−3.80350469
C	−2.35078411	3.23525469	−2.89856673
C	−2.78607673	1.01805462	−3.74272695
O	−3.22217420	−0.67729624	−0.66864860
S	−2.17825609	−0.02852535	0.09247725
O	−1.65454826	1.20703129	−0.49371925
C	−3.03894177	0.59550527	1.61516692
F	−3.60537403	−0.39798536	2.31228047
O	−1.14379210	−0.90409027	0.64034998
F	−2.16367639	1.22208608	2.44029002
F	−3.99661050	1.48480134	1.30386049
H	−3.79245421	1.28359042	−4.09824952
H	0.97312586	0.04252490	1.54741357
H	2.46584107	−0.84394678	−0.80605491
H	0.71005316	−1.18591741	−0.60573864
H	1.29666967	−0.41636893	−2.10586258
H	0.52991468	2.57236600	2.29524713
H	−2.38886166	0.22360193	−4.37716358
H	−2.85179009	0.65796932	−2.71790050
H	−3.34505431	3.60238179	−3.19368571
H	−2.39709124	2.83613463	−1.88642782
H	−1.65203863	4.07558062	−2.93247327
H	−2.75343974	3.00804994	−5.56354911
H	−1.08708434	3.52320155	−5.20666264
H	−1.39296149	1.88726018	−5.82166982
H	2.41324089	1.89770015	−2.79403076
H	1.19324399	3.17170017	−2.81494137
H	−0.38486478	1.61945706	−3.42471855

5.10 Reactions: Thiamine

5.10.1 Pyruvic Acid

2

E (TPSSh/def2-TZVP) =	−1502.70748854
$G - E$ (TPSSh/def2-TZVP) =	0.30477827
$H - E$ (TPSSh/def2-TZVP) =	0.38527625
E (DLPNO-CCSD(T)/def-TZVPP//TPSSh/def2-TZVP) =	−1495.26889964
E (DLPNO-CCSD(T)/def-QZVPP//TPSSh/def2-TZVP) =	−1495.32721283
E (DLPNO-CCSD(T)tight/def-QZVPP//TPSSh/def2-TZVP) =	
E (DLPNO-CCSD(T)/CBS//TPSSh/def2-TZVP) =	−1500.43859148

C	−2.12505523	−3.28686933	3.37605748
C	−2.21674476	−2.13910031	2.66527038
C	−2.44008579	−2.24117200	1.22687083
N	−2.52959315	−3.50954868	0.66130877
C	−2.42839077	−4.56764269	1.39479351
N	−2.23827097	−4.49710983	2.75639580
C	−1.97677976	−0.81950464	3.31854467
N	−3.18511339	0.04994472	3.37921792
C	−3.10786445	1.38007132	3.78707448
C	−4.31238448	2.01305733	3.67056888
S	−5.49840327	0.92225932	3.04300292
C	−4.38358773	−0.32543454	2.96288832
C	−1.81497860	1.94629059	4.26208336
C	−4.65686597	3.44319019	3.92050073
C	−4.81394162	4.22647211	2.61242761
O	−5.16947728	5.54391122	2.99540124
N	−2.56673685	−1.18856063	0.48624114
C	−2.50904884	−5.93538816	0.79974765
C	−0.36835253	1.59735040	0.54482846
C	−1.76697227	2.22111089	0.67766811
O	−2.79843511	1.40144157	0.75217851
O	−0.03272747	0.74707459	1.35042510
C	0.52634331	2.16079651	−0.51012642
O	−1.85945111	3.42378916	0.79515104
H	−1.49114828	1.46881138	5.19113476
H	−1.02926013	1.80832357	3.51655909
H	−1.92460090	3.01225437	4.44585526
H	−3.87323345	3.90720229	4.52076634
H	−5.58604380	3.52065633	4.49099633
H	−3.88013427	4.19748105	2.04260816
H	−5.60205363	3.76348669	2.00029568
H	−5.08810550	6.12386974	2.22822292
H	−1.23290120	−0.25615080	2.74595217
H	−1.62225786	−0.94796227	4.34228870
H	−1.94501627	−3.31023849	4.44331318
H	−2.14960129	−5.34790258	3.29332475
H	−2.71333720	−1.47038037	−0.48312878
H	−4.59016049	−1.31823447	2.60035533
H	0.57057489	3.24711437	−0.40188115
H	1.51845399	1.71943556	−0.44234864
H	0.09138547	1.96260449	−1.49573229
H	−2.68561776	−5.84909482	−0.26864012
H	−1.57588672	−6.48058019	0.96816386
H	−3.32054914	−6.50833506	1.25718304
H	−2.58011337	0.38938708	0.66641290

$\mathbf{TS_{2\text{-}IV}}$

E (TPSSh/def2-TZVP) =	−1502.67105118
$G - E$ (TPSSh/def2-TZVP) =	0.30668285
$H - E$ (TPSSh/def2-TZVP) =	0.38551399
E (DLPNO-CCSD(T)/def-TZVPP//TPSSh/def2-TZVP) =	−1495.23351348
E (DLPNO-CCSD(T)/def-QZVPP//TPSSh/def2-TZVP) =	−1495.29173501
E (DLPNO-CCSD(T)tight/def-QZVPP//TPSSh/def2-TZVP) =	
E (DLPNO-CCSD(T)/CBS//TPSSh/def2-TZVP) =	−1500.40002855

C	−1.62596927	−2.46226910	3.42718172
C	−1.73826713	−1.63995243	2.35319807
C	−2.64450273	−2.07447363	1.32414220
N	−3.41932320	−3.18308537	1.48507373
C	−3.28148800	−3.91985793	2.54945142
N	−2.37383590	−3.59829432	3.51611238
C	−0.94616182	−0.36257940	2.24727308
N	−1.80781695	0.83109852	2.29322732
C	−2.34119495	1.35068722	3.48946759
C	−3.10586676	2.44603179	3.24016849
S	−3.11501508	2.76213609	1.52730178
C	−2.11789083	1.45312710	1.14231426
C	−2.02583977	0.73662808	4.81207233
C	−3.86481205	3.30083958	4.20250972
C	−5.37814745	3.15779061	4.04157226
O	−5.96086608	3.96306797	5.05225952
N	−2.77328901	−1.43249723	0.18310941
C	−4.10665025	−5.14544775	2.75060363
C	−0.78553285	1.28133264	−0.86634360
C	−2.00755385	1.87899962	−1.59377660
O	−2.61719895	0.95895588	−2.36103904
O	−0.57469835	0.06845908	−1.01430665
C	0.24271746	2.24365625	−0.35902040
O	−2.37550176	3.01868924	−1.49938791
H	−2.47225458	−0.25680422	4.91584700
H	−0.94734397	0.64867017	4.97196157
H	−2.42429244	1.35908676	5.61118057
H	−3.60000845	4.35249042	4.06601496
H	−3.60064972	3.03643307	5.22857935
H	−5.67960898	3.48821805	3.03926036
H	−5.66505275	2.10274528	4.15285712
H	−6.91601750	3.99762899	4.91827308
H	−0.41261465	−0.32329961	1.29556545
H	−0.21444942	−0.31277720	3.05370456
H	−0.95271149	−2.26665339	4.25109434
H	−2.26813588	−4.20451010	4.31947787
H	−3.42744867	−1.79522865	−0.49694446
H	−2.17434195	−0.64921023	−0.08448420
H	−0.21252705	3.17609551	−0.03410081
H	0.83860351	1.79323608	0.43397689
H	0.91275429	2.46454174	−1.19871083
H	−4.74970042	−5.28895282	1.88691564
H	−3.46616841	−6.02289472	2.87558111
H	−4.72316081	−5.04705950	3.64858506
H	−2.03698511	0.16861386	−2.31724434

IV

E (TPSSh/def2-TZVP) =	−1502.71109033
$G - E$ (TPSSh/def2-TZVP) =	0.31101512
$H - E$ (TPSSh/def2-TZVP) =	0.38702161
E (DLPNO-CCSD(T)/def-TZVPP//TPSSh/def2-TZVP) =	−1495.26025163
E (DLPNO-CCSD(T)/def-QZVPP//TPSSh/def2-TZVP) =	−1495.31832597
E (DLPNO-CCSD(T)tight/def-QZVPP//TPSSh/def2-TZVP) =	
E (DLPNO-CCSD(T)/CBS//TPSSh/def2-TZVP) =	−1500.44765562

C	−1.46659366	−2.47690757	3.34597977
C	−1.71540467	−1.61154638	2.33834069
C	−2.81398261	−1.92361821	1.43244197
N	−3.64513468	−2.99224055	1.74592637
C	−3.35668293	−3.78136640	2.72790515
N	−2.25216154	−3.58116141	3.52203862
C	−0.85296104	−0.39629331	2.14435042
N	−1.65226593	0.86048147	2.13065525
C	−2.31150802	1.30964832	3.28034080
C	−3.00576283	2.45517441	3.04344765
S	−2.83020903	2.95227197	1.40045576
C	−1.83883677	1.62363251	1.04507820
C	−2.19749945	0.59415624	4.58334091
C	−3.87153875	3.23318834	3.98124890
C	−5.32641409	2.75806752	3.94973024
O	−6.00657480	3.52468753	4.92550090
N	−3.01819736	−1.21959337	0.36565443
C	−4.21371665	−4.96075012	3.05431374
C	−1.17064370	1.43400741	−0.30764926
C	−1.98683282	2.19951895	−1.38211623
O	−2.02986860	1.57272357	−2.54717028
O	−1.03844648	0.08955397	−0.69448187
C	0.23213777	2.07359696	−0.26948479
O	−2.48533206	3.27921940	−1.17704323
H	−2.76859413	−0.33593606	4.58552880
H	−1.15861549	0.36050523	4.82734110
H	−2.58857174	1.23298831	5.37336028
H	−3.84550605	4.29575064	3.73043607
H	−3.49567478	3.13823761	5.00232399
H	−5.74461681	2.91129602	2.94658564
H	−5.37147968	1.68365195	4.17674783
H	−6.95423085	3.35121137	4.86468545
H	−0.32125846	−0.43965617	1.19702090
H	−0.12853784	−0.30769202	2.95438747
H	−0.65852470	−2.34859422	4.05493569
H	−2.04708147	−4.23146303	4.26698354
H	−3.82667124	−1.57157570	−0.14502011
H	−1.86649136	−0.43331056	−0.35183908
H	0.16373373	3.14049835	−0.05861675
H	0.83902495	1.58571388	0.49470134
H	0.70666140	1.92505027	−1.24007582
H	−5.04983718	−4.99597546	2.36165834
H	−3.63764262	−5.88671967	2.97028211
H	−4.59198147	−4.89208373	4.07815082
H	−1.59579952	0.70104777	−2.40274372

5.10.2 Glyceraldehyde

2

E (TPSSh/def2-TZVP) = −1503.86969071
$G - E$ (TPSSh/def2-TZVP) = 0.32625269
$H - E$ (TPSSh/def2-TZVP) = 0.40988743
E (DLPNO-CCSD(T)/def-TZVPP//TPSSh/def2-TZVP) = −1496.40526614
E (DLPNO-CCSD(T)/def-QZVPP//TPSSh/def2-TZVP) = −1496.46398310
E (DLPNO-CCSD(T)tight/def-QZVPP//TPSSh/def2-TZVP) =
E (DLPNO-CCSD(T)/CBS//TPSSh/def2-TZVP) = −1501.60945903

C	0.76006441	0.12005460	1.56495492
C	0.28058751	−1.01603740	1.01855394
C	1.18029284	−1.85495194	0.23033484
N	2.52084870	−1.46622506	0.13096781
C	2.92206843	−0.37234643	0.68327988
N	2.07815039	0.45256501	1.39847155
C	−1.15895353	−1.39843655	1.11979119
N	−1.34033445	−2.65671706	1.89514859
C	−1.41642067	−2.71157005	3.28508142
C	−1.58004996	−3.99146635	3.72985301
S	−1.63061808	−5.08713082	2.38736046
C	−1.43211736	−3.82961836	1.29261495
C	−1.31829580	−1.47179540	4.10422346
C	−1.75568650	−4.47903253	5.13162046
C	−3.22918737	−4.66311155	5.50118678
O	−3.24351458	−5.05815892	6.86066874
N	0.71633486	−2.90686924	−0.35025854
C	−4.57118885	0.56006347	2.19266589
C	−5.19506012	−0.78068416	1.85519015
C	−6.32794055	−1.11106408	2.84861461
O	−5.75187696	−1.57118923	4.07324538
O	−3.44025782	0.71037497	2.59342288
O	−4.25440451	−1.82971622	1.85070341
H	−0.31845408	−1.03709027	4.02841060
H	−2.04429188	−0.72683923	3.77134017
H	−1.51040757	−1.70417151	5.14979542
H	−1.30689728	−3.76455246	5.82418376
H	−1.23564674	−5.42862522	5.27594867
H	−3.77304565	−3.72089671	5.34796118
H	−3.68254261	−5.42510284	4.85311383
H	−4.15035721	−5.25754440	7.12469013
H	−1.56625768	−1.60449579	0.13075212
H	−1.76964183	−0.63960901	1.60456557
H	0.15355827	0.81666840	2.13006992
H	2.43509069	1.31125673	1.78983558
C	4.34584206	0.07272656	0.57740573
H	1.46522068	−3.36726417	−0.86968800
H	−1.32677326	−3.92761942	0.22409403
H	−5.24498683	1.43212049	2.06187322
H	−5.62694804	−0.69344739	0.85047049
H	−6.90635192	−1.94518522	2.45239801
H	−6.99593311	−0.25995543	3.00548818
H	−5.60678313	−0.82111323	4.66394930
H	−4.25255878	−2.16742761	2.76304524
H	4.90304331	−0.65683819	−0.00355475
H	4.40830790	1.04824275	0.08649155
H	4.79637566	0.16609549	1.56970927

$TS_{2\text{-}IV}$

E (TPSSh/def2-TZVP) =	−1503.84867949
$G - E$ (TPSSh/def2-TZVP) =	0.33000791
$H - E$ (TPSSh/def2-TZVP) =	0.40951082
E (DLPNO-CCSD(T)/def-TZVPP//TPSSh/def2-TZVP) =	−1496.37734246
E (DLPNO-CCSD(T)/def-QZVPP//TPSSh/def2-TZVP) =	−1496.43582357
E (DLPNO-CCSD(T)tight/def-QZVPP//TPSSh/def2-TZVP) =	
E (DLPNO-CCSD(T)/CBS//TPSSh/def2-TZVP) =	−1501.58495415

C	1.16610169	−0.12479839	2.50314515
C	0.05872826	−0.60087739	1.88001227
C	0.23014449	−0.96778564	0.49768213
N	1.45344476	−0.91848205	−0.10292625
C	2.48300422	−0.47872604	0.56029283
N	2.36241120	−0.05561009	1.85071960
C	−1.26835785	−0.69074024	2.58805745
N	−1.66433416	−2.08642319	2.84091105
C	−1.26192063	−2.79238614	3.98903191
C	−1.72894358	−4.06749116	3.97055926
S	−2.65233233	−4.29727473	2.51255129
C	−2.42567868	−2.72027661	1.92970115
C	−0.45924489	−2.13910332	5.06376802
C	−1.57540259	−5.13495558	5.00540378
C	−2.80335624	−5.24976381	5.90825276
O	−2.50518910	−6.26201278	6.85645999
N	−0.77251676	−1.35416668	−0.25872145
C	−4.16055191	−1.32371150	0.67351703
C	−4.96463532	−1.13004932	1.94525342
C	−6.30930315	−0.48086653	1.54464606
O	−6.10552206	0.90039308	1.27353731
O	−3.49853547	−0.42194617	0.17047291
O	−4.30959484	−0.29518139	2.87900656
H	0.52481400	−1.82470467	4.70413653
H	−0.97268666	−1.26406133	5.47219166
H	−0.30119054	−2.83881500	5.88271416
H	−0.70336507	−4.92875816	5.63042321
H	−1.40196754	−6.10339224	4.53025132
H	−2.99581018	−4.28504637	6.39676497
H	−3.68446978	−5.50812912	5.30666235
H	−3.27582439	−6.40306982	7.42013312
H	−2.06777902	−0.24387758	1.99609138
H	−1.22221338	−0.15636660	3.53553331
H	1.15994010	0.22638126	3.52619834
H	3.17442270	0.30732504	2.33262554
C	3.83596944	−0.42621257	−0.06559647
H	−0.58368980	−1.56583045	−1.22859011
H	−1.73753252	−1.39532981	0.07391636
H	−4.38470821	−2.24666367	0.11108165
H	−5.14700976	−2.10112896	2.41023506
H	−6.98798981	−0.52119436	2.39665049
H	−6.77110719	−1.00186265	0.69906744
H	−5.60450056	0.98696189	0.44901943
H	−4.59947410	0.60759517	2.65524838
H	4.27369505	0.57011494	0.03623704
H	4.50473178	−1.14103916	0.42287760
H	3.75235638	−0.68052711	−1.11848598

IV

E (TPSSh/def2-TZVP) =	−1503.89427711
$G - E$ (TPSSh/def2-TZVP) =	0.33493013
$H - E$ (TPSSh/def2-TZVP) =	0.41202465
E (DLPNO-CCSD(T)/def-TZVPP//TPSSh/def2-TZVP) =	−1496.40876891
E (DLPNO-CCSD(T)/def-QZVPP//TPSSh/def2-TZVP) =	−1496.46699124
E (DLPNO-CCSD(T)tight/def-QZVPP//TPSSh/def2-TZVP) =	
E (DLPNO-CCSD(T)/CBS//TPSSh/def2-TZVP) =	−1501.63653316

C	0.93747585	0.15141707	2.53147425
C	−0.11724345	−0.48322379	1.97399671
C	0.08677076	−1.13663001	0.68539001
N	1.38272208	−1.21551636	0.18442439
C	2.35138289	−0.59281374	0.76945066
N	2.15999460	0.13713462	1.91942508
C	−1.46230555	−0.46560623	2.64110676
N	−1.96206177	−1.84558396	2.89306337
C	−1.35304940	−2.69391454	3.82458460
C	−1.89857359	−3.94125590	3.81934736
S	−3.16628807	−4.02125812	2.64838370
C	−2.95827813	−2.40135168	2.20381579
C	−0.26221317	−2.20858299	4.71737180
C	−1.56489627	−5.11969099	4.67544708
C	−2.52459485	−5.27488325	5.85734780
O	−2.06324469	−6.39841289	6.58340551
N	−0.90666856	−1.63706737	0.02489404
C	−3.93574944	−1.75214413	1.25576504
C	−5.06955843	−1.09767182	2.09100859
C	−6.03296345	−0.32651479	1.17378946
O	−5.48429368	0.94926445	0.86931992
O	−3.36930666	−0.79417142	0.40873136
O	−4.52675103	−0.21348972	3.05154358
H	0.66145979	−2.03019663	4.16400255
H	−0.54820063	−1.28189893	5.22044730
H	−0.06380101	−2.95409601	5.48467122
H	−0.54794780	−5.01867574	5.05842627
H	−1.58892392	−6.03779451	4.08364618
H	−2.51238382	−4.36256643	6.46874917
H	−3.54850431	−5.42281223	5.48841003
H	−2.65726332	−6.56438799	7.32612802
H	−2.21564650	0.01617377	2.02515567
H	−1.42091013	0.04518234	3.60229122
H	0.87896838	0.69146380	3.46800461
H	2.93747062	0.62950758	2.33484099
C	3.74215324	−0.63369590	0.22446239
H	−0.57381075	−2.05436204	−0.84360349
H	−2.44236529	−1.12309199	0.13844107
H	−4.38959180	−2.57331231	0.67912699
H	−5.60461920	−1.89514621	2.61600502
H	−6.96353337	−0.13739888	1.70940537
H	−6.25429746	−0.90091586	0.26863527
H	−4.70916843	0.78443076	0.30725885
H	−4.64504851	0.67287247	2.65752846
H	4.08728671	0.37478307	−0.02082874
H	4.43289447	−1.05596327	0.95976978
H	3.75412003	−1.24414230	−0.67408008
